# Germline and somatic mutations in the pathology of pineal cyst: A whole‐exome sequencing study of 93 individuals

**DOI:** 10.1002/mgg3.1691

**Published:** 2021-05-04

**Authors:** Yuanqing Yan, Rebecca Martinez, Maria N. Rasheed, Joshua Cahal, Zhen Xu, Yanning Rui, Krista J. Qualmann, John P. Hagan, Dong H. Kim

**Affiliations:** ^1^ Vivian L. Smith Department of Neurosurgery McGovern Medical School The University of Texas Health Science Center at Houston Houston TX USA; ^2^ Memorial Hermann Hospital Mischer Neuroscience Institute Houston TX USA

**Keywords:** germline mutation, pineal cyst, somatic mutation, whole exome sequencing

## Abstract

**Background:**

Pineal cyst is a benign lesion commonly occurring in people of any age. Until now, the underlying molecular alterations have not been explored.

**Methods:**

We performed whole exome sequencing of 93 germline samples and 21 pineal cyst tissue samples to illustrate its genetic architecture and somatic mutations. The dominant and recessive inheritance modes were considered, and a probability was calculated to evaluate the significance of variant overrepresentation.

**Results:**

By analyzing pineal cyst as a Mendelian disease with a dominant inheritance pattern, we identified 42,325 rare germline variants, and NM_001004711.1:c.476A>G was highly enriched (FDR<0.2). By analyzing it as a recessive disorder, we identified 753 homozygous rare variants detected in at least one pineal cyst sample each. One *STIM2* rare variant, NM_001169117.1:c.1652C>T, was overrepresented (FDR<0.05). Analyzing at a gene‐based level, we identified a list of the most commonlymutated germline genes, including *POP4*, *GNGT2* and *TMEM254*. A somatic mutation analysis of 21 samples identified 16 variants in 15 genes, which mainly participated in the biological processes of gene expression and epigenetic regulation, immune response modulation, and transferase activity.

**Conclusion:**

These molecular profiles are novel for this condition and provide data for investigators interested in pineal cysts.

## Graphical abstract

Whole exome sequencing of 93 germline samples and 21 pineal cyst tissue samples was performed to study the molecular mechanism of pineal cysts. A list of overrepresented germline genetic variants and somatic mutations were identified for future validation.

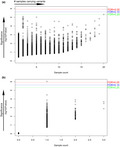

## 1 INTRODUCTION

The pineal gland is a tiny endocrine organ situated in the epithalamus, near the center of the brain, and known as the “third eye” (Schiller, 1995). It synthesizes and secretes melatonin to maintain physiological circadian rhythm and regulate the reproductive hormones in mammals (Borjigin et al., 2012). A study in rat pineal gland tissue demonstrates that it is a heterogeneous organ and consists of at least nine different cell types with different gene expression profiles and functionality (Mays et al., 2018). Pinealocytes are the most abundant cell type, accounting for 90–95% of the pineal gland cells, and participate in melatonin synthesis (Moller & Baeres, 2002).

A pineal cyst is a benign lesion in the pineal gland that can occur in people of any age (Bosnjak et al., 2009). The occurrence of pineal cyst is common, with its incidence in high‐resolution magnetic resonance imaging (MRI) ranging from 1% to 23% (Nevins et al., 2016). The incidence of pineal cyst is higher in females than in males, especially for those in the 21 to 30 year age group (Sawamura et al., 1995). Pineal cysts are usually asymptomatic and could remain so for many years (Bosnjak et al., 2009). However, some affected individuals report symptoms which include headache, visual and oculomotor disturbances, obstructive hydrocephalus, Parinaud syndrome, pineal apoplexy and in rare cases sudden death (Bosnjak et al., 2009; Michielsen et al., 2002; Milroy & Smith, 1996; Na et al., 2014; Nevins et al., 2016). The size of asymptomatic cysts is usually less than 10 mm in diameter, while that reported in symptomatic cysts ranges from 7 mm to 45 mm (Bosnjak et al., 2009). Few pineal cyst patients require surgery; however, surgical intervention should be considered in symptomatic patients, such as those suffering from hydrocephalus, progression of neurologic symptoms, or increasing cyst size (Bosnjak et al., 2009).

The cause of pineal cyst is unknown and the potential for genetic predisposition to this disease has not been studied. The overall aims of this study are to seek the germline genetic variants that may cause the pathology of pineal cysts, assuming pineal cyst is a Mendelian disease, and to delineate the acquired somatic alteration profile of pineal cyst tissue via somatic mutation analysis. From whole exome sequencing in 93 samples, we identified the overrepresented germline genetic variants and genes. Somatic mutation analysis of 21 pineal cyst tissue samples revealed the potential acquired molecular alterations in pineal cyst pathology.

## 2 MATERIALS AND METHODS

### 2.1 Ethical compliance

This study was approved by the Committee for the Protection of Human Subjects at the University of Texas Health Science Center at Houston.

### 2.2 Human patients

Blood samples were collected as part of the Neurosciences Research Repository (NRR) in the Vivian L. Smith Department of Neurosurgery at McGovern Medical School. Germline DNA was collected via blood samples. Blood was collected by venipuncture of an antecubital vein in K_2_EDTA vacutainer tubes (BD) and processed within an hour of draw. Blood was centrifuged at 4°C, 800 *g* for 10 minutes and buffy coat was harvested and frozen at −80°C until DNA extraction. Residual pineal cyst tissues from patients who required surgery were collected and flash frozen in liquid nitrogen. Tissue samples were frozen at −80°C until DNA extraction.

### 2.3 DNA extraction

DNA from human buffy coat samples was extracted by QIAamp DNA Blood Mini Kit (Cat No. 51106, Qiagen Inc). In brief, protease was added to the buffy coat followed by cell lysis. Released DNA was bound to QIAamp spin column and eluted into 1X TE buffer following the product's instruction.

### 2.4 Whole exome sequencing and germline variants calling

All samples were sequenced at the Genome Technology Access Center at the Washington University in St. Louis on the Illumina NovaSeq 6000 platform, with the use of 151 base pair paired‐end reads. Exome was captured by xGen Exome Research Panel v2 kit (Integrated DNA Technologies Inc.). Sequencing reads were mapped to human genome build hg19 using the BWA tool (Li & Durbin, 2009, 2010), after the reads quality control and adapters trimming. Reads pairs having the same unclipped alignment start and unclipped alignment end were marked using MarkDuplicates program and then subjected to base quality score recalibration in the Genome Analysis Toolkit (GATK version 4.1.4.0; McKenna et al., 2010). Variants call were performed by HaplotypeCaller program with GVCF mode in GATK following the best practices pipeline, which detected single nucleotide variants (SNVs) and small insertion or deletion (indel) variants. Variants were further recalibrated by the standard Variant Quality Score Recalibration (VQSR) tool, which uses machine learning algorithm to generate an adaptive error model and filter out sequencing artifacts. Variants passing VQSR metric remained for further analysis.

### 2.5 Quality control and variants/genotypes filtering

To obtain high quality variants, quality control and variant/genotype filtering were performed. Specifically, the genotypes with genotype quality (GQ) score less than 20 were set to missing. The variants with a minimum depth of coverage of less than 10 were excluded from further analysis. We excluded variants with p value of Hardy Weinberg Equilibrium (HWE) test less than 1e‐6. Genotypes with heterozygous variants and allele balance of less than 0.2 or larger than 0.8 were set as missing. Only the genotypes with a call rate of 1.0 were kept. In addition, we manually checked the variants in low‐complexity regions in Integrative Genomics Viewer (IGV), especially the loci in which multiple variants were detected, and excluded any sequencing artifacts(Robinson et al., 2011).

### 2.6 Variants annotation and rare variants selection

The functional annotation of the variants was performed by the ANNOtate VARiation (ANNOVAR) program (Wang et al., 2010). This study focused on rare variants in protein‐coding regions. Therefore, only the variants with a population minor allele frequency (MAF) less than 0.05 were retained. Population MAF refers to the maximum value of MAF in the gnomAD genomes and exomes datasets. Synonymous SNVs were removed from further analysis. The variants in locations not expected to cause significant changes in protein function (UTR5, ncRNA_intronic, intronic, UTR3, intergenic, upstream, downstream, upstream;downstream, UTR5;UTR3 and ncRNA_exonic) and variants within pseudogenes were excluded.

### 2.7 Significance of rare variant overrepresentation in the cohort

We calculated the probability of variant overrepresentation in our cohort assuming the pineal cyst was either a dominant (including X‐linked dominant) or recessive disease. Population MAF in the gnomAD database was used to denote the probability of the variant to be detected in the general population. GnomAD reports MAFs for each variant from two datasets: gnomAD exomes and gnomAD genomes. The larger of these two MAF values was used and denoted as $P_{\text{maf}}$. If the variant's MAF was not provided in either dataset, it was manually set to 3e‐6(smaller than the minimum MAF (3.976e‐6) deposited in gnomAD). By analyzing pineal cyst as a dominant disease, the probability of an affected individual carrying the variant was calculated as $P_{\text{ind}}=2P_{\text{maf}}\left(1‐P_{\text{maf}}\right)+P_{\text{maf}}^{2}$. By analyzing pineal cyst as a recessive disease, the probability of an affected individual carrying the variant was calculated as $P_{\text{ind}}=P_{\text{maf}}^{2}$. In this cohort, we calculated the number of individuals carrying the variants as $N_{\text{Carrying}}$ (For the assumption of recessive disease, $N_{\text{Carrying}}$ refers to the number of individuals carrying homozygous variants). The probability to detect such $N_{\text{Carrying}}$ samples carrying each variant was computed as:

$P\left(N_{\text{Carrying}};N_{\text{Total}},P_{\text{ind}}\right)=\frac{N_{\text{Total}}!}{\left(N_{\text{Total}}‐N_{\text{Carrying}}\right)!N_{\text{Carrying}}!}{\left(P_{\text{ind}}\right)}^{N_{\text{Carrying}}}{\left(1‐P_{\text{ind}}\right)}^{\left(N_{\text{Total}}‐N_{\text{Carrying}}\right)}$.

Here, $N_{\text{Total}}$ denotes the total number of patients in this cohort. To adjust for multiplicity, the Bonferroni method was used to control for false discovery rate (FDR).

### 2.8 Gene‐based collapsing analysis

A gene‐based analysis is an alternative approach to dissect the disease's genetic architecture. In this study, variants with indel mutation (frameshift insertion, stop gain, frameshift deletion, nonframeshift deletion, nonframeshift insertion, and stop loss) and nonsynonymous SNVs with damaging effects predicted by at least two of the four algorithms were selected for gene level analysis. The four algorithms to predict damaging scores were: SIFT (Sorting Intolerant From Tolerant; Ng & Henikoff, 2003), Polyphen2 HVAR(Polymorphism Phenotyping v.2; Adzhubei et al., 2010), CADD (Combined Annotation Dependent Depletion; Rentzsch et al., 2019), and FATHMM‐MKL (Functional Analysis Through Hidden Markov Models–Multiple Kernel Learning; Shihab et al., 2015). For CADD, a predicted damaging score larger than 15 was considered damaging. The total number of damaging variants identified in a particular gene were then aggregated by the cohort allelic sum test (CAST) method (Morgenthaler & Thilly, 2007). CAST is a widely used approach in genetics studies by collapsing all rare variants within a region, such as the exons of a gene, into a binary value for each subject to indicate whether the individual has any rare variants detected within that region (Morgenthaler & Thilly, 2007). To account for the differences in variant detection frequency that occur based on gene length, we normalized the frequency of sample count (number of rare variants detected) for each gene based on its protein length, which was downloaded from the UniProt database.

### 2.9 Somatic variants calling

Sequencing reads were mapped to human genomics build hg19 using the BWA mapping algorithm (Li & Durbin, 2009, 2010). Duplicate alignments in the BAM file were marked by MarkDuplicates and sorted by the coordinate in the GATK bundle (McKenna et al., 2010). The sorted BAM files were then subjected to base recalibration by the BaseRecalibrator program. The germline sequencing data of 93 pineal cyst buffy coat samples were used for the panel of normal (PON) creation in the Mutect2 tool. In this study, 21 pineal cyst tissue samples and matched buffy coat germline samples were used for the somatic variant calling. The somatic variants were called by the Mutect2 tool following the best practices pipeline. Germline variants were removed based on the gnomAD database, PON in our 93 samples, and the matched germline samples of each tissue as conducted in Mutect2 with the default cutoff value set. The remaining variants were further subjected to quality control and filtered by sequence context artifacts and contamination. The functional annotation of the variants was performed by the ANNOVAR program (Wang et al., 2010). The final list of variants was subjected to manual checkup in IGV and obvious sequencing artifacts were excluded from further analysis.

### 2.10 Statistical analysis

The probability of variant overrepresentation was calculated based on the binomial distribution assumption. All statistical analysis and graph production were conducted in R (version 3.5.1).

## 3 RESULTS

### 3.1 Patient characteristics

All patients included in the study were diagnosed with pineal cyst at the University of Texas Health Science Center at Houston and presented for elective surgery at Memorial Hermann Hospital (Houston, Texas, USA) from 2015 to 2019. Of the 93 patients, the majority (n = 80, 86.02%) are female; only 13 (13.978%) are male. The average age was 35.06 years old (standard deviation (SD) = 9.26, range = 18–68). Ninety‐one (97.85%) patients are Caucasian.

### 3.2 Overrepresentation of germline variants

The 93 buffy coat samples were subjected to whole exome sequencing with an average depth of coverage of 85X and an average of 77% of the target base pairs having at least 30X coverage. In this study, only rare variants were considered. A total of 42,325 rare variants remained after the low quality variant/genotype filtering (Table S1).

**FIGURE 1:**
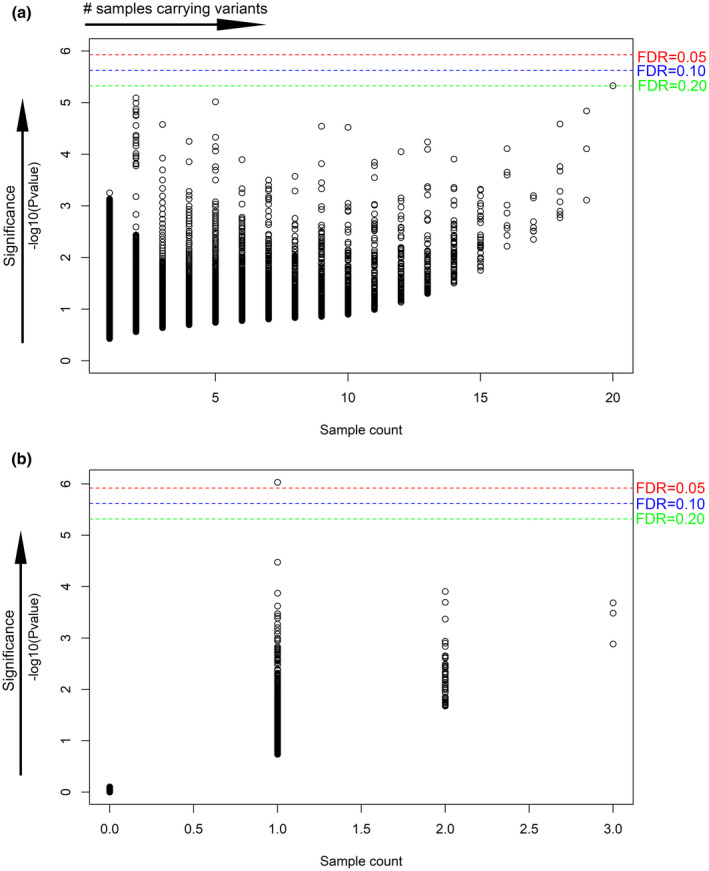
Scatter plot of p value vs sample count. (a) Scatter plot for dominant disorder. (b) Scatter plot for recessive disorder. Each point in the plot represented as one variant, and the x‐axis denoted the number of samples carrying this variant. The y‐axis is the –log10 p value calculated from a binomial probability distribution. A Bonferroni correction was applied, and three different FDR cutoffs were provided. The red dashed line denoted the FDR equal to 0.05, while the blue and green dashed lines denoted the value of 0.1 and 0.2, respectively

When assuming pineal cyst as a dominant genetic disorder (including autosomal and X‐linked dominant), we assumed disease‐causing variants would be overrepresented in our disease‐centralized cohort. For each unique variant, we counted the number of patient samples that carried the variant. The sample count for each variant ranged from 1 to 20 (Figure 1a, Table S2). 29,696 (70.15%) variants were detected in only one sample each (Table S2). We identified one variant that was detected in 20 samples and three variants that were detected in 19 samples (Figure 1a, Table S2). In addition, we used a series of more stringent MAF cutoffs to obtain rarer variants (Table S2). With a population MAF cutoff of 0.0005, there were five variants that were detected in three samples, 163 variants detected in two samples and 14,535 variants detected in one sample each (Table S2). To obtain an unbiased estimation of variant overrepresentation, we calculated the probability of variant detection under the null hypothesis of no variant enrichment in this cohort (Figure 1a). Using the Bonferroni correction method and setting FDR cutoff to 0.2 (i.e. the result is likely to be valid 4 out of 5 times), we found one overrepresented variant in *OR4D9* (Olfactory Receptor Family 4 Subfamily D Member 9; Figure 1a, Table 1). The NM_001004711.1:c.476A>G variant is located on chromosome 11 at position 59,282,861 (Table 1). This variant was detected in 20 patient samples and the MAFs in gnomAD genomes and exomes database were 0.0329 and 0.0359, respectively (Table 1). The variant results in the amino acid change of p.Q159R which is predicted to be severely detrimental to the protein's function by three of four programs (Table 1). Through the interaction with the UniProt database, we evaluated the effects of variant on protein motifs/domains and found that this variant may affect protein's extracellular domain. IGV inspection of this variant showed it had a good depth of variant coverage (Figure S1).

**TABLE 1:** Rare variants overrepresented in pineal cyst

Variant	Chr	Start	End	Ref	Alt	Gene	AAChange	Gnomad genomes MAF	Gnomad exoms MAF	#Sample carrying variant	Raw P	FDR	SIFT	Polyphen2_HVAR	CADD	fathmm.MKL_coding
Dominant disorder																
NM_001004711.1:c.476A>G	11	59282861	59282861	A	G	OR4D9	p.Q159R	0.0329	0.0359	20	4.72E‐06	0.1997	D	D	21	N
Recessive disorder																
NM_001169117.1:c.1652C>T	4	27019495	27019495	C	T	STIM2	p.P551L	.	0.0001	1	9.30E‐07	0.0386	T	B	14.31	D

When assuming pineal cyst as an autosomal recessive disorder, we assumed homozygous disease‐causing variants would be overrepresented in our disease‐centralized cohort. After excluding variants located on the X chromosome, 41,491 variants remained and 753 homozygous variants were detected in at least one patient sample at a population MAF cutoff of 0.05 (Figure 1B, Table S3). Furthermore, three homozygous variants were detected in three samples each and 54 homozygous variants were detected in two samples each (Figure 1B, Table S3). Other population MAF cutoffs were also used to define rarer variants, which identified one homozygous variant detected in one sample at a population MAF cutoff equal to 0.0005 (Table S3). A probability calculation to evaluate the variant overrepresentation in this cohort revealed a significant homozygous variant in the *STIM2* (Stromal Interaction Molecule 2; OMIM: 610841) gene (Figure 1B, Table 1). This variant, NM_001169117.1:c.1652C>T, is a nonsynonymous SNV located on chromosome 4 at the position of 27,019,495, which causes the amino acid change of p.P551L (Figure S2). Functionality of histidine‐ and proline‐rich (His/Pro‐rich) domain of STIM2 could be affected by this variant. The MAF of this variant in the gnomAD exomes database was 0.0001 and it was not present in the gnomAD genomes database (Table 1). IGV inspection revealed good depth of variant coverage (Figure S2).

### 3.3 Gene‐based analysis in pineal cyst

Potential genetic alterations resulting in pineal cyst were also analyzed at the gene level by aggregating identified rare variants using the CAST approach. A total of 11,035 mutated genes were identified and the frequencies of mutations were counted (Table S4). Previously, we observed 20 samples carrying the *OR4D9* variant, NM_001004711.1:c.476A>G, in the variant level analysis, while any rare variant in *OR4D9* was detected in 21 patient samples (Table 1, Table S4). In a gene‐based level analysis, larger genes tend to have more variants detected. In order to avoid this bias, we normalized the frequency of mutations with the protein length. We defined this as the mutation fraction, which ranged from 0.00022 to 0.15556. The five most frequently mutated genes were *POP4* (Processing Of Precursor 4 Homolog, Ribonuclease P/MRP Subunit; OMIM:606114), *GNGT2* (G Protein Subunit Gamma Transducin 2; OMIM:139391), *TEME254* (Transmembrane Protein 254), *GPHB5* (Glycoprotein Hormone Subunit Beta 5; OMIM:609652) and *DLC1* (Deleted in Liver Cancer 1 Rho GTPase Activating Protein; OMIM:604258; Figure 2).

**FIGURE 2:**
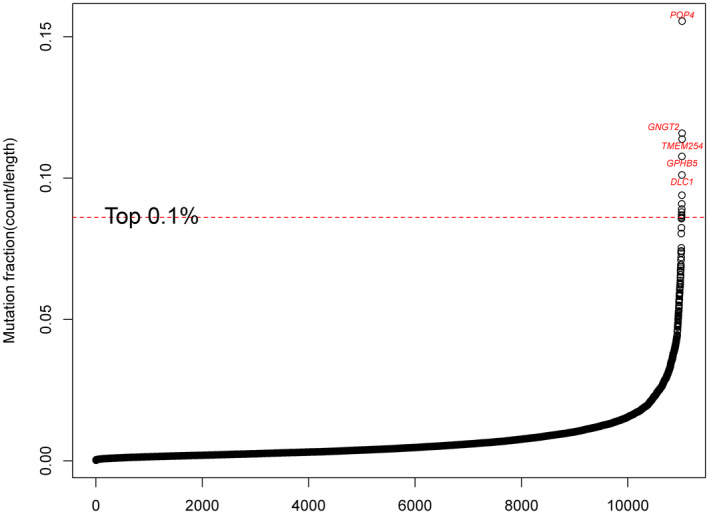
Scatter plot of mutation fraction. Frequency of gene mutation was counted and normalized to the protein length to obtain mutation fraction. The red dashed line represented the cutoff of top 0.1%. Genes above red dashed line were the one having highest mutation fraction

Without clear evidence of an inherited genetic predisposition for pineal cysts, using a population MAF cutoff of 0.05 to identify potential disease‐causing rare variants could be considered arbitrary. Based on the common hypothesis that very rare genetic variants have a large effect size on disease(Gibson, 2012), we performed additional analyses by setting a more stringent population MAF cutoff of 0.001 to define very rare variants and repeated our aggregation via CAST approach. With this stringent threshold, a total of 7,139 mutated genes were identified with the mutation fraction ranging from 0.00017 to 0.04444 (Sup. Figure 3). The ten most frequently mutated genes in this analysis were *POP4*, *MTRNR2L3* (MT‐RNR2 Like 3), *MTRNR2L9* (MT‐RNR2 Like 9), *EFCAB8* (EF‐Hand Calcium Binding Domain 8), *PKIB* (CAMP‐Dependent Protein Kinase Inhibitor Beta; OMIM:606914), *TOR1AIP2* (Torsin 1A Interacting Protein 2; OMIM:614513), *POP1* (POP1 Homolog, Ribonuclease P/MRP Subunit; OMIM: 602486), *ARSD* (Arylsulfatase D; OMIM:300002), *NOS2* (Nitric Oxide Synthase 2; OMIM:163730) and *TLCD1* (TLC Domain Containing 1; Figure S3).

**FIGURE 3:**
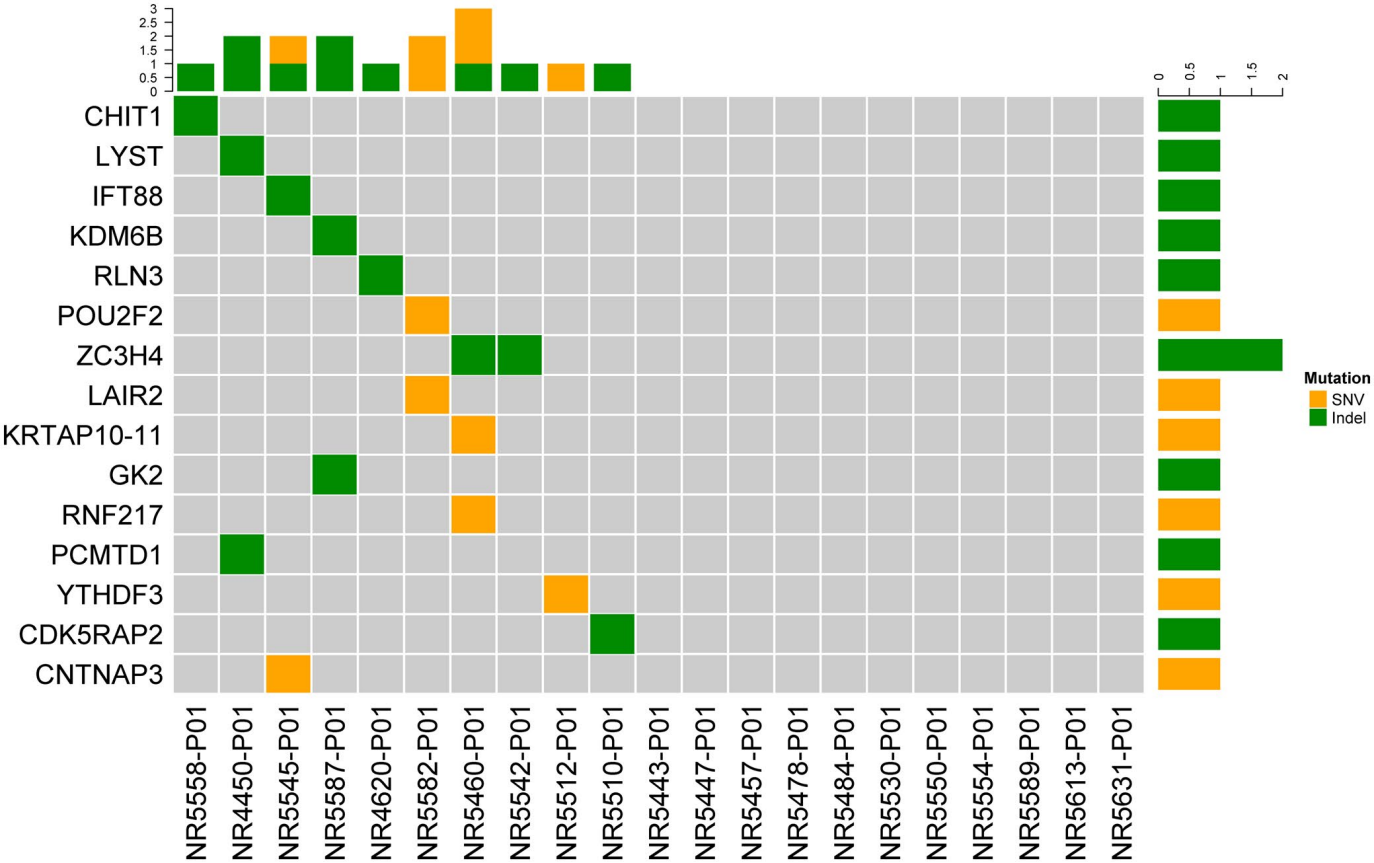
Heatmap of somatic mutation in 21 pineal cyst. 15 gene mutations were plotted with SNV colored in orange and indel colored in green. Each row represented one mutant gene, while each column represented one sample. Samples without mutation were in grey color

### 3.4 Somatic mutation in pineal cyst

The pathological phenotype of pineal cyst could be attributed to inherited genetic alterations and/or acquired somatic mutations. To evaluate the presence of somatic mutations, we sequenced 21 pineal cyst tissue samples with matched germline controls. The average depth of coverage for the somatic mutations was 155X and an average of 93.6% of the target base pairs had at least 30X coverage. A total of 16 variants in 15 genes were identified, including six nonsynonymous SNVs, one splicing mutation, one frameshift deletion, four nonframeshift deletions, two nonframeshift substitutions, one stop gain, and one stop loss (Figure 3). These 16 variants were detected in ten pineal cyst tissue samples (Figure 3, Table S5). *ZC3H4* (Zinc Finger CCCH‐Type Containing 4), responsible for epithelial to mesenchymal transition and inflammation activation in pulmonary macrophages during silicosis (Jiang et al., 2019; Yang et al., 2018), was the only gene with somatic mutations detected in two samples, while each of the other genes had a mutation detected in only one sample (Figure 3). Two immune system process genes, *CHIT1* (Chitinase 1; OMIM:600031) and *LAIR2* (Leukocyte Associated Immunoglobulin Like Receptor 2; OMIM:602993), were altered in the pineal cyst tissue via frameshift deletion and nonsynonymous SNV, respectively. A nonframeshift deletion was observed in *LYST* (Lysosomal Trafficking Regulator; OMIM:606897), which regulates the function of lysosomes and lysosome‐related organelles (Ji et al., 2016). A nonframeshift substitution was observed in *CDK5RAP2* (Cyclin‐Dependent Kinase 5 Regulatory Subunit Associated Protein 2; OMIM:608201), which mediates centriole engagement and microtubule nucleation (Table S5). Interestingly, we found a stop loss alteration in *RLN3* (Relaxin 3; OMIM:606855), a gene modulating key neurobiological functions in mammal which includes circadian rhythm, response to stress, and metabolism(Smith et al., 2011). A nonsynonymous SNV in *CNTNAP3*(Contactin Associated Protein Family Member 3;OMIM:610517), and a splicing in *IFT88*(Intraflagellar Transport 88;OMIM:600595) were also observed. We also found mutations in genes responsible for gene expression and epigenetic regulation: *KDM6B* (Lysine Demethylase 6B; OMIM:611577), *YTHDF3* (YTH N6‐Methyladenosine RNA Binding Protein 3; OMIM:618669) and *POU2F2* (POU Class 2 Homeobox 2; OMIM:164176; Figure 3). Finally, somatic mutations were found in three genes encoding proteins with transferase activity, *PCMTD1* (Protein‐L‐Isoaspartate (D‐Aspartate) O‐Methyltransferase Domain Containing 1), *RNF217* (Ring Finger Protein 217; OMIM:618592), and *GK2* (Glycerol Kinase 2; OMIM:600148; Figure 3).

## 4 DISCUSSION

To the authors’ knowledge, this is the first study to perform whole exome sequencing in individuals with pineal cysts, both from germline DNA and cyst tissue itself. It is currently unknown whether there is a genetic contribution in individuals with pineal cysts. Therefore, the analysis was performed under assumptions of a de novo dominant or recessive model. We identified a list of candidate genes and variants which were overrepresented in our cohort. While we are uncertain as to the significance of these findings, they do serve as data for future validation. In addition to or alternatively from having an inherited genetic etiology, pineal cysts could be derived from acquired mutations accumulated over time or due to environmental exposures. Sequencing 21 pineal cyst tissue samples provided the somatic mutation profiles to gain more insights into this disease. Assuming the pineal cyst trait as a dominant condition, the *OR4D9* variant, NM_001004711.1:c.476A>G, was highly overrepresented with a FDR less than 0.2. *OR4D9* encodes an olfactory receptor which participates in the neuronal response to trigger the perception of a smell in the nose(Malnic et al., 2004). Gene expression of *OR4D9* in the pineal gland is detectable as provided from BioGPS database(Wu et al., 2009). The presence of OR4D9 protein in the pineal gland could invoke other distinct signaling pathways in addition to its known G protein‐mediated transduction signaling function(Dalesio et al., 2018). When considering pineal cyst as a recessive condition, the *STIM2* variant, NM_001169117.1:c.1652C>T, was detected with a FDR less than 0.05. Expression of *STIM2* in the pineal gland was reported to be detectable (Wu et al., 2009). STIM2 protein modulates the biological function through the calcium signaling pathway in many tissues (Nelson & Roe, 2018), although its role in the pineal gland has not yet been reported.

An alternative way to gain insights into the landscape of genetic alterations in pineal cyst is to examine the presence of rare variants at the gene‐based level by aggregating all detected rare variants. In this study, we preselected the damaging variants for each gene and then applied the CAST approach for the variant aggregation. The pros of this approach are the resulting comprehensive understanding of the molecular alterations architecture by combining all the damaging effects together. However, large genes tend to have more variants detected by random chance. In order to avoid this bias, we normalized gene mutation frequency by its corresponding protein length to generate the mutation fraction. Based on the hypothesis that rare variants have a large genetic effect size (Gibson, 2012), we performed the gene based level analysis using two different population MAF cutoffs to define rare variants: 0.05 and 0.001. The purpose of setting these two MAF cutoffs was to both capture the largest number of potential disease causing rare variants by sacrificing the consideration of genetic effect size (0.05 cutoff), and aggregating the variants with largest potential genetic effect size by sacrificing the number of potential disease causing variants (0.001 cutoff).

Although there was no genetic study reported in pineal cyst, some previous studies did observe an association of retinoblastoma with pineal cyst (Gupta et al., 2016; Popovic et al., 2007; Rodjan et al., 2010). This implied the potential genetic mutation of *RB1* gene or 13q deletion in the pathology of pineal cyst (Amare et al., 1999; D'Elia et al., 2013; Kivela et al., 2003; Parma et al., 2017; Popovic et al., 2007). We revisited our WES data and found one patient (out of 93 samples) carrying *RB1* gene mutation (Table S1). This is a nonsynonymous SNV, resulting in amino acid change of p.R656W (Table S1). In addition, *RB1* is a tumor suppressor gene and plays significant role in cancer biology(Dyson, 2016; Knudsen et al., 2020). Interestingly, we targeted *DLC1*, a tumor suppressor gene(Zhang & Li, 2020), from our five most frequently mutated genes in the gene‐based analysis (Table S4). *DLC1* encodes a GTPase‐activating protein which is one of the Rho family members(Zhang & Li, 2020). In multiple cancers, expression level of DLC1 is downregulated which leads to the continual unregulated cell proliferation, migration and invasion (Jensch et al., 2018; Wang et al., 2020; Wu et al., 2018).

Somatic mutation analysis revealed the acquired mutations in pineal cyst tissue and was a complementary profile to the inherited genetic alterations. This allowed for a better understanding of disease pathology. We identified 15 mutated genes, many of which had biological processes of gene expression and epigenetic regulation (*KDM6B*, *YTHDF3* and *POU2F2*), immune response modulation (*CHIT1*, *LAIR2* and *LYST*), and transferase activity (*PCMTD1*, *RNF217* and *GK2*). Of these genes, *LYST* encodes a protein that regulates the function of lysosomes and its mutation is associated with the lysosomal storage disorder Chediak‐Higashi syndrome (Gil‐Krzewska et al., 2016; Song et al., 2020). Biological functionality of *LYST* in the pineal gland is still under study, but it was shown that the number of lysosomes in adult rat pineal gland tissue increases during aging(Calvo & Boya, 1984). In chicken pineal gland tissue, the number and activity of lysosomes were affected by the light‐dark cycle, with larger numbers and greater activity observed during the light period(Ohshima & Matsuo, 1989). Another interesting mutated gene identified in our study is *RLN3*, an abundant neuropeptide which plays central physiological and pharmacological functions in brain(Ma et al., 2017). One of the key biological functions involving *RLN3* is the coordination of circadian rhythm and the circadian system in mammals, which is in line with the physiological function of pineal gland (Smith et al.,^2011^, ^2012^). We also identified *KDM6B*, a histone modifier, which epigenetically regulates gene expression by catalyzing the demethylation of H3k27me2/3(Burchfield et al., 2015), and *YTHDF3*, which binds N^6^‐methyladenosine‐containing RNA and facilitates its translation and decay (Shi et al., 2017). Being the transcription factor that specifically binds to the octamer motif, somatic mutation *POU2F2* was detected in one pineal cyst sample (Scheidereit et al., 1988). In addition, our observation of gene mutations involved in immune response modulation and transferase activity, implied that pineal cyst could be caused by a multitude of factors. Of note, one limitation of this analysis is that somatic variants called in this cohort have a low variant allele fraction (Table S5). This low coverage could be due to the known heterogeneity of the pineal gland, which includes at least nine different cell types (Mays et al., 2018). Alternatively, it could also result from heterogeneity within the pineal cyst itself, with only a small subset of cells carrying the somatic mutations.

Although there are many merits to our study, it also has limitations. One limitation is the small sample size and the lack of matched healthy controls. To alleviate this drawback, we calculated the probability of overrepresentation of variants to capture the best potentially disease‐causing genetic alterations in this cohort. We acknowledge that the overrepresented variants are not necessarily the disease‐causing agents. Another limitation is the unknown incidence of genetic predisposition to pineal cyst or its most likely inheritance pattern. Alternative etiologies that were not evaluated by our current analyses include autosomal recessive inheritance via compound heterozygous mutations, X‐linked recessive mutations, or more complex genetic origins, such as multigenic or multi‐factorial inheritance. Alternatively, there may be no inherited genetic component involved.

This study is in a discovery phase attempting to uncover any potential disease‐causing molecular alterations, assuming pineal cyst is a genetically inherited disease. In conclusion, we provide a list of potential candidates, at both the variants level and the gene‐based level, using both dominant and recessive inheritance models, for future validation. Our study provides the first analysis of this disease cohort and provides a discovery cohort which may be utilized in future validation studies.

## CONFLICT OF INTERESTS

The authors declare that they have no competing interests.

## AUTHOR CONTRIBUTIONS

Y.Y. and D.H.K designed the study, performed the statistical analysis, interpreted the results, and wrote the paper. M.N.R., J.C., Z.X., Y.R., and J.P.H., performed the DNA extraction and interpreted the results. R.M. organized clinical data, collected the samples, and interpreted the results. K.J.Q. collected the samples and interpreted the results.

## Supporting information

Fig S1

Fig S2

Fig S3

Table S1

Table S2

Table S3

Table S4

Table S5

## Data Availability

All data are available in the manuscript or the supplementary materials.
