# Supplementary figures and images for: Germline and somatic mutations in the pathology of pineal cyst: A whole‐exome sequencing study of 93 individuals
Source: Mol Genet Genomic Med. 2021 May 4;9(6):e1691. doi: 10.1002/mgg3.1691 (PMC8222845; doi:10.1002/mgg3.1691)

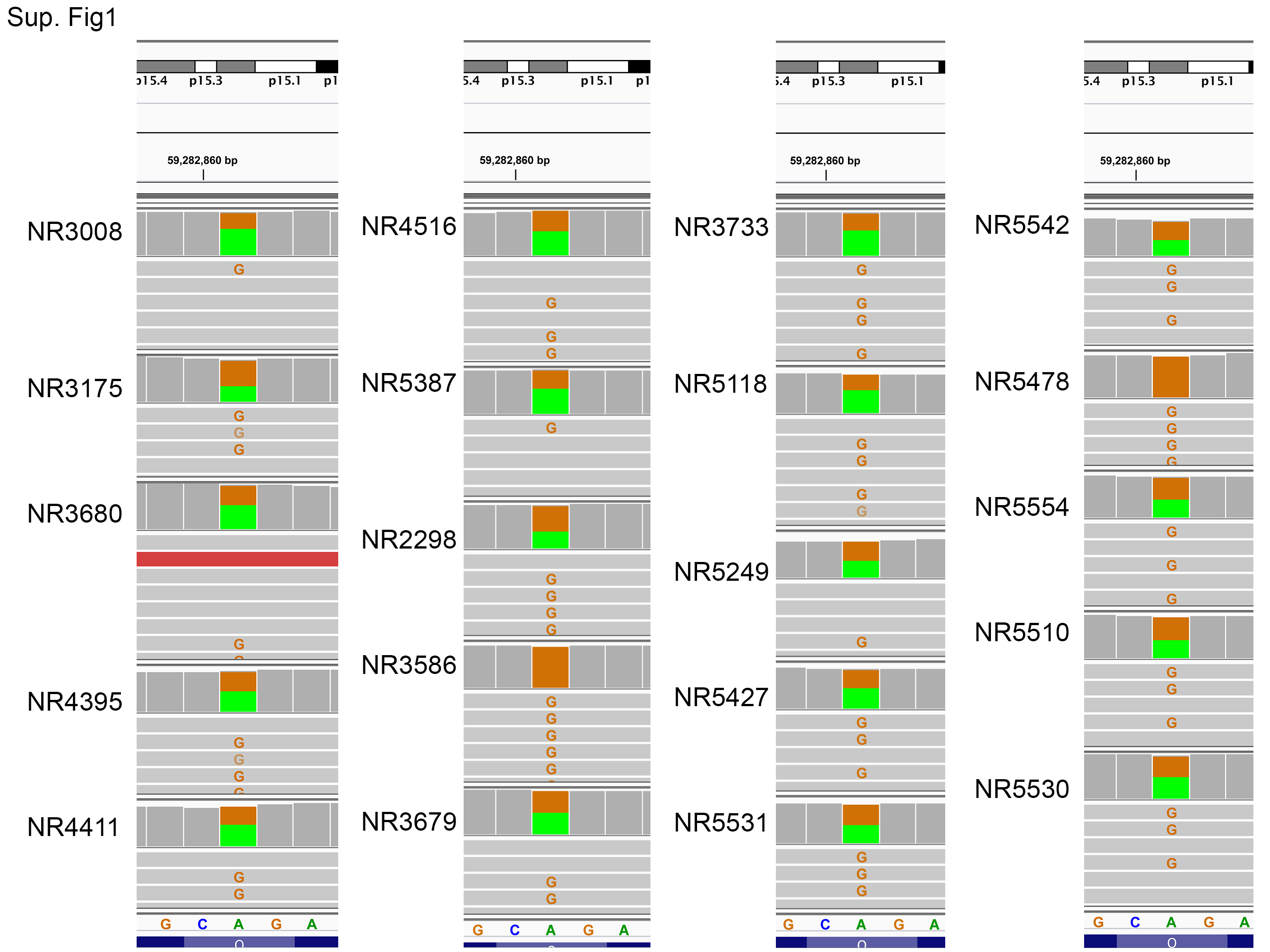

Supplement: Supplementary file 1 — Fig S1 [file MGG3-9-e1691-s002.tiff]

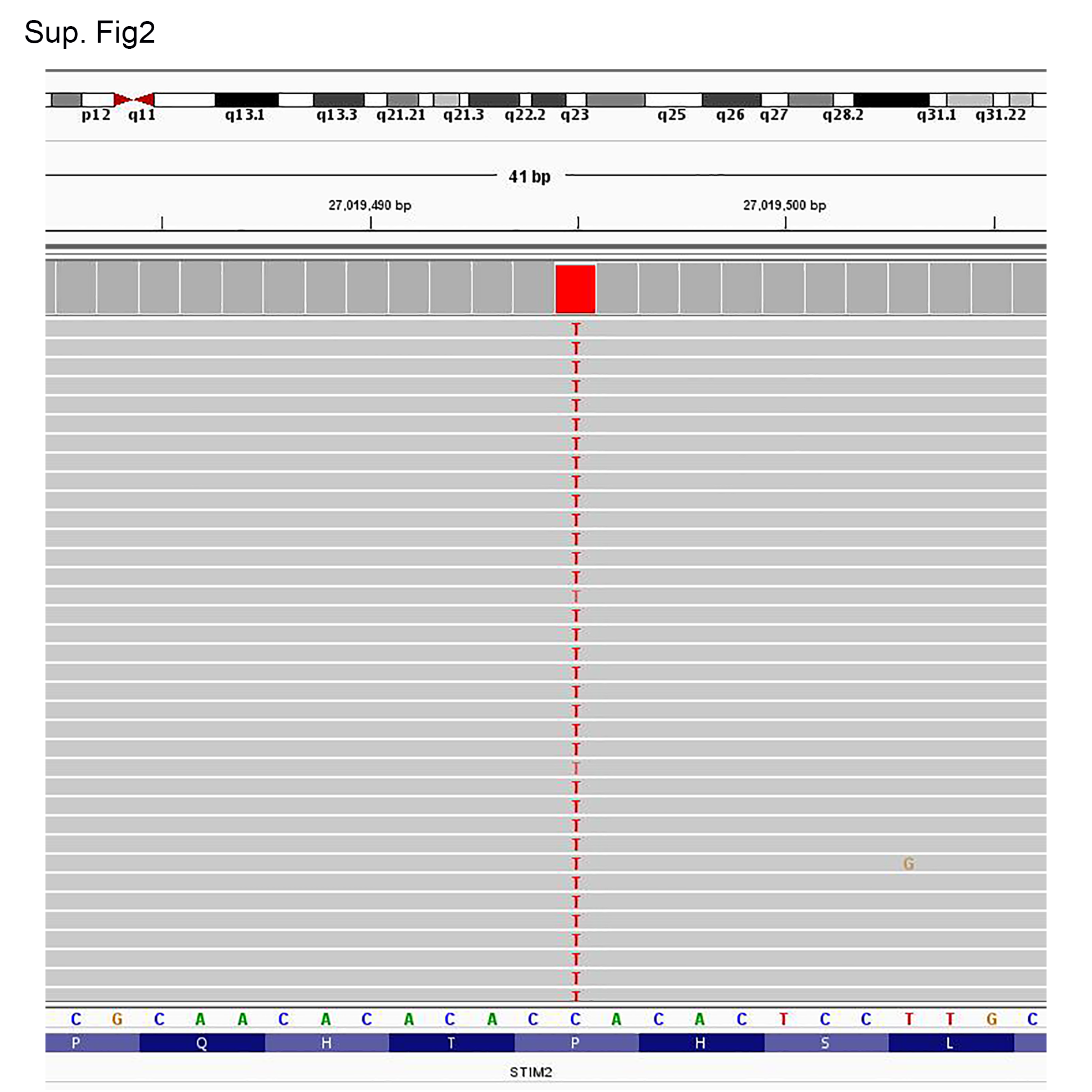

Supplement: Supplementary file 2 — Fig S2 [file MGG3-9-e1691-s005.tiff]

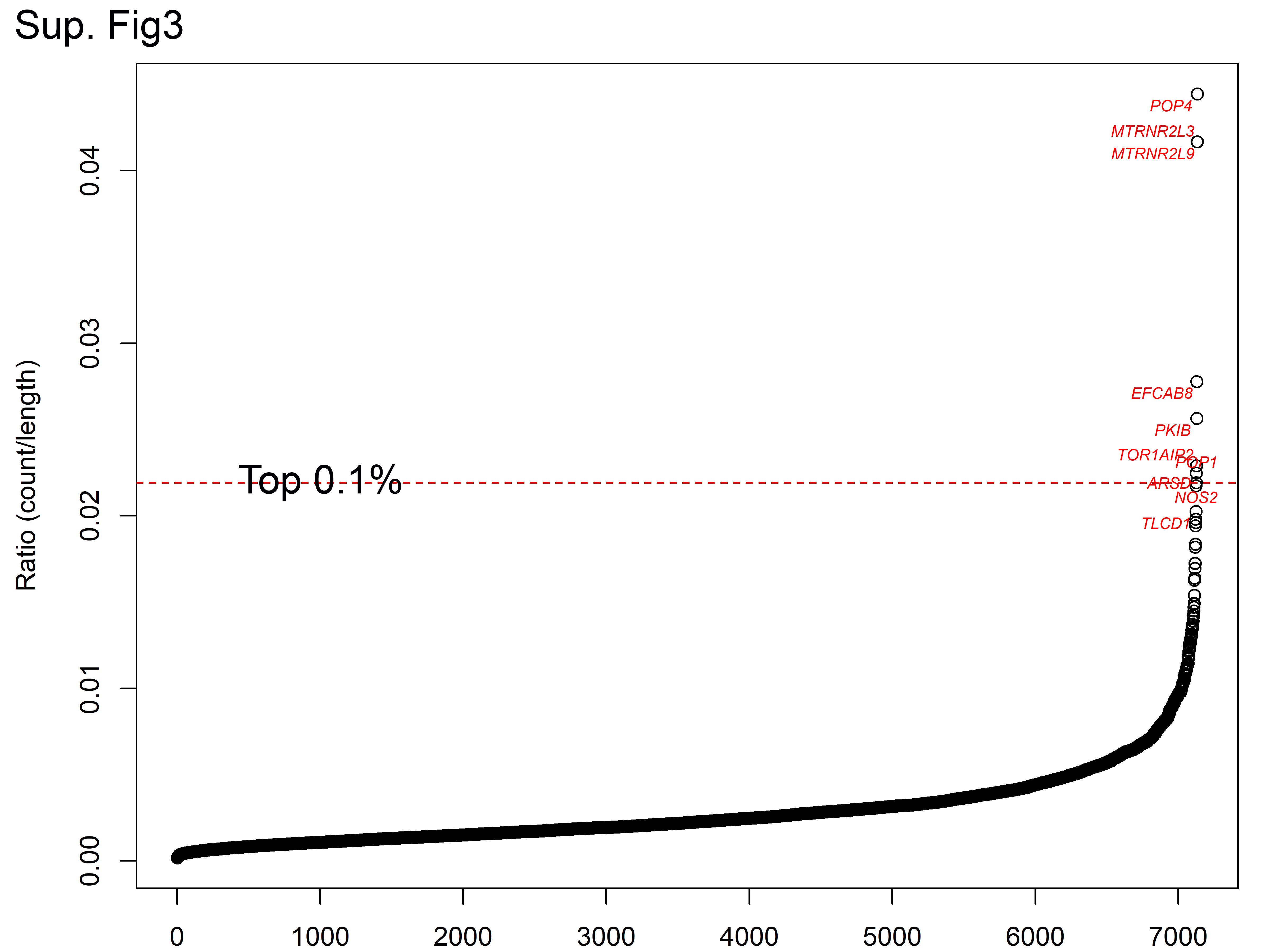

Supplement: Supplementary file 3 — Fig S3 [file MGG3-9-e1691-s008.tiff]
