# Supplementary material for: Germline and somatic mutations in the pathology of pineal cyst: A whole‐exome sequencing study of 93 individuals
Source: Mol Genet Genomic Med. 2021 May 4;9(6):e1691. doi: 10.1002/mgg3.1691 (PMC8222845; doi:10.1002/mgg3.1691)
Supplement: Supplementary file 5 — Table S2 [file MGG3-9-e1691-s007.pdf]

Sample Count	GnomAD MAF cutoff							
	5.00E-04	0.001	0.005	0.01	0.02	0.03	0.04	0.05
**1**	14535	16730	23653	26420	28276	29062	29483	29696
**2**	163	334	2056	3331	4335	4673	4862	4973
**3**	5	26	434	1102	1856	2170	2299	2396
**4**	0	0	85	420	1024	1346	1487	1576
**5**	0	0	23	132	540	812	955	1040
**6**	0	0	1	40	249	464	619	710
**7**	0	0	0	16	134	339	517	609
**8**	0	0	0	1	49	173	278	385
**9**	0	0	0	1	26	105	202	316
**10**	0	0	0	0	7	50	118	200
**11**	0	0	0	0	3	31	71	146
**12**	0	0	0	0	1	12	48	95
**13**	0	0	0	0	1	8	30	68
**14**	0	0	0	0	0	3	21	54
**15**	0	0	0	0	0	0	11	32
**16**	0	0	0	0	0	1	6	10
**17**	0	0	0	0	0	0	0	7
**18**	0	0	0	0	0	0	2	8
**19**	0	0	0	0	0	0	1	3
**20**	0	0	0	0	0	0	1	1
