# Supplementary material for: Germline and somatic mutations in the pathology of pineal cyst: A whole‐exome sequencing study of 93 individuals
Source: Mol Genet Genomic Med. 2021 May 4;9(6):e1691. doi: 10.1002/mgg3.1691 (PMC8222845; doi:10.1002/mgg3.1691)
Supplement: Supplementary file 6 — Table S3 [file MGG3-9-e1691-s003.pdf]

Sample Count	Gnomad MAF cutoff							
	5.00E-04	0.001	0.005	0.01	0.02	0.03	0.04	0.05
**0**	14389	16726	25669	30730	35559	38094	39645	40738
**1**	1	2	36	81	203	357	519	696
**2**	0	0	0	0	3	10	31	54
**3**	0	0	0	0	0	0	2	3
