# Supplementary material for: Germline and somatic mutations in the pathology of pineal cyst: A whole‐exome sequencing study of 93 individuals
Source: Mol Genet Genomic Med. 2021 May 4;9(6):e1691. doi: 10.1002/mgg3.1691 (PMC8222845; doi:10.1002/mgg3.1691)
Supplement: Supplementary file 7 — Table S4 [file MGG3-9-e1691-s001.pdf]

gene	count	length	Mutation fraction
DYNC1H1	1	4646	0.000215239
CFAP47	1	3187	0.000313775
NF1	1	2839	0.000352237
TLN1	1	2541	0.000393546
FAS	1	2511	0.000398248
RAD54L	1	2492	0.000401284
RIF1	1	2472	0.000404531
DOPEY1	1	2465	0.00040568
KNL1	1	2342	0.000426985
BRWD1	1	2320	0.000431034
ARMCX4	1	2290	0.000436681
GON4L	1	2241	0.000446229
ASCC3	1	2202	0.000454133
HUWE1	2	4374	0.000457247
MED13	1	2174	0.000459982
CACNA1D	1	2161	0.000462749
DOCK7	1	2140	0.00046729
TANC2	1	1990	0.000502513
ZNF638	1	1978	0.000505561
POLR2A	1	1970	0.000507614
HELZ	1	1942	0.000514933
MYH2	1	1941	0.000515198
TRRAP	2	3859	0.000518269
MAK	1	1907	0.000524384
AKAP11	1	1901	0.000526039
NYNRIN	1	1898	0.00052687
NUP210L	1	1888	0.000529661
BRCA1	1	1863	0.000536769
RBP1	1	1857	0.000538503
ARFGEF1	1	1849	0.000540833
KIAA1549L	1	1849	0.000540833
KIAA0368	1	1845	0.000542005
PLXNB2	1	1838	0.00054407
ZSWIM8	1	1837	0.000544366
TNRC6B	1	1833	0.000545554
MUC12	3	5478	0.000547645
WDR11	1	1821	0.000549149
FRMPD3	1	1810	0.000552486
WNK3	1	1800	0.000555556
RBBP6	1	1792	0.000558036
ARFGEF2	1	1785	0.000560224
DCDC1	1	1783	0.000560852
STRC	1	1775	0.00056338
WDFY3	2	3526	0.000567215
RGPD4	1	1758	0.000568828

PEAK1	1	1746	0.000572738
NALCN	1	1738	0.000575374
SHANK3	1	1731	0.000577701
SUPT6H	1	1726	0.000579374
ITSN1	1	1721	0.000581058
PCLO	3	5142	0.000583431
NRXN2	1	1712	0.000584112
CDC42BPB	1	1711	0.000584454
UNC13A	1	1703	0.000587199
PBRM1	1	1689	0.000592066
ADGB	1	1667	0.00059988
PHRF1	1	1649	0.000606428
ABCA5	1	1642	0.000609013
MROH1	1	1641	0.000609385
RNF17	1	1623	0.000616143
DNMT1	1	1616	0.000618812
SETBP1	1	1596	0.000626566
GLI2	1	1586	0.000630517
MAST1	1	1570	0.000636943
UGGT1	1	1555	0.000643087
ABCC9	1	1549	0.000645578
ARHGEF12	1	1544	0.000647668
ADGRG4	2	3080	0.000649351
CLASP1	1	1538	0.000650195
MAST1	1	1538	0.000650195
TOP2A	1	1531	0.000653168
KIAA2022	1	1516	0.000659631
FAM135A	1	1515	0.000660066
CDK13	1	1512	0.000661376
QARS	1	1512	0.000661376
PRDM15	1	1507	0.00066357
LMTK2	1	1503	0.000665336
ATP7A	1	1500	0.000666667
RAPGEF2	1	1499	0.000667111
ZCCHC6	1	1495	0.000668896
WDR7	1	1490	0.000671141
COL2A1	1	1487	0.000672495
CECR2	1	1484	0.000673854
ADGRL1	1	1474	0.000678426
PRX	1	1461	0.000684463
PTPRM	1	1452	0.000688705
PDS5B	1	1447	0.000691085
PCP2	1	1446	0.000691563
PIK3C2G	1	1445	0.000692042
PTPRG	1	1445	0.000692042
ARHGAP31	1	1444	0.000692521
C9orf84	1	1444	0.000692521

PTPRK	1	1439	0.000694927
WRN	1	1432	0.000698324
DAPK1	1	1430	0.000699301
YTHDC2	1	1430	0.000699301
ANKRD50	1	1429	0.00069979
YEATS2	1	1422	0.000703235
UACA	1	1416	0.000706215
POGZ	1	1410	0.00070922
CRB1	1	1406	0.000711238
NIPBL	2	2804	0.000713267
KDM6A	1	1401	0.000713776
MST1R	1	1400	0.000714286
RAB3GAP2	1	1393	0.000717875
RPAP1	1	1393	0.000717875
SBNO1	1	1393	0.000717875
DISP3	1	1392	0.000718391
POLR3A	1	1390	0.000719424
CNTNAP1	1	1384	0.000722543
CARMIL1	1	1371	0.000729395
MED23	1	1368	0.000730994
COL1A2	1	1366	0.000732064
BRD4	1	1362	0.000734214
CHD5	2	2715	0.000736648
ROCK1	1	1354	0.000738552
CADPS	1	1353	0.000739098
CLSPN	1	1339	0.000746826
AOX1	1	1338	0.000747384
PDS5A	1	1337	0.000747943
MAPK8IP3	1	1336	0.000748503
RPTOR	1	1335	0.000749064
SOS1	1	1333	0.000750188
IL16	1	1332	0.000750751
KDM3A	1	1321	0.000757002
NCAN	1	1321	0.000757002
KIF16B	1	1317	0.000759301
SRA1	1	1312	0.000762195
AFF2	1	1311	0.000762777
MAST3	1	1309	0.000763942
ERBB4	1	1308	0.000764526
HECTD1	2	2610	0.000766284
GIGYF2	1	1299	0.000769823
POLH	2	2590	0.000772201
KIAA0754	1	1291	0.000774593
CNTNAP3	1	1288	0.000776398
STIL	1	1287	0.000777001
ABCB4	1	1286	0.000777605
RAB11FIP1	1	1283	0.000779423

SETD2	2	2564	0.000780031
TTLL5	1	1281	0.00078064
HYDIN	4	5121	0.000781097
SCAP	1	1279	0.000781861
NPC1	1	1278	0.000782473
PRDM16	1	1276	0.000783699
KIAA0825	1	1275	0.000784314
HDLBP	1	1268	0.000788644
SEC24B	1	1268	0.000788644
NOMO2	1	1267	0.000789266
SGO2	1	1265	0.000790514
TRAPPC10	1	1259	0.000794281
NEK1	1	1258	0.000794913
STAG1	1	1258	0.000794913
ARID4A	1	1257	0.000795545
RBP1	1	1257	0.000795545
CSPP1	1	1256	0.000796178
MUC1	1	1255	0.000796813
BRIP1	1	1249	0.000800641
TPP2	1	1249	0.000800641
MMS22L	1	1243	0.000804505
RYR2	4	4967	0.000805315
IFT122	1	1241	0.000805802
SLC4A2	1	1241	0.000805802
MDP1	1	1239	0.000807103
EDRF1	1	1238	0.000807754
PCDH9	1	1237	0.000808407
KCNMA1	1	1236	0.000809061
SPTAN1	2	2472	0.000809061
SLK	1	1235	0.000809717
SMC1B	1	1235	0.000809717
KIF4B	1	1234	0.000810373
MAP1B	2	2468	0.000810373
ANO8	1	1232	0.000811688
WDR11	1	1224	0.000816993
ATP2B1	1	1220	0.000819672
ATP2B3	1	1220	0.000819672
EIF5B	1	1220	0.000819672
SMC3	1	1217	0.000821693
PLCB1	1	1216	0.000822368
DGKD	1	1214	0.000823723
SLC4A7	1	1214	0.000823723
SLC12A2	1	1212	0.000825083
AFF1	1	1210	0.000826446
EGFR	1	1210	0.000826446
HIPK1	1	1210	0.000826446
ATP8B2	1	1209	0.00082713

ADAMTS19	1	1207	0.0008285
PPFIA1	1	1202	0.000831947
NLRP5	1	1200	0.000833333
HIPK2	1	1198	0.000834725
RGS3	1	1198	0.000834725
ZBTB38	1	1195	0.00083682
RC3H2	1	1191	0.000839631
PUM1	1	1186	0.00084317
PLCB2	1	1185	0.000843882
PPFIA4	1	1185	0.000843882
MPHOSPH9	1	1183	0.000845309
ITGA2	1	1181	0.00084674
TDRD1	1	1180	0.000847458
ITGA1	1	1179	0.000848176
PC	1	1178	0.000848896
C20orf194	1	1177	0.000849618
PLCB4	1	1175	0.000851064
ZBED4	1	1171	0.000853971
ANKFY1	1	1169	0.000855432
ZNF862	1	1169	0.000855432
FNIP1	1	1166	0.000857633
LEPR	1	1165	0.000858369
KDM2A	1	1162	0.000860585
SIN3B	1	1162	0.000860585
NPIPB11	1	1161	0.000861326
ERG	1	1159	0.000862813
HEPH	1	1158	0.000863558
CACNA1E	2	2313	0.000864678
CD163	1	1156	0.000865052
CILP2	1	1156	0.000865052
MAML2	1	1156	0.000865052
CARD11	1	1154	0.000866551
AP3D1	1	1153	0.000867303
ITGAM	1	1152	0.000868056
NRDC	1	1151	0.00086881
WNK2	2	2297	0.000870701
PRDM10	1	1147	0.00087184
MAN2A1	1	1144	0.000874126
MYBPC1	1	1141	0.000876424
CEP135	1	1140	0.000877193
MAML3	1	1138	0.000878735
MYO1B	1	1136	0.000880282
PCDH18	1	1135	0.000881057
SPATA31C2	1	1134	0.000881834
UBN1	1	1134	0.000881834
VCL	1	1134	0.000881834
ALB	1	1133	0.000882613

ICE1	2	2266	0.000882613
SEL1L3	1	1132	0.000883392
TERT	1	1132	0.000883392
ITGA6	1	1130	0.000884956
ASAP1	1	1129	0.00088574
COBLL1	1	1128	0.000886525
NCKAP1	1	1128	0.000886525
TRIM33	1	1127	0.000887311
XPO6	1	1125	0.000888889
SLC9C2	1	1124	0.00088968
ZEB1	1	1124	0.00088968
ZNF407	2	2248	0.00088968
USP43	1	1123	0.000890472
USF3	2	2245	0.000890869
CFAP70	1	1121	0.000892061
EFL1	1	1120	0.000892857
LIG3	1	1119	0.000893655
ARID1B	2	2236	0.000894454
SLC4A10	1	1118	0.000894454
ADAMTS6	1	1117	0.000895255
ERC1	1	1116	0.000896057
MCF2L2	1	1114	0.000897666
OTUD4	1	1114	0.000897666
STARD13	1	1113	0.000898473
KCNH8	1	1107	0.000903342
GLI1	1	1106	0.000904159
SMARCC1	1	1105	0.000904977
TRPM8	1	1104	0.000905797
CACNA2D1	1	1103	0.000906618
SECISBP2L	1	1101	0.000908265
SLC12A1	1	1099	0.000909918
APOBR	1	1097	0.000911577
KDM4B	1	1096	0.000912409
PLCL1	1	1095	0.000913242
SMC6	1	1091	0.00091659
BUB1	1	1085	0.000921659
SRGAP1	1	1085	0.000921659
AMOT	1	1084	0.000922509
BICRAL	1	1079	0.000926784
MYO9B	2	2157	0.000927214
TMEM132B	1	1078	0.000927644
RAPGEF1	1	1077	0.000928505
TSHZ1	1	1077	0.000928505
NEMF	1	1076	0.000929368
RANBP2	3	3224	0.000930521
ZNF518B	1	1074	0.000931099
GUCY2C	1	1073	0.000931966

SEMA6D	1	1073	0.000931966
YLPM1	2	2146	0.000931966
MED12L	2	2145	0.000932401
PAXIP1	1	1069	0.000935454
PCDH7	1	1069	0.000935454
ZBTB21	1	1066	0.000938086
DGKI	1	1065	0.000938967
ITGA8	1	1063	0.000940734
NLRP12	1	1061	0.000942507
BRD1	1	1058	0.00094518
KIF11	1	1056	0.00094697
EPHB2	1	1055	0.000947867
CEBPZ	1	1054	0.000948767
MFHAS1	1	1052	0.00095057
RBM44	1	1052	0.00095057
ANKRD27	1	1050	0.000952381
SENP7	1	1050	0.000952381
ITGA5	1	1049	0.000953289
TLR7	1	1049	0.000953289
RASA1	1	1047	0.00095511
NUP214	2	2090	0.000956938
ARMC4	1	1044	0.000957854
KLB	1	1044	0.000957854
ARHGAP32	2	2087	0.000958313
INTS3	1	1043	0.000958773
ZSCAN20	1	1043	0.000958773
SKIV2L2	1	1042	0.000959693
RANBP9	1	1041	0.000960615
TLR8	1	1041	0.000960615
PCDH10	1	1040	0.000961538
ITGA2B	1	1039	0.000962464
NACA	2	2078	0.000962464
BMPR2	1	1038	0.000963391
HIP1	1	1037	0.00096432
IPO8	1	1037	0.00096432
PREP	1	1037	0.00096432
NLRP3	1	1036	0.000965251
ULK2	1	1036	0.000965251
ITGA9	1	1035	0.000966184
DOCK9	2	2069	0.000966651
CNKSR2	1	1034	0.000967118
KSR2	1	1034	0.000967118
NLRP11	1	1033	0.000968054
ITGA4	1	1032	0.000968992
MORC2	1	1032	0.000968992
PPP1R12A	1	1030	0.000970874
MYO10	2	2058	0.000971817

ZNF197	1	1029	0.000971817
INO80D	1	1027	0.00097371
CPED1	1	1026	0.000974659
PARP10	1	1025	0.00097561
HERC5	1	1024	0.000976563
NLRC4	1	1024	0.000976563
TRHDE	1	1024	0.000976563
ATP1A1	1	1023	0.000977517
CNTN1	1	1018	0.000982318
ADGRG2	1	1017	0.000983284
KCNH4	1	1017	0.000983284
NCAPG	1	1015	0.000985222
ZBTB4	1	1013	0.000987167
OFD1	1	1012	0.000988142
PHF20	1	1012	0.000988142
HDAC9	1	1011	0.00098912
GART	1	1010	0.000990099
DENND1A	1	1009	0.00099108
DENND2A	1	1009	0.00099108
LIG3	1	1009	0.00099108
PRPF4B	1	1007	0.000993049
MYO1D	1	1006	0.000994036
TMC5	1	1006	0.000994036
ZNF316	1	1004	0.000996016
GOLGA2	1	1002	0.000998004
ATP2A1	1	1001	0.000999001
SEC23IP	1	1000	0.001
HYOU1	1	999	0.001001001
INTS8	1	995	0.001005025
KANK4	1	995	0.001005025
TMEM67	1	995	0.001005025
INPP5B	1	993	0.001007049
SCUBE3	1	993	0.001007049
CASP8AP2	2	1982	0.001009082
SMG8	1	991	0.001009082
LVRN	1	990	0.001010101
KCNH1	1	989	0.001011122
JMY	1	988	0.001012146
KCNH5	1	988	0.001012146
EPHA4	1	986	0.001014199
TXNDC11	1	985	0.001015228
NR3C2	1	984	0.00101626
ICE2	1	982	0.00101833
EML4	1	981	0.001019368
RAI14	1	980	0.001020408
EML6	2	1958	0.00102145
AP2A1	1	977	0.001023541

CHD5	2	1954	0.001023541
RBM15	1	977	0.001023541
SYNPO2L	1	977	0.001023541
EPHA1	1	976	0.00102459
SYCP1	1	976	0.00102459
TRANK1	3	2925	0.001025641
WDR59	1	974	0.001026694
SLC8A1	1	973	0.001027749
CSF1R	1	972	0.001028807
CSE1L	1	971	0.001029866
RECK	1	971	0.001029866
ZC3H7A	1	971	0.001029866
MYO19	1	970	0.001030928
MUC16	15	14507	0.001033984
LGR6	1	967	0.001034126
INTS4	1	963	0.001038422
KIF5B	1	963	0.001038422
RASIP1	1	963	0.001038422
SART3	1	963	0.001038422
CFAP65	2	1925	0.001038961
INTS7	1	962	0.001039501
PCDHGA4	1	962	0.001039501
XPOT	1	962	0.001039501
DICER1	2	1922	0.001040583
FGD1	1	961	0.001040583
KIF23	1	960	0.001041667
ANKRD36	2	1915	0.001044386
COL21A1	1	957	0.001044932
CHAF1A	1	956	0.001046025
CLSTN3	1	956	0.001046025
FBXO10	1	956	0.001046025
MATN2	1	956	0.001046025
ANO4	1	955	0.00104712
CEP112	1	955	0.00104712
HAUS6	1	955	0.00104712
MAP3K10	1	954	0.001048218
PSMD1	1	953	0.001049318
UNC5D	1	953	0.001049318
CATSPERE	1	951	0.001051525
CCDC15	1	951	0.001051525
PCDH20	1	951	0.001051525
CCDC80	1	950	0.001052632
KSR2	1	950	0.001052632
PPP4R1	1	950	0.001052632
ZCCHC14	1	949	0.001053741
NOV	2	1896	0.001054852
PLXNA1	2	1896	0.001054852

SOGA3	1	947	0.001055966
ARHGAP4	1	946	0.001057082
ROR2	1	943	0.001060445
WDR3	1	943	0.001060445
DCAF5	1	942	0.001061571
INTU	1	942	0.001061571
PAK1	1	942	0.001061571
CFAP69	1	941	0.001062699
ERAP1	1	941	0.001062699
KDM7A	1	941	0.001062699
PRPF6	1	941	0.001062699
GRIN1	1	938	0.001066098
ROR1	1	937	0.001067236
RALGAPA2	2	1873	0.001067806
TAF1	2	1872	0.001068376
MTHFD1	1	935	0.001069519
NPAS3	1	933	0.001071811
DOCK1	2	1865	0.001072386
TRPM7	2	1865	0.001072386
RBM12	1	932	0.001072961
DENND4A	2	1863	0.001073537
GPATCH1	1	931	0.001074114
PCDHGA5	1	931	0.001074114
PCDHGB2	1	931	0.001074114
RB1	1	928	0.001077586
MCF2	1	925	0.001081081
ICAM5	1	924	0.001082251
TBC1D30	1	924	0.001082251
CDH20	1	923	0.001083424
PCDHGB5	1	923	0.001083424
ATAD5	2	1844	0.001084599
PGAP1	1	922	0.001084599
USP29	1	922	0.001084599
GRIK1	1	918	0.001089325
NBEAL2	3	2754	0.001089325
HK1	1	917	0.001090513
PAXBP1	1	917	0.001090513
SPEN	4	3664	0.001091703
SAFB	1	915	0.001092896
SPIDR	1	915	0.001092896
CLCA1	1	914	0.001094092
ECT2	1	914	0.001094092
SLC12A9	1	914	0.001094092
TRAK2	1	914	0.001094092
ANO5	1	913	0.00109529
WDR44	1	913	0.00109529
ARHGEF1	1	912	0.001096491

E2F7	1	911	0.001097695
KCNB2	1	911	0.001097695
LTBP3	2	1821	0.001098298
ADAM12	1	909	0.00110011
GOLGA6L2	1	909	0.00110011
ZBTB41	1	909	0.00110011
EIF4G2	1	907	0.001102536
TUBGCP3	1	907	0.001102536
CBL	1	906	0.001103753
C3orf20	1	904	0.001106195
THAP9	1	903	0.00110742
FAM193B	1	902	0.001108647
BRWD3	2	1802	0.001109878
SLFN11	1	901	0.001109878
CENPE	3	2701	0.0011107
DDHD1	1	900	0.001111111
EPB41L4B	1	900	0.001111111
TNPO1	1	898	0.001113586
TNPO2	1	897	0.001114827
LPIN2	1	896	0.001116071
ZNF574	1	896	0.001116071
RASSF4	1	895	0.001117318
ZNF281	1	895	0.001117318
BCL11B	1	894	0.001118568
MAP4K3	1	894	0.001118568
SLFN5	1	891	0.001122334
FAM171A1	1	890	0.001123596
RBM15B	1	890	0.001123596
PKN3	1	889	0.001124859
PTCHD1	1	888	0.001126126
SEMA6B	1	888	0.001126126
SH3RF1	1	888	0.001126126
PIK3C3	1	887	0.001127396
ADGRE1	1	886	0.001128668
KIDINS220	2	1771	0.001129305
KMT5B	1	885	0.001129944
CHSY3	1	882	0.001133787
PML	1	882	0.001133787
AMPD2	1	879	0.001137656
GRM3	1	879	0.001137656
RGPD3	2	1758	0.001137656
TMEM245	1	879	0.001137656
MAP7D3	1	876	0.001141553
ARHGAP42	1	874	0.001144165
COPG1	1	874	0.001144165
GAREM2	1	874	0.001144165
KIAA1551	2	1747	0.00114482

COL5A3	2	1745	0.001146132
NDST4	1	872	0.001146789
SIMC1	1	872	0.001146789
PRPF40B	1	871	0.001148106
ZNF473	1	871	0.001148106
DNM2	1	870	0.001149425
POLK	1	870	0.001149425
DNM3	1	869	0.001150748
CDC42BPA	2	1732	0.001154734
PKD1L3	2	1732	0.001154734
RNF216	1	866	0.001154734
CEP97	1	865	0.001156069
PROM1	1	865	0.001156069
NAA16	1	864	0.001157407
ADAM15	1	863	0.001158749
ENPP2	1	863	0.001158749
MCM4	1	863	0.001158749
PARP4	2	1724	0.001160093
TAF4B	1	862	0.001160093
PIWIL1	1	861	0.00116144
CHD8	3	2581	0.00116234
SH2D3C	1	860	0.001162791
MAP3K12	1	859	0.001164144
PDE6C	1	858	0.001165501
AGAP1	1	857	0.001166861
BCORL1	2	1711	0.001168907
AKAP4	1	854	0.00117096
DISC1	1	854	0.00117096
PARP8	1	854	0.00117096
RICTOR	2	1708	0.00117096
ZNF341	1	854	0.00117096
AKAP3	1	853	0.001172333
TGS1	1	853	0.001172333
XRN1	2	1706	0.001172333
ARAP2	2	1704	0.001173709
LONP2	1	852	0.001173709
TAS1R3	1	852	0.001173709
MTOR	3	2549	0.001176932
AHR	1	848	0.001179245
CRNKL1	1	848	0.001179245
MAP4K5	1	846	0.001182033
SLITRK2	1	845	0.001183432
PRICKLE2	1	844	0.001184834
HMCN2	6	5059	0.001186005
COL4A5	2	1685	0.001186944
JADE1	1	842	0.001187648
UNC5A	1	842	0.001187648

SHPRH	2	1683	0.001188354
NEK4	1	841	0.001189061
CFAP221	1	840	0.001190476
ESCO1	1	840	0.001190476
FAM91A1	1	838	0.001193317
ADAMTS4	1	837	0.001194743
APBA1	1	837	0.001194743
DNAAF2	1	837	0.001194743
NCAM2	1	837	0.001194743
SLITRK4	1	837	0.001194743
STRIP1	1	837	0.001194743
COL4A1	2	1669	0.001198322
CCSER2	1	834	0.001199041
RASA3	1	834	0.001199041
ALMS1	5	4168	0.001199616
IFT88	1	833	0.00120048
KIFC3	1	833	0.00120048
MAP4K1	1	833	0.00120048
KAT2B	1	832	0.001201923
TMEM63B	1	832	0.001201923
CDH16	1	829	0.001206273
IQGAP1	2	1657	0.001207001
MYSM1	1	828	0.001207729
SOX6	1	828	0.001207729
NHS	2	1651	0.001211387
FSCB	1	825	0.001212121
HNRNPU	1	825	0.001212121
IL4R	1	825	0.001212121
PRDM1	1	825	0.001212121
ZNF229	1	825	0.001212121
MALT1	1	824	0.001213592
MAPK8IP2	1	824	0.001213592
AREL1	1	823	0.001215067
ANAPC2	1	822	0.001216545
FES	1	822	0.001216545
IQCA1	1	822	0.001216545
ZNF41	1	821	0.001218027
DDX23	1	820	0.001219512
ELFN2	1	820	0.001219512
NUP93	1	819	0.001221001
CLCN3	1	818	0.001222494
DLG3	1	817	0.00122399
NELL2	1	816	0.00122549
PI4KB	1	816	0.00122549
IQGAP3	2	1631	0.001226242
SLC9A1	1	815	0.001226994
GPR156	1	814	0.001228501

IGDCC3	1	814	0.001228501
CAPN7	1	813	0.001230012
SYCP2L	1	812	0.001231527
RNF10	1	811	0.001233046
ZNF839	1	811	0.001233046
EVI5	1	810	0.001234568
JAKMIP2	1	810	0.001234568
LRRC8A	1	810	0.001234568
PDE4D	1	809	0.001236094
PGBD1	1	809	0.001236094
WHAMM	1	809	0.001236094
10-Mar	1	808	0.001237624
ZNF841	1	808	0.001237624
OSBP	1	807	0.001239157
FGFR3	1	806	0.001240695
ACE2	1	805	0.001242236
ADCY10	2	1610	0.001242236
CEP85L	1	805	0.001242236
CLCN7	1	805	0.001242236
TACC1	1	805	0.001242236
CIC	2	1608	0.001243781
CPT1C	1	803	0.00124533
LRRC8B	1	803	0.00124533
CHSY1	1	802	0.001246883
NPAS4	1	802	0.001246883
RPS6KA5	1	802	0.001246883
UHRF2	1	802	0.001246883
USP10	2	1604	0.001246883
CDH20	1	801	0.001248439
PRDM4	1	801	0.001248439
RAPGEF6	2	1601	0.001249219
PCDHB10	1	800	0.00125
TAF5	1	800	0.00125
ZNF227	1	799	0.001251564
CATSPERD	1	798	0.001253133
HASPIN	1	798	0.001253133
PCDHB13	1	798	0.001253133
SLC9A4	1	798	0.001253133
AFG3L2	1	797	0.001254705
IMPG1	1	797	0.001254705
VPS35	1	796	0.001256281
MARK1	1	795	0.001257862
NAALADL2	1	795	0.001257862
PCDHB4	1	795	0.001257862
PLAA	1	795	0.001257862
EVI5L	1	794	0.001259446
SEL1L	1	794	0.001259446

ZNF148	1	794	0.001259446
ADGRB2	2	1585	0.00126183
CEP170	2	1584	0.001262626
CNOT1	3	2376	0.001262626
KIFAP3	1	792	0.001262626
RINT1	1	792	0.001262626
IQUB	1	791	0.001264223
ABCC8	2	1581	0.001265022
PLEKHG6	1	790	0.001265823
WDR24	1	790	0.001265823
ZNF560	1	790	0.001265823
ARNT	1	789	0.001267427
CDH9	1	789	0.001267427
MED15	1	788	0.001269036
KIAA1586	1	787	0.001270648
CASS4	1	786	0.001272265
CDH7	1	785	0.001273885
USP1	1	785	0.001273885
SP4	1	784	0.00127551
BRINP2	1	783	0.001277139
CEP89	1	783	0.001277139
DDX21	1	783	0.001277139
PPP1R12C	1	782	0.001278772
TTC37	2	1564	0.001278772
VAC14	1	782	0.001278772
ZNF786	1	782	0.001278772
AKAP9	5	3907	0.001279754
SP3	1	781	0.00128041
ERCC6L2	2	1561	0.00128123
ASAH2	1	780	0.001282051
BICRA	2	1560	0.001282051
CUL5	1	780	0.001282051
PTPN12	1	780	0.001282051
TSC22D2	1	780	0.001282051
PDE10A	1	779	0.001283697
ZW10	1	779	0.001283697
CENPF	4	3114	0.001284522
PRPF8	3	2335	0.001284797
ABLIM1	1	778	0.001285347
BRSK1	1	778	0.001285347
C11orf63	1	778	0.001285347
INO80	2	1556	0.001285347
KIRREL3	1	778	0.001285347
MAGED1	1	778	0.001285347
ZFYVE1	1	777	0.001287001
DUOX1	2	1551	0.001289491
CHPF	1	775	0.001290323

GCC1	1	775	0.001290323
QARS	1	775	0.001290323
TBC1D12	1	775	0.001290323
MAMLD1	1	774	0.00129199
CMIP	1	773	0.001293661
LONRF1	1	773	0.001293661
PANK4	1	773	0.001293661
CHSY3	1	772	0.001295337
KDM5B	2	1544	0.001295337
PAPD7	1	772	0.001295337
TLK2	1	772	0.001295337
LRFN1	1	771	0.001297017
UTP14A	1	771	0.001297017
CMTR2	1	770	0.001298701
COG7	1	770	0.001298701
ECE1	1	770	0.001298701
ZFYVE16	2	1539	0.001299545
ADAM11	1	769	0.00130039
ITGB8	1	769	0.00130039
LRIF1	1	769	0.00130039
RIF1	1	769	0.00130039
ZNF585A	1	769	0.00130039
ZNF585B	1	769	0.00130039
LRRC7	2	1537	0.001301236
AFAP1L1	1	768	0.001302083
CUL3	1	768	0.001302083
SLC45A4	1	768	0.001302083
MAATS1	1	767	0.001303781
NSUN2	1	767	0.001303781
SLIT1	2	1534	0.001303781
ABCB9	1	766	0.001305483
BRAF	1	766	0.001305483
BRINP3	1	766	0.001305483
SIM1	1	766	0.001305483
UTP14C	1	766	0.001305483
NFAT5	2	1531	0.001306336
ERICH3	2	1530	0.00130719
FAM160B1	1	765	0.00130719
LRCH2	1	765	0.00130719
SLC30A5	1	765	0.00130719
ZDHHC8	1	765	0.00130719
SLIT2	2	1529	0.001308044
FASTKD5	1	764	0.001308901
OSBPL10	1	764	0.001308901
ANKFN1	1	763	0.001310616
AOC3	1	763	0.001310616
APLP2	1	763	0.001310616

PHTF1	1	762	0.001312336
EHBP1L1	2	1523	0.001313198
ADGRB3	2	1522	0.00131406
TATDN2	1	761	0.00131406
FIGN	1	759	0.001317523
GIT2	1	759	0.001317523
LONRF3	1	759	0.001317523
ZYG11A	1	759	0.001317523
APBB2	1	758	0.001319261
DPY19L2	1	758	0.001319261
RRP1B	1	758	0.001319261
RUSC2	2	1516	0.001319261
KCNC3	1	757	0.001321004
KIRREL	1	757	0.001321004
LINS1	1	757	0.001321004
TRMT44	1	757	0.001321004
AOC2	1	756	0.001322751
CENPI	1	756	0.001322751
SH2B1	1	756	0.001322751
SPIRE1	1	756	0.001322751
VWA2	1	755	0.001324503
LONRF2	1	754	0.00132626
NBN	1	754	0.00132626
PPIG	1	754	0.00132626
CEL	1	753	0.001328021
MARK3	1	753	0.001328021
CDRT1	1	752	0.001329787
TRPC7	2	1503	0.001330672
ZNF184	1	751	0.001331558
ZNF337	1	751	0.001331558
BCL9L	2	1499	0.001334223
FSD2	1	749	0.001335113
SEMA3B	1	749	0.001335113
STAT4	1	748	0.001336898
SYNJ2	2	1496	0.001336898
RAD54L	1	747	0.001338688
DIDO1	3	2240	0.001339286
BOP1	1	746	0.001340483
CLCN5	1	746	0.001340483
EXT1	1	746	0.001340483
MEP1A	1	746	0.001340483
ARHGAP23	2	1491	0.001341382
EXOC3	1	745	0.001342282
CNOT10	1	744	0.001344086
TRIM3	1	744	0.001344086
ZNF483	1	744	0.001344086
TBX3	1	743	0.001345895

ZNF267	1	743	0.001345895
AQR	2	1485	0.001346801
CD44	1	742	0.001347709
BAZ1B	2	1483	0.001348618
ADARB1	1	741	0.001349528
MGAT5	1	741	0.001349528
NCAPH	1	741	0.001349528
PZP	2	1482	0.001349528
DCLK1	1	740	0.001351351
LRRN4	1	740	0.001351351
POLI	1	740	0.001351351
MUC2	7	5179	0.001351612
GAPVD1	2	1478	0.00135318
NRXN1	2	1477	0.001354096
CCDC88B	2	1476	0.001355014
GRAMD1B	1	738	0.001355014
DDX50	1	737	0.001356852
DPP3	1	737	0.001356852
PGGHG	1	737	0.001356852
MED13L	3	2210	0.001357466
NBEA	4	2946	0.001357773
KNTC1	3	2209	0.001358081
SLC6A16	1	736	0.001358696
MID2	1	735	0.001360544
RPS6KA1	1	735	0.001360544
SYDE1	1	735	0.001360544
MCM5	1	734	0.001362398
RAD54L2	2	1467	0.001363327
FNDC7	1	733	0.001364256
FOXK1	1	733	0.001364256
HIC1	1	733	0.001364256
APEH	1	732	0.00136612
F13A1	1	732	0.00136612
MAP7D2	1	732	0.00136612
RNF19B	1	732	0.00136612
PLA2R1	2	1463	0.001367054
GPR149	1	731	0.001367989
NKTR	2	1462	0.001367989
PBXIP1	1	731	0.001367989
POLA1	2	1462	0.001367989
RFWD2	1	731	0.001367989
TAGAP	1	731	0.001367989
HNRNPM	1	730	0.001369863
CPEB4	1	729	0.001371742
NCDN	1	729	0.001371742
RANBP9	1	729	0.001371742
SLC15A2	1	729	0.001371742

DOCK10	3	2186	0.00137237
MAGI2	2	1455	0.00137457
CTCF	1	727	0.001375516
ESRP2	1	727	0.001375516
MTIF2	1	727	0.001375516
TERB1	1	727	0.001375516
ADAM20	1	726	0.00137741
HLCS	1	726	0.00137741
ARFGEF3	3	2177	0.001378043
DNAAF1	1	725	0.00137931
EEF2K	1	725	0.00137931
MCCC1	1	725	0.00137931
RNPEPL1	1	725	0.00137931
SLC9A7	1	725	0.00137931
BBS10	1	723	0.001383126
DPY19L4	1	723	0.001383126
GGA3	1	723	0.001383126
LRSAM1	1	723	0.001383126
MUC4	3	2169	0.001383126
PTPRU	2	1446	0.001383126
TRIM42	1	723	0.001383126
ATXN7L2	1	722	0.001385042
GNE	1	722	0.001385042
CPSF1	2	1443	0.001386001
BBS2	1	721	0.001386963
LRRFIP2	1	721	0.001386963
WDCP	1	721	0.001386963
PAMR1	1	720	0.001388889
HOOK2	1	719	0.001390821
MCM7	1	719	0.001390821
NOL11	1	719	0.001390821
SETDB2	1	719	0.001390821
TECTA	3	2155	0.001392111
EXT2	1	718	0.001392758
HOOK3	1	718	0.001392758
NFKBIZ	1	718	0.001392758
MTO1	1	717	0.0013947
FLYWCH1	1	716	0.001396648
IKBKE	1	716	0.001396648
KIAA1958	1	716	0.001396648
RSPH4A	1	716	0.001396648
TRO	2	1431	0.001397624
BBS7	1	715	0.001398601
EPS8L2	1	715	0.001398601
KIAA1161	1	714	0.00140056
LRRC4B	1	713	0.001402525
LRRN2	1	713	0.001402525

MIPEP	1	713	0.001402525
SDCCAG8	1	713	0.001402525
ZFYVE9	2	1425	0.001403509
PDE4C	1	712	0.001404494
USP44	1	712	0.001404494
ALOXE3	1	711	0.00140647
DDHD2	1	711	0.00140647
KHSRP	1	711	0.00140647
LTF	1	710	0.001408451
PREP	1	710	0.001408451
FBXO40	1	709	0.001410437
PDE1C	1	709	0.001410437
TMCC2	1	709	0.001410437
BLM	2	1417	0.001411433
ADGRF2	1	708	0.001412429
CAST	1	708	0.001412429
MRE11	1	708	0.001412429
SLC15A1	1	708	0.001412429
FBXW7	1	707	0.001414427
ZNF224	1	707	0.001414427
C2CD2L	1	706	0.001416431
G2E3	1	706	0.001416431
KIF2A	1	706	0.001416431
SLC6A9	1	706	0.001416431
RRBP1	2	1410	0.00141844
SYN1	1	705	0.00141844
ZNF611	1	705	0.00141844
MTMR8	1	704	0.001420455
CAPN8	1	703	0.001422475
DNAJC14	1	702	0.001424501
PHACTR4	1	702	0.001424501
SLCO1B3	1	702	0.001424501
VRTN	1	702	0.001424501
CEP83	1	701	0.001426534
FBXL17	1	701	0.001426534
PI4KA	3	2102	0.001427212
HHIP	1	700	0.001428571
C10orf90	1	699	0.001430615
DNAI1	1	699	0.001430615
GFPT1	1	699	0.001430615
HRC	1	699	0.001430615
MAN1B1	1	699	0.001430615
TBC1D23	1	699	0.001430615
VPS53	1	699	0.001430615
ACSL1	1	698	0.001432665
NOA1	1	698	0.001432665
TSGA10	1	698	0.001432665

TCTN2	1	697	0.00143472
NIN	3	2090	0.001435407
ANKRD30B	2	1392	0.001436782
IL1RAPL1	1	696	0.001436782
SESTD1	1	696	0.001436782
SLITRK1	1	696	0.001436782
ZNF607	1	696	0.001436782
ZSWIM3	1	696	0.001436782
ATAD2	2	1390	0.001438849
LMBRD2	1	695	0.001438849
SATL1	1	695	0.001438849
FZD8	1	694	0.001440922
KIF15	2	1388	0.001440922
NFE2L3	1	694	0.001440922
PADI6	1	694	0.001440922
TBC1D14	1	693	0.001443001
TGM3	1	693	0.001443001
ZNF441	1	693	0.001443001
ZNF180	1	692	0.001445087
PALLD	2	1383	0.001446132
EPB42	1	691	0.001447178
FBXL5	1	691	0.001447178
GCNA	1	691	0.001447178
KAT6B	3	2073	0.001447178
NOBOX	1	691	0.001447178
POU6F2	1	691	0.001447178
RPF1	1	691	0.001447178
SLCO1B1	1	691	0.001447178
TBC1D15	1	691	0.001447178
HEG1	2	1381	0.001448226
HEATR5B	3	2071	0.001448576
CNGA1	1	690	0.001449275
GUCY1A3	1	690	0.001449275
SLC34A2	1	690	0.001449275
ITPR1	4	2758	0.001450326
ACSS1	1	689	0.001451379
ZNF185	1	689	0.001451379
SCLT1	1	688	0.001453488
SEL1L2	1	688	0.001453488
SRPK2	1	688	0.001453488
ZBTB48	1	688	0.001453488
NCOA6	3	2063	0.001454193
USP47	2	1375	0.001454545
AATK	2	1374	0.001455604
PATZ1	1	687	0.001455604
ACSS3	1	686	0.001457726
EOMES	1	686	0.001457726

HSPA12B	1	686	0.001457726
IL1RAPL2	1	686	0.001457726
LRRC49	1	686	0.001457726
DLL4	1	685	0.001459854
RNF6	1	685	0.001459854
SLC3A1	1	685	0.001459854
ARHGEF10	2	1369	0.00146092
FOXRED2	1	684	0.001461988
KBTBD7	1	684	0.001461988
KIF24	2	1368	0.001461988
TWNK	1	684	0.001461988
BRCA2	5	3418	0.001462844
DIXDC1	1	683	0.001464129
ITPKC	1	683	0.001464129
LRRN4	1	683	0.001464129
SV2B	1	683	0.001464129
ZNF263	1	683	0.001464129
ZNF510	1	683	0.001464129
TTC39B	1	682	0.001466276
FLT4	2	1363	0.001467351
MPHOSPH10	1	681	0.001468429
ZNF292	4	2723	0.001468968
ARHGEF18	2	1361	0.001469508
ANOS1	1	680	0.001470588
FOXP4	1	680	0.001470588
CR1	3	2039	0.001471309
NPC1L1	2	1359	0.00147167
FAM178B	1	679	0.001472754
MS4A14	1	679	0.001472754
ZNF283	1	679	0.001472754
KTN1	2	1357	0.001473839
TMEM94	2	1356	0.001474926
OGFR	1	677	0.001477105
PANX2	1	677	0.001477105
WDR43	1	677	0.001477105
WDTC1	1	677	0.001477105
DOCK3	3	2030	0.001477833
ALOX15B	1	676	0.00147929
KCNQ1	1	676	0.00147929
LIMD1	1	676	0.00147929
PRKCD	1	676	0.00147929
NEXN	1	675	0.001481481
NDC1	1	674	0.00148368
RGS9	1	674	0.00148368
ANXA6	1	673	0.001485884
VASN	1	673	0.001485884
SCN5A	3	2016	0.001488095

SHCBP1	1	672	0.001488095
ZBTB33	1	672	0.001488095
MACF1	11	7388	0.001488901
ERBB3	2	1342	0.001490313
SYTL4	1	671	0.001490313
WDR19	2	1342	0.001490313
ZNF443	1	671	0.001490313
ANGEL1	1	670	0.001492537
FEM1A	1	669	0.001494768
FLT1	2	1338	0.001494768
GLS	1	669	0.001494768
GTPBP1	1	669	0.001494768
HIF3A	1	669	0.001494768
LZTS2	1	669	0.001494768
RBM14	1	669	0.001494768
PTPRJ	2	1337	0.001495886
IGSF1	2	1336	0.001497006
MTA2	1	668	0.001497006
GCN1	4	2671	0.001497566
ITPR3	4	2671	0.001497566
MID1	1	667	0.00149925
OS9	1	667	0.00149925
TCF4	1	667	0.00149925
TUBGCP4	1	667	0.00149925
LAMA3	5	3333	0.00150015
HECTD4	6	3996	0.001501502
LEO1	1	666	0.001501502
NRXN2	1	666	0.001501502
BFSP1	1	665	0.001503759
CCDC138	1	665	0.001503759
MYH14	3	1995	0.001503759
RPAP3	1	665	0.001503759
TTC30B	1	665	0.001503759
TRIP12	3	1992	0.001506024
TNKS	2	1327	0.001507159
ZNF644	2	1327	0.001507159
ALOX12	1	663	0.001508296
ARHGAP18	1	663	0.001508296
PADI1	1	663	0.001508296
RNF145	1	663	0.001508296
ZIC5	1	663	0.001508296
GGT7	1	662	0.001510574
HDC	1	662	0.001510574
IL12RB1	1	662	0.001510574
KMT2A	6	3969	0.001511716
EMSY	2	1322	0.001512859
F13B	1	661	0.001512859

FRMPD4	2	1322	0.001512859
PMEL	1	661	0.001512859
ZNF81	1	661	0.001512859
SPAG9	2	1321	0.001514005
ACOX1	1	660	0.001515152
FLRT2	1	660	0.001515152
MMP2	1	660	0.001515152
ZNF540	1	660	0.001515152
HKR1	1	659	0.001517451
MYH10	3	1976	0.001518219
ENG	1	658	0.001519757
INTS9	1	658	0.001519757
SASS6	1	657	0.00152207
TIPARP	1	657	0.00152207
ITIH6	2	1313	0.001523229
EML5	3	1969	0.001523616
NUP85	1	656	0.00152439
SETD1B	3	1966	0.001525941
SMPD3	1	655	0.001526718
CLUH	2	1309	0.001527884
NGLY1	1	654	0.001529052
TCF20	3	1960	0.001530612
MAN1A1	1	653	0.001531394
PTPRC	2	1306	0.001531394
SLC44A3	1	653	0.001531394
TMCC1	1	653	0.001531394
ADGRE3	1	652	0.001533742
C12orf40	1	652	0.001533742
GP1BA	1	652	0.001533742
UNC80	5	3258	0.001534684
LTBP3	2	1303	0.001534919
ASTN1	2	1302	0.001536098
GUSB	1	651	0.001536098
RRN3	1	651	0.001536098
ZNF816	1	651	0.001536098
DSPP	2	1301	0.001537279
FLNB	4	2602	0.001537279
ZNF532	2	1301	0.001537279
ATP8B3	2	1300	0.001538462
FAF1	1	650	0.001538462
AMBRA1	2	1298	0.001540832
EHMT1	2	1298	0.001540832
SCNN1G	1	649	0.001540832
FOXN1	1	648	0.00154321
RAF1	1	648	0.00154321
UBR4	8	5183	0.001543508
C17orf53	1	647	0.001545595

CEP72	1	647	0.001545595
COQ8A	1	647	0.001545595
KCND1	1	647	0.001545595
LIMK1	1	647	0.001545595
LRWD1	1	647	0.001545595
SLC39A4	1	647	0.001545595
AKAP8L	1	646	0.001547988
KIF12	1	646	0.001547988
SLC27A1	1	646	0.001547988
UBE2O	2	1292	0.001547988
SETDB1	2	1291	0.001549187
ADAMTS9	3	1935	0.001550388
ARHGAP25	1	645	0.001550388
DYNC1I1	1	645	0.001550388
SYT16	1	645	0.001550388
ZNF681	1	645	0.001550388
SENP1	1	644	0.001552795
ARHGAP33	2	1287	0.001554002
DENND5A	2	1287	0.001554002
AMFR	1	643	0.00155521
ASNSD1	1	643	0.00155521
SLC27A4	1	643	0.00155521
TMEM62	1	643	0.00155521
ZFHX2	4	2572	0.00155521
ZNF799	1	643	0.00155521
LCT	3	1927	0.001556824
IVNS1ABP	1	642	0.001557632
NDC80	1	642	0.001557632
ZNF699	1	642	0.001557632
CAPN6	1	641	0.001560062
ZNF709	1	641	0.001560062
ALAS1	1	640	0.0015625
LRRC4C	1	640	0.0015625
KIAA1107	2	1279	0.001563722
CYFIP2	2	1278	0.001564945
EYA4	1	639	0.001564945
GGA1	1	639	0.001564945
ILDR2	1	639	0.001564945
KRT2	1	639	0.001564945
ZNF182	1	639	0.001564945
GBP7	1	638	0.001567398
NOL4	1	638	0.001567398
PCSK2	1	638	0.001567398
SRPRA	1	638	0.001567398
ULK4	2	1275	0.001568627
GCLC	1	637	0.001569859
RAB11FIP4	1	637	0.001569859

ARNTL2	1	636	0.001572327
MOCS1	1	636	0.001572327
NAF1	1	636	0.001572327
ODF2L	1	636	0.001572327
PAPOLB	1	636	0.001572327
SLC6A7	1	636	0.001572327
TP73	1	636	0.001572327
ZFP90	1	636	0.001572327
ZNF790	1	636	0.001572327
ATF7IP	2	1270	0.001574803
LRFN4	1	635	0.001574803
LRP10	3	1905	0.001574803
MPL	1	635	0.001574803
SYK	1	635	0.001574803
KLHL22	1	634	0.001577287
KLHL31	1	634	0.001577287
GBP6	1	633	0.001579779
HAUS5	1	633	0.001579779
ARMCX2	1	632	0.001582278
GOLGA8H	1	632	0.001582278
MIGA1	1	632	0.001582278
ZNF792	1	632	0.001582278
CCDC93	1	631	0.001584786
DDX53	1	631	0.001584786
DEF6	1	631	0.001584786
SMPD1	1	631	0.001584786
ARHGAP29	2	1261	0.001586043
CCDC120	1	630	0.001587302
EIF2AK1	1	630	0.001587302
PP2D1	1	630	0.001587302
SHC4	1	630	0.001587302
SLC3A2	1	630	0.001587302
TRIM25	1	630	0.001587302
DYRK1B	1	629	0.001589825
IL23R	1	629	0.001589825
ASH2L	1	628	0.001592357
NTN4	1	628	0.001592357
SNX18	1	628	0.001592357
FEM1B	1	627	0.001594896
SUCO	2	1254	0.001594896
ZNF442	1	627	0.001594896
ZNF462	4	2506	0.001596169
PEX5L	1	626	0.001597444
ZNF729	2	1252	0.001597444
ERCC6L	2	1250	0.0016
GCKR	1	625	0.0016
GRHL2	1	625	0.0016

CAMSAP3	2	1249	0.001601281
LRRIQ3	1	624	0.001602564
IRGQ	1	623	0.001605136
KBTBD11	1	623	0.001605136
KBTBD12	1	623	0.001605136
KRT9	1	623	0.001605136
L3MBTL4	1	623	0.001605136
MAK	1	623	0.001605136
COLGALT1	1	622	0.001607717
DTX2	1	622	0.001607717
FANCG	1	622	0.001607717
FOXJ3	1	622	0.001607717
GALNT6	1	622	0.001607717
PRLR	1	622	0.001607717
TTBK2	2	1244	0.001607717
ZNF728	1	622	0.001607717
ATP2B2	2	1243	0.00160901
PPIP5K2	2	1243	0.00160901
ZFAT	2	1243	0.00160901
KIF1BP	1	621	0.001610306
KLHL6	1	621	0.001610306
ATP2B2	2	1241	0.001611604
ATP2B4	2	1241	0.001611604
IMPG2	2	1241	0.001611604
CDC16	1	620	0.001612903
COL27A1	3	1860	0.001612903
ITK	1	620	0.001612903
KLHL8	1	620	0.001612903
PRC1	1	620	0.001612903
RANBP10	1	620	0.001612903
SLC27A2	1	620	0.001612903
ZNF93	1	620	0.001612903
GBF1	3	1859	0.001613771
KMT2E	3	1858	0.001614639
CFAP54	5	3096	0.001614987
ATG16L2	1	619	0.001615509
CRTC3	1	619	0.001615509
DTX4	1	619	0.001615509
GUCY1B3	1	619	0.001615509
TCF4	1	619	0.001615509
CFAP61	2	1237	0.001616815
RASGRF2	2	1237	0.001616815
RHOT1	1	618	0.001618123
RHOT2	1	618	0.001618123
SLC5A12	1	618	0.001618123
ZNF48	1	618	0.001618123
BRD8	2	1235	0.001619433

FEM1C	1	617	0.001620746
FNBP1	1	617	0.001620746
GLCE	1	617	0.001620746
SCG2	1	617	0.001620746
SLC6A2	1	617	0.001620746
SYVN1	1	617	0.001620746
VPS33B	1	617	0.001620746
ZNF221	1	617	0.001620746
SBF2	3	1849	0.001622499
HIPK4	1	616	0.001623377
LBR	1	615	0.001626016
NUB1	1	615	0.001626016
PRICKLE3	1	615	0.001626016
SCG2	1	615	0.001626016
VGF	1	615	0.001626016
ACHE	1	614	0.001628664
PAPSS2	1	614	0.001628664
SLC6A12	1	614	0.001628664
ZSCAN2	1	614	0.001628664
ADAR	2	1226	0.001631321
CCDC168	4	2452	0.001631321
GGA2	1	613	0.001631321
MAU2	1	613	0.001631321
TTC39A	1	613	0.001631321
CTNND2	2	1225	0.001632653
STAG3	2	1225	0.001632653
BTBD9	1	612	0.001633987
COPA	2	1224	0.001633987
ZNF354B	1	612	0.001633987
PHKA1	2	1223	0.001635323
ALG9	1	611	0.001636661
FAM111A	1	611	0.001636661
RHOBTB3	1	611	0.001636661
SORCS3	2	1222	0.001636661
BOD1L1	5	3051	0.001638807
DHX8	2	1220	0.001639344
DOCK2	3	1830	0.001639344
MYNN	1	610	0.001639344
SEC31A	2	1220	0.001639344
SLC23A3	1	610	0.001639344
ZNF667	1	610	0.001639344
ALB	1	609	0.001642036
JAG1	2	1218	0.001642036
RASGRP2	1	609	0.001642036
SAP130	2	1217	0.001643385
DENND6A	1	608	0.001644737
KIAA1468	2	1216	0.001644737

LAMA4	3	1823	0.001645639
GPR158	2	1215	0.001646091
ZSWIM6	2	1215	0.001646091
BRPF1	2	1214	0.001647446
CUZD1	1	607	0.001647446
FPGT	1	607	0.001647446
GALNT18	1	607	0.001647446
RAP1GDS1	1	607	0.001647446
RYK	1	607	0.001647446
SMARCC2	2	1214	0.001647446
TBX18	1	607	0.001647446
ZEB2	2	1214	0.001647446
DSEL	2	1212	0.001650165
GRM5	2	1212	0.001650165
MAGED2	1	606	0.001650165
RUFY2	1	606	0.001650165
WDR1	1	606	0.001650165
ZNF652	1	606	0.001650165
MOV10L1	2	1211	0.001651528
DMXL1	5	3027	0.0016518
FNBP1L	1	605	0.001652893
IGFALS	1	605	0.001652893
MBD1	1	605	0.001652893
USP2	1	605	0.001652893
HERC2	8	4834	0.001654944
BIRC3	1	604	0.001655629
CHL1	2	1208	0.001655629
KLHL15	1	604	0.001655629
PTGS2	1	604	0.001655629
EGF	2	1207	0.001657001
PRSS56	1	603	0.001658375
FRY	5	3013	0.001659476
CKAP4	1	602	0.00166113
LRRC40	1	602	0.00166113
SLC47A2	1	602	0.00166113
ZMYND11	1	602	0.00166113
TRIP10	1	601	0.001663894
DCAF8L1	1	600	0.001666667
INTS11	1	600	0.001666667
KIAA1755	2	1200	0.001666667
KRT84	1	600	0.001666667
MYEF2	1	600	0.001666667
ST6GALNAC1	1	600	0.001666667
TAF2	2	1199	0.001668057
AFM	1	599	0.001669449
ANAPC7	1	599	0.001669449
ARSJ	1	599	0.001669449

CENPB	1	599	0.001669449
DDX52	1	599	0.001669449
DENND3	2	1198	0.001669449
IL18RAP	1	599	0.001669449
CDYL	1	598	0.001672241
LILRB2	1	598	0.001672241
MTMR4	2	1195	0.00167364
CEP70	1	597	0.001675042
FRMD3	1	597	0.001675042
GAN	1	597	0.001675042
PAPPA2	3	1791	0.001675042
RNGTT	1	597	0.001675042
EEFSEC	1	596	0.001677852
NR2C2	1	596	0.001677852
ARHGAP20	2	1191	0.001679261
WNK1	4	2382	0.001679261
GBP3	1	595	0.001680672
ATP8A2	2	1188	0.001683502
CDC14A	1	594	0.001683502
GAD1	1	594	0.001683502
ASH1L	5	2969	0.001684069
LINGO4	1	593	0.001686341
MIGA2	1	593	0.001686341
PDE9A	1	593	0.001686341
PTH1R	1	593	0.001686341
ZNF284	1	593	0.001686341
ANKRD36C	3	1778	0.001687289
CILP	2	1184	0.001689189
EYA1	1	592	0.001689189
FBLN2	2	1184	0.001689189
GBP1	1	592	0.001689189
HPSE2	1	592	0.001689189
RNF180	1	592	0.001689189
CTPS1	1	591	0.001692047
SPATC1	1	591	0.001692047
USP34	6	3546	0.001692047
KIAA2012	2	1180	0.001694915
LILRB5	1	590	0.001694915
LRRTM4	1	590	0.001694915
NPAS1	1	590	0.001694915
SYT3	1	590	0.001694915
CSRNP1	1	589	0.001697793
EGR4	1	589	0.001697793
FARSB	1	589	0.001697793
PLBD2	1	589	0.001697793
POF1B	1	589	0.001697793
PPP2R1A	1	589	0.001697793

ZBTB46	1	589	0.001697793
ZNF806	1	589	0.001697793
PES1	1	588	0.00170068
SLFN5	1	588	0.00170068
ALAS2	1	587	0.001703578
ASB14	1	587	0.001703578
IRF2BP2	1	587	0.001703578
KLHL3	1	587	0.001703578
MAPK4	1	587	0.001703578
SACM1L	1	587	0.001703578
TCTN1	1	587	0.001703578
ROS1	4	2347	0.001704303
PDE4DIP	4	2346	0.00170503
SLF2	2	1173	0.00170503
ACSM3	1	586	0.001706485
EZR	1	586	0.001706485
LAMB3	2	1172	0.001706485
RFX8	1	586	0.001706485
SLU7	1	586	0.001706485
PCNX1	4	2341	0.001708672
BCOR	3	1755	0.001709402
BEST1	1	585	0.001709402
CD96	1	585	0.001709402
DLG3	1	585	0.001709402
EIF2A	1	585	0.001709402
LGALS3BP	1	585	0.001709402
UBR2	3	1755	0.001709402
EIF2D	1	584	0.001712329
IPP	1	584	0.001712329
ZBTB7A	1	584	0.001712329
CFI	1	583	0.001715266
IFT172	3	1749	0.001715266
LRRC47	1	583	0.001715266
N4BP2L2	1	583	0.001715266
NUDCD1	1	583	0.001715266
C2orf81	1	582	0.001718213
DCP1A	1	582	0.001718213
FAM149B1	1	582	0.001718213
FKBP10	1	582	0.001718213
SHOC2	1	582	0.001718213
ZNF319	1	582	0.001718213
ZNF326	1	582	0.001718213
AFF4	2	1163	0.00171969
ITGAX	2	1163	0.00171969
FSIP1	1	581	0.00172117
LRRC15	1	581	0.00172117
ZNF674	1	581	0.00172117

FBXO24	1	580	0.001724138
NELFB	1	580	0.001724138
PHACTR1	1	580	0.001724138
POMGNT2	1	580	0.001724138
SYN3	1	580	0.001724138
WASHC5	2	1159	0.001725626
PRRC2C	5	2896	0.001726519
BCO2	1	579	0.001727116
KPRP	1	579	0.001727116
NR1D2	1	579	0.001727116
XKR7	1	579	0.001727116
CFHR4	1	578	0.001730104
HACL1	1	578	0.001730104
NPAP1	2	1156	0.001730104
RFTN1	1	578	0.001730104
ACSM2B	1	577	0.001733102
IGF2BP1	1	577	0.001733102
KIAA1328	1	577	0.001733102
MSN	1	577	0.001733102
TBL1X	1	577	0.001733102
ZNF384	1	577	0.001733102
BAG3	1	575	0.00173913
EBF2	1	575	0.00173913
THBD	1	575	0.00173913
ZNF417	1	575	0.00173913
FBN1	5	2871	0.001741553
CDC7	1	574	0.00174216
NT5E	1	574	0.00174216
PCDH19	2	1148	0.00174216
SNX33	1	574	0.00174216
SCAF4	2	1147	0.001743679
POLR1A	3	1720	0.001744186
TOGARAM1	3	1720	0.001744186
BICDL1	1	573	0.001745201
GMEB1	1	573	0.001745201
KLC1	1	573	0.001745201
MROH9	1	573	0.001745201
SLC41A2	1	573	0.001745201
ADCY3	2	1144	0.001748252
EFCAB12	1	572	0.001748252
GPC5	1	572	0.001748252
GPNMB	1	572	0.001748252
RTP5	1	572	0.001748252
SZT2	6	3432	0.001748252
COL24A1	3	1714	0.001750292
ENAM	2	1142	0.001751313
GALNT2	1	571	0.001751313

MAGEC1	2	1142	0.001751313
PDZD3	1	571	0.001751313
SLC40A1	1	571	0.001751313
ZMYM1	2	1142	0.001751313
COL4A2	3	1712	0.001752336
PDE3A	2	1141	0.001752848
SREBF2	2	1141	0.001752848
TTC17	2	1141	0.001752848
CEP152	3	1710	0.001754386
DPYSL3	1	570	0.001754386
TBC1D8	2	1140	0.001754386
SIGLEC1	3	1709	0.001755413
MEX3B	1	569	0.001757469
PRKAG2	1	569	0.001757469
TTC22	1	569	0.001757469
ALG13	2	1137	0.001759015
ST5	2	1137	0.001759015
ACCSL	1	568	0.001760563
MLLT3	1	568	0.001760563
TRAF3	1	568	0.001760563
ZNF358	1	568	0.001760563
ZNF648	1	568	0.001760563
ANKS1A	2	1134	0.001763668
CES1	1	567	0.001763668
CES2	1	567	0.001763668
RABGGTA	1	567	0.001763668
TGFBR2	1	567	0.001763668
WEE2	1	567	0.001763668
AHCTF1	4	2266	0.001765225
BTBD7	2	1132	0.001766784
CDC45	1	566	0.001766784
INPP5F	2	1132	0.001766784
JAK2	2	1132	0.001766784
MCOLN2	1	566	0.001766784
MIS18BP1	2	1132	0.001766784
TMEM151B	1	566	0.001766784
TTC34	1	566	0.001766784
ADNP2	2	1131	0.001768347
ABL1	2	1130	0.001769912
SPN	1	565	0.001769912
PLCH1	3	1693	0.001772002
COASY	1	564	0.00177305
SLC1A6	1	564	0.00177305
COL4A6	3	1691	0.001774098
PKDREJ	4	2253	0.001775411
CCDC63	1	563	0.001776199
MCCC2	1	563	0.001776199

ZNF529	1	563	0.001776199
AGFG1	1	562	0.001779359
ANLN	2	1124	0.001779359
ASXL3	4	2248	0.001779359
JAK3	2	1124	0.001779359
MMP25	1	562	0.001779359
PLAT	1	562	0.001779359
ZNF460	1	562	0.001779359
MUC17	8	4493	0.001780548
USP36	2	1123	0.001780944
USP54	3	1684	0.001781473
ARID3B	1	561	0.001782531
SH3BP2	1	561	0.001782531
SLC38A9	1	561	0.001782531
GP5	1	560	0.001785714
PRIMPOL	1	560	0.001785714
MDN1	10	5596	0.001786991
ADCY1	2	1119	0.00178731
TRPA1	2	1119	0.00178731
BORA	1	559	0.001788909
C9	1	559	0.001788909
CES2	1	559	0.001788909
DCAF10	1	559	0.001788909
NXPE3	1	559	0.001788909
PHACTR3	1	559	0.001788909
YTHDF1	1	559	0.001788909
KIAA0100	4	2235	0.001789709
CEP126	2	1117	0.00179051
DGKZ	2	1117	0.00179051
GBP3	1	558	0.001792115
GLUD2	1	558	0.001792115
ISYNA1	1	558	0.001792115
SPATS2L	1	558	0.001792115
TEX15	5	2789	0.001792757
CAMTA1	3	1673	0.001793186
ARIH1	1	557	0.001795332
CNOT6	1	557	0.001795332
CPNE4	1	557	0.001795332
GATB	1	557	0.001795332
TRAF5	1	557	0.001795332
CAD	4	2225	0.001797753
GALNT13	1	556	0.001798561
HIRIP3	1	556	0.001798561
UTP18	1	556	0.001798561
SPAG17	4	2223	0.00179937
CFAP43	3	1665	0.001801802
SLC22A20	1	555	0.001801802

SMOX	1	555	0.001801802
ZNF57	1	555	0.001801802
C3	3	1663	0.001803969
DNAJC1	1	554	0.001805054
PIGB	1	554	0.001805054
ZNF205	1	554	0.001805054
ASPSCR1	1	553	0.001808318
CCDC85A	1	553	0.001808318
FOXC1	1	553	0.001808318
GLP2R	1	553	0.001808318
IL20RA	1	553	0.001808318
PREX1	3	1659	0.001808318
PRSS53	1	553	0.001808318
SLC22A12	1	553	0.001808318
C12orf4	1	552	0.001811594
PTBP3	1	552	0.001811594
GUCY2D	2	1103	0.001813237
CROCC2	3	1653	0.001814882
PALMD	1	551	0.001814882
PIK3CG	2	1102	0.001814882
RCBTB2	1	551	0.001814882
RELA	1	551	0.001814882
SLC22A13	1	551	0.001814882
TPTE	1	551	0.001814882
TRIM8	1	551	0.001814882
CCDC102A	1	550	0.001818182
CTTN	1	550	0.001818182
FMNL1	2	1100	0.001818182
IDS	1	550	0.001818182
SRGAP3	2	1099	0.001819836
KIF14	3	1648	0.001820388
GNL3	1	549	0.001821494
MTMR8	1	549	0.001821494
MYO1F	2	1098	0.001821494
NECTIN3	1	549	0.001821494
SPNS2	1	549	0.001821494
TIGD7	1	549	0.001821494
LIFR	2	1097	0.001823154
REST	2	1097	0.001823154
CPNE2	1	548	0.001824818
LARP1	2	1096	0.001824818
LGI3	1	548	0.001824818
PHF2	2	1096	0.001824818
SLC22A7	1	548	0.001824818
INTS1	4	2190	0.001826484
DDX21	1	547	0.001828154
ELP3	1	547	0.001828154

ICAM3	1	547	0.001828154
ITPRIP	1	547	0.001828154
LACTB	1	547	0.001828154
LMOD2	1	547	0.001828154
SLC22A15	1	547	0.001828154
SLC22A25	1	547	0.001828154
SLC38A4	1	547	0.001828154
TXLNA	1	546	0.001831502
AIRE	1	545	0.001834862
MIER2	1	545	0.001834862
PAK1	1	545	0.001834862
PPP1R13B	2	1090	0.001834862
RPUSD2	1	545	0.001834862
FREM1	4	2179	0.001835704
CCDC82	1	544	0.001838235
RANBP17	2	1088	0.001838235
HIVEP1	5	2718	0.001839588
UBAP2L	2	1087	0.001839926
CDS1	1	543	0.001841621
CHEK2	1	543	0.001841621
DALRD3	1	543	0.001841621
EGR1	1	543	0.001841621
EHD2	1	543	0.001841621
HDC	1	543	0.001841621
KCNN1	1	543	0.001841621
NEFL	1	543	0.001841621
SDK2	4	2172	0.001841621
ZCCHC7	1	543	0.001841621
ZNF280B	1	543	0.001841621
PAPPA	3	1627	0.001843884
BAZ2B	4	2168	0.001845018
LIN9	1	542	0.001845018
PGM3	1	542	0.001845018
SLC1A3	1	542	0.001845018
TERF2	1	542	0.001845018
UBE3C	2	1083	0.001846722
AP3B2	2	1082	0.001848429
ATL3	1	541	0.001848429
NR5A2	1	541	0.001848429
PLA2G4C	1	541	0.001848429
SLC2A10	1	541	0.001848429
SUGP2	2	1082	0.001848429
IPO4	2	1081	0.001850139
NES	3	1621	0.001850709
KRT73	1	540	0.001851852
PIP5K1B	1	540	0.001851852
SNTB2	1	540	0.001851852

TENM3	5	2699	0.001852538
PCCB	1	539	0.001855288
ANKRD52	2	1076	0.001858736
ERVFRD-1	1	538	0.001858736
EYA2	1	538	0.001858736
KATNAL2	1	538	0.001858736
LIPE	2	1076	0.001858736
RBBP5	1	538	0.001858736
SLC22A17	1	538	0.001858736
TROVE2	1	538	0.001858736
ZKSCAN3	1	538	0.001858736
ZNF554	1	538	0.001858736
GP2	1	537	0.001862197
PDP1	1	537	0.001862197
PLD1	2	1074	0.001862197
SLC9B2	1	537	0.001862197
VPS13C	7	3753	0.001865175
ARSK	1	536	0.001865672
C3orf30	1	536	0.001865672
CARD9	1	536	0.001865672
GBA	1	536	0.001865672
HSF2	1	536	0.001865672
PLD5	1	536	0.001865672
RNF31	2	1072	0.001865672
SRC	1	536	0.001865672
EHD2	1	535	0.001869159
EHD3	1	535	0.001869159
FAM196B	1	535	0.001869159
NRBP1	1	535	0.001869159
PCDH8	2	1070	0.001869159
PDE1A	1	535	0.001869159
ZNF480	1	535	0.001869159
CAMSAP1	3	1602	0.001872659
CD276	1	534	0.001872659
POLR3C	1	534	0.001872659
SNRNP200	4	2136	0.001872659
STK35	1	534	0.001872659
UGT1A4	1	534	0.001872659
UGT1A5	1	534	0.001872659
ZNF397	1	534	0.001872659
B4GALNT1	1	533	0.001876173
FMO5	1	533	0.001876173
MGAT3	1	533	0.001876173
NT5C1B-RDH14	1	533	0.001876173
PUM2	2	1066	0.001876173
RPE65	1	533	0.001876173
SLC37A1	1	533	0.001876173

ZFP14	1	533	0.001876173
BMPR1A	1	532	0.001879699
CHRM5	1	532	0.001879699
SLC18A3	1	532	0.001879699
ZFP82	1	532	0.001879699
RB1CC1	3	1594	0.001882058
HHLA1	1	531	0.001883239
NXF3	1	531	0.001883239
PAF1	1	531	0.001883239
PHF21B	1	531	0.001883239
PRMT3	1	531	0.001883239
RIC8A	1	531	0.001883239
SMARCD2	1	531	0.001883239
TMEM266	1	531	0.001883239
ZGPAT	1	531	0.001883239
ZNF492	1	531	0.001883239
ZNF837	1	531	0.001883239
ANKRD53	1	530	0.001886792
CCT6B	1	530	0.001886792
FAM126B	1	530	0.001886792
M1AP	1	530	0.001886792
NTNG2	1	530	0.001886792
RBM27	2	1060	0.001886792
UGT1A10	1	530	0.001886792
ATP5B	1	529	0.001890359
BAIAP2L2	1	529	0.001890359
BBOF1	1	529	0.001890359
HEXA	1	529	0.001890359
KPNA2	1	529	0.001890359
HERC4	2	1057	0.001892148
ADGRB1	3	1584	0.001893939
ALPI	1	528	0.001893939
KDM4C	2	1056	0.001893939
TH	1	528	0.001893939
BTN2A1	1	527	0.001897533
CAT	1	527	0.001897533
OSR1	1	527	0.001897533
OXSR1	1	527	0.001897533
UNC79	5	2635	0.001897533
ARHGEF3	1	526	0.001901141
FUS	1	526	0.001901141
TSEN54	1	526	0.001901141
ZGRF1	4	2104	0.001901141
KIAA2026	4	2103	0.001902045
DNMBP	3	1577	0.001902346
BUB1B	2	1050	0.001904762
CORO2A	1	525	0.001904762

HERC3	2	1050	0.001904762
SLC32A1	1	525	0.001904762
SOWAHC	1	525	0.001904762
BDP1	5	2624	0.001905488
API5	1	524	0.001908397
ITGAV	2	1048	0.001908397
LRRC1	1	524	0.001908397
PLEKHA6	2	1048	0.001908397
PPP2R5C	1	524	0.001908397
SAP130	2	1048	0.001908397
ZNF479	1	524	0.001908397
ZNF683	1	524	0.001908397
DIP2A	3	1571	0.001909612
ATP9A	2	1047	0.00191022
NPR2	2	1047	0.00191022
GPT2	1	523	0.001912046
MAGEE2	1	523	0.001912046
NPEPL1	1	523	0.001912046
PSPC1	1	523	0.001912046
SIAE	1	523	0.001912046
SLC16A6	1	523	0.001912046
SLC35F5	1	523	0.001912046
SLC7A10	1	523	0.001912046
ZNF765	1	523	0.001912046
CCDC129	2	1044	0.001915709
CYP26C1	1	522	0.001915709
GIN1	1	522	0.001915709
LRRTM1	1	522	0.001915709
MB21D1	1	522	0.001915709
RCC2	1	522	0.001915709
SOAT2	1	522	0.001915709
TPTE2	1	522	0.001915709
TRAF6	1	522	0.001915709
CASP10	1	521	0.001919386
FAM126A	1	521	0.001919386
KPNA3	1	521	0.001919386
MSL3	1	521	0.001919386
PNKP	1	521	0.001919386
RUNX2	1	521	0.001919386
SIPA1	2	1042	0.001919386
VANGL2	1	521	0.001919386
VEZF1	1	521	0.001919386
NACAD	3	1562	0.001920615
CEP295	5	2601	0.001922338
CREB3L2	1	520	0.001923077
DCAF17	1	520	0.001923077
DSG4	2	1040	0.001923077

DYRK4	1	520	0.001923077
HMGCS1	1	520	0.001923077
KRT4	1	520	0.001923077
MARCO	1	520	0.001923077
MEX3A	1	520	0.001923077
MMP25	1	520	0.001923077
NT5DC2	1	520	0.001923077
SMG9	1	520	0.001923077
SPATA2	1	520	0.001923077
FRMD4A	2	1039	0.001924928
BEND7	1	519	0.001926782
IRX4	1	519	0.001926782
KCNG4	1	519	0.001926782
POTEJ	2	1038	0.001926782
SNX2	1	519	0.001926782
DIP2C	3	1556	0.001928021
GNAS	2	1037	0.00192864
ALDH1A2	1	518	0.001930502
ANKRD13D	1	518	0.001930502
ATE1	1	518	0.001930502
SARS2	1	518	0.001930502
TBX5	1	518	0.001930502
FAM47C	2	1035	0.001932367
AEBP2	1	517	0.001934236
ATG13	1	517	0.001934236
CHRND	1	517	0.001934236
FRMD4B	2	1034	0.001934236
LAD1	1	517	0.001934236
SLTM	2	1034	0.001934236
SNTG1	1	517	0.001934236
TRABD2B	1	517	0.001934236
TRIM65	1	517	0.001934236
USP30	1	517	0.001934236
ZNF582	1	517	0.001934236
CARD10	2	1032	0.001937984
LRRN2	1	516	0.001937984
LRRTM2	1	516	0.001937984
RTN3	2	1032	0.001937984
TSTD2	1	516	0.001937984
FAR1	1	515	0.001941748
FAR2	1	515	0.001941748
GJA9	1	515	0.001941748
IFNAR2	1	515	0.001941748
RNF38	1	515	0.001941748
ZNF695	1	515	0.001941748
KIF17	2	1029	0.001943635
ANKRD34B	1	514	0.001945525

APCDD1	1	514	0.001945525
CYP24A1	1	514	0.001945525
DKC1	1	514	0.001945525
IMPDH1	1	514	0.001945525
SLC18A2	1	514	0.001945525
STK33	1	514	0.001945525
TBL1XR1	1	514	0.001945525
IQCH	2	1027	0.00194742
CCDC102B	1	513	0.001949318
LRRC24	1	513	0.001949318
NEFH	2	1026	0.001949318
ACVR2B	1	512	0.001953125
CYP26B1	1	512	0.001953125
ETV3	1	512	0.001953125
KLF11	1	512	0.001953125
PODNL1	1	512	0.001953125
TIGD4	1	512	0.001953125
ARCN1	1	511	0.001956947
GIMAP1-GIMAP5	1	511	0.001956947
PAOX	1	511	0.001956947
PRDM11	1	511	0.001956947
TRIM7	1	511	0.001956947
RAB44	2	1021	0.001958864
CAST	1	510	0.001960784
ENTPD1	1	510	0.001960784
FBXO15	1	510	0.001960784
FH	1	510	0.001960784
PIAS4	1	510	0.001960784
SLC16A14	1	510	0.001960784
ZSCAN18	1	510	0.001960784
PLEKHM2	2	1019	0.001962709
STK31	2	1019	0.001962709
CYP4X1	1	509	0.001964637
DLD	1	509	0.001964637
HARS	1	509	0.001964637
IKZF3	1	509	0.001964637
LCK	1	509	0.001964637
LGSN	1	509	0.001964637
NFIA	1	509	0.001964637
PRAME	1	509	0.001964637
TRMT5	1	509	0.001964637
HCFC1	4	2035	0.001965602
ANKHD1	5	2542	0.001966955
ABI1	1	508	0.001968504
CKAP5	4	2032	0.001968504
EPOR	1	508	0.001968504
FAM227B	1	508	0.001968504

GPR101	1	508	0.001968504
HMGCS2	1	508	0.001968504
MMP19	1	508	0.001968504
NFIC	1	508	0.001968504
TOPBP1	3	1522	0.001971091
ARSA	1	507	0.001972387
LDHD	1	507	0.001972387
MATK	1	507	0.001972387
MKRN3	1	507	0.001972387
PARP1	2	1014	0.001972387
RASSF10	1	507	0.001972387
SLC41A3	1	507	0.001972387
SLC7A5	1	507	0.001972387
UAP1L1	1	507	0.001972387
XPNPEP3	1	507	0.001972387
GARNL3	2	1013	0.001974334
CDK5RAP3	1	506	0.001976285
CDYL2	1	506	0.001976285
GCM2	1	506	0.001976285
IARS2	2	1012	0.001976285
NOS1AP	1	506	0.001976285
PLD4	1	506	0.001976285
SLC38A2	1	506	0.001976285
ANKRD18B	2	1011	0.001978239
FAM83B	2	1011	0.001978239
MAN2B1	2	1011	0.001978239
TRPM6	4	2022	0.001978239
ASIC5	1	505	0.001980198
ATF2	1	505	0.001980198
PDIA3	1	505	0.001980198
SLC16A5	1	505	0.001980198
TEX45	1	505	0.001980198
DNAJC3	1	504	0.001984127
EPHA10	2	1008	0.001984127
GPRIN1	2	1008	0.001984127
ONECUT2	1	504	0.001984127
PRPF19	1	504	0.001984127
SIRPA	1	504	0.001984127
SLC16A8	1	504	0.001984127
YAP1	1	504	0.001984127
ZNF596	1	504	0.001984127
ACVRL1	1	503	0.001988072
CYP11B1	1	503	0.001988072
CYP3A7	1	503	0.001988072
CYP51A1	1	503	0.001988072
INPP5J	2	1006	0.001988072
LEMD2	1	503	0.001988072

THUMPD2	1	503	0.001988072
ZBTB37	1	503	0.001988072
ZNF730	1	503	0.001988072
SCN1A	4	2009	0.00199104
BMPR1B	1	502	0.001992032
CYP2J2	1	502	0.001992032
KDELC1	1	502	0.001992032
NFIX	1	502	0.001992032
PHF12	2	1004	0.001992032
WAS	1	502	0.001992032
CUX1	3	1505	0.001993355
ANO2	2	1003	0.001994018
SCN2A	4	2005	0.001995012
CACNA1A	5	2506	0.001995211
ACCS	1	501	0.001996008
EFCAB5	3	1503	0.001996008
GDF5	1	501	0.001996008
SEPSECS	1	501	0.001996008
SLC37A2	1	501	0.001996008
SLC7A11	1	501	0.001996008
TCTE1	1	501	0.001996008
GPATCH8	3	1502	0.001997337
RNF40	2	1001	0.001998002
ENDOD1	1	500	0.002
NFKBIE	1	500	0.002
RBPJ	1	500	0.002
SERPING1	1	500	0.002
SLC24A5	1	500	0.002
TRIM69	1	500	0.002
MERTK	2	999	0.002002002
NCAPD3	3	1498	0.00200267
CCDC105	1	499	0.002004008
KCNA2	1	499	0.002004008
KCNK5	1	499	0.002004008
KIF19	2	998	0.002004008
LIPC	1	499	0.002004008
PPP5C	1	499	0.002004008
PRPF31	1	499	0.002004008
SERPIND1	1	499	0.002004008
ZNF727	1	499	0.002004008
IGF2R	5	2491	0.002007226
MBD5	3	1494	0.002008032
WASF2	1	498	0.002008032
ZNF497	1	498	0.002008032
ABCC10	3	1492	0.002010724
ZFC3H1	4	1989	0.002011061
ADPGK	1	497	0.002012072

BNC1	2	994	0.002012072
CYP26A1	1	497	0.002012072
NLRP4	2	994	0.002012072
PPP2R5B	1	497	0.002012072
RETREG1	1	497	0.002012072
RNF111	2	994	0.002012072
EMC1	2	993	0.002014099
ANGPT2	1	496	0.002016129
ANKRD18A	2	992	0.002016129
C2orf16	4	1984	0.002016129
SLC2A3	1	496	0.002016129
NLRP9	2	991	0.002018163
RECQL5	2	991	0.002018163
A1BG	1	495	0.002020202
EFCAB14	1	495	0.002020202
KCNA1	1	495	0.002020202
MCM3AP	4	1980	0.002020202
RAD18	1	495	0.002020202
RGS7	1	495	0.002020202
SHE	1	495	0.002020202
SLC17A5	1	495	0.002020202
GRIN2B	3	1484	0.002021563
NOTCH2	5	2471	0.002023472
ANKRD33B	1	494	0.002024291
CYP2A13	1	494	0.002024291
GPR135	1	494	0.002024291
NAF1	1	494	0.002024291
PCYOX1L	1	494	0.002024291
SRSF4	1	494	0.002024291
WDR37	1	494	0.002024291
ZFP36L2	1	494	0.002024291
EPHB4	2	987	0.002026342
KANSL1L	2	987	0.002026342
BMP1	2	986	0.002028398
CHRNE	1	493	0.002028398
DUS2	1	493	0.002028398
EFEMP1	1	493	0.002028398
FSCN1	1	493	0.002028398
PIGV	1	493	0.002028398
TP53BP1	4	1972	0.002028398
ALG11	1	492	0.00203252
DYNC1LI2	1	492	0.00203252
KRBA2	1	492	0.00203252
MORC1	2	984	0.00203252
PPIL4	1	492	0.00203252
EPHA3	2	983	0.002034588
A2M	3	1474	0.002035278

FBXL7	1	491	0.00203666
FGB	1	491	0.00203666
KATNA1	1	491	0.00203666
LETM2	1	491	0.00203666
SYT9	1	491	0.00203666
TMEM200A	1	491	0.00203666
ZSCAN22	1	491	0.00203666
ZNF469	8	3925	0.002038217
ANO3	2	981	0.002038736
AP5M1	1	490	0.002040816
FOXN3	1	490	0.002040816
MDM4	1	490	0.002040816
NLRP7	2	980	0.002040816
SMYD1	1	490	0.002040816
TPH2	1	490	0.002040816
ARHGAP21	4	1958	0.002042901
NEK8	2	979	0.002042901
PTPRN	2	979	0.002042901
HIVEP2	5	2446	0.002044154
LMBR1L	1	489	0.00204499
SCN10A	4	1956	0.00204499
ZNF772	1	489	0.00204499
COL3A1	3	1466	0.002046385
INPP4A	2	977	0.002047083
ZC3H7B	2	977	0.002047083
ANXA7	1	488	0.00204918
CCDC77	1	488	0.00204918
COL1A1	3	1464	0.00204918
GRIN2A	3	1464	0.00204918
LRRC74A	1	488	0.00204918
NCOA2	3	1464	0.00204918
NCOR1	5	2440	0.00204918
PTGER4	1	488	0.00204918
UHRF1BP1L	3	1464	0.00204918
FGD5	3	1462	0.002051984
ABCA2	5	2435	0.002053388
NEO1	3	1461	0.002053388
RPRD2	3	1461	0.002053388
SLC7A9	1	487	0.002053388
TRIM50	1	487	0.002053388
ZBTB32	1	487	0.002053388
ZIK1	1	487	0.002053388
CEP164	3	1460	0.002054795
ERG	1	486	0.002057613
KRT86	1	486	0.002057613
MECP2	1	486	0.002057613
TRIM58	1	486	0.002057613

AGT	1	485	0.002061856
BCL2L13	1	485	0.002061856
CELF5	1	485	0.002061856
CHL1	2	970	0.002061856
POLG2	1	485	0.002061856
SPAG8	1	485	0.002061856
ZNF639	1	485	0.002061856
CCDC18	3	1454	0.002063274
CACNB3	1	484	0.002066116
CCDC65	1	484	0.002066116
CTSF	1	484	0.002066116
PKD2	2	968	0.002066116
STK10	2	968	0.002066116
MYH7	4	1935	0.002067183
ATP6V1H	1	483	0.002070393
CCDC47	1	483	0.002070393
DDX6	1	483	0.002070393
ERLEC1	1	483	0.002070393
PRAMEF12	1	483	0.002070393
UPF3B	1	483	0.002070393
ZNF774	1	483	0.002070393
DBT	1	482	0.002074689
G3BP2	1	482	0.002074689
HDAC1	1	482	0.002074689
TRIM37	2	964	0.002074689
PACS1	2	963	0.002076843
PCDHAC1	2	963	0.002076843
SH3BP4	2	963	0.002076843
AGFG2	1	481	0.002079002
AKT2	1	481	0.002079002
CELF6	1	481	0.002079002
HTR2B	1	481	0.002079002
LILRA6	1	481	0.002079002
PI4K2B	1	481	0.002079002
DDC	1	480	0.002083333
EDIL3	1	480	0.002083333
HTRA1	1	480	0.002083333
NR6A1	1	480	0.002083333
PROSER3	1	480	0.002083333
PXYLP1	1	480	0.002083333
UHRF1BP1	3	1440	0.002083333
XYLT1	2	959	0.002085506
BCL6B	1	479	0.002087683
CCDC33	2	958	0.002087683
PAIP1	1	479	0.002087683
SLITRK5	2	958	0.002087683
UTP3	1	479	0.002087683

ERC2	2	957	0.002089864
MYLK	4	1914	0.002089864
HFM1	3	1435	0.002090592
TRAPPC8	3	1435	0.002090592
DIRC2	1	478	0.00209205
FOXD3	1	478	0.00209205
GAS8	1	478	0.00209205
GDF10	1	478	0.00209205
HTR3A	1	478	0.00209205
MDGA2	2	956	0.00209205
NOS1	3	1434	0.00209205
PRAMEF11	1	478	0.00209205
4-Sep	1	478	0.00209205
SLC16A7	1	478	0.00209205
ZHX3	2	956	0.00209205
CHRD	2	955	0.002094241
CLSTN2	2	955	0.002094241
THRAP3	2	955	0.002094241
NCKAP5	4	1909	0.002095338
PLXNB3	4	1909	0.002095338
ETV1	1	477	0.002096436
GCGR	1	477	0.002096436
MYBPH	1	477	0.002096436
PPOX	1	477	0.002096436
SLC2A8	1	477	0.002096436
SMARCAL1	2	954	0.002096436
TRIM72	1	477	0.002096436
ZNF622	1	477	0.002096436
PCNT	7	3336	0.002098321
RAI1	4	1906	0.002098636
SAFB2	2	953	0.002098636
DMKN	1	476	0.00210084
EVX2	1	476	0.00210084
GPKOW	1	476	0.00210084
MEIOC	2	952	0.00210084
NARFL	1	476	0.00210084
RNMT	1	476	0.00210084
TINAG	1	476	0.00210084
TOM1L1	1	476	0.00210084
CNNM1	2	951	0.002103049
ATP10D	3	1426	0.002103787
LYST	8	3801	0.002104709
PCDHA13	2	950	0.002105263
PCDHA3	2	950	0.002105263
PCDHA6	2	950	0.002105263
RTL3	1	475	0.002105263
TUBE1	1	475	0.002105263

UBQLNL	1	475	0.002105263
RIC1	3	1423	0.002108222
CBLC	1	474	0.002109705
CCDC149	1	474	0.002109705
CCDC6	1	474	0.002109705
HADHB	1	474	0.002109705
PRAMEF2	1	474	0.002109705
RNF14	1	474	0.002109705
SAAL1	1	474	0.002109705
PLXNA4	4	1894	0.002111932
ZNF268	2	947	0.002111932
GPR179	5	2367	0.002112379
DST	16	7570	0.002113606
CAMK4	1	473	0.002114165
DUS1L	1	473	0.002114165
RGL4	1	473	0.002114165
SERINC3	1	473	0.002114165
SPTBN1	5	2364	0.002115059
IFIT2	1	472	0.002118644
MBOAT7	1	472	0.002118644
PCDHGC5	2	944	0.002118644
RGS6	1	472	0.002118644
UNC45A	2	944	0.002118644
CDH19	7	3298	0.002122498
DENND2D	1	471	0.002123142
DGKQ	2	942	0.002123142
MLKL	1	471	0.002123142
NCOA7	2	942	0.002123142
ZFYVE19	1	471	0.002123142
ZNF385B	1	471	0.002123142
ZNF106	4	1883	0.00212427
ERBIN	3	1412	0.002124646
FHAD1	3	1412	0.002124646
GABBR2	2	941	0.002125399
PDE2A	2	941	0.002125399
ADAMDEC1	1	470	0.00212766
NR0B1	1	470	0.00212766
ABHD16B	1	469	0.002132196
CDK14	1	469	0.002132196
MYOCD	2	938	0.002132196
PCDHGC4	2	938	0.002132196
PFKFB4	1	469	0.002132196
PNLIPRP2	1	469	0.002132196
RUFY3	1	469	0.002132196
SLC25A25	1	469	0.002132196
UFSP2	1	469	0.002132196
MORC4	2	937	0.002134472

SMCR8	2	937	0.002134472
CHN2	1	468	0.002136752
CHRNA5	1	468	0.002136752
NUP50	1	468	0.002136752
TMEM151A	1	468	0.002136752
ARMC5	2	935	0.002139037
DMAP1	1	467	0.002141328
IGSF21	1	467	0.002141328
KIF27	3	1401	0.002141328
OSBPL6	2	934	0.002141328
PSEN1	1	467	0.002141328
SBF1	4	1868	0.002141328
SLC17A1	1	467	0.002141328
ZIC3	1	467	0.002141328
NLRC5	4	1866	0.002143623
PGR	2	933	0.002143623
ASB18	1	466	0.002145923
CHIT1	1	466	0.002145923
CTDSPL2	1	466	0.002145923
LRRC6	1	466	0.002145923
MPP1	1	466	0.002145923
MTX1	1	466	0.002145923
NIPAL4	1	466	0.002145923
PCDHGA3	2	932	0.002145923
MAL	2	931	0.002148228
TANC1	4	1861	0.002149382
ADRA2A	1	465	0.002150538
CNTRL	5	2325	0.002150538
PNLIP	1	465	0.002150538
RANBP3L	1	465	0.002150538
SRPX2	1	465	0.002150538
STK38	1	465	0.002150538
THEGL	1	465	0.002150538
MYO16	4	1858	0.002152853
ASL	1	464	0.002155172
FRMD8	1	464	0.002155172
LRRC34	1	464	0.002155172
SERPINC1	1	464	0.002155172
NUP155	3	1391	0.002156722
GBA2	2	927	0.002157497
AASS	2	926	0.002159827
DCAF12L1	1	463	0.002159827
DCAF12L2	1	463	0.002159827
GLP1R	1	463	0.002159827
PTPN4	2	926	0.002159827
SIGLEC9	1	463	0.002159827
SUCLA2	1	463	0.002159827

ROCK2	3	1388	0.002161383
NFATC2	2	925	0.002162162
BTAF1	4	1849	0.002163332
EXOC2	2	924	0.002164502
INPP4B	2	924	0.002164502
INTS12	1	462	0.002164502
KREMEN2	1	462	0.002164502
NUDT12	1	462	0.002164502
SEMG1	1	462	0.002164502
SLC38A7	1	462	0.002164502
ALDH1L2	2	923	0.002166847
CCAR2	2	923	0.002166847
NRP1	2	923	0.002166847
SRCAP	7	3230	0.002167183
C9orf43	1	461	0.002169197
CALCRL	1	461	0.002169197
CDS1	1	461	0.002169197
CREB3L3	1	461	0.002169197
F9	1	461	0.002169197
ITPKA	1	461	0.002169197
RNH1	1	461	0.002169197
TGFB1I1	1	461	0.002169197
WWP1	2	922	0.002169197
ZFP2	1	461	0.002169197
EIF3A	3	1382	0.002170767
DCTN4	1	460	0.002173913
FBRS	1	460	0.002173913
IRAK4	1	460	0.002173913
LIPI	1	460	0.002173913
NR1H2	1	460	0.002173913
WRAP73	1	460	0.002173913
CFAP99	1	459	0.002178649
CHN1	1	459	0.002178649
FEZF2	1	459	0.002178649
IL6ST	2	918	0.002178649
IL7R	1	459	0.002178649
OXSM	1	459	0.002178649
SLC46A1	1	459	0.002178649
STPG2	1	459	0.002178649
WDR41	1	459	0.002178649
ZMYM2	3	1377	0.002178649
ARID2	4	1835	0.002179837
PIEZO2	6	2752	0.002180233
CD4	1	458	0.002183406
FAM155A	1	458	0.002183406
FICD	1	458	0.002183406
GALK2	1	458	0.002183406

HTR2C	1	458	0.002183406
NFYC	1	458	0.002183406
SERGEF	1	458	0.002183406
FAM13B	2	915	0.002185792
TMTC3	2	915	0.002185792
CHD2	4	1828	0.002188184
CHKA	1	457	0.002188184
FKBP5	1	457	0.002188184
GLRA1	1	457	0.002188184
LARP1B	2	914	0.002188184
VIPR1	1	457	0.002188184
ZNF140	1	457	0.002188184
AFDN	4	1824	0.002192982
DNMT3A	2	912	0.002192982
GRM4	2	912	0.002192982
PLA1A	1	456	0.002192982
PSMD12	1	456	0.002192982
SLC29A2	1	456	0.002192982
ZNF556	1	456	0.002192982
IGF1R	3	1367	0.002194587
ITIH1	2	911	0.00219539
LIG4	2	911	0.00219539
LTBP2	4	1821	0.002196595
ADGRF1	2	910	0.002197802
DDX47	1	455	0.002197802
EPHX1	1	455	0.002197802
KIAA1147	1	455	0.002197802
KIF20B	4	1820	0.002197802
TNFRSF1A	1	455	0.002197802
RFX7	3	1363	0.002201027
BMP5	1	454	0.002202643
DAPK3	1	454	0.002202643
FBXW2	1	454	0.002202643
GDF9	1	454	0.002202643
GRM8	2	908	0.002202643
HTR3D	1	454	0.002202643
KYAT3	1	454	0.002202643
NCR3LG1	1	454	0.002202643
PCED1A	1	454	0.002202643
SNAP91	2	907	0.002205072
MSH6	3	1360	0.002205882
TNIK	3	1360	0.002205882
C17orf97	1	453	0.002207506
COPB2	2	906	0.002207506
FAM117A	1	453	0.002207506
PPP2R3C	1	453	0.002207506
TUBD1	1	453	0.002207506

STON2	2	905	0.002209945
PLIN4	3	1357	0.002210759
ECT2L	2	904	0.002212389
ETNK1	1	452	0.002212389
FAM222A	1	452	0.002212389
FBXO47	1	452	0.002212389
IDH2	1	452	0.002212389
PARD3	3	1356	0.002212389
TFAP2D	1	452	0.002212389
TLR3	2	904	0.002212389
ZNF598	2	904	0.002212389
ZNF672	1	452	0.002212389
AKAP1	2	903	0.002214839
ZZZ3	2	903	0.002214839
KIF13A	4	1805	0.002216066
AGBL2	2	902	0.002217295
IRF4	1	451	0.002217295
POU3F1	1	451	0.002217295
RUSC1	2	902	0.002217295
TINF2	1	451	0.002217295
TUBG1	1	451	0.002217295
ZNF222	1	451	0.002217295
BECN1	1	450	0.002222222
CCSER1	2	900	0.002222222
LANCL2	1	450	0.002222222
MARS	2	900	0.002222222
SNX4	1	450	0.002222222
SQOR	1	450	0.002222222
ZFP62	2	900	0.002222222
ZNF446	1	450	0.002222222
PRRT4	2	899	0.002224694
C7orf26	1	449	0.002227171
CDCP2	1	449	0.002227171
CMPK2	1	449	0.002227171
RMND1	1	449	0.002227171
AZIN1	1	448	0.002232143
EVI2B	1	448	0.002232143
N4BP1	2	896	0.002232143
AXL	2	894	0.002237136
BAG5	1	447	0.002237136
CSNK1G3	1	447	0.002237136
EXOC1	2	894	0.002237136
FBXO9	1	447	0.002237136
HTR3C	1	447	0.002237136
NR1H3	1	447	0.002237136
PRDM9	2	894	0.002237136
SETX	6	2677	0.002241315

CENPJ	3	1338	0.002242152
CLEC18C	1	446	0.002242152
GPATCH4	1	446	0.002242152
GRWD1	1	446	0.002242152
SAMD7	1	446	0.002242152
SMOC2	1	446	0.002242152
SOX3	1	446	0.002242152
WIPI1	1	446	0.002242152
KDM2B	3	1336	0.002245509
RTTN	5	2226	0.002246181
ALS2CR12	1	445	0.002247191
BCKDHA	1	445	0.002247191
C10orf88	1	445	0.002247191
C12orf66	1	445	0.002247191
DCAF13	1	445	0.002247191
KIF20A	2	890	0.002247191
MGAT1	1	445	0.002247191
MTRF1	1	445	0.002247191
SCLY	1	445	0.002247191
TYRO3	2	890	0.002247191
CACNA1C	5	2221	0.002251238
FADS2	1	444	0.002252252
FOXF2	1	444	0.002252252
MINK1	3	1332	0.002252252
MOCOS	2	888	0.002252252
TPH1	1	444	0.002252252
VPS4B	1	444	0.002252252
ANKRD11	6	2663	0.002253098
CNTNAP2	3	1331	0.002253944
ZFYVE28	2	887	0.002254791
MUC5B	13	5762	0.002256161
CHST9	1	443	0.002257336
CPM	1	443	0.002257336
DPH1	1	443	0.002257336
EFEMP2	1	443	0.002257336
ESYT3	2	886	0.002257336
GALNTL5	1	443	0.002257336
IQCG	1	443	0.002257336
STK24	1	443	0.002257336
UNC13C	5	2214	0.002258356
MAP3K19	3	1328	0.002259036
DIS3L2	2	885	0.002259887
CCDC171	3	1326	0.002262443
MEIOB	1	442	0.002262443
TRIM14	1	442	0.002262443
ZMYM6	3	1325	0.002264151
LTN1	4	1766	0.002265006

CCDC91	1	441	0.002267574
CDH1	2	882	0.002267574
COL26A1	1	441	0.002267574
HTR3B	1	441	0.002267574
LRRC17	1	441	0.002267574
SPP1	1	441	0.002267574
TMTC1	2	882	0.002267574
UBXN6	1	441	0.002267574
FAAP100	2	881	0.002270148
PPP6R1	2	881	0.002270148
ABCB11	3	1321	0.002271007
ENPP6	1	440	0.002272727
GABRP	1	440	0.002272727
HTR6	1	440	0.002272727
SNX31	1	440	0.002272727
MANBA	2	879	0.002275313
SP100	2	879	0.002275313
ABHD8	1	439	0.002277904
OAT	1	439	0.002277904
PKD2	2	878	0.002277904
PNMA8A	1	439	0.002277904
TERF1	1	439	0.002277904
GRM6	2	877	0.002280502
MED16	2	877	0.002280502
CCDC78	1	438	0.002283105
CLN3	1	438	0.002283105
VIPR2	1	438	0.002283105
ZNF641	1	438	0.002283105
ANK3	10	4377	0.00228467
AGAP3	2	875	0.002285714
ENPP3	2	875	0.002285714
KLHL29	2	875	0.002285714
MCM10	2	875	0.002285714
PDE5A	2	875	0.002285714
BYSL	1	437	0.00228833
E2F2	1	437	0.00228833
IPCEF1	1	437	0.00228833
OXR1	2	874	0.00228833
P3H4	1	437	0.00228833
TFAP2A	1	437	0.00228833
ZNF521	3	1311	0.00228833
PPP4R4	2	873	0.002290951
SOBP	2	873	0.002290951
VLDLR	2	873	0.002290951
GCM1	1	436	0.002293578
KCNQ2	2	872	0.002293578
NWD2	4	1742	0.002296211

TRPV4	2	871	0.002296211
BPTF	7	3046	0.002298096
CADM2	1	435	0.002298851
DLG2	2	870	0.002298851
HYAL1	1	435	0.002298851
MBOAT4	1	435	0.002298851
NXN	1	435	0.002298851
PSG6	1	435	0.002298851
TMEM130	1	435	0.002298851
ZBTB25	1	435	0.002298851
ZNF629	2	869	0.002301496
AAMP	1	434	0.002304147
CMAS	1	434	0.002304147
ENO2	1	434	0.002304147
ENO3	1	434	0.002304147
PDCD6IP	2	868	0.002304147
PLIN3	1	434	0.002304147
6-Sep	1	434	0.002304147
CCDC178	2	867	0.002306805
E2F8	2	867	0.002306805
VSIG10L	2	867	0.002306805
ESRRB	1	433	0.002309469
FAM98B	1	433	0.002309469
NFRKB	3	1299	0.002309469
SFMBT1	2	866	0.002309469
SMPD4	2	866	0.002309469
TRPM3	4	1732	0.002309469
INSRR	3	1297	0.00231303
CCNA2	1	432	0.002314815
DNM1	2	864	0.002314815
FOXB2	1	432	0.002314815
GRAMD2B	1	432	0.002314815
NPTX1	1	432	0.002314815
PCED1B	1	432	0.002314815
RNF44	1	432	0.002314815
14-Sep	1	432	0.002314815
TXNDC5	1	432	0.002314815
WDR18	1	432	0.002314815
ZNF99	2	864	0.002314815
FSIP2	16	6907	0.002316491
DPP9	2	863	0.002317497
AXIN1	2	862	0.002320186
GAL3ST3	1	431	0.002320186
NPTX2	1	431	0.002320186
PMS2	2	862	0.002320186
STRADA	1	431	0.002320186
SYT11	1	431	0.002320186

TRPC7	2	862	0.002320186
AMER3	2	861	0.00232288
ATXN7L1	2	861	0.00232288
BSDC1	1	430	0.002325581
GCNT7	1	430	0.002325581
KRT18	1	430	0.002325581
PPM1N	1	430	0.002325581
RBM34	1	430	0.002325581
ZNF713	1	430	0.002325581
LRP12	2	859	0.002328289
FCRL1	1	429	0.002331002
GLA	1	429	0.002331002
IFT57	1	429	0.002331002
KCNB1	2	858	0.002331002
MAGEA11	1	429	0.002331002
NCAM1	2	858	0.002331002
SRFBP1	1	429	0.002331002
TTC7A	2	858	0.002331002
ZNF275	1	429	0.002331002
ZXDC	2	858	0.002331002
RPGRIP1	3	1286	0.002332815
TTK	2	857	0.002333722
COL20A1	3	1284	0.002336449
ELK1	1	428	0.002336449
HNRNPUL1	2	856	0.002336449
ISLR	1	428	0.002336449
KCTD16	1	428	0.002336449
LRRC42	1	428	0.002336449
NEU3	1	428	0.002336449
PALD1	2	856	0.002336449
RHBDF2	2	856	0.002336449
RNF128	1	428	0.002336449
ZNF423	3	1284	0.002336449
DNAAF5	2	855	0.002339181
RHBDF1	2	855	0.002339181
ZNF814	2	855	0.002339181
SPTBN4	6	2564	0.002340094
ATP6V1C2	1	427	0.00234192
DDB2	1	427	0.00234192
IRF3	1	427	0.00234192
KCNJ2	1	427	0.00234192
PLXNB1	5	2135	0.00234192
RPL4	1	427	0.00234192
VDR	1	427	0.00234192
C1orf112	2	853	0.002344666
ABCA3	4	1704	0.002347418
ACPT	1	426	0.002347418

DCTN1	3	1278	0.002347418
EGLN1	1	426	0.002347418
INHBA	1	426	0.002347418
IP6K2	1	426	0.002347418
IVD	1	426	0.002347418
KCTD18	1	426	0.002347418
NMUR1	1	426	0.002347418
PIWIL4	2	852	0.002347418
PSG9	1	426	0.002347418
RAD9B	1	426	0.002347418
SLC16A13	1	426	0.002347418
STK25	1	426	0.002347418
ZC3H15	1	426	0.002347418
ZNF562	1	426	0.002347418
DDX31	2	851	0.002350176
TGFBR3	2	851	0.002350176
CCDC180	4	1701	0.002351558
CACNG6	1	425	0.002352941
CAVIN2	1	425	0.002352941
F2R	1	425	0.002352941
HCRTR1	1	425	0.002352941
PLEKHA2	1	425	0.002352941
RASA2	2	850	0.002352941
SCRN2	1	425	0.002352941
PLA2G4F	2	849	0.002355713
RASGRF1	3	1273	0.002356638
CHST8	1	424	0.002358491
DIAPH1	3	1272	0.002358491
KRT20	1	424	0.002358491
SBK1	1	424	0.002358491
SERPINE3	1	424	0.002358491
SLC22A18	1	424	0.002358491
SLC35A5	1	424	0.002358491
STAMBP	1	424	0.002358491
PLEKHG4B	3	1271	0.002360346
MAP3K11	2	847	0.002361275
ACP2	1	423	0.002364066
AP1M1	1	423	0.002364066
ARHGAP12	2	846	0.002364066
CRAMP1	3	1269	0.002364066
FAM43A	1	423	0.002364066
GHRHR	1	423	0.002364066
GPR83	1	423	0.002364066
PIGM	1	423	0.002364066
SERPINA3	1	423	0.002364066
SH2D5	1	423	0.002364066
SMPD2	1	423	0.002364066

TTLL1	1	423	0.002364066
ZNF557	1	423	0.002364066
VAV1	2	845	0.002366864
HTR1A	1	422	0.002369668
JAKMIP3	2	844	0.002369668
PAX6	1	422	0.002369668
SERPINA11	1	422	0.002369668
STYK1	1	422	0.002369668
SYT1	1	422	0.002369668
ZBTB42	1	422	0.002369668
ADAMTS7	4	1686	0.002372479
ACADM	1	421	0.002375297
BEND5	1	421	0.002375297
C7orf25	1	421	0.002375297
OSBPL7	2	842	0.002375297
PIP4K2C	1	421	0.002375297
RCC1	1	421	0.002375297
SYT15	1	421	0.002375297
TRMU	1	421	0.002375297
ZNF584	1	421	0.002375297
SLITRK6	2	841	0.002378121
ADCY5	3	1261	0.002379064
COBL	3	1261	0.002379064
HMBOX1	1	420	0.002380952
HNRNPDL	1	420	0.002380952
L3MBTL1	2	840	0.002380952
LANCL3	1	420	0.002380952
LZTR1	2	840	0.002380952
NAPA	1	420	0.002380952
NAPSA	1	420	0.002380952
NFIB	1	420	0.002380952
PELI2	1	420	0.002380952
RTN4RL2	1	420	0.002380952
UXS1	1	420	0.002380952
BZW1	1	419	0.002386635
CCDC85C	1	419	0.002386635
CPA1	1	419	0.002386635
GPR63	1	419	0.002386635
IKBKG	1	419	0.002386635
PSG9	1	419	0.002386635
PYGO1	1	419	0.002386635
RASSF8	1	419	0.002386635
SEMA4G	2	838	0.002386635
SYT2	1	419	0.002386635
TACC3	2	838	0.002386635
UHMK1	1	419	0.002386635
VEGFC	1	419	0.002386635

MOGS	2	837	0.002389486
AP3M2	1	418	0.002392344
ARRB1	1	418	0.002392344
BAAT	1	418	0.002392344
KCNJ16	1	418	0.002392344
PSMC4	1	418	0.002392344
RASSF5	1	418	0.002392344
SERPINH1	1	418	0.002392344
SPRED2	1	418	0.002392344
STRADB	1	418	0.002392344
TMTC2	2	836	0.002392344
ZNF546	2	836	0.002392344
ZNF566	1	418	0.002392344
PHLDB2	3	1253	0.002394254
BCL11A	2	835	0.00239521
CMTR1	2	835	0.00239521
ANKS4B	1	417	0.002398082
CALR	1	417	0.002398082
CNGB1	3	1251	0.002398082
FDFT1	1	417	0.002398082
LAMP1	1	417	0.002398082
PGK2	1	417	0.002398082
PREB	1	417	0.002398082
SERPINA11	1	417	0.002398082
SLC9A3	2	834	0.002398082
ZNF232	1	417	0.002398082
RAPH1	3	1250	0.0024
DCHS2	7	2916	0.002400549
SEMA4C	2	833	0.00240096
INF2	3	1249	0.002401922
ADAM23	2	832	0.002403846
IPMK	1	416	0.002403846
PIP4K2B	1	416	0.002403846
PSTPIP1	1	416	0.002403846
RCSD1	1	416	0.002403846
RIPK4	2	832	0.002403846
TMPRSS11B	1	416	0.002403846
ATG2B	5	2078	0.00240616
RILP	2	831	0.002406739
DAW1	1	415	0.002409639
RUNX3	1	415	0.002409639
ZPLD1	1	415	0.002409639
ODF2	2	829	0.002412545
PDE8A	2	829	0.002412545
C12orf50	1	414	0.002415459
ELFN1	2	828	0.002415459
IDH1	1	414	0.002415459

IRS1	3	1242	0.002415459
LRR1	1	414	0.002415459
NR2F2	1	414	0.002415459
SLMAP	2	828	0.002415459
STK17A	1	414	0.002415459
USP6NL	2	828	0.002415459
ZNF438	2	828	0.002415459
TTN	83	34350	0.002416303
ADRB2	1	413	0.002421308
ELP2	2	826	0.002421308
FAM171B	2	826	0.002421308
FAM187A	1	413	0.002421308
GOT1	1	413	0.002421308
HIF1A	2	826	0.002421308
PRSS35	1	413	0.002421308
TMEM184A	1	413	0.002421308
ZDHHC6	1	413	0.002421308
WIZ	4	1651	0.002422774
ARID3C	1	412	0.002427184
ART4	1	412	0.002427184
DDX20	2	824	0.002427184
DOC2B	1	412	0.002427184
FKBP8	1	412	0.002427184
INPP5A	1	412	0.002427184
NPAS2	2	824	0.002427184
ZDHHC11	1	412	0.002427184
ZNF821	1	412	0.002427184
SMARCA4	4	1647	0.002428658
ATMIN	2	823	0.002430134
FAM83G	2	823	0.002430134
USP16	2	823	0.002430134
PLCB3	3	1234	0.002431118
DPEP1	1	411	0.00243309
EIF4A3	1	411	0.00243309
FER	2	822	0.00243309
GSDMB	1	411	0.00243309
GTF3C4	2	822	0.00243309
IHH	1	411	0.00243309
NAGA	1	411	0.00243309
SMARCE1	1	411	0.00243309
SUSD2	2	822	0.00243309
CEP95	2	821	0.002436054
WDR87	7	2873	0.002436478
CFH	3	1231	0.002437043
EHBP1	3	1231	0.002437043
MAP4K2	2	820	0.002439024
MECOM	3	1230	0.002439024

PAFAH1B1	1	410	0.002439024
RMDN2	1	410	0.002439024
SERPINI1	1	410	0.002439024
TDG	1	410	0.002439024
TFDP1	1	410	0.002439024
WDFY1	1	410	0.002439024
POM121C	3	1229	0.002441009
C9orf3	2	819	0.002442002
MYLK3	2	819	0.002442002
KIF21B	4	1637	0.002443494
KMT2C	12	4911	0.002443494
AMDHD2	1	409	0.002444988
CDAN1	3	1227	0.002444988
PTPN23	4	1636	0.002444988
ZDHHC23	1	409	0.002444988
ATP13A3	3	1226	0.002446982
NHSL2	3	1225	0.00244898
HNF4G	1	408	0.00245098
NCEH1	1	408	0.00245098
SERBP1	1	408	0.00245098
SSB	1	408	0.00245098
KCTD3	2	815	0.002453988
ARHGAP26	2	814	0.002457002
CEP250	6	2442	0.002457002
MNDA	1	407	0.002457002
TACR1	1	407	0.002457002
TMEM184B	1	407	0.002457002
ZFP1	1	407	0.002457002
ZNF365	1	407	0.002457002
MAP6	2	813	0.002460025
RTEL1	3	1219	0.002461034
BHMT	1	406	0.002463054
CCBE1	1	406	0.002463054
GLMP	1	406	0.002463054
HERPUD2	1	406	0.002463054
KLHDC2	1	406	0.002463054
OLFML3	1	406	0.002463054
PSMC5	1	406	0.002463054
PYGO2	1	406	0.002463054
ZSCAN31	1	406	0.002463054
RYR3	12	4870	0.002464066
DEPDC1	2	811	0.002466091
TRIL	2	811	0.002466091
SPOCD1	3	1216	0.002467105
CC2D2A	4	1620	0.002469136
FCHO2	2	810	0.002469136
HIPK3	3	1215	0.002469136

NRP1	2	810	0.002469136
SERPINB12	1	405	0.002469136
UBAC1	1	405	0.002469136
CCNE2	1	404	0.002475248
KCNAB3	1	404	0.002475248
KIAA0930	1	404	0.002475248
SNX5	1	404	0.002475248
SYNE4	1	404	0.002475248
UBA5	1	404	0.002475248
WSB2	1	404	0.002475248
EPC2	2	807	0.002478315
SPON1	2	807	0.002478315
LRP6	4	1613	0.002479851
AMT	1	403	0.00248139
GPRC5B	1	403	0.00248139
JMJD6	1	403	0.00248139
PCBP4	1	403	0.00248139
QTRT1	1	403	0.00248139
RILPL1	1	403	0.00248139
SNX32	1	403	0.00248139
TMEM246	1	403	0.00248139
ZCCHC3	1	403	0.00248139
DZIP3	3	1208	0.002483444
B3GNT9	1	402	0.002487562
MAP3K4	4	1608	0.002487562
11-Mar	1	402	0.002487562
OPN3	1	402	0.002487562
PTGER1	1	402	0.002487562
SMYD4	2	804	0.002487562
BRPF3	3	1205	0.002489627
ARHGEF7	2	803	0.00249066
HECW1	4	1606	0.00249066
ZBTB17	2	803	0.00249066
ZXDB	2	803	0.00249066
B3GNT7	1	401	0.002493766
CRTAP	1	401	0.002493766
FGFR4	2	802	0.002493766
PTPA	2	802	0.002493766
PTPRA	2	802	0.002493766
TARSL2	2	802	0.002493766
USP32	4	1604	0.002493766
PCDHB8	2	801	0.002496879
C1orf141	1	400	0.0025
DOC2A	1	400	0.0025
DRD3	1	400	0.0025
KRT19	1	400	0.0025
MAP3K20	2	800	0.0025

OAS1	1	400	0.0025
OPRM1	1	400	0.0025
RRAGD	1	400	0.0025
SART1	2	800	0.0025
SPN	1	400	0.0025
TBC1D13	1	400	0.0025
TFG	1	400	0.0025
WDFY2	1	400	0.0025
EIF4G1	4	1599	0.002501563
TTLL4	3	1199	0.002502085
CDH8	2	799	0.002503129
SHROOM3	5	1996	0.00250501
BRS3	1	399	0.002506266
CIPC	1	399	0.002506266
ELOA	2	798	0.002506266
GPR137B	1	399	0.002506266
INPP1	1	399	0.002506266
MYPOP	1	399	0.002506266
SGPP2	1	399	0.002506266
SMC2	3	1197	0.002506266
USP10	2	798	0.002506266
ATP13A4	3	1196	0.002508361
ADGRG7	2	797	0.00250941
CASD1	2	797	0.00250941
ATG4A	1	398	0.002512563
CATSPER3	1	398	0.002512563
CCNB2	1	398	0.002512563
CYTH1	1	398	0.002512563
GAL3ST2	1	398	0.002512563
KISS1R	1	398	0.002512563
NUGGC	2	796	0.002512563
OTUD3	1	398	0.002512563
POLR3D	1	398	0.002512563
SERPINE2	1	398	0.002512563
SP5	1	398	0.002512563
ST8SIA6	1	398	0.002512563
STOML1	1	398	0.002512563
TBX1	1	398	0.002512563
TRHR	1	398	0.002512563
LAMC2	3	1193	0.002514669
SCN9A	5	1988	0.002515091
NAV3	6	2385	0.002515723
AGAP2	3	1192	0.002516779
PKP4	3	1192	0.002516779
ACAA2	1	397	0.002518892
ELL3	1	397	0.002518892
LHX3	1	397	0.002518892

PLEKHG4	3	1191	0.002518892
SEC14L6	1	397	0.002518892
SERPINB10	1	397	0.002518892
TOR3A	1	397	0.002518892
TJP2	3	1190	0.002521008
UHRF1	2	793	0.002522068
DAB2IP	3	1189	0.002523129
EIF4G3	4	1585	0.002523659
CACNA1G	6	2377	0.00252419
ARID5B	3	1188	0.002525253
CTSE	1	396	0.002525253
DDI1	1	396	0.002525253
ERCC8	1	396	0.002525253
FBXO38	3	1188	0.002525253
GPR84	1	396	0.002525253
MAEA	1	396	0.002525253
MGAT5B	2	792	0.002525253
MVK	1	396	0.002525253
OLA1	1	396	0.002525253
RTN4IP1	1	396	0.002525253
SIGLEC14	1	396	0.002525253
SNIP1	1	396	0.002525253
SS18L1	1	396	0.002525253
ZNF606	2	792	0.002525253
ANK2	10	3957	0.002527167
EYS	8	3165	0.002527646
FAM47A	2	791	0.002528445
SLC26A9	2	791	0.002528445
ZMYND8	3	1186	0.002529511
CABS1	1	395	0.002531646
CBWD1	1	395	0.002531646
GNB5	1	395	0.002531646
GTF2H2C	1	395	0.002531646
KIF9	2	790	0.002531646
MRPS31	1	395	0.002531646
PTGDR2	1	395	0.002531646
TEX44	1	395	0.002531646
TSC22D4	1	395	0.002531646
CDH10	2	788	0.002538071
CERS4	1	394	0.002538071
GORAB	1	394	0.002538071
JAML	1	394	0.002538071
MARK2	2	788	0.002538071
NDRG1	1	394	0.002538071
PHAX	1	394	0.002538071
SAPCD2	1	394	0.002538071
SPATA31D1	4	1576	0.002538071

ITGA7	3	1181	0.00254022
ATG4B	1	393	0.002544529
CCNF	2	786	0.002544529
FAM47E	1	393	0.002544529
ITGAE	3	1179	0.002544529
KCNK4	1	393	0.002544529
KIN	1	393	0.002544529
PROKR1	1	393	0.002544529
RMND5B	1	393	0.002544529
SEC31B	3	1179	0.002544529
XXYLT1	1	393	0.002544529
ZCCHC2	3	1178	0.002546689
AP1G2	2	785	0.002547771
COG4	2	785	0.002547771
PHTF2	2	785	0.002547771
SEMA3F	2	785	0.002547771
SLC9C1	3	1177	0.002548853
FAM53C	1	392	0.00255102
GALK1	1	392	0.00255102
PLXNC1	4	1568	0.00255102
SPATA6L	1	392	0.00255102
SPOPL	1	392	0.00255102
FYB	2	783	0.002554278
RNF43	2	783	0.002554278
ALLC	1	391	0.002557545
BDKRB2	1	391	0.002557545
PAX5	1	391	0.002557545
RBMX	1	391	0.002557545
RMND5A	1	391	0.002557545
TRDMT1	1	391	0.002557545
FBP2	3	1172	0.002559727
MEFV	2	781	0.002560819
ACO2	2	780	0.002564103
CHI3L2	1	390	0.002564103
GFOD1	1	390	0.002564103
HRH4	1	390	0.002564103
HS3ST3B1	1	390	0.002564103
PPP1R21	2	780	0.002564103
RBM33	3	1170	0.002564103
SLC26A4	2	780	0.002564103
THBS1	3	1170	0.002564103
MYCBP2	12	4678	0.002565199
SORCS1	3	1168	0.002568493
FLNC	7	2725	0.002568807
PCYT2	1	389	0.002570694
SCAP	1	389	0.002570694
SH2D2A	1	389	0.002570694

UBE3D	1	389	0.002570694
KIAA1217	5	1943	0.00257334
CAGE1	2	777	0.002574003
MTMR10	2	777	0.002574003
ARR3	1	388	0.00257732
CD1E	1	388	0.00257732
HOXA13	1	388	0.00257732
HTR4	1	388	0.00257732
LMNTD1	1	388	0.00257732
MIIP	1	388	0.00257732
MYLK4	1	388	0.00257732
PDHA2	1	388	0.00257732
PGC	1	388	0.00257732
POFUT1	1	388	0.00257732
PRR5	1	388	0.00257732
SOX7	1	388	0.00257732
SSTR4	1	388	0.00257732
MYH4	5	1939	0.002578649
RYR1	13	5038	0.002580389
ADAM23	2	775	0.002580645
ADAM28	2	775	0.002580645
KAZN	2	775	0.002580645
SEMA3E	2	775	0.002580645
TRIO	8	3097	0.002583145
BBOX1	1	387	0.002583979
GTPBP10	1	387	0.002583979
HSD17B2	1	387	0.002583979
PHKG1	1	387	0.002583979
REXO5	2	774	0.002583979
SIRPB2	1	387	0.002583979
CPT1A	2	773	0.002587322
DGCR8	2	773	0.002587322
TLE4	2	773	0.002587322
PCDH17	3	1159	0.002588438
PRUNE2	8	3088	0.002590674
ASXL1	4	1541	0.002595717
C2orf69	1	385	0.002597403
CIART	1	385	0.002597403
CTBS	1	385	0.002597403
GDPGP1	1	385	0.002597403
GFOD2	1	385	0.002597403
MR1	1	385	0.002597403
NSMCE4A	1	385	0.002597403
TLE1	2	770	0.002597403
NOS2	3	1153	0.002601908
CCR10	1	384	0.002604167
KCNK18	1	384	0.002604167

KIF25	1	384	0.002604167
NPY1R	1	384	0.002604167
NSUN4	1	384	0.002604167
SPHK1	1	384	0.002604167
KIAA0586	4	1533	0.002609263
AGL	4	1532	0.002610966
DCLK2	2	766	0.002610966
DLK1	1	383	0.002610966
FGD4	2	766	0.002610966
KCNU1	3	1149	0.002610966
NOX5	2	765	0.002614379
SNRK	2	765	0.002614379
TRPV6	2	765	0.002614379
ATP9B	3	1147	0.002615519
DMBX1	1	382	0.002617801
FETUB	1	382	0.002617801
TICRR	5	1910	0.002617801
TSHR	2	764	0.002617801
APPL2	2	763	0.002621232
PLPPR4	2	763	0.002621232
SATB1	2	763	0.002621232
CEP85	2	762	0.002624672
LDHAL6B	1	381	0.002624672
PRKAR1B	1	381	0.002624672
SELENOP	1	381	0.002624672
SFTPB	1	381	0.002624672
ARHGEF11	4	1522	0.002628121
THAP12	2	761	0.002628121
ADH4	1	380	0.002631579
ANKRD63	1	380	0.002631579
DMP1	2	760	0.002631579
DPF1	1	380	0.002631579
ERCC2	2	760	0.002631579
FAP	2	760	0.002631579
FEN1	1	380	0.002631579
GPR132	1	380	0.002631579
MEGF10	3	1140	0.002631579
NPRL2	1	380	0.002631579
SASH3	1	380	0.002631579
SEC63	2	760	0.002631579
ST8SIA3	1	380	0.002631579
TMEM229A	1	380	0.002631579
UBL7	1	380	0.002631579
RASAL2	3	1139	0.002633889
SLC26A6	2	759	0.002635046
ANHX	1	379	0.002638522
SERPINB1	1	379	0.002638522

SUN5	1	379	0.002638522
TMEM173	1	379	0.002638522
WIF1	1	379	0.002638522
MFN2	2	757	0.002642008
FILIP1L	3	1135	0.002643172
B3GNT5	1	378	0.002645503
CCR7	1	378	0.002645503
DIEXF	2	756	0.002645503
DPF3	1	378	0.002645503
HNRNPA3	1	378	0.002645503
LOXL4	2	756	0.002645503
OVCH1	3	1134	0.002645503
TIMD4	1	378	0.002645503
ZNF608	4	1512	0.002645503
ATR	7	2644	0.002647504
SH3PXD2A	3	1133	0.002647838
ANAPC5	2	755	0.002649007
MTSS1	2	755	0.002649007
CCDC113	1	377	0.00265252
DUSP11	1	377	0.00265252
GNA13	1	377	0.00265252
PTPDC1	2	754	0.00265252
RNF215	1	377	0.00265252
TPST2	1	377	0.00265252
ZKSCAN7	2	754	0.00265252
ABCA1	6	2261	0.002653693
LATS1	3	1130	0.002654867
FRYL	8	3013	0.002655161
TMEM131	5	1883	0.002655337
ELOA2	2	753	0.002656042
LOXL3	2	753	0.002656042
PCSK1	2	753	0.002656042
ACTBL2	1	376	0.002659574
C12orf10	1	376	0.002659574
RCCD1	1	376	0.002659574
RNF133	1	376	0.002659574
SP6	1	376	0.002659574
TRABD	1	376	0.002659574
WDR86	1	376	0.002659574
HMCN1	15	5635	0.002661934
ARHGAP5	4	1502	0.002663116
NAV1	5	1877	0.002663825
ADH1B	1	375	0.002666667
AS3MT	1	375	0.002666667
COL28A1	3	1125	0.002666667
CPS1	4	1500	0.002666667
DEK	1	375	0.002666667

GPER1	1	375	0.002666667
MSTN	1	375	0.002666667
NAP1L4	1	375	0.002666667
ST3GAL3	1	375	0.002666667
UBE2Q2	1	375	0.002666667
EFHC2	2	749	0.002670227
MAP7	2	749	0.002670227
RFX3	2	749	0.002670227
SGSM3	2	749	0.002670227
FANCF	1	374	0.002673797
FCGR1A	1	374	0.002673797
GNA15	1	374	0.002673797
GPN1	1	374	0.002673797
JPH3	2	748	0.002673797
PBX4	1	374	0.002673797
PPP1R3A	3	1122	0.002673797
ST6GALNAC2	1	374	0.002673797
MTF1	3	1121	0.002676182
MYT1	3	1121	0.002676182
WDR6	3	1121	0.002676182
BIRC6	13	4857	0.002676549
FAM83C	2	747	0.002677376
HNRNPUL2	2	747	0.002677376
MED25	2	747	0.002677376
MTSS1L	2	747	0.002677376
PKP1	2	747	0.002677376
SUSD1	2	747	0.002677376
TPX2	2	747	0.002677376
WDR91	2	747	0.002677376
ARFIP1	1	373	0.002680965
CCDC34	1	373	0.002680965
CEP41	1	373	0.002680965
FAM129B	2	746	0.002680965
GPR173	1	373	0.002680965
LIPT1	1	373	0.002680965
MAGEC2	1	373	0.002680965
NSDHL	1	373	0.002680965
PRG3	1	373	0.002680965
RASSF7	1	373	0.002680965
CUL2	2	745	0.002684564
B3GNT3	1	372	0.002688172
B4GALT2	1	372	0.002688172
GMDS	1	372	0.002688172
H2AFY2	1	372	0.002688172
PARVA	1	372	0.002688172
PCSK5	5	1860	0.002688172
SELL	1	372	0.002688172

USP18	1	372	0.002688172
POU2F1	2	743	0.00269179
PRRC2B	6	2229	0.00269179
RET	3	1114	0.002692998
ELF3	1	371	0.002695418
HPDL	1	371	0.002695418
NKX2-1	1	371	0.002695418
PCBP3	1	371	0.002695418
SCYL3	2	742	0.002695418
SV2A	2	742	0.002695418
ZNF700	2	742	0.002695418
ZNF707	1	371	0.002695418
SENP6	3	1112	0.002697842
ZBTB20	2	741	0.002699055
ABCD2	2	740	0.002702703
ARPC1A	1	370	0.002702703
CABP1	1	370	0.002702703
FCHSD2	2	740	0.002702703
KRTAP10-7	1	370	0.002702703
LRRC19	1	370	0.002702703
OPRL1	1	370	0.002702703
PDGFD	1	370	0.002702703
RASEF	2	740	0.002702703
PXDN	4	1479	0.00270453
SIX5	2	739	0.00270636
SUZ12	2	739	0.00270636
MYO1E	3	1108	0.002707581
NAT16	1	369	0.002710027
POLD1	3	1107	0.002710027
PRPSAP2	1	369	0.002710027
SVIL	6	2214	0.002710027
PSME4	5	1843	0.002712968
PLOD2	2	737	0.002713704
CELSR3	9	3312	0.002717391
EXOG	1	368	0.002717391
FAM81A	1	368	0.002717391
HAUS7	1	368	0.002717391
KRIT1	2	736	0.002717391
OSBPL9	2	736	0.002717391
RAE1	1	368	0.002717391
SNAPC1	1	368	0.002717391
ADAM2	2	735	0.002721088
DGKA	2	735	0.002721088
TRAPPC12	2	735	0.002721088
WDR72	3	1102	0.002722323
SCN4A	5	1836	0.002723312
ACLY	3	1101	0.002724796

CCDC150	3	1101	0.002724796
CTNNAL1	2	734	0.002724796
DMRTC2	1	367	0.002724796
FCRL3	2	734	0.002724796
MAPK12	1	367	0.002724796
PCYT1A	1	367	0.002724796
RBM48	1	367	0.002724796
UROD	1	367	0.002724796
WISP1	1	367	0.002724796
SORBS2	3	1100	0.002727273
TMEM132D	3	1099	0.002729754
ATP7B	4	1465	0.002730375
AASDH	3	1098	0.00273224
ABI3	1	366	0.00273224
ACTL8	1	366	0.00273224
GHSR	1	366	0.00273224
HTR1F	1	366	0.00273224
INHA	1	366	0.00273224
LEFTY1	1	366	0.00273224
NPHP1	2	732	0.00273224
PPP1R9A	3	1098	0.00273224
RTCA	1	366	0.00273224
SETD7	1	366	0.00273224
SMS	1	366	0.00273224
TDRD7	3	1098	0.00273224
XRCC5	2	732	0.00273224
KCNN3	2	731	0.002735978
ZNF615	2	731	0.002735978
SI	5	1827	0.002736727
CXADR	1	365	0.002739726
FCGRT	1	365	0.002739726
PCBP2	1	365	0.002739726
SH3GLB1	1	365	0.002739726
SLC35G1	1	365	0.002739726
SUGT1	1	365	0.002739726
WNT16	1	365	0.002739726
ZMYND12	1	365	0.002739726
TANGO6	3	1094	0.00274223
ATAD2B	4	1458	0.002743484
TRDN	2	729	0.002743484
STAB2	7	2551	0.002744022
ITGB4	5	1822	0.002744237
FRAS1	11	4008	0.002744511
MLLT6	3	1093	0.002744739
FIBP	1	364	0.002747253
LNX1	2	728	0.002747253
PCCA	2	728	0.002747253

PDLIM3	1	364	0.002747253
VPS72	1	364	0.002747253
ZDHHC9	1	364	0.002747253
TUBGCP6	5	1819	0.002748763
ANKRD6	2	727	0.002751032
ELMO1	2	727	0.002751032
NDUFS1	2	727	0.002751032
SV2C	2	727	0.002751032
ADA	1	363	0.002754821
ADD2	2	726	0.002754821
BHMT2	1	363	0.002754821
C20orf96	1	363	0.002754821
CCNT1	2	726	0.002754821
GBX1	1	363	0.002754821
GPR78	1	363	0.002754821
LHX6	1	363	0.002754821
RFC4	1	363	0.002754821
RFFL	1	363	0.002754821
SPATA22	1	363	0.002754821
TECRL	1	363	0.002754821
MED12	6	2177	0.002756086
FANCD2	4	1451	0.00275672
LATS2	3	1088	0.002757353
ZSCAN10	2	725	0.002758621
GOLGB1	9	3259	0.002761583
ACSBG1	2	724	0.002762431
CCR10	1	362	0.002762431
GPR4	1	362	0.002762431
HMMR	2	724	0.002762431
MAB21L3	1	362	0.002762431
MTNR1B	1	362	0.002762431
DCC	4	1447	0.00276434
TSC2	5	1807	0.002767017
HDAC4	3	1084	0.002767528
ADAM21	2	722	0.002770083
ATAD1	1	361	0.002770083
B3GNTL1	1	361	0.002770083
CAAP1	1	361	0.002770083
CEP131	3	1083	0.002770083
FUT3	1	361	0.002770083
GPR183	1	361	0.002770083
GPR25	1	361	0.002770083
GPR52	1	361	0.002770083
HMBS	1	361	0.002770083
LIMCH1	3	1083	0.002770083
PCMTD2	1	361	0.002770083
SPATA17	1	361	0.002770083

TTC23L	1	361	0.002770083
SETD5	4	1442	0.002773925
PTPRT	4	1441	0.00277585
CCDC86	1	360	0.002777778
CDK10	1	360	0.002777778
FAM102B	1	360	0.002777778
GPR15	1	360	0.002777778
LETMD1	1	360	0.002777778
NIPA2	1	360	0.002777778
NRG3	2	720	0.002777778
PTPN7	1	360	0.002777778
TEFM	1	360	0.002777778
TGM5	2	720	0.002777778
WARS2	1	360	0.002777778
AKNA	4	1439	0.002779708
SLC4A4	3	1079	0.002780352
ZRANB3	3	1079	0.002780352
CLIP1	4	1438	0.002781641
MROH6	2	719	0.002781641
CC2D2B	4	1437	0.002783577
MYOM3	4	1437	0.002783577
CHAD	1	359	0.002785515
FCRL1	1	359	0.002785515
FUT6	1	359	0.002785515
ISL2	1	359	0.002785515
KLF8	1	359	0.002785515
MCUR1	1	359	0.002785515
PDHB	1	359	0.002785515
PLPPR3	2	718	0.002785515
SLC25A39	1	359	0.002785515
TMOD1	1	359	0.002785515
USP28	3	1077	0.002785515
FASN	7	2511	0.002787734
FAM214A	3	1076	0.002788104
ARNT2	2	717	0.0027894
DQX1	2	717	0.0027894
RSPH6A	2	717	0.0027894
POTEI	3	1075	0.002790698
C19orf67	1	358	0.002793296
DNAJC18	1	358	0.002793296
GJA5	1	358	0.002793296
GPR20	1	358	0.002793296
LRRN1	2	716	0.002793296
12-Sep	1	358	0.002793296
ZNF391	1	358	0.002793296
MX2	2	715	0.002797203
KIAA0319	3	1072	0.002798507

ATP1B4	1	357	0.00280112
G6PC	1	357	0.00280112
HUNK	2	714	0.00280112
PCMTD1	1	357	0.00280112
SLC12A8	2	714	0.00280112
BSN	11	3926	0.002801834
KIAA0355	3	1070	0.002803738
CDH13	2	713	0.002805049
POLH	2	713	0.002805049
CSMD1	10	3564	0.002805836
PKD1L1	8	2849	0.002808003
FADS6	1	356	0.002808989
LPO	2	712	0.002808989
MLLT10	3	1068	0.002808989
RIPOR2	3	1068	0.002808989
SERTAD4	1	356	0.002808989
SLCO1C1	2	712	0.002808989
TAB3	2	712	0.002808989
TMC4	2	712	0.002808989
ZCWPW2	1	356	0.002808989
SOGA1	4	1423	0.002810963
ZMIZ1	3	1067	0.002811621
WDR60	3	1066	0.002814259
ATXN3L	1	355	0.002816901
CCR1	1	355	0.002816901
CCR8	1	355	0.002816901
LGALS9	1	355	0.002816901
NCL	2	710	0.002816901
NDUFA10	1	355	0.002816901
PON1	1	355	0.002816901
STX5	1	355	0.002816901
TRIM9	2	710	0.002816901
PDZD2	8	2839	0.002817894
DLG4	3	1064	0.002819549
LLGL1	3	1064	0.002819549
CAPRIN1	2	709	0.002820874
ATOH1	1	354	0.002824859
FAM181A	1	354	0.002824859
GNAI1	1	354	0.002824859
HMCES	1	354	0.002824859
KLHDC8B	1	354	0.002824859
LRRN3	2	708	0.002824859
P3H2	2	708	0.002824859
POLR3E	2	708	0.002824859
UBE2Z	1	354	0.002824859
WNT11	1	354	0.002824859
NPR1	3	1061	0.002827521

BCL6	2	706	0.002832861
DCST1	2	706	0.002832861
FEZ2	1	353	0.002832861
ISG20L2	1	353	0.002832861
MAFA	1	353	0.002832861
THPO	1	353	0.002832861
THUMPD1	1	353	0.002832861
TMPRSS9	3	1059	0.002832861
TRIM63	1	353	0.002832861
TECPR2	4	1411	0.002834869
ZNF609	4	1411	0.002834869
SLF1	3	1058	0.002835539
CLIP4	2	705	0.002836879
TTC12	2	705	0.002836879
HOXD9	1	352	0.002840909
PSD4	3	1056	0.002840909
RAD51AP1	1	352	0.002840909
CASC3	2	703	0.00284495
GTF3C1	6	2109	0.00284495
RNF214	2	703	0.00284495
USHBP1	2	703	0.00284495
DIS3L	3	1054	0.0028463
CEACAM5	2	702	0.002849003
EIF2B2	1	351	0.002849003
EP400	9	3159	0.002849003
FAM118B	1	351	0.002849003
GBE1	2	702	0.002849003
HAO2	1	351	0.002849003
LPAR2	1	351	0.002849003
NOL9	2	702	0.002849003
PRKACG	1	351	0.002849003
SALL4	3	1053	0.002849003
TMEM115	1	351	0.002849003
WNT8A	1	351	0.002849003
WNT8B	1	351	0.002849003
ZNF124	1	351	0.002849003
ZPBP	1	351	0.002849003
CEP162	4	1403	0.002851033
ACSS2	2	701	0.002853067
PUS7L	2	701	0.002853067
SH3BP1	2	701	0.002853067
ATP6V0D2	1	350	0.002857143
C5AR1	1	350	0.002857143
CCNO	1	350	0.002857143
FOXA3	1	350	0.002857143
KLHDC8A	1	350	0.002857143
NUDT9	1	350	0.002857143

PAX4	1	350	0.002857143
SPESP1	1	350	0.002857143
TGDS	1	350	0.002857143
TMEM206	1	350	0.002857143
TRIM24	3	1050	0.002857143
ULK1	3	1050	0.002857143
ZNF234	2	700	0.002857143
ZNF367	1	350	0.002857143
WDR90	5	1748	0.002860412
MASP1	2	699	0.00286123
METTL13	2	699	0.00286123
NOLC1	2	699	0.00286123
UVRAG	2	699	0.00286123
NLRP8	3	1048	0.002862595
ANKRD30A	4	1397	0.002863278
GLE1	2	698	0.00286533
HARBI1	1	349	0.00286533
PTER	1	349	0.00286533
RPF1	1	349	0.00286533
TAF7	1	349	0.00286533
WNT7A	1	349	0.00286533
WNT7B	1	349	0.00286533
PRKCG	2	697	0.00286944
MARF1	5	1742	0.002870264
ARSA	1	348	0.002873563
C2CD2	2	696	0.002873563
GBX2	1	348	0.002873563
HPR	1	348	0.002873563
JPH2	2	696	0.002873563
OPN1SW	1	348	0.002873563
RHO	1	348	0.002873563
SBK2	1	348	0.002873563
TAAR9	1	348	0.002873563
YOD1	1	348	0.002873563
ZSCAN16	1	348	0.002873563
ASPM	10	3477	0.002876043
ADGRF4	2	695	0.002877698
DVL1	2	695	0.002877698
FSHR	2	695	0.002877698
NUFIP2	2	695	0.002877698
DAO	1	347	0.002881844
GPR148	1	347	0.002881844
JUND	1	347	0.002881844
MAGEB1	1	347	0.002881844
MAP2K3	1	347	0.002881844
MTHFD2L	1	347	0.002881844
OR2M2	1	347	0.002881844

DCLRE1A	3	1040	0.002884615
ADGRG1	2	693	0.002886003
CRTC2	2	693	0.002886003
ZBED5	2	693	0.002886003
PLEKHG1	4	1385	0.002888087
ANXA1	1	346	0.002890173
ENKD1	1	346	0.002890173
HPF1	1	346	0.002890173
MOS	1	346	0.002890173
NEK8	2	692	0.002890173
POLR1C	1	346	0.002890173
PRMT7	2	692	0.002890173
SAE1	1	346	0.002890173
SELENOV	1	346	0.002890173
SUCLG1	1	346	0.002890173
TFB1M	1	346	0.002890173
MAP3K21	3	1036	0.002895753
ARHGEF4	2	690	0.002898551
CEBPB	1	345	0.002898551
CPD	4	1380	0.002898551
FOXL1	1	345	0.002898551
GHITM	1	345	0.002898551
LNX2	2	690	0.002898551
LRTM1	1	345	0.002898551
OR11G2	1	345	0.002898551
SLC27A5	2	690	0.002898551
MEPCE	2	689	0.002902758
THOP1	2	689	0.002902758
PDZD7	3	1033	0.002904163
PHLDB1	4	1377	0.002904866
CALHM3	1	344	0.002906977
CEACAM6	1	344	0.002906977
EPM2A	1	344	0.002906977
FRRS1L	1	344	0.002906977
FST	1	344	0.002906977
KIF5A	3	1032	0.002906977
LRRC75A	1	344	0.002906977
NOL10	2	688	0.002906977
NTM	1	344	0.002906977
OR4A15	1	344	0.002906977
PCGF2	1	344	0.002906977
SNX16	1	344	0.002906977
SRSF6	1	344	0.002906977
TBC1D25	2	688	0.002906977
UBAC2	1	344	0.002906977
ANKRD12	6	2062	0.002909796
ZNF445	3	1031	0.002909796

CLCNKA	2	687	0.002911208
DROSHA	4	1374	0.002911208
TGM2	2	687	0.002911208
APOL6	1	343	0.002915452
ATP1A4	3	1029	0.002915452
BDH1	1	343	0.002915452
KIAA1324L	3	1029	0.002915452
MAMDC2	2	686	0.002915452
MRNIP	1	343	0.002915452
PNCK	1	343	0.002915452
TBXA2R	1	343	0.002915452
CNTN3	3	1028	0.002918288
BMPER	2	685	0.002919708
RNF103	2	685	0.002919708
MYO18A	6	2054	0.00292113
ABHD4	1	342	0.002923977
AQP9	1	342	0.002923977
ARRDC5	1	342	0.002923977
CNTN4	3	1026	0.002923977
CXCR6	1	342	0.002923977
DMRTB1	1	342	0.002923977
GALM	1	342	0.002923977
GPSM2	2	684	0.002923977
HOXC10	1	342	0.002923977
PRPF18	1	342	0.002923977
RNLS	1	342	0.002923977
SIRPB2	1	342	0.002923977
TMEM59L	1	342	0.002923977
TTPAL	1	342	0.002923977
WBP1L	1	342	0.002923977
NAT10	3	1025	0.002926829
ACSL5	2	683	0.002928258
LRRN1	2	683	0.002928258
PRKCH	2	683	0.002928258
SBNO2	4	1366	0.002928258
TGFBI	2	683	0.002928258
THOC5	2	683	0.002928258
SPTBN2	7	2390	0.00292887
ARFIP2	1	341	0.002932551
C1orf56	1	341	0.002932551
C9orf64	1	341	0.002932551
CAB39	1	341	0.002932551
CASP12	1	341	0.002932551
CCNY	1	341	0.002932551
FAM13A	3	1023	0.002932551
MR1	1	341	0.002932551
MSANTD3-TMEFF1	1	341	0.002932551

THOC6	1	341	0.002932551
ACOX2	2	681	0.002936858
GAS2L1	2	681	0.002936858
EPHB6	3	1021	0.002938296
CEMIP	4	1361	0.002939015
TIAM2	5	1701	0.002939447
DUSP12	1	340	0.002941176
FERMT2	2	680	0.002941176
GLDC	3	1020	0.002941176
GNB2	1	340	0.002941176
GNPAT	2	680	0.002941176
GPALPP1	1	340	0.002941176
HAPLN2	1	340	0.002941176
HEATR3	2	680	0.002941176
HOXD10	1	340	0.002941176
MCHR2	1	340	0.002941176
NSUN3	1	340	0.002941176
OR5T3	1	340	0.002941176
PAWR	1	340	0.002941176
PHF21A	2	680	0.002941176
REM2	1	340	0.002941176
RPGR	3	1020	0.002941176
ST3GAL1	1	340	0.002941176
STKLD1	2	680	0.002941176
TP63	2	680	0.002941176
ZNF488	1	340	0.002941176
JCAD	4	1359	0.002943341
DNHD1	14	4753	0.002945508
MISP	2	679	0.002945508
RTL1	4	1358	0.002945508
KMT2B	8	2715	0.002946593
CTSB	1	339	0.002949853
DBX2	1	339	0.002949853
ERMARD	2	678	0.002949853
IGBP1	1	339	0.002949853
KDR	4	1356	0.002949853
PHF20L1	3	1017	0.002949853
RAD51	1	339	0.002949853
USP50	1	339	0.002949853
ZNF665	2	678	0.002949853
SMG5	3	1016	0.002952756
POR	2	677	0.00295421
TAF6	2	677	0.00295421
TMCO3	2	677	0.00295421
TOPAZ1	5	1692	0.002955083
PTPRN2	3	1015	0.002955665
ADCY9	4	1353	0.002956393

DFFB	1	338	0.00295858
FBP1	1	338	0.00295858
GAB2	2	676	0.00295858
GPR160	1	338	0.00295858
HOXD11	1	338	0.00295858
KIF1A	5	1690	0.00295858
LSAMP	1	338	0.00295858
SLC25A37	1	338	0.00295858
SLC25A40	1	338	0.00295858
SLC35G6	1	338	0.00295858
TAS2R39	1	338	0.00295858
TCF25	2	676	0.00295858
USP31	4	1352	0.00295858
ZNF418	2	676	0.00295858
MIB2	3	1013	0.0029615
VPS13D	13	4388	0.002962625
BMX	2	675	0.002962963
SLC25A13	2	675	0.002962963
KL	3	1012	0.002964427
UBA7	3	1012	0.002964427
IGSF9B	4	1349	0.002965159
ABHD13	1	337	0.002967359
CA14	1	337	0.002967359
DGAT2L6	1	337	0.002967359
DNAJB4	1	337	0.002967359
FIGNL1	2	674	0.002967359
HEY2	1	337	0.002967359
IFNGR2	1	337	0.002967359
OXGR1	1	337	0.002967359
RAPGEF4	3	1011	0.002967359
SLC35D2	1	337	0.002967359
XPNPEP2	2	674	0.002967359
NFASC	4	1347	0.002969562
ABCG8	2	673	0.002971768
ADGRF5	4	1346	0.002971768
FXR2	2	673	0.002971768
KIZ	2	673	0.002971768
GRID1	3	1009	0.002973241
HLTF	3	1009	0.002973241
AACS	2	672	0.00297619
ACKR1	1	336	0.00297619
CAPN10	2	672	0.00297619
GPR82	1	336	0.00297619
HIBADH	1	336	0.00297619
IL17REL	1	336	0.00297619
LGALS12	1	336	0.00297619
MAGEB17	1	336	0.00297619

NDFIP2	1	336	0.00297619
PRKCA	2	672	0.00297619
SPDYE1	1	336	0.00297619
ST6GALNAC5	1	336	0.00297619
TOR1B	1	336	0.00297619
PCDHAC2	3	1007	0.002979146
HAP1	2	671	0.002980626
SORBS3	2	671	0.002980626
ZNF282	2	671	0.002980626
NUP205	6	2012	0.002982107
ARHGEF39	1	335	0.002985075
ATF6	2	670	0.002985075
B3GAT3	1	335	0.002985075
CD1D	1	335	0.002985075
DVL1	2	670	0.002985075
FAS	1	335	0.002985075
LCA5L	2	670	0.002985075
METAP1D	1	335	0.002985075
OR9K2	1	335	0.002985075
PSG5	1	335	0.002985075
RHPN1	2	670	0.002985075
SPDEF	1	335	0.002985075
TADA1	1	335	0.002985075
TRAF7	2	670	0.002985075
GUF1	2	669	0.002989537
MMP15	2	669	0.002989537
ZMYM5	2	669	0.002989537
MBD6	3	1003	0.002991027
DHDH	1	334	0.002994012
GMNC	1	334	0.002994012
LCMT1	1	334	0.002994012
LDHB	1	334	0.002994012
MAGIX	1	334	0.002994012
MAT2B	1	334	0.002994012
MOGAT2	1	334	0.002994012
MTG1	1	334	0.002994012
RSRC1	1	334	0.002994012
SUCNR1	1	334	0.002994012
VAX1	1	334	0.002994012
MICAL3	6	2002	0.002997003
SKOR2	3	1001	0.002997003
TOP3A	3	1001	0.002997003
CCDC154	2	667	0.002998501
FERMT3	2	667	0.002998501
IL17RE	2	667	0.002998501
STRA6	2	667	0.002998501
CHD3	6	2000	0.003

APBB1IP	2	666	0.003003003
CD1C	1	333	0.003003003
CTSL	1	333	0.003003003
EN2	1	333	0.003003003
GJA4	1	333	0.003003003
GPR33	1	333	0.003003003
ST3GAL4	1	333	0.003003003
TAS2R38	1	333	0.003003003
EPHB3	3	998	0.003006012
GIMAP8	2	665	0.003007519
NCKAP5L	4	1330	0.003007519
PADI2	2	665	0.003007519
SH3KBP1	2	665	0.003007519
APPL2	2	664	0.003012048
HNRNPAB	1	332	0.003012048
KCNK17	1	332	0.003012048
LDHA	1	332	0.003012048
NEIL2	1	332	0.003012048
SIX3	1	332	0.003012048
SLAMF6	1	332	0.003012048
ZNF710	2	664	0.003012048
CSPG4	7	2322	0.003014643
AGAP6	2	663	0.003016591
ALS2	5	1657	0.003017502
HELZ2	8	2649	0.003020008
EPM2A	1	331	0.003021148
FLT3	3	993	0.003021148
PARVG	1	331	0.003021148
SALL1	4	1324	0.003021148
TAL1	1	331	0.003021148
TRUB2	1	331	0.003021148
ZNF660	1	331	0.003021148
JPH1	2	661	0.003025719
ADGRA3	4	1321	0.003028009
AIP	1	330	0.003030303
ALAD	1	330	0.003030303
GINM1	1	330	0.003030303
GPR3	1	330	0.003030303
MTMR7	2	660	0.003030303
REPS2	2	660	0.003030303
SCN8A	6	1980	0.003030303
WDR5B	1	330	0.003030303
CACNA1F	6	1977	0.003034901
CEP76	2	659	0.003034901
TRMT1	2	659	0.003034901
SCUBE1	3	988	0.003036437
B3GALT6	1	329	0.003039514

C3orf38	1	329	0.003039514
C6orf163	1	329	0.003039514
CPT2	2	658	0.003039514
CTSK	1	329	0.003039514
FAM43B	1	329	0.003039514
KCTD13	1	329	0.003039514
SLC7A2	2	658	0.003039514
TSSC4	1	329	0.003039514
ZZEF1	9	2961	0.003039514
CEP120	3	986	0.003042596
EDEM1	2	657	0.00304414
RIN3	3	985	0.003045685
ATXN2	4	1313	0.003046458
ATPAF1	1	328	0.00304878
C1orf127	2	656	0.00304878
HEYL	1	328	0.00304878
HOXD1	1	328	0.00304878
MSI2	1	328	0.00304878
NPBWR1	1	328	0.00304878
POU5F2	1	328	0.00304878
PRSS48	1	328	0.00304878
RAD50	4	1312	0.00304878
RCN3	1	328	0.00304878
SDS	1	328	0.00304878
PHC3	3	983	0.003051882
ACADVL	2	655	0.003053435
FOXO1	2	655	0.003053435
LMLN	2	655	0.003053435
USP24	8	2620	0.003053435
FRMPD2	4	1309	0.003055768
B4GALT7	1	327	0.003058104
CLVS2	1	327	0.003058104
COL25A1	2	654	0.003058104
COQ5	1	327	0.003058104
GLB1L	2	654	0.003058104
GPS2	1	327	0.003058104
HOGA1	1	327	0.003058104
HSPA5	2	654	0.003058104
MAPRE2	1	327	0.003058104
NAIF1	1	327	0.003058104
OR6A2	1	327	0.003058104
PNRC1	1	327	0.003058104
RAB3GAP1	3	981	0.003058104
TNFAIP2	2	654	0.003058104
TNRC6A	6	1962	0.003058104
VPS26A	1	327	0.003058104
GRIK5	3	980	0.003061224

NYAP2	2	653	0.003062787
TRIM32	2	653	0.003062787
ARID1A	7	2285	0.003063457
DEDD2	1	326	0.003067485
DOK4	1	326	0.003067485
MTHFD1L	3	978	0.003067485
NRCAM	4	1304	0.003067485
OLFML2A	2	652	0.003067485
OR11H12	1	326	0.003067485
OR51M1	1	326	0.003067485
OR5T1	1	326	0.003067485
PIM3	1	326	0.003067485
SCRIB	5	1630	0.003067485
VGLL3	1	326	0.003067485
SLITRK3	3	977	0.003070624
MELK	2	651	0.003072197
TDRD3	2	651	0.003072197
ACP5	1	325	0.003076923
CYC1	1	325	0.003076923
EIF3I	1	325	0.003076923
HMGCL	1	325	0.003076923
IGFBP2	1	325	0.003076923
KCTD12	1	325	0.003076923
LDAH	1	325	0.003076923
MACROD1	1	325	0.003076923
MRPL1	1	325	0.003076923
NEDD4L	3	975	0.003076923
OR1S2	1	325	0.003076923
PRG3	1	325	0.003076923
RNF20	3	975	0.003076923
SALL3	4	1300	0.003076923
TNIP3	1	325	0.003076923
LPA	14	4548	0.003078276
ABCA9	5	1624	0.003078818
ABCA12	8	2595	0.003082852
VPS18	3	973	0.003083248
ANXA10	1	324	0.00308642
AVL9	2	648	0.00308642
CAPS2	4	1296	0.00308642
DDX43	2	648	0.00308642
MAGEB16	1	324	0.00308642
NKX2-5	1	324	0.00308642
OR56B1	1	324	0.00308642
PSMD7	1	324	0.00308642
PUM3	2	648	0.00308642
SLC2A13	2	648	0.00308642
TBC1D17	2	648	0.00308642

CLASP2	4	1294	0.00309119
FAM161B	2	647	0.00309119
FZD1	2	647	0.00309119
MYO3A	5	1616	0.003094059
SHROOM2	5	1616	0.003094059
ASB11	1	323	0.003095975
ATG2A	6	1938	0.003095975
B3GAT2	1	323	0.003095975
OR2AE1	1	323	0.003095975
OR4K5	1	323	0.003095975
OR51S1	1	323	0.003095975
OR52B2	1	323	0.003095975
PLK3	2	646	0.003095975
PPWD1	2	646	0.003095975
WEE1	2	646	0.003095975
YDJC	1	323	0.003095975
PDIA4	2	645	0.003100775
ADAMTS1	3	967	0.003102378
ANPEP	3	967	0.003102378
KIAA0753	3	967	0.003102378
ZKSCAN2	3	967	0.003102378
ARG1	1	322	0.00310559
C2orf71	4	1288	0.00310559
CRHBP	1	322	0.00310559
MAP3K13	3	966	0.00310559
MRGPRX3	1	322	0.00310559
PARP16	1	322	0.00310559
RNF113B	1	322	0.00310559
SFXN2	1	322	0.00310559
SLC35B1	1	322	0.00310559
ZNF568	2	644	0.00310559
ENOX1	2	643	0.00311042
SLC5A5	2	643	0.00311042
SLCO2A1	2	643	0.00311042
PREX2	5	1606	0.003113325
C16orf62	3	963	0.003115265
GJA9	1	321	0.003115265
GJD2	1	321	0.003115265
LRP8	3	963	0.003115265
RASSF4	1	321	0.003115265
TMUB2	1	321	0.003115265
TSTA3	1	321	0.003115265
UBE2U	1	321	0.003115265
PLXND1	6	1925	0.003116883
KIAA1211L	3	962	0.003118503
WBP11	2	641	0.003120125
CAPN5	2	640	0.003125

OR2C3	1	320	0.003125
OR51E2	1	320	0.003125
OR7C1	1	320	0.003125
SH3D21	2	640	0.003125
TSPAN32	1	320	0.003125
VSTM4	1	320	0.003125
ZNF549	2	640	0.003125
GTF2IRD1	3	959	0.003128259
PEX5	2	639	0.00312989
SF1	2	639	0.00312989
SLC17A2	2	639	0.00312989
C8orf48	1	319	0.003134796
CRYL1	1	319	0.003134796
GPR55	1	319	0.003134796
KCNC2	2	638	0.003134796
KLK3	2	638	0.003134796
NUBPL	1	319	0.003134796
OR10Q1	1	319	0.003134796
OR2S2	1	319	0.003134796
OR7A5	1	319	0.003134796
OR8K1	1	319	0.003134796
TMEM121	1	319	0.003134796
ZNF143	2	638	0.003134796
AMOTL1	3	956	0.003138075
GOLGA4	7	2230	0.003139013
MEI1	4	1274	0.003139717
MDGA1	3	955	0.003141361
COQ9	1	318	0.003144654
GDPD3	1	318	0.003144654
IL27RA	2	636	0.003144654
OR13C2	1	318	0.003144654
OR13C9	1	318	0.003144654
OR1B1	1	318	0.003144654
OR2T34	1	318	0.003144654
OR56A1	1	318	0.003144654
PLSCR1	1	318	0.003144654
RDH5	1	318	0.003144654
ALPK3	6	1907	0.003146303
PTPRF	6	1907	0.003146303
TRAK1	3	953	0.003147954
ZC3H18	3	953	0.003147954
ZNF507	3	953	0.003147954
CACNA1I	7	2223	0.003148898
KCNC4	2	635	0.003149606
CABIN1	7	2220	0.003153153
APOE	1	317	0.003154574
C16orf86	1	317	0.003154574

CA5B	1	317	0.003154574
DUS4L	1	317	0.003154574
FCGR2A	1	317	0.003154574
HMG20B	1	317	0.003154574
IBSP	1	317	0.003154574
LAPTM4B	1	317	0.003154574
OR2B11	1	317	0.003154574
OR51E1	1	317	0.003154574
OR52M1	1	317	0.003154574
OR6Q1	1	317	0.003154574
RCN2	1	317	0.003154574
RLBP1	1	317	0.003154574
SLC6A19	2	634	0.003154574
TAS2R14	1	317	0.003154574
UPP2	1	317	0.003154574
VGLL2	1	317	0.003154574
ZNF592	4	1267	0.003157064
TBC1D9	4	1266	0.003159558
AP1B1	3	949	0.003161222
AKR1B15	1	316	0.003164557
CCDC42	1	316	0.003164557
ELP5	1	316	0.003164557
GOLGA8M	2	632	0.003164557
ICK	2	632	0.003164557
OR2AG1	1	316	0.003164557
OR2AG2	1	316	0.003164557
OR2G6	1	316	0.003164557
OR2K2	1	316	0.003164557
OR2T11	1	316	0.003164557
OR5AP2	1	316	0.003164557
POLR3F	1	316	0.003164557
RGS22	4	1264	0.003164557
TAS2R3	1	316	0.003164557
TNFAIP1	1	316	0.003164557
ZNF860	2	632	0.003164557
IARS	4	1262	0.003169572
TBRG4	2	631	0.003169572
ITPKB	3	946	0.003171247
C1GALT1C1L	1	315	0.003174603
EIF2A	1	315	0.003174603
NGDN	1	315	0.003174603
OR10H2	1	315	0.003174603
OR2V1	1	315	0.003174603
OR8J3	1	315	0.003174603
SOWAHD	1	315	0.003174603
UNC5B	3	945	0.003174603
ZFP37	2	630	0.003174603

UBR3	6	1888	0.003177966
SYNJ1	5	1573	0.00317864
INPPL1	4	1258	0.00317965
PLS1	2	629	0.00317965
TNC	7	2201	0.003180373
L1CAM	4	1257	0.00318218
PPFIA2	4	1257	0.00318218
ART4	1	314	0.003184713
BCCIP	1	314	0.003184713
FAHD2B	1	314	0.003184713
FBXO21	2	628	0.003184713
JPH4	2	628	0.003184713
MAGEA6	1	314	0.003184713
OR10T2	1	314	0.003184713
OR4K2	1	314	0.003184713
OR5D13	1	314	0.003184713
OR5D14	1	314	0.003184713
OR9A4	1	314	0.003184713
PRSS21	1	314	0.003184713
SERHL2	1	314	0.003184713
SLC39A3	1	314	0.003184713
SPP1	1	314	0.003184713
THAP11	1	314	0.003184713
TREX1	1	314	0.003184713
YIF1B	1	314	0.003184713
CHRNA4	2	627	0.003189793
STARD9	15	4700	0.003191489
ANKRA2	1	313	0.003194888
GNAS	2	626	0.003194888
KCTD10	1	313	0.003194888
LRRC52	1	313	0.003194888
NR4A3	2	626	0.003194888
OR1G1	1	313	0.003194888
OR1J2	1	313	0.003194888
OR1M1	1	313	0.003194888
OR4E2	1	313	0.003194888
OR4M1	1	313	0.003194888
OR4Q3	1	313	0.003194888
OR56A4	1	313	0.003194888
OR6V1	1	313	0.003194888
PIM1	1	313	0.003194888
REV3L	10	3130	0.003194888
SYNM	5	1565	0.003194888
SYP	1	313	0.003194888
TESK1	2	626	0.003194888
UNG	1	313	0.003194888
ZNF189	2	626	0.003194888

ZNF471	2	626	0.003194888
NWD1	5	1564	0.003196931
ANKLE2	3	938	0.003198294
MAGEL2	4	1249	0.003202562
UTRN	11	3433	0.003204195
ACTR8	2	624	0.003205128
AFTPH	3	936	0.003205128
CA4	1	312	0.003205128
HSD17B12	1	312	0.003205128
KEAP1	2	624	0.003205128
LOR	1	312	0.003205128
NTHL1	1	312	0.003205128
OR2L13	1	312	0.003205128
OR2L2	1	312	0.003205128
OR2L8	1	312	0.003205128
OR51B2	1	312	0.003205128
OR6B2	1	312	0.003205128
OR6C75	1	312	0.003205128
OR6C76	1	312	0.003205128
OR6X1	1	312	0.003205128
OR7D2	1	312	0.003205128
OR7D4	1	312	0.003205128
OR7G3	1	312	0.003205128
OXNAD1	1	312	0.003205128
PCDHA5	3	936	0.003205128
RNASEH2B	1	312	0.003205128
DOCK5	6	1870	0.003208556
ABCF2	2	623	0.003210273
XPNPEP1	2	623	0.003210273
ARHGAP40	2	622	0.003215434
CCDC17	2	622	0.003215434
FAM234B	2	622	0.003215434
FRMD6	2	622	0.003215434
OR10G8	1	311	0.003215434
OR2A5	1	311	0.003215434
OR5M8	1	311	0.003215434
OR5P3	1	311	0.003215434
OR6B1	1	311	0.003215434
OR8D2	1	311	0.003215434
OR8H1	1	311	0.003215434
PPIL6	1	311	0.003215434
SLC25A1	1	311	0.003215434
TAF6L	2	622	0.003215434
TMEM177	1	311	0.003215434
PCDHGA2	3	932	0.003218884
PCDHGA7	3	932	0.003218884
PMS1	3	932	0.003218884

SRL	3	932	0.003218884
CEP295NL	2	621	0.003220612
ELL	2	621	0.003220612
KLHL40	2	621	0.003220612
MINDY2	2	621	0.003220612
TOX4	2	621	0.003220612
MKL1	3	931	0.003222342
PCDHGA1	3	931	0.003222342
TRPC6	3	931	0.003222342
AMMECR1L	1	310	0.003225806
B3GALT5	1	310	0.003225806
GPN2	1	310	0.003225806
JAM3	1	310	0.003225806
NTAN1	1	310	0.003225806
OR2A4	1	310	0.003225806
OR2A42	1	310	0.003225806
OR51B4	1	310	0.003225806
OR5H14	1	310	0.003225806
OR5M9	1	310	0.003225806
OR9Q1	1	310	0.003225806
SLC6A3	2	620	0.003225806
ZFP42	1	310	0.003225806
SCYL2	3	929	0.003229279
DDX59	2	619	0.003231018
FER1L6	6	1857	0.003231018
KLC4	2	619	0.003231018
MAP3K2	2	619	0.003231018
SLC7A3	2	619	0.003231018
TCF7L2	2	619	0.003231018
ZNF668	2	619	0.003231018
AURKC	1	309	0.003236246
CNN2	1	309	0.003236246
MNAT1	1	309	0.003236246
OR10J5	1	309	0.003236246
OR1L8	1	309	0.003236246
OR2AP1	1	309	0.003236246
OR4B1	1	309	0.003236246
OR4C13	1	309	0.003236246
OR4C6	1	309	0.003236246
OR5AC2	1	309	0.003236246
OR5B2	1	309	0.003236246
OR6C4	1	309	0.003236246
TAS2R31	1	309	0.003236246
TAS2R46	1	309	0.003236246
THAP7	1	309	0.003236246
UPRT	1	309	0.003236246
WBP2NL	1	309	0.003236246

DDX58	3	925	0.003243243
NUP107	3	925	0.003243243
GRIN2C	4	1233	0.00324412
MEGF6	5	1541	0.003244646
ASRGL1	1	308	0.003246753
C14orf159	2	616	0.003246753
CA6	1	308	0.003246753
CFAP73	1	308	0.003246753
COPE	1	308	0.003246753
ENTPD4	2	616	0.003246753
KLHL36	2	616	0.003246753
OR2D2	1	308	0.003246753
RFX5	2	616	0.003246753
RPA1	2	616	0.003246753
SPG21	1	308	0.003246753
ACSF2	2	615	0.003252033
KCNT1	4	1230	0.003252033
TBKBP1	2	615	0.003252033
ZNF653	2	615	0.003252033
PKD1	14	4303	0.003253544
C2orf78	3	922	0.003253796
ANKRD55	2	614	0.003257329
HS3ST1	1	307	0.003257329
MMRN1	4	1228	0.003257329
OR4N2	1	307	0.003257329
PABPC1L	2	614	0.003257329
SCRT2	1	307	0.003257329
SLC39A9	1	307	0.003257329
TAS2R10	1	307	0.003257329
RBM20	4	1227	0.003259984
AR	3	920	0.00326087
IGSF8	2	613	0.003262643
CLCA4	3	919	0.003264418
WDR47	3	919	0.003264418
ABHD10	1	306	0.003267974
INCENP	3	918	0.003267974
KBTBD3	2	612	0.003267974
MRPL45	1	306	0.003267974
OTOP1	2	612	0.003267974
PGM2	2	612	0.003267974
PPT1	1	306	0.003267974
NSMAF	3	917	0.003271538
LTA4H	2	611	0.003273322
PLEKHN1	2	611	0.003273322
PCNX2	7	2137	0.00327562
ADGRG6	4	1221	0.003276003
CDK3	1	305	0.003278689

CLDN16	1	305	0.003278689
DNASE1L3	1	305	0.003278689
GNPTG	1	305	0.003278689
NT5C1B	2	610	0.003278689
OR5M11	1	305	0.003278689
PAF1	1	305	0.003278689
PEX26	1	305	0.003278689
RETSAT	2	610	0.003278689
RNF148	1	305	0.003278689
SLC5A8	2	610	0.003278689
STK16	1	305	0.003278689
TSPAN12	1	305	0.003278689
RALGDS	3	914	0.003282276
SLIT3	5	1523	0.003282994
PDE12	2	609	0.003284072
DIO3	1	304	0.003289474
DTWD1	1	304	0.003289474
NSMCE3	1	304	0.003289474
PRR16	1	304	0.003289474
SLC25A34	1	304	0.003289474
TOMM70	2	608	0.003289474
FAM208B	8	2430	0.003292181
NFXL1	3	911	0.003293085
ZNF721	3	911	0.003293085
DMXL2	10	3036	0.003293808
WDR62	5	1518	0.003293808
MYRFL	3	910	0.003296703
RAD54B	3	910	0.003296703
CDK4	1	303	0.00330033
CTSZ	1	303	0.00330033
ECHDC3	1	303	0.00330033
OR4X2	1	303	0.00330033
RHBDL2	1	303	0.00330033
RNF144B	1	303	0.00330033
SCARA3	2	606	0.00330033
TM9SF1	2	606	0.00330033
SKIDA1	3	908	0.003303965
GDPD5	2	605	0.003305785
PODXL2	2	605	0.003305785
LGR5	3	907	0.003307607
WHRN	3	907	0.003307607
ADAM22	3	906	0.003311258
ALKBH4	1	302	0.003311258
CTNNA1	3	906	0.003311258
NEK7	1	302	0.003311258
NTN1	2	604	0.003311258
RECQL4	4	1208	0.003311258

STX17	1	302	0.003311258
TMC2	3	906	0.003311258
ZNF300	2	604	0.003311258
TTF1	3	905	0.003314917
METTL25	2	603	0.00331675
CELSR1	10	3014	0.00331785
KANSL3	3	904	0.003318584
TCHHL1	3	904	0.003318584
ATP13A1	4	1204	0.003322259
EBF4	2	602	0.003322259
EVPLL	1	301	0.003322259
LARS2	3	903	0.003322259
NKX2-6	1	301	0.003322259
ORAI1	1	301	0.003322259
POU6F1	1	301	0.003322259
PPIE	1	301	0.003322259
SCO1	1	301	0.003322259
SLC25A15	1	301	0.003322259
CAMTA2	4	1202	0.003327787
GALNT17	2	601	0.003327787
UBQLN4	2	601	0.003327787
DSC2	3	901	0.003329634
ADGRV1	21	6306	0.003330162
ANKRD54	1	300	0.003333333
CD38	1	300	0.003333333
GID4	1	300	0.003333333
GIMAP7	1	300	0.003333333
MLPH	2	600	0.003333333
NT5C3B	1	300	0.003333333
PHF13	1	300	0.003333333
SPATS1	1	300	0.003333333
TMEM158	1	300	0.003333333
CIZ1	3	898	0.003340757
DNAH8	15	4490	0.003340757
KIF18A	3	898	0.003340757
C1orf94	2	598	0.003344482
CACNB1	2	598	0.003344482
CHD1L	3	897	0.003344482
CRX	1	299	0.003344482
CTIF	2	598	0.003344482
F11R	1	299	0.003344482
GALNT17	2	598	0.003344482
KCNH7	4	1196	0.003344482
NHEJ1	1	299	0.003344482
NMRAL1	1	299	0.003344482
PPP1R3D	1	299	0.003344482
RGN	1	299	0.003344482

SC5D	1	299	0.003344482
SETD9	1	299	0.003344482
TMEM38A	1	299	0.003344482
ZDHHC3	1	299	0.003344482
FLG2	8	2391	0.00334588
EPS15	3	896	0.003348214
SHROOM4	5	1493	0.003348962
IGSF3	4	1194	0.003350084
KARS	2	597	0.003350084
NDOR1	2	597	0.003350084
DAG1	3	895	0.003351955
SF3B2	3	895	0.003351955
MAGI1	5	1491	0.003353454
GOLPH3	1	298	0.003355705
KHK	1	298	0.003355705
MID1	1	298	0.003355705
MYLK2	2	596	0.003355705
OTOP3	2	596	0.003355705
PRR32	1	298	0.003355705
SFMBT2	3	894	0.003355705
SLC5A10	2	596	0.003355705
TNNT2	1	298	0.003355705
MVP	3	893	0.003359462
ABCA13	17	5058	0.003361012
ESR1	2	595	0.003361345
TNFRSF8	2	595	0.003361345
A1CF	2	594	0.003367003
ARID5A	2	594	0.003367003
CHDH	2	594	0.003367003
DSCR3	1	297	0.003367003
FAM212B	1	297	0.003367003
GRB10	2	594	0.003367003
MEMO1	1	297	0.003367003
MPST	1	297	0.003367003
PIGC	1	297	0.003367003
RNF212	1	297	0.003367003
RPL5	1	297	0.003367003
SETD3	2	594	0.003367003
SHC3	2	594	0.003367003
SLC4A11	3	891	0.003367003
TACO1	1	297	0.003367003
TMEM169	1	297	0.003367003
TMEM178A	1	297	0.003367003
UBXN1	1	297	0.003367003
WDR63	3	891	0.003367003
PRKD3	3	890	0.003370787
BBS1	2	593	0.003372681

CRY2	2	593	0.003372681
GATAD2B	2	593	0.003372681
FCHO1	3	889	0.003374578
ATIC	2	592	0.003378378
CAMLG	1	296	0.003378378
CANX	2	592	0.003378378
CAT	1	296	0.003378378
CD74	1	296	0.003378378
GLYAT	1	296	0.003378378
IMP4	2	592	0.003378378
LYSMD4	1	296	0.003378378
SULT1B1	1	296	0.003378378
TAF15	2	592	0.003378378
TMEM156	1	296	0.003378378
TTLL2	2	592	0.003378378
MRC2	5	1479	0.003380663
BBS9	3	887	0.003382187
PAN3	3	887	0.003382187
FYCO1	5	1478	0.00338295
ABL2	4	1182	0.003384095
GBP2	2	591	0.003384095
AGBL5	3	886	0.003386005
WDR35	4	1181	0.00338696
AQP12B	1	295	0.003389831
AQP9	1	295	0.003389831
C2orf72	1	295	0.003389831
CDHR3	3	885	0.003389831
F3	1	295	0.003389831
NAPA	1	295	0.003389831
PWWP2B	2	590	0.003389831
SFRP2	1	295	0.003389831
STAP1	1	295	0.003389831
IGSF9	4	1179	0.003392706
FAM117B	2	589	0.003395586
KLHL25	2	589	0.003395586
TAF5L	2	589	0.003395586
TM9SF3	2	589	0.003395586
ATP11B	4	1177	0.003398471
LRRC38	1	294	0.003401361
STX19	1	294	0.003401361
ZNF346	1	294	0.003401361
ZNF676	2	588	0.003401361
ADAMTSL1	6	1762	0.003405221
LRRC66	3	880	0.003409091
DSCAML1	7	2053	0.003409644
CRY1	2	586	0.003412969
ELMOD2	1	293	0.003412969

MASTL	3	879	0.003412969
MSMO1	1	293	0.003412969
OTUD6B	1	293	0.003412969
PDPR	3	879	0.003412969
TMPRSS13	2	586	0.003412969
AP5B1	3	878	0.003416856
PRKD2	3	878	0.003416856
DLEC1	6	1755	0.003418803
ITGAL	4	1170	0.003418803
CACNA1B	8	2339	0.003420265
BCAS1	2	584	0.003424658
BTN3A3	2	584	0.003424658
C11orf57	1	292	0.003424658
CCND3	1	292	0.003424658
CLDN23	1	292	0.003424658
FBXO16	1	292	0.003424658
GIMAP6	1	292	0.003424658
HNMT	1	292	0.003424658
KRTAP10-9	1	292	0.003424658
ME2	2	584	0.003424658
MTFR1L	1	292	0.003424658
FBXO41	3	875	0.003428571
MIOS	3	875	0.003428571
OTOGL	8	2332	0.003430532
RALGPS2	2	583	0.003430532
STS	2	583	0.003430532
TOR1AIP1	2	583	0.003430532
UBR1	6	1749	0.003430532
KMT2D	19	5537	0.003431461
ATP8A1	4	1164	0.003436426
C19orf66	1	291	0.003436426
EXOSC7	1	291	0.003436426
GLTPD2	1	291	0.003436426
METTL8	1	291	0.003436426
PHYHD1	1	291	0.003436426
PKHD1	14	4074	0.003436426
PQLC2	1	291	0.003436426
SIX2	1	291	0.003436426
SLC25A30	1	291	0.003436426
STARD10	1	291	0.003436426
TLX3	1	291	0.003436426
ZC3H8	1	291	0.003436426
LRP2	16	4655	0.003437164
PRR12	7	2036	0.003438114
RALGAPA1	7	2036	0.003438114
CFAP58	3	872	0.003440367
MLH3	5	1453	0.003441156

CSMD2	12	3487	0.003441354
SLAIN2	2	581	0.003442341
SLC9A8	2	581	0.003442341
SOCS6	2	581	0.003442341
EVPL	7	2033	0.003443187
SULF1	3	871	0.003444317
C22orf31	1	290	0.003448276
CA8	1	290	0.003448276
CCNK	2	580	0.003448276
ERGIC1	1	290	0.003448276
METTL3	2	580	0.003448276
PRSS27	1	290	0.003448276
RAPGEF5	2	580	0.003448276
SULF2	3	870	0.003448276
CCDC88C	7	2028	0.003451677
CLCN6	3	869	0.003452244
FAAH	2	579	0.003454231
NRIP1	4	1158	0.003454231
WDFY4	11	3184	0.003454774
PTCH1	5	1447	0.003455425
RGS12	5	1447	0.003455425
PCM1	7	2024	0.003458498
FAM92A	1	289	0.003460208
GNPDA1	1	289	0.003460208
MRGPRG	1	289	0.003460208
PXK	2	578	0.003460208
RARS2	2	578	0.003460208
SGCD	1	289	0.003460208
SLFN12	2	578	0.003460208
TXNL1	1	289	0.003460208
DMGDH	3	866	0.003464203
ACSM2A	2	577	0.003466205
MSL2	2	577	0.003466205
SLC15A4	2	577	0.003466205
STAU1	2	577	0.003466205
FREM2	11	3169	0.003471127
ASB8	1	288	0.003472222
DLG2	2	576	0.003472222
EPN1	2	576	0.003472222
GLYATL3	1	288	0.003472222
GRK6	2	576	0.003472222
LACTB2	1	288	0.003472222
NAXE	1	288	0.003472222
PGLYRP2	2	576	0.003472222
PSMG1	1	288	0.003472222
TMCO5A	1	288	0.003472222
TOX3	2	576	0.003472222

LRRK1	7	2015	0.003473945
AOAH	2	575	0.003478261
IL1RL2	2	575	0.003478261
KCNA3	2	575	0.003478261
POLL	2	575	0.003478261
PRTG	4	1150	0.003478261
LRP1B	16	4599	0.003479017
NSD3	5	1437	0.003479471
VPS13B	14	4022	0.003480855
ASXL2	5	1435	0.003484321
C10orf71	5	1435	0.003484321
C1QL2	1	287	0.003484321
DLX3	1	287	0.003484321
FCAR	1	287	0.003484321
FOXJ2	2	574	0.003484321
NEURL1	2	574	0.003484321
ORC1	3	861	0.003484321
ROGDI	1	287	0.003484321
SGSM1	4	1148	0.003484321
STAG1	1	287	0.003484321
ANKAR	5	1434	0.00348675
LRRD1	3	860	0.003488372
ASPG	2	573	0.003490401
MYRIP	3	859	0.003492433
CASKIN1	5	1431	0.00349406
CLN8	1	286	0.003496503
HSPH1	3	858	0.003496503
ME1	2	572	0.003496503
RNH1	1	286	0.003496503
NOP14	3	857	0.003500583
VPS8	5	1428	0.003501401
COL19A1	4	1142	0.003502627
PRR35	2	571	0.003502627
VILL	3	856	0.003504673
C1QTNF2	1	285	0.003508772
CYBB	2	570	0.003508772
FAM227A	2	570	0.003508772
HEATR9	2	570	0.003508772
MKKS	2	570	0.003508772
SLAMF8	1	285	0.003508772
VPS37B	1	285	0.003508772
KIAA1210	6	1709	0.003510825
PDE6B	3	854	0.003512881
ARSI	2	569	0.003514938
CFHR5	2	569	0.003514938
SLC43A2	2	569	0.003514938
SPATA16	2	569	0.003514938

TIE1	4	1138	0.003514938
APC	10	2843	0.003517411
EXPH5	7	1989	0.003519356
ERMN	1	284	0.003521127
FHL5	1	284	0.003521127
ICMT	1	284	0.003521127
KDM1A	3	852	0.003521127
PEF1	1	284	0.003521127
PLPP1	1	284	0.003521127
RIOK1	2	568	0.003521127
SLAIN1	2	568	0.003521127
SLC11A2	2	568	0.003521127
THSD1	3	852	0.003521127
TLX2	1	284	0.003521127
ZDHHC24	1	284	0.003521127
ZNF675	2	568	0.003521127
ZSCAN29	3	852	0.003521127
SMG6	5	1419	0.003523608
ESF1	3	851	0.003525264
LPIN3	3	851	0.003525264
CEP350	11	3117	0.003529034
ASPDH	1	283	0.003533569
BOLL	1	283	0.003533569
OCSTAMP	2	566	0.003533569
RNF220	2	566	0.003533569
VDAC1	1	283	0.003533569
NPNT	2	565	0.003539823
PELP1	4	1130	0.003539823
CD22	3	847	0.003541913
ANTXR1	2	564	0.003546099
ATF5	1	282	0.003546099
DLGAP5	3	846	0.003546099
IGFBP7	1	282	0.003546099
KLK11	1	282	0.003546099
RAD1	1	282	0.003546099
RRP15	1	282	0.003546099
ZNF211	2	564	0.003546099
TNS2	5	1409	0.003548616
CAPRIN2	4	1127	0.003549246
CTNNBL1	2	563	0.003552398
GRK1	2	563	0.003552398
SLC22A6	2	563	0.003552398
TPP1	2	563	0.003552398
ZNF461	2	563	0.003552398
ARSH	2	562	0.003558719
AXIN2	3	843	0.003558719
C15orf41	1	281	0.003558719

FASLG	1	281	0.003558719
LBX1	1	281	0.003558719
MAPRE3	1	281	0.003558719
NRIP2	1	281	0.003558719
PGM1	2	562	0.003558719
TTC7B	3	843	0.003558719
TTLL6	3	843	0.003558719
MYOM1	6	1685	0.003560831
GCC2	6	1684	0.003562945
TMEM209	2	561	0.003565062
ZNF394	2	561	0.003565062
MAP7D1	3	841	0.003567182
ADGRA1	2	560	0.003571429
CLEC1A	1	280	0.003571429
MFSD7	2	560	0.003571429
MIF	2	560	0.003571429
MYH9	7	1960	0.003571429
NFX1	4	1120	0.003571429
RSPH3	2	560	0.003571429
TEX33	1	280	0.003571429
ZNF619	2	560	0.003571429
LRIG3	4	1119	0.00357462
SLC38A10	4	1119	0.00357462
UBAP2	4	1119	0.00357462
TLR4	3	839	0.003575685
ZKSCAN5	3	839	0.003575685
LRRC71	2	559	0.003577818
MKS1	2	559	0.003577818
NCOR2	9	2514	0.003579952
APOLD1	1	279	0.003584229
CERKL	2	558	0.003584229
FANCC	2	558	0.003584229
GJC3	1	279	0.003584229
HRASLS5	1	279	0.003584229
KCNMB3	1	279	0.003584229
MRPL46	1	279	0.003584229
PODXL	2	558	0.003584229
SEMA4B	3	837	0.003584229
TFIP11	3	837	0.003584229
ZHX2	3	837	0.003584229
EPC1	3	836	0.003588517
HNF1B	2	557	0.003590664
RALGPS1	2	557	0.003590664
CD97	3	835	0.003592814
MCPH1	3	835	0.003592814
CD19	2	556	0.003597122
CNPY3	1	278	0.003597122

DHRS4	1	278	0.003597122
5-Mar	1	278	0.003597122
MSANTD1	1	278	0.003597122
MYH15	7	1946	0.003597122
PDE3B	4	1112	0.003597122
SLC22A31	2	556	0.003597122
TTPA	1	278	0.003597122
VEPH1	3	833	0.003601441
ACOT12	2	555	0.003603604
CNKSR3	2	555	0.003603604
WDR81	7	1941	0.003606388
QRICH2	6	1663	0.003607937
CASP3	1	277	0.003610108
GUCY2F	4	1108	0.003610108
IMPA1	1	277	0.003610108
PRICKLE1	3	831	0.003610108
TMEM53	1	277	0.003610108
TMEM55B	1	277	0.003610108
TNFRSF4	1	277	0.003610108
UCK1	1	277	0.003610108
ATP5A1	2	553	0.003616637
GK2	2	553	0.003616637
HP1BP3	2	553	0.003616637
MCOLN3	2	553	0.003616637
CDH22	3	828	0.003623188
CTDSPL	1	276	0.003623188
FAM234A	2	552	0.003623188
GALNT14	2	552	0.003623188
KLK10	1	276	0.003623188
MAP3K9	4	1104	0.003623188
MGRN1	2	552	0.003623188
PPP1R13L	3	828	0.003623188
RSPH9	1	276	0.003623188
SOX21	1	276	0.003623188
STX12	1	276	0.003623188
ZNF688	1	276	0.003623188
TTC28	9	2481	0.00362757
VIL1	3	827	0.00362757
ROBO2	5	1378	0.003628447
CBS	2	551	0.003629764
FN1	9	2477	0.003633428
C17orf78	1	275	0.003636364
CENPV	1	275	0.003636364
DGCR2	2	550	0.003636364
EXOSC3	1	275	0.003636364
HOMEZ	2	550	0.003636364
PLPBP	1	275	0.003636364

TMEM45B	1	275	0.003636364
R3HDM1	4	1099	0.003639672
ADAM17	3	824	0.003640777
MTMR9	2	549	0.003642987
SLC33A1	2	549	0.003642987
ZNF266	2	549	0.003642987
ADGRE2	3	823	0.0036452
CCDC28A	1	274	0.003649635
EPS8	3	822	0.003649635
FGFR1	3	822	0.003649635
MAD2L1BP	1	274	0.003649635
NARS	2	548	0.003649635
TMEM106B	1	274	0.003649635
ZCCHC11	6	1644	0.003649635
ADAMTS17	4	1095	0.003652968
MCM6	3	821	0.00365408
BCO1	2	547	0.003656307
REC8	2	547	0.003656307
LRIG1	4	1093	0.003659652
PHKB	4	1093	0.003659652
TMF1	4	1093	0.003659652
AAAS	2	546	0.003663004
CDT1	2	546	0.003663004
DIO2	1	273	0.003663004
EGFL7	1	273	0.003663004
HMX2	1	273	0.003663004
NKX2-2	1	273	0.003663004
PDCD1LG2	1	273	0.003663004
PSMB10	1	273	0.003663004
SPSB4	1	273	0.003663004
USPL1	4	1092	0.003663004
PLA2G4D	3	818	0.003667482
STK39	2	545	0.003669725
TBX4	2	545	0.003669725
CHD7	11	2997	0.003670337
BAZ2A	7	1905	0.003674541
ABT1	1	272	0.003676471
C1orf226	1	272	0.003676471
HOXA9	1	272	0.003676471
KCNRG	1	272	0.003676471
NEUROG2	1	272	0.003676471
STK11IP	4	1088	0.003676471
ZNF891	2	544	0.003676471
ZSCAN25	2	544	0.003676471
FAT2	16	4349	0.003679007
PIK3R4	5	1358	0.003681885
FMNL2	4	1086	0.003683241

GJA10	2	543	0.003683241
GPR108	2	543	0.003683241
RETREG2	2	543	0.003683241
ZNF852	2	543	0.003683241
CDH15	3	814	0.003685504
CMYA5	15	4069	0.003686409
ICAM4	1	271	0.003690037
IL1A	1	271	0.003690037
NUBP2	1	271	0.003690037
RNF217	2	542	0.003690037
TEPP	1	271	0.003690037
TULP1	2	542	0.003690037
ARPP21	3	812	0.003694581
CHAMP1	3	812	0.003694581
DNAAF3	2	541	0.003696858
EHD4	2	541	0.003696858
FAM20A	2	541	0.003696858
RFT1	2	541	0.003696858
ECE2	3	811	0.003699137
EXOC6B	3	811	0.003699137
FCGBP	20	5405	0.003700278
ADCY7	4	1080	0.003703704
MED4	1	270	0.003703704
PPM1M	1	270	0.003703704
RIPK2	2	540	0.003703704
RPA2	1	270	0.003703704
SLBP	1	270	0.003703704
SMUG1	1	270	0.003703704
TSPAN14	1	270	0.003703704
TSPAN17	1	270	0.003703704
UBXN8	1	270	0.003703704
ZNF33A	3	810	0.003703704
ZNF519	2	540	0.003703704
ZP4	2	540	0.003703704
CNGB3	3	809	0.003708282
NSD1	10	2696	0.003709199
FAM189A1	2	539	0.003710575
NTNG1	2	539	0.003710575
RP1	8	2156	0.003710575
SACS	17	4579	0.003712601
MCM3	3	808	0.003712871
SCYL1	3	808	0.003712871
AQP1	1	269	0.003717472
C1orf74	1	269	0.003717472
CEBPD	1	269	0.003717472
CELA2A	1	269	0.003717472
CENPK	1	269	0.003717472

DOLK	2	538	0.003717472
GATAD1	1	269	0.003717472
ZNF155	2	538	0.003717472
ZNF594	3	807	0.003717472
ABI3BP	4	1075	0.00372093
ATXN2L	4	1075	0.00372093
TMEM63C	3	806	0.003722084
KIF7	5	1343	0.003723008
CPNE1	2	537	0.003724395
CPNE3	2	537	0.003724395
LGI4	2	537	0.003724395
TSC22D1	4	1073	0.003727866
EP300	9	2414	0.003728252
ACBD4	1	268	0.003731343
ANP32E	1	268	0.003731343
C21orf33	1	268	0.003731343
CAPNS1	1	268	0.003731343
DGKB	3	804	0.003731343
FANCE	2	536	0.003731343
MED8	1	268	0.003731343
RPP30	1	268	0.003731343
TSSK3	1	268	0.003731343
ZNF623	2	536	0.003731343
ZNF737	2	536	0.003731343
ZNF226	3	803	0.00373599
ANKRD31	7	1873	0.00373732
ANKRD34C	2	535	0.003738318
ITPRIPL2	2	535	0.003738318
P4HA2	2	535	0.003738318
SOCS6	2	535	0.003738318
ALDH16A1	3	802	0.003740648
RSBN1	3	802	0.003740648
WDR33	5	1336	0.003742515
APOA1	1	267	0.003745318
ARHGAP11B	1	267	0.003745318
BEND4	2	534	0.003745318
C15orf52	2	534	0.003745318
CD82	1	267	0.003745318
DPH6	1	267	0.003745318
EHD1	2	534	0.003745318
ELANE	1	267	0.003745318
LPCAT1	2	534	0.003745318
MS4A12	1	267	0.003745318
NAGS	2	534	0.003745318
RBMXL3	4	1067	0.003748828
LENG8	3	800	0.00375
NOC3L	3	800	0.00375

RPS6KC1	4	1066	0.003752345
TBC1D31	4	1066	0.003752345
ZNF846	2	533	0.003752345
C5orf42	12	3197	0.003753519
ITGB5	3	799	0.003754693
CBX2	2	532	0.003759398
CCER2	1	266	0.003759398
FAAH2	2	532	0.003759398
FMO3	2	532	0.003759398
ITGB7	3	798	0.003759398
ITM2B	1	266	0.003759398
OSR1	1	266	0.003759398
RASL12	1	266	0.003759398
REC114	1	266	0.003759398
RPL7A	1	266	0.003759398
SLC35E2	1	266	0.003759398
ZNF20	2	532	0.003759398
DNAJC21	2	531	0.003766478
NLRP2	4	1062	0.003766478
DPP10	3	796	0.003768844
SLC4A1AP	3	796	0.003768844
MGAM	7	1857	0.003769521
ZNF451	4	1061	0.003770028
ADCK1	2	530	0.003773585
ALDH18A1	3	795	0.003773585
CDX1	1	265	0.003773585
CEACAM7	1	265	0.003773585
ELF5	1	265	0.003773585
ELOVL6	1	265	0.003773585
ESPL1	8	2120	0.003773585
GPM6B	1	265	0.003773585
OLAH	1	265	0.003773585
PRR23B	1	265	0.003773585
SLC45A2	2	530	0.003773585
UROS	1	265	0.003773585
PHLPP2	5	1323	0.003779289
NOP58	2	529	0.003780718
PLXDC2	2	529	0.003780718
TYR	2	529	0.003780718
UGT2B7	2	529	0.003780718
ADGRG5	2	528	0.003787879
ANKDD1B	2	528	0.003787879
COPS7B	1	264	0.003787879
CTRL	1	264	0.003787879
LENG1	1	264	0.003787879
METTL21C	1	264	0.003787879
MYOZ2	1	264	0.003787879

NELFA	2	528	0.003787879
PDZD9	1	264	0.003787879
SPNS1	2	528	0.003787879
UGT2B4	2	528	0.003787879
ZNF626	2	528	0.003787879
USP25	4	1055	0.003791469
MFSD6	3	791	0.003792668
USP19	5	1318	0.003793627
EOGT	2	527	0.003795066
ADAM30	3	790	0.003797468
JADE2	3	790	0.003797468
NCBP1	3	790	0.003797468
SH3D19	3	790	0.003797468
CLEC16A	4	1053	0.00379867
DRGX	1	263	0.003802281
FBXW10	4	1052	0.003802281
LMAN1L	2	526	0.003802281
LRFN2	3	789	0.003802281
MTX2	1	263	0.003802281
NECAP2	1	263	0.003802281
RAET1E	1	263	0.003802281
RFPL4B	1	263	0.003802281
RIMBP2	4	1052	0.003802281
TAX1BP1	3	789	0.003802281
ITGA3	4	1051	0.003805899
NASP	3	788	0.003807107
TEPSIN	2	525	0.003809524
ARID4B	5	1312	0.003810976
PCDHB15	3	787	0.003811944
PIKFYVE	8	2098	0.003813155
SSH1	4	1049	0.003813155
SSC5D	6	1573	0.003814367
CD99L2	1	262	0.003816794
CDR1	1	262	0.003816794
COMTD1	1	262	0.003816794
CYP4F11	2	524	0.003816794
ELN	3	786	0.003816794
FITM2	1	262	0.003816794
GZMA	1	262	0.003816794
PGBD5	2	524	0.003816794
SLC1A1	2	524	0.003816794
SLC2A2	2	524	0.003816794
SPATA20	3	786	0.003816794
SPIB	1	262	0.003816794
TMIGD1	1	262	0.003816794
VANGL1	2	524	0.003816794
DYNC1LI1	2	523	0.003824092

SMG1	14	3661	0.003824092
CDH5	3	784	0.003826531
FAM186A	9	2351	0.003828158
AMIGO2	2	522	0.003831418
ANKDD1A	2	522	0.003831418
ENOPH1	1	261	0.003831418
FBXO7	2	522	0.003831418
GALNS	2	522	0.003831418
GSR	2	522	0.003831418
MEGF11	4	1044	0.003831418
RNF208	1	261	0.003831418
SNX1	2	522	0.003831418
SPR	1	261	0.003831418
UAP1	2	522	0.003831418
ZNF107	3	783	0.003831418
NLRP13	4	1043	0.003835091
ANO9	3	782	0.003836317
DNAJC16	3	782	0.003836317
SLC45A1	3	782	0.003836317
ARFGAP2	2	521	0.003838772
CORIN	4	1042	0.003838772
CYP11A1	2	521	0.003838772
ADRA1B	2	520	0.003846154
BAMBI	1	260	0.003846154
CA3	1	260	0.003846154
CACNB4	2	520	0.003846154
CACNG6	1	260	0.003846154
CCDC127	1	260	0.003846154
CFAP157	2	520	0.003846154
DNAJC9	1	260	0.003846154
HOXC9	1	260	0.003846154
IRAK1BP1	1	260	0.003846154
KRT78	2	520	0.003846154
NSA2	1	260	0.003846154
PFKFB3	2	520	0.003846154
PFKM	3	780	0.003846154
RIC8B	2	520	0.003846154
SPPL2A	2	520	0.003846154
UPK1B	1	260	0.003846154
CREB3L1	2	519	0.003853565
CYP4A11	2	519	0.003853565
DPYS	2	519	0.003853565
LAP3	2	519	0.003853565
ZNF749	3	778	0.003856041
CADPS2	5	1296	0.003858025
BARD1	3	777	0.003861004
BPGM	1	259	0.003861004

HGS	3	777	0.003861004
KCTD4	1	259	0.003861004
KLHDC7A	3	777	0.003861004
LRRC3B	1	259	0.003861004
NR3C1	3	777	0.003861004
PCGF1	1	259	0.003861004
PDZRN4	4	1036	0.003861004
RAB34	1	259	0.003861004
SERINC4	2	518	0.003861004
TNFRSF10C	1	259	0.003861004
CUBN	14	3623	0.003864201
KIAA1522	4	1035	0.003864734
USP31	4	1035	0.003864734
ANKEF1	3	776	0.003865979
RTN1	3	776	0.003865979
MEGF8	11	2845	0.003866432
ALDH2	2	517	0.003868472
CHRNG	2	517	0.003868472
GTF2F1	2	517	0.003868472
HINFP	2	517	0.003868472
NECTIN1	2	517	0.003868472
RBPJL	2	517	0.003868472
AMN1	1	258	0.003875969
CISH	1	258	0.003875969
DACT2	3	774	0.003875969
DHCR24	2	516	0.003875969
EIF3J	1	258	0.003875969
FRG1	1	258	0.003875969
KPNA7	2	516	0.003875969
RFWD3	3	774	0.003875969
RSG1	1	258	0.003875969
TMEM99	1	258	0.003875969
UPK1A	1	258	0.003875969
ARHGEF17	8	2063	0.003877848
FCRL4	2	515	0.003883495
GJA10	2	515	0.003883495
NAGPA	2	515	0.003883495
CDON	5	1287	0.003885004
PTPRZ1	9	2315	0.003887689
CFAP53	2	514	0.003891051
CNTN6	4	1028	0.003891051
CRB2	5	1285	0.003891051
FCER1A	1	257	0.003891051
FMNL3	4	1028	0.003891051
LRRC3	1	257	0.003891051
NOL7	1	257	0.003891051
OASL	2	514	0.003891051

PSD2	3	771	0.003891051
SARS	2	514	0.003891051
SLC7A14	3	771	0.003891051
YIPF5	1	257	0.003891051
MAST2	7	1798	0.003893215
APP	3	770	0.003896104
DMP1	2	513	0.003898635
KAT5	2	513	0.003898635
MMP27	2	513	0.003898635
BMS1	5	1282	0.003900156
HMGXB3	6	1538	0.00390117
ABCB1	5	1280	0.00390625
ASIC2	2	512	0.00390625
ENKUR	1	256	0.00390625
KLK15	1	256	0.00390625
PSMA8	1	256	0.00390625
RAB26	1	256	0.00390625
RGL1	3	768	0.00390625
TMEM74B	1	256	0.00390625
APC2	9	2303	0.003907946
ARHGEF10L	5	1279	0.003909304
PLCE1	9	2302	0.003909644
CDCA2	4	1023	0.003910068
CCDC125	2	511	0.003913894
ZBTB45	2	511	0.003913894
CD101	4	1021	0.003917728
SLC12A3	4	1021	0.003917728
CD3EAP	2	510	0.003921569
ETV5	2	510	0.003921569
FBXO44	1	255	0.003921569
KRTAP10-2	1	255	0.003921569
LLGL2	4	1020	0.003921569
NECTIN4	2	510	0.003921569
TNFRSF9	1	255	0.003921569
ZNF419	2	510	0.003921569
PHF3	8	2039	0.003923492
ZFHX4	14	3567	0.003924867
BEST2	2	509	0.003929273
PRIM2	2	509	0.003929273
EPPK1	20	5088	0.003930818
MPP2	3	763	0.003931848
BARX1	1	254	0.003937008
CCDC90B	1	254	0.003937008
CDC42EP3	1	254	0.003937008
CHADL	3	762	0.003937008
CREB5	2	508	0.003937008
DISP1	6	1524	0.003937008

EDC3	2	508	0.003937008
MEOX1	1	254	0.003937008
PGAM1	1	254	0.003937008
TESMIN	2	508	0.003937008
TNFSF9	1	254	0.003937008
TTI2	2	508	0.003937008
CAPG	4	1015	0.003940887
PROB1	4	1015	0.003940887
PLEKHM3	3	761	0.003942181
SEMA4A	3	761	0.003942181
ALG6	2	507	0.003944773
MEF2A	2	507	0.003944773
THUMPD3	2	507	0.003944773
TOM1L2	2	507	0.003944773
CLDND1	1	253	0.003952569
KDM4E	2	506	0.003952569
PPP1R35	1	253	0.003952569
R3HDML	1	253	0.003952569
TFPT	1	253	0.003952569
HDAC7	4	1011	0.003956479
GGCX	3	758	0.003957784
PAX7	2	505	0.003960396
WASL	2	505	0.003960396
LRP1	18	4544	0.003961268
MAPKBP1	6	1514	0.003963012
MAN2B2	4	1009	0.003964321
C1QTNF8	1	252	0.003968254
CEACAM3	1	252	0.003968254
CPXM2	3	756	0.003968254
CYP7A1	2	504	0.003968254
IKBKB	3	756	0.003968254
KLC3	2	504	0.003968254
MLH1	3	756	0.003968254
ORC6	1	252	0.003968254
PIGW	2	504	0.003968254
PSMD5	2	504	0.003968254
ZNF511	1	252	0.003968254
AUTS2	5	1259	0.003971406
PMFBP1	4	1007	0.003972195
SLC39A6	3	755	0.00397351
TRIM56	3	755	0.00397351
IRF7	2	503	0.003976143
TCP11	2	503	0.003976143
GEMIN5	6	1508	0.00397878
ANKUB1	2	502	0.003984064
ANP32B	1	251	0.003984064
C17orf82	1	251	0.003984064

CDK19	2	502	0.003984064
ERBB2	5	1255	0.003984064
FGF23	1	251	0.003984064
HOXB4	1	251	0.003984064
HPS3	4	1004	0.003984064
MTF1	3	753	0.003984064
STRN4	3	753	0.003984064
ABCB7	3	752	0.003989362
MARK4	3	752	0.003989362
MYBL1	3	752	0.003989362
NISCH	6	1504	0.003989362
TPCN2	3	752	0.003989362
APCDD1L	2	501	0.003992016
CLMN	4	1002	0.003992016
CYP8B1	2	501	0.003992016
SLC2A5	2	501	0.003992016
AOC1	3	751	0.003994674
SEMA3C	3	751	0.003994674
ATP8B1	5	1251	0.003996803
EFCAB6	6	1501	0.003997335
ARHGAP9	3	750	0.004
B3GALNT2	2	500	0.004
C1orf68	1	250	0.004
C3orf70	1	250	0.004
FAM83F	2	500	0.004
HOXB9	1	250	0.004
IGDCC4	5	1250	0.004
KRTAP9-1	1	250	0.004
MME	3	750	0.004
NPTXR	2	500	0.004
POU3F3	2	500	0.004
TCFL5	2	500	0.004
TMEM106C	1	250	0.004
BACH1	5	1249	0.004003203
APBA2	3	749	0.00400534
CLK2	2	499	0.004008016
ANGPT1	2	498	0.004016064
BPIFA2	1	249	0.004016064
FSCN3	2	498	0.004016064
NDUFV2	1	249	0.004016064
NMT2	2	498	0.004016064
QSOX1	3	747	0.004016064
SIRT1	3	747	0.004016064
SPIN4	1	249	0.004016064
UTP23	1	249	0.004016064
ZBTB47	3	747	0.004016064
ERCC6	6	1493	0.004018754

NAV2	10	2488	0.004019293
WDPCP	3	746	0.004021448
WNK4	5	1243	0.004022526
CRISPLD2	2	497	0.004024145
KCNH6	4	994	0.004024145
SLC17A4	2	497	0.004024145
IGHMBP2	4	993	0.004028197
CAMSAP2	6	1489	0.00402955
CAPNS2	1	248	0.004032258
CDH1	2	496	0.004032258
CNPY4	1	248	0.004032258
DOK3	2	496	0.004032258
PLAGL2	2	496	0.004032258
RASL11B	1	248	0.004032258
RPL7	1	248	0.004032258
SECTM1	1	248	0.004032258
SLC2A11	2	496	0.004032258
TCAIM	2	496	0.004032258
TMEM104	2	496	0.004032258
ZBTB40	5	1239	0.004035513
CUX2	6	1486	0.004037685
DTNA	3	743	0.004037685
TLE2	3	743	0.004037685
ENTPD8	2	495	0.004040404
FOXD2	2	495	0.004040404
ZNF212	2	495	0.004040404
ZMYND15	3	742	0.004043127
STOX1	4	989	0.004044489
CSMD3	15	3707	0.004046399
ATP6V1D	1	247	0.004048583
BDNF	1	247	0.004048583
C3orf80	1	247	0.004048583
COQ10A	1	247	0.004048583
FAM71E1	1	247	0.004048583
KRT12	2	494	0.004048583
NIPSNAP3B	1	247	0.004048583
ONECUT3	2	494	0.004048583
PEX11A	1	247	0.004048583
RNASEL	3	741	0.004048583
STC1	1	247	0.004048583
TAOK2	5	1235	0.004048583
TM2D3	1	247	0.004048583
ZSCAN30	2	494	0.004048583
ACVR1C	2	493	0.004056795
AMIGO1	2	493	0.004056795
LRRC14	2	493	0.004056795
PEPD	2	493	0.004056795

TMCO6	2	493	0.004056795
SLC4A3	5	1232	0.004058442
ADAM18	3	739	0.00405954
GARS	3	739	0.00405954
MYO7A	9	2215	0.004063205
ANKH	2	492	0.004065041
ATP23	1	246	0.004065041
BRMS1	1	246	0.004065041
FUT11	2	492	0.004065041
HSF4	2	492	0.004065041
LIN37	1	246	0.004065041
LYPD4	1	246	0.004065041
MRM2	1	246	0.004065041
PSMA6	1	246	0.004065041
RAET1L	1	246	0.004065041
SIPA1L2	7	1722	0.004065041
SNRNP35	1	246	0.004065041
TOM1	2	492	0.004065041
YWHAB	1	246	0.004065041
YWHAH	1	246	0.004065041
ACACB	10	2458	0.004068348
PIGG	4	983	0.004069176
PRKCE	3	737	0.004070556
CYP2B6	2	491	0.00407332
KCNS3	2	491	0.00407332
NLRP1	6	1473	0.00407332
COL7A1	12	2944	0.004076087
PDE4B	3	736	0.004076087
ZC3H14	3	736	0.004076087
MON2	7	1717	0.004076878
TDRD5	4	981	0.004077472
ACTL10	1	245	0.004081633
C1QA	1	245	0.004081633
CLEC14A	2	490	0.004081633
COG1	4	980	0.004081633
CYP2C19	2	490	0.004081633
MFSD14A	2	490	0.004081633
PEX6	4	980	0.004081633
PRRX1	1	245	0.004081633
RSL1D1	2	490	0.004081633
SP8	2	490	0.004081633
STON1	3	735	0.004081633
TEKT3	2	490	0.004081633
SNAPC4	6	1469	0.004084411
MZF1	3	734	0.004087193
PMPCB	2	489	0.00408998
ZNF71	2	489	0.00408998

PCDH15	8	1955	0.004092072
CHD1	7	1710	0.004093567
ERN1	4	977	0.004094166
ADAMTS18	5	1221	0.004095004
CREBBP	10	2442	0.004095004
ARL10	1	244	0.004098361
DCXR	1	244	0.004098361
DGKH	5	1220	0.004098361
FAM168A	1	244	0.004098361
HLX	2	488	0.004098361
IL31RA	3	732	0.004098361
KLK6	1	244	0.004098361
METTL7A	1	244	0.004098361
MMP11	2	488	0.004098361
MS4A2	1	244	0.004098361
PTGER2	2	488	0.004098361
RND3	1	244	0.004098361
YIPF4	1	244	0.004098361
PXDNL	6	1463	0.004101162
PLEKHG3	5	1219	0.004101723
CHTF18	4	975	0.004102564
GOLGA5	3	731	0.004103967
ALDH8A1	2	487	0.004106776
PTDSS2	2	487	0.004106776
SLC38A1	2	487	0.004106776
SF3B3	5	1217	0.004108463
AFAP1	3	730	0.004109589
ANAPC1	8	1944	0.004115226
C1QTNF5	1	243	0.004115226
CRISP2	1	243	0.004115226
FAM160A2	4	972	0.004115226
FGF12	1	243	0.004115226
GSTO2	1	243	0.004115226
NR1H4	2	486	0.004115226
PLEKHB1	1	243	0.004115226
RABL6	3	729	0.004115226
TMEM109	1	243	0.004115226
TRPV5	3	729	0.004115226
TRPM4	5	1214	0.004118616
FBN2	12	2912	0.004120879
HOOK1	3	728	0.004120879
VCAN	14	3396	0.004122497
ZSWIM1	2	485	0.004123711
GPD2	3	727	0.004126547
SLC6A17	3	727	0.004126547
PCSK6	4	969	0.004127967
APIP	1	242	0.004132231

BRD3	3	726	0.004132231
CTNND1	4	968	0.004132231
ENTPD6	2	484	0.004132231
IL34	1	242	0.004132231
MYF6	1	242	0.004132231
SRSF11	2	484	0.004132231
TPSD1	1	242	0.004132231
ZNF804A	5	1209	0.004135649
EXOC8	3	725	0.004137931
TIMELESS	5	1208	0.004139073
DDX49	2	483	0.004140787
PGD	2	483	0.004140787
SHMT1	2	483	0.004140787
WIPF3	2	483	0.004140787
COL4A4	7	1690	0.004142012
KDM5A	7	1690	0.004142012
CHD9	12	2897	0.004142216
DLG4	3	724	0.004143646
SLCO4C1	3	724	0.004143646
ADGRL3	6	1447	0.00414651
AK7	3	723	0.004149378
CDCA4	1	241	0.004149378
DUSP10	2	482	0.004149378
EPS8L1	3	723	0.004149378
MKRN1	2	482	0.004149378
NRIP3	1	241	0.004149378
PARD3B	5	1205	0.004149378
PDCL2	1	241	0.004149378
PHOSPHO2	1	241	0.004149378
PRSS58	1	241	0.004149378
RCAN3	1	241	0.004149378
SYNC	2	482	0.004149378
XIRP2	14	3374	0.004149378
ZNF750	3	723	0.004149378
TNS3	6	1445	0.004152249
IQSEC1	4	963	0.004153686
USP4	4	963	0.004153686
EXOC3L4	3	722	0.004155125
FLNA	11	2647	0.004155648
NOS3	5	1203	0.004156276
PTCH2	5	1203	0.004156276
CLK4	2	481	0.004158004
PUS3	2	481	0.004158004
ZNF177	2	481	0.004158004
PAN2	5	1202	0.004159734
CTSA	2	480	0.004166667
DLX4	1	240	0.004166667

DNAJA3	2	480	0.004166667
HDGF	1	240	0.004166667
KLF10	2	480	0.004166667
KRTCAP3	1	240	0.004166667
SESN2	2	480	0.004166667
SNRPN	1	240	0.004166667
THEM4	1	240	0.004166667
U2AF1	1	240	0.004166667
FAT3	19	4557	0.00416941
SLCO6A1	3	719	0.004172462
CHRNA9	2	479	0.004175365
FAM196A	2	479	0.004175365
GABRR1	2	479	0.004175365
PPM1B	2	479	0.004175365
TMEM161A	2	479	0.004175365
SAMD4A	3	718	0.004178273
ZNF28	3	718	0.004178273
ENPEP	4	957	0.004179728
CLDN14	1	239	0.0041841
DDX19A	2	478	0.0041841
DIABLO	1	239	0.0041841
IZUMO3	1	239	0.0041841
NKX2-8	1	239	0.0041841
OPN4	2	478	0.0041841
PTX4	2	478	0.0041841
SCARB2	2	478	0.0041841
THBS3	4	956	0.0041841
TMCO1	1	239	0.0041841
ZNF470	3	717	0.0041841
AK9	8	1911	0.00418629
SYDE2	5	1194	0.004187605
ADAM19	4	955	0.004188482
DRD5	2	477	0.004192872
MAZ	2	477	0.004192872
MEIS2	2	477	0.004192872
NARS2	2	477	0.004192872
OC90	2	477	0.004192872
TMCC3	2	477	0.004192872
TRMT61B	2	477	0.004192872
WWC2	5	1192	0.004194631
ALS2CL	4	953	0.004197272
NOD1	4	953	0.004197272
HDAC7	4	952	0.004201681
LRRN4CL	1	238	0.004201681
MDM1	3	714	0.004201681
SHISA3	1	238	0.004201681
TSPAN4	1	238	0.004201681

LRP10	3	713	0.004207574
ZBTB1	3	713	0.004207574
CNDP2	2	475	0.004210526
DAP	2	475	0.004210526
ERMAP	2	475	0.004210526
RRNAD1	2	475	0.004210526
RRP9	2	475	0.004210526
XRN2	4	950	0.004210526
ZMPSTE24	2	475	0.004210526
SSH2	6	1423	0.004216444
CBR4	1	237	0.004219409
DDN	3	711	0.004219409
FBXO34	3	711	0.004219409
IFIT1B	2	474	0.004219409
LYPLAL1	1	237	0.004219409
MAPK8IP1	3	711	0.004219409
NRL	1	237	0.004219409
RAB23	1	237	0.004219409
RNF166	1	237	0.004219409
TMBIM6	1	237	0.004219409
VRK3	2	474	0.004219409
ZNF141	2	474	0.004219409
ZNF230	2	474	0.004219409
VWA3A	5	1184	0.004222973
MAP3K14	4	947	0.004223865
PCDHA4	4	947	0.004223865
RNF213	22	5207	0.004225082
APBB1	3	710	0.004225352
MUM1	3	710	0.004225352
ALG10B	2	473	0.00422833
BEST4	2	473	0.00422833
KREMEN1	2	473	0.00422833
SETD6	2	473	0.00422833
ZSCAN21	2	473	0.00422833
ARHGEF16	3	709	0.004231312
DNAH9	19	4486	0.004235399
CATSPER4	2	472	0.004237288
CWC27	2	472	0.004237288
EID2	1	236	0.004237288
GAMT	1	236	0.004237288
NRXN1	2	472	0.004237288
NUDT8	1	236	0.004237288
RABL3	1	236	0.004237288
REP15	1	236	0.004237288
SRA1	1	236	0.004237288
SYCP3	1	236	0.004237288
TMEM8B	2	472	0.004237288

ZBED6CL	1	236	0.004237288
NUP210	8	1887	0.004239534
DHX33	3	707	0.004243281
MMP9	3	707	0.004243281
PRDM13	3	707	0.004243281
TMEM260	3	707	0.004243281
ITIH5	4	942	0.004246285
MMP13	2	471	0.004246285
NONO	2	471	0.004246285
SNED1	6	1413	0.004246285
ZDBF2	10	2354	0.004248088
ADD3	3	706	0.004249292
INTS13	3	706	0.004249292
MYO15A	15	3530	0.004249292
SLC44A2	3	706	0.004249292
PCDHA12	4	941	0.004250797
ATM	13	3056	0.004253927
ATP6AP1	2	470	0.004255319
BHLHA9	1	235	0.004255319
CHCHD6	1	235	0.004255319
DERL3	1	235	0.004255319
DES	2	470	0.004255319
EXOSC5	1	235	0.004255319
L3MBTL2	3	705	0.004255319
MOSPD3	1	235	0.004255319
PRSS37	1	235	0.004255319
SWI5	1	235	0.004255319
TRAF4	2	470	0.004255319
ZNHIT6	2	470	0.004255319
AP2A2	4	939	0.004259851
NLN	3	704	0.004261364
DDX42	4	938	0.004264392
KRT7	2	469	0.004264392
PELI3	2	469	0.004264392
TTC38	2	469	0.004264392
THBS2	5	1172	0.004266212
SSPO	22	5150	0.004271845
COQ6	2	468	0.004273504
MALSU1	1	234	0.004273504
MIDN	2	468	0.004273504
PCDHGA10	4	936	0.004273504
RSPO4	1	234	0.004273504
TAPBPL	2	468	0.004273504
TK1	1	234	0.004273504
TRIML1	2	468	0.004273504
UBTD2	1	234	0.004273504
ZFYVE21	1	234	0.004273504

PCDHGA11	4	935	0.004278075
MEP1B	3	701	0.004279601
STAB1	11	2570	0.004280156
TBC1D1	5	1168	0.004280822
ADHFE1	2	467	0.004282655
C6	4	934	0.004282655
GABRG3	2	467	0.004282655
GABRR3	2	467	0.004282655
KRT36	2	467	0.004282655
SIGLEC7	2	467	0.004282655
SMAD9	2	467	0.004282655
CAPN2	3	700	0.004285714
DOCK8	9	2099	0.004287756
C4orf22	1	233	0.004291845
CCDC70	1	233	0.004291845
CLUL1	2	466	0.004291845
CS	2	466	0.004291845
FAM173B	1	233	0.004291845
FUCA1	2	466	0.004291845
IQCC	2	466	0.004291845
LAPTM4A	1	233	0.004291845
TEX35	1	233	0.004291845
TMEM150B	1	233	0.004291845
TMEM40	1	233	0.004291845
TRPM5	5	1165	0.004291845
FHOD1	5	1164	0.004295533
UNC45B	4	931	0.004296455
CCNA1	2	465	0.004301075
E2F3	2	465	0.004301075
GABRG1	2	465	0.004301075
GABRR2	2	465	0.004301075
TSEN2	2	465	0.004301075
ZSCAN32	3	697	0.004304161
COL22A1	7	1626	0.004305043
ALG1	2	464	0.004310345
CD302	1	232	0.004310345
KCTD11	1	232	0.004310345
RNF125	1	232	0.004310345
SEC14L5	3	696	0.004310345
PRMT9	4	927	0.004314995
ETAA1	4	926	0.004319654
PLIN5	2	463	0.004319654
UBA3	2	463	0.004319654
ZNF486	2	463	0.004319654
ALK	7	1620	0.004320988
COL15A1	6	1388	0.004322767
RTL9	6	1388	0.004322767

CORO7	4	925	0.004324324
ENPP1	4	925	0.004324324
HEPACAM2	2	462	0.004329004
LURAP1L	1	231	0.004329004
PAFAH1B3	1	231	0.004329004
PLEKHS1	2	462	0.004329004
RAPGEF3	4	923	0.004333694
PCSK9	3	692	0.00433526
NUCB1	2	461	0.004338395
SF1	2	461	0.004338395
SMTNL2	2	461	0.004338395
TMEM2	6	1383	0.004338395
USH1G	2	461	0.004338395
COL9A1	4	921	0.004343105
MYRF	5	1151	0.004344049
ACAN	11	2530	0.004347826
FAM3A	1	230	0.004347826
HOXA7	1	230	0.004347826
LRAT	1	230	0.004347826
NMRK2	1	230	0.004347826
RNF141	1	230	0.004347826
ZMIZ2	4	920	0.004347826
TJP3	4	919	0.004352557
PTCD3	3	689	0.004354136
SP110	3	689	0.004354136
TONSL	6	1378	0.004354136
TRAPPC9	5	1148	0.004355401
C1S	3	688	0.004360465
SRRM2	12	2752	0.004360465
USP49	3	688	0.004360465
HK2	4	917	0.00436205
C9orf135	1	229	0.004366812
CCDC134	1	229	0.004366812
CLCNKB	3	687	0.004366812
GK2	2	458	0.004366812
KNOP1	2	458	0.004366812
NEFM	4	916	0.004366812
RTBDN	1	229	0.004366812
SWSAP1	1	229	0.004366812
SYNGR3	1	229	0.004366812
TMEM182	1	229	0.004366812
TPP1	2	458	0.004366812
VCPKMT	1	229	0.004366812
ZMAT4	1	229	0.004366812
NCAPG2	5	1143	0.004374453
BAG4	2	457	0.004376368
PDLIM7	2	457	0.004376368

TMPRSS5	2	457	0.004376368
CD9	1	228	0.004385965
CPSF3	3	684	0.004385965
KCNA7	2	456	0.004385965
PRKD1	4	912	0.004385965
SCFD2	3	684	0.004385965
SLC29A1	2	456	0.004385965
TGM4	3	684	0.004385965
THAP2	1	228	0.004385965
TLDC1	2	456	0.004385965
ZC2HC1C	2	456	0.004385965
ACTN4	4	911	0.004390779
COLQ	2	455	0.004395604
KRT35	2	455	0.004395604
MFSD10	2	455	0.004395604
MSH3	5	1137	0.004397537
PLEKHH1	6	1364	0.004398827
ZNF16	3	682	0.004398827
HACE1	4	909	0.00440044
GLA	1	227	0.004405286
PILRB	1	227	0.004405286
SAMD11	3	681	0.004405286
SPAG7	1	227	0.004405286
TRNP1	1	227	0.004405286
ZMYND19	1	227	0.004405286
COL6A3	14	3177	0.004406673
TACC2	13	2948	0.004409769
DYNC2H1	19	4307	0.004411423
COL10A1	3	680	0.004411765
UNKL	3	680	0.004411765
TRAPPC11	5	1133	0.004413063
AMN	2	453	0.004415011
AP4M1	2	453	0.004415011
FGG	2	453	0.004415011
GCNT4	2	453	0.004415011
SERINC1	2	453	0.004415011
NUTM1	5	1132	0.004416961
CFAP74	7	1584	0.004419192
KIAA1524	4	905	0.00441989
MAP10	4	905	0.00441989
ARL6IP6	1	226	0.004424779
CUX1	3	678	0.004424779
ETV6	2	452	0.004424779
GAS6	3	678	0.004424779
GSTK1	1	226	0.004424779
HIST1H1B	1	226	0.004424779
MYL10	1	226	0.004424779

SPCS2	1	226	0.004424779
SRRM1	4	904	0.004424779
TMEM204	1	226	0.004424779
TMEM98	1	226	0.004424779
YAE1D1	1	226	0.004424779
ABCA8	7	1581	0.004427577
HPS5	5	1129	0.004428698
FERMT1	3	677	0.004431315
GRIP1	5	1128	0.004432624
SDE2	2	451	0.00443459
FRMPD1	7	1578	0.004435995
CEP290	11	2479	0.004437273
FOCAD	8	1801	0.004441977
CLC	1	225	0.004444444
FAM189A2	2	450	0.004444444
MOB4	1	225	0.004444444
POLN	4	900	0.004444444
PRG3	1	225	0.004444444
SLC5A11	3	675	0.004444444
SOCS3	1	225	0.004444444
THYN1	1	225	0.004444444
DBF4	3	674	0.004451039
RBM6	5	1123	0.00445236
TUBA8	2	449	0.004454343
UBN2	6	1347	0.004454343
LZTS3	3	673	0.004457652
LILRB4	2	448	0.004464286
RFT1	2	448	0.004464286
SLC5A2	3	672	0.004464286
TIMP4	1	224	0.004464286
AMER2	3	671	0.004470939
SRP72	3	671	0.004470939
ZNF335	6	1342	0.004470939
C2orf54	2	447	0.004474273
CCKBR	2	447	0.004474273
MYO3B	6	1341	0.004474273
TBX20	2	447	0.004474273
SPECC1L	5	1117	0.004476276
FAM169A	3	670	0.004477612
SPATA5	4	893	0.004479283
ASTN2	6	1339	0.004480956
APCS	1	223	0.004484305
CAPN13	3	669	0.004484305
GJB7	1	223	0.004484305
IRS2	6	1338	0.004484305
NADK	2	446	0.004484305
NLRP6	4	892	0.004484305

SCNN1A	3	669	0.004484305
TFDP2	2	446	0.004484305
TMEM114	1	223	0.004484305
TSPEAR	3	669	0.004484305
ZNF3	2	446	0.004484305
BEST3	3	668	0.004491018
FAM189B	3	668	0.004491018
KAT6A	9	2004	0.004491018
FAM205A	6	1335	0.004494382
LPIN1	4	890	0.004494382
ACO1	4	889	0.004499438
ACSBG2	3	666	0.004504505
CACNG1	1	222	0.004504505
CCM2	2	444	0.004504505
CHMP3	1	222	0.004504505
CSHL1	1	222	0.004504505
GADD45GIP1	1	222	0.004504505
GSTA3	1	222	0.004504505
PACSIN1	2	444	0.004504505
PHF14	4	888	0.004504505
VWC2L	1	222	0.004504505
ZNF506	2	444	0.004504505
TTLL7	4	887	0.004509583
POU3F2	2	443	0.004514673
SORL1	10	2214	0.004516712
RBBP8NL	3	664	0.004518072
EXOSC10	4	885	0.004519774
PDE8B	4	885	0.004519774
PDGFRB	5	1106	0.004520796
ALKBH7	1	221	0.004524887
EDNRB	2	442	0.004524887
HGSNAT	3	663	0.004524887
KCNE4	1	221	0.004524887
MXD1	1	221	0.004524887
MXRA8	2	442	0.004524887
PADI4	3	663	0.004524887
RAB27A	1	221	0.004524887
RANBP6	5	1105	0.004524887
RSAD1	2	442	0.004524887
TMEM225B	1	221	0.004524887
ZNF773	2	442	0.004524887
FASTKD3	3	662	0.004531722
MX1	3	662	0.004531722
ARAP3	7	1544	0.004533679
ANKMY2	2	441	0.004535147
EVA1C	2	441	0.004535147
MROH7	6	1323	0.004535147

NDST1	4	882	0.004535147
SH3RF3	4	882	0.004535147
ABCA10	7	1543	0.004536617
CD180	3	661	0.004538578
UBASH3A	3	661	0.004538578
DDX54	4	881	0.004540295
DNAH5	21	4624	0.004541522
SIK3	6	1321	0.004542014
LAMB4	8	1761	0.004542873
C1orf228	2	440	0.004545455
CLDN6	1	220	0.004545455
CREG1	1	220	0.004545455
GAS2L2	4	880	0.004545455
GATD1	1	220	0.004545455
GPR180	2	440	0.004545455
TIMP2	1	220	0.004545455
ZMYND10	2	440	0.004545455
SYNE1	40	8797	0.004547005
SLC24A1	5	1099	0.004549591
LAMA1	14	3075	0.004552846
ARHGAP1	2	439	0.004555809
MFAP1	2	439	0.004555809
MRPS30	2	439	0.004555809
PPL	8	1756	0.004555809
SLC17A2	2	439	0.004555809
BNIP3L	1	219	0.00456621
C7orf72	2	438	0.00456621
EFCAB3	2	438	0.00456621
IL12A	1	219	0.00456621
LHFPL5	1	219	0.00456621
MAGEH1	1	219	0.00456621
RNF150	2	438	0.00456621
TMPRSS11F	2	438	0.00456621
UBE3A	4	875	0.004571429
CCDC27	3	656	0.004573171
SLC26A7	3	656	0.004573171
NUP188	8	1749	0.004574042
MAST4	12	2623	0.004574914
RGMB	2	437	0.004576659
SPAG4	2	437	0.004576659
TGOLN2	2	437	0.004576659
TMPRSS4	2	437	0.004576659
TUT1	4	874	0.004576659
ZNF491	2	437	0.004576659
ABCG2	3	655	0.004580153
FGD2	3	655	0.004580153
CACNA2D3	5	1091	0.004582951

RP1L1	11	2400	0.004583333
CNBD1	2	436	0.004587156
CNTNAP4	6	1308	0.004587156
RAB27B	1	218	0.004587156
RAB4A	1	218	0.004587156
RRAS	1	218	0.004587156
SPHK2	3	654	0.004587156
SYCE2	1	218	0.004587156
TBX6	2	436	0.004587156
PDGFRA	5	1089	0.004591368
ANKS6	4	871	0.004592423
CDK15	2	435	0.004597701
EPAS1	4	870	0.004597701
ORC5	2	435	0.004597701
OXA1L	2	435	0.004597701
PIGU	2	435	0.004597701
SDCCAG3	2	435	0.004597701
SLC38A8	2	435	0.004597701
STK40	2	435	0.004597701
ZNF432	3	652	0.004601227
ARHGEF40	7	1519	0.004608295
BLOC1S4	1	217	0.004608295
CALY	1	217	0.004608295
CGNL1	6	1302	0.004608295
CSH2	1	217	0.004608295
DUSP19	1	217	0.004608295
GH1	1	217	0.004608295
PSMC3IP	1	217	0.004608295
RIT2	1	217	0.004608295
RSRC2	2	434	0.004608295
ZCRB1	1	217	0.004608295
QSER1	8	1735	0.004610951
KIAA1549	9	1950	0.004615385
MTMR14	3	650	0.004615385
ARHGAP39	5	1083	0.004616805
KCNH3	5	1083	0.004616805
SLC12A7	5	1083	0.004616805
GREB1	9	1949	0.004617753
APOL5	2	433	0.004618938
C16orf59	2	433	0.004618938
RECQL	3	649	0.004622496
SLC28A1	3	649	0.004622496
WDR64	5	1081	0.004625347
SHANK1	10	2161	0.004627487
ALG14	1	216	0.00462963
C8orf82	1	216	0.00462963
DCAF16	1	216	0.00462963

EPRS	7	1512	0.00462963
KRT17	2	432	0.00462963
MAP3K1	7	1512	0.00462963
MOB3C	1	216	0.00462963
MRPL24	1	216	0.00462963
RAB5C	1	216	0.00462963
SUCLG2	2	432	0.00462963
TMEM80	1	216	0.00462963
ZCWPW1	3	648	0.00462963
ZNF365	1	216	0.00462963
C9orf131	5	1079	0.00463392
MALRD1	10	2156	0.004638219
NBAS	11	2371	0.004639393
FAM69B	2	431	0.004640371
FOXN2	2	431	0.004640371
FOXP3	2	431	0.004640371
PLAU	2	431	0.004640371
SP7	2	431	0.004640371
FLRT1	3	646	0.004643963
ZNF493	3	646	0.004643963
ALG1L2	1	215	0.004651163
DARS2	3	645	0.004651163
FAAP24	1	215	0.004651163
HAND1	1	215	0.004651163
HIST1H1A	1	215	0.004651163
LYSMD2	1	215	0.004651163
MMP24	3	645	0.004651163
MPZL2	1	215	0.004651163
MRPL43	1	215	0.004651163
NPFFR1	2	430	0.004651163
PDE6A	4	860	0.004651163
PER1	6	1290	0.004651163
POTEF	5	1075	0.004651163
RAB5B	1	215	0.004651163
SCN2B	1	215	0.004651163
ZFP64	3	645	0.004651163
POFUT2	2	429	0.004662005
SLC37A4	2	429	0.004662005
HEATR1	10	2144	0.004664179
HSPA6	3	643	0.00466563
MAGEC3	3	643	0.00466563
THEMIS2	3	643	0.00466563
EEF1AKMT1	1	214	0.004672897
FAM167A	1	214	0.004672897
KDELR3	1	214	0.004672897
NPM2	1	214	0.004672897
OVOL3	1	214	0.004672897

PRDX5	1	214	0.004672897
RFLNB	1	214	0.004672897
RHOJ	1	214	0.004672897
SMCO1	1	214	0.004672897
TCF23	1	214	0.004672897
TESC	1	214	0.004672897
COL17A1	7	1497	0.004676019
PRSS36	4	855	0.004678363
SPTB	10	2137	0.004679457
MAN1A2	3	641	0.004680187
DAAM2	5	1068	0.004681648
AKAP5	2	427	0.004683841
PUS1	2	427	0.004683841
RALGAPB	7	1494	0.004685408
MICAL1	5	1067	0.004686036
TENM2	13	2774	0.004686373
GBP4	3	640	0.0046875
SLCO1B7	3	640	0.0046875
KALRN	14	2986	0.004688547
DLG5	9	1919	0.004689943
CLEC4C	1	213	0.004694836
HIST1H1C	1	213	0.004694836
INSL6	1	213	0.004694836
MOSPD1	1	213	0.004694836
PSG11	2	426	0.004694836
RIF1	1	213	0.004694836
SHROOM1	4	852	0.004694836
TMEM186	1	213	0.004694836
ZFAND5	1	213	0.004694836
CDK12	7	1490	0.004697987
SPINK5	5	1064	0.004699248
RLF	9	1914	0.004702194
ABHD2	2	425	0.004705882
CWC25	2	425	0.004705882
ZNF2	2	425	0.004705882
PLEKHG5	5	1062	0.004708098
GALNT8	3	637	0.004709576
ITSN2	8	1697	0.004714202
FNDC5	1	212	0.004716981
GLB1L2	3	636	0.004716981
NOP9	3	636	0.004716981
PXMP4	1	212	0.004716981
RAB15	1	212	0.004716981
SPOCK2	2	424	0.004716981
UPF2	6	1272	0.004716981
ZNF385C	2	424	0.004716981
ZNF597	2	424	0.004716981

MAP1S	5	1059	0.004721435
MYO7B	10	2116	0.004725898
VPS13A	15	3174	0.004725898
AP1M2	2	423	0.004728132
FBXL2	2	423	0.004728132
MRPL37	2	423	0.004728132
OXER1	2	423	0.004728132
RIMS1	8	1692	0.004728132
SERINC5	2	423	0.004728132
SHF	2	423	0.004728132
GTPBP4	3	634	0.004731861
PRMT9	4	845	0.004733728
B3GALT2	2	422	0.004739336
BAK1	1	211	0.004739336
C19orf47	2	422	0.004739336
CABLES1	3	633	0.004739336
CSNK1G1	2	422	0.004739336
DUSP26	1	211	0.004739336
FIBIN	1	211	0.004739336
FKBP14	1	211	0.004739336
GSTCD	3	633	0.004739336
MCHR1	2	422	0.004739336
NBPF3	3	633	0.004739336
PDGFA	1	211	0.004739336
REXO4	2	422	0.004739336
RGS2	1	211	0.004739336
RILPL2	1	211	0.004739336
SDF2	1	211	0.004739336
UBE2Q1	2	422	0.004739336
FARP2	5	1054	0.004743833
OTUD7B	4	843	0.004744958
NUP153	7	1475	0.004745763
ARL6IP4	2	421	0.004750594
SYT12	2	421	0.004750594
TMPRSS11A	2	421	0.004750594
TRMT1	2	421	0.004750594
PABPC3	3	631	0.004754358
TEC	3	631	0.004754358
ANKRD10	2	420	0.004761905
COPZ2	1	210	0.004761905
CRELD1	2	420	0.004761905
GPLD1	4	840	0.004761905
GSTP1	1	210	0.004761905
KRTAP4-9	1	210	0.004761905
MRM3	2	420	0.004761905
NTF4	1	210	0.004761905
POLR2E	1	210	0.004761905

RGS17	1	210	0.004761905
RHOD	1	210	0.004761905
DACT3	3	629	0.004769475
TDRD6	10	2096	0.004770992
CKMT2	2	419	0.00477327
KCNAB1	2	419	0.00477327
MOK	2	419	0.00477327
TBC1D32	6	1257	0.00477327
VAT1L	2	419	0.00477327
GNPTAB	6	1256	0.00477707
LRFN3	3	628	0.00477707
CLIP2	5	1046	0.004780115
AMTN	1	209	0.004784689
C19orf68	3	627	0.004784689
CCDC22	3	627	0.004784689
DTNB	3	627	0.004784689
GLTP	1	209	0.004784689
SRP68	3	627	0.004784689
TOPORS	5	1045	0.004784689
DOPEY2	11	2298	0.004786771
IFT140	7	1462	0.004787962
FAM208A	8	1670	0.004790419
CRAT	3	626	0.004792332
MAG	3	626	0.004792332
TEC	3	626	0.004792332
WDR76	3	626	0.004792332
LMTK3	7	1460	0.004794521
KIAA1109	24	5005	0.004795205
CKMT1B	2	417	0.004796163
PVR	2	417	0.004796163
SAMD14	2	417	0.004796163
TRAPPC13	2	417	0.004796163
TSPYL5	2	417	0.004796163
ZC3H13	8	1668	0.004796163
DAGLA	5	1042	0.004798464
ZNF780B	4	833	0.004801921
APAF1	6	1248	0.004807692
CNTN2	5	1040	0.004807692
COL4A3BP	3	624	0.004807692
CRIP2	1	208	0.004807692
MED18	1	208	0.004807692
MYL6B	1	208	0.004807692
NKAIN4	1	208	0.004807692
PAPSS1	3	624	0.004807692
PRB2	2	416	0.004807692
SIDT2	4	832	0.004807692
TRAF1	2	416	0.004807692

ZG16B	1	208	0.004807692
SORT1	4	831	0.004813478
JARID2	6	1246	0.004815409
ZNF30	3	623	0.004815409
CRYBG2	8	1661	0.004816376
CD163L1	7	1453	0.004817619
CSNK1G2	2	415	0.004819277
CARD6	5	1037	0.004821601
PITPNM1	6	1244	0.004823151
SLC24A4	3	622	0.004823151
ARAP1	7	1450	0.004827586
ACOT6	1	207	0.004830918
FGF16	1	207	0.004830918
HHLA2	2	414	0.004830918
ITPK1	2	414	0.004830918
MGMT	1	207	0.004830918
SPRYD4	1	207	0.004830918
TIMP1	1	207	0.004830918
TSPYL4	2	414	0.004830918
UBE2E3	1	207	0.004830918
MYO1H	5	1032	0.004844961
PRKDC	20	4128	0.004844961
JAG2	6	1238	0.004846527
SLC27A6	3	619	0.004846527
APOPT1	1	206	0.004854369
BLVRB	1	206	0.004854369
C5orf30	1	206	0.004854369
DLL3	3	618	0.004854369
MLNR	2	412	0.004854369
MPV17L2	1	206	0.004854369
PTPRCAP	1	206	0.004854369
RAB18	1	206	0.004854369
RALA	1	206	0.004854369
TEX38	1	206	0.004854369
TPD52L2	1	206	0.004854369
NCOA1	7	1441	0.004857738
PHKA2	6	1235	0.0048583
NAT1	3	617	0.004862237
COL6A1	5	1028	0.004863813
ATAD3C	2	411	0.00486618
KDM1B	4	822	0.00486618
STAC2	2	411	0.00486618
ZNF679	2	411	0.00486618
TNFRSF11A	3	616	0.00487013
FGFR2	4	821	0.004872107
AUP1	2	410	0.004878049
CRYBB2	1	205	0.004878049

DRAP1	1	205	0.004878049
HSPB1	1	205	0.004878049
IQCF1	1	205	0.004878049
MIS12	1	205	0.004878049
NR2E3	2	410	0.004878049
RERGL	1	205	0.004878049
ZNF236	9	1845	0.004878049
PSD	5	1024	0.004882813
ADAM9	4	819	0.004884005
ARHGAP11A	5	1023	0.004887586
OGDH	5	1023	0.004887586
PPARGC1B	5	1023	0.004887586
ABHD3	2	409	0.004889976
ARMC9	4	818	0.004889976
EXOC3L2	2	409	0.004889976
KLRG2	2	409	0.004889976
LRBA	14	2863	0.004889976
GPR37	3	613	0.004893964
KCNA5	3	613	0.004893964
PODN	3	613	0.004893964
RUNDC1	3	613	0.004893964
FGD6	7	1430	0.004895105
B9D1	1	204	0.004901961
C16orf45	1	204	0.004901961
DUXA	1	204	0.004901961
GAPDHS	2	408	0.004901961
KCNK13	2	408	0.004901961
POLR3H	1	204	0.004901961
RPS5	1	204	0.004901961
TPD52L1	1	204	0.004901961
ADAMTS14	6	1223	0.004905969
RIPOR1	6	1223	0.004905969
BCL9	7	1426	0.004908836
MYO1G	5	1018	0.004911591
ARRDC2	2	407	0.004914005
ELK3	2	407	0.004914005
EVX1	2	407	0.004914005
POC1A	2	407	0.004914005
REXO1	6	1221	0.004914005
ZNF174	2	407	0.004914005
HIRA	5	1017	0.004916421
AHNAK	29	5890	0.004923599
DNAH1	21	4265	0.004923798
FLG	20	4061	0.004924895
C1orf109	1	203	0.004926108
HDGFL3	1	203	0.004926108
HS3ST3A1	2	406	0.004926108

NOP2	4	812	0.004926108
PHKG2	2	406	0.004926108
POGK	3	609	0.004926108
RASL10A	1	203	0.004926108
SERHL2	1	203	0.004926108
ZNF571	3	609	0.004926108
TAF1L	9	1826	0.004928806
DNAH17	22	4462	0.004930524
TLR10	4	811	0.004932182
GALNT11	3	608	0.004934211
TDP1	3	608	0.004934211
TRIM66	6	1216	0.004934211
ABHD1	2	405	0.004938272
BMT2	2	405	0.004938272
CTH	2	405	0.004938272
SLC35E2B	2	405	0.004938272
ZNF785	2	405	0.004938272
RPN1	3	607	0.004942339
F5	11	2224	0.004946043
BCL7B	1	202	0.004950495
IER5L	2	404	0.004950495
MXRA5	14	2828	0.004950495
PLEKHA1	2	404	0.004950495
PRRG2	1	202	0.004950495
RAVER1	3	606	0.004950495
RGS16	1	202	0.004950495
SAC3D1	2	404	0.004950495
TBL3	4	808	0.004950495
TM4SF1	1	202	0.004950495
TM4SF4	1	202	0.004950495
TWIST1	1	202	0.004950495
KIF1B	9	1816	0.004955947
MIA2	7	1412	0.004957507
PHF23	2	403	0.004962779
PLA2G6	4	806	0.004962779
ROBO4	5	1007	0.004965243
DEUP1	3	604	0.004966887
SGSM2	5	1006	0.004970179
BSPRY	2	402	0.004975124
GUCA1A	1	201	0.004975124
PSMB2	1	201	0.004975124
PTAR1	2	402	0.004975124
SNX10	1	201	0.004975124
TAGLN	1	201	0.004975124
TM4SF18	1	201	0.004975124
ZNF169	3	603	0.004975124
VWF	14	2813	0.004976893

CARD14	5	1004	0.00498008
COL11A1	9	1806	0.004983389
FNDC3B	6	1204	0.004983389
CANT1	2	401	0.004987531
GOLM1	2	401	0.004987531
MNX1	2	401	0.004987531
RILP	2	401	0.004987531
SLC35B3	2	401	0.004987531
TGIF1	2	401	0.004987531
AHDC1	8	1603	0.004990643
ESCO2	3	601	0.004991681
DDX55	3	600	0.005
IFT74	3	600	0.005
PHF24	2	400	0.005
PROZ	2	400	0.005
RCVRN	1	200	0.005
TMEM211	1	200	0.005
TMEM43	2	400	0.005
SCUBE2	5	999	0.005005005
LAMB2	9	1798	0.005005562
DACH2	3	599	0.005008347
ZNF343	3	599	0.005008347
LIPK	2	399	0.005012531
SIRT3	2	399	0.005012531
NR4A1	3	598	0.005016722
SLC6A5	4	797	0.005018821
AIP	1	199	0.005025126
BRD9	3	597	0.005025126
BSCL2	2	398	0.005025126
CADM3	2	398	0.005025126
CD69	1	199	0.005025126
CDH11	4	796	0.005025126
CRADD	1	199	0.005025126
DNAJC5B	1	199	0.005025126
HPGDS	1	199	0.005025126
NMRK1	1	199	0.005025126
PCDHB3	4	796	0.005025126
PEMT	1	199	0.005025126
PRDX1	1	199	0.005025126
SAP25	1	199	0.005025126
SRBD1	5	995	0.005025126
SRRM3	3	597	0.005025126
SSSCA1	1	199	0.005025126
TCF15	1	199	0.005025126
UNC93B1	3	597	0.005025126
SPAG5	6	1193	0.005029338
PAN2	5	994	0.005030181

SPG7	4	795	0.005031447
FEZ1	3	596	0.005033557
CEP170B	8	1589	0.005034613
MET	7	1390	0.005035971
SLC13A1	3	595	0.005042017
SNX9	3	595	0.005042017
CAD	4	793	0.005044136
APOA4	2	396	0.005050505
APOO	1	198	0.005050505
ARMC7	1	198	0.005050505
COMMD9	1	198	0.005050505
DENR	1	198	0.005050505
DHX57	7	1386	0.005050505
FAM219B	1	198	0.005050505
LBX2	1	198	0.005050505
NECAB3	2	396	0.005050505
RAB34	1	198	0.005050505
SAMD9L	8	1584	0.005050505
SAR1A	1	198	0.005050505
SOCS2	1	198	0.005050505
TNRC18	15	2968	0.005053908
ATRIP	4	791	0.00505689
H6PD	4	791	0.00505689
BIN1	3	593	0.005059022
PALB2	6	1186	0.005059022
STXBP5L	6	1186	0.005059022
DCAF4L2	2	395	0.005063291
GABPB1	2	395	0.005063291
SH3GLB2	2	395	0.005063291
ZNF200	2	395	0.005063291
ABCC11	7	1382	0.005065123
CARD16	1	197	0.005076142
CRYBA2	1	197	0.005076142
GNAS	2	394	0.005076142
GPX4	1	197	0.005076142
ITGB3	4	788	0.005076142
ITGB6	4	788	0.005076142
PAK4	3	591	0.005076142
PXN	3	591	0.005076142
UGCG	2	394	0.005076142
ZNF763	2	394	0.005076142
ZWILCH	3	591	0.005076142
TPR	12	2363	0.00507829
ZNF578	3	590	0.005084746
CRTAM	2	393	0.005089059
HPD	2	393	0.005089059
KMT5A	2	393	0.005089059

UBQLN1	3	589	0.005093379
BANK1	4	785	0.005095541
PCSK7	4	785	0.005095541
BMP15	2	392	0.005102041
CERS5	2	392	0.005102041
CHMP1A	1	196	0.005102041
CRH	1	196	0.005102041
DAB1	3	588	0.005102041
E4F1	4	784	0.005102041
EN1	2	392	0.005102041
GPR162	3	588	0.005102041
HS1BP3	2	392	0.005102041
HSPB8	1	196	0.005102041
NME8	3	588	0.005102041
NPVF	1	196	0.005102041
PAFAH2	2	392	0.005102041
PANX3	2	392	0.005102041
RBSN	4	784	0.005102041
SERPINB11	2	392	0.005102041
DAP3	5	979	0.005107252
RFX1	5	979	0.005107252
ZNF280D	5	979	0.005107252
TLN2	13	2542	0.005114083
FAM46C	2	391	0.00511509
SEMA3G	4	782	0.00511509
VPS51	4	782	0.00511509
FCRL5	5	977	0.005117707
GBP5	3	586	0.005119454
MFSD6L	3	586	0.005119454
PCNX4	6	1172	0.005119454
NEURL4	8	1562	0.005121639
CA5B	1	195	0.005128205
COMMD3	1	195	0.005128205
CYBA	1	195	0.005128205
DLC1	9	1755	0.005128205
ESAM	2	390	0.005128205
EXOSC1	1	195	0.005128205
GAD2	3	585	0.005128205
IFNW1	1	195	0.005128205
IVL	3	585	0.005128205
KRTAP4-11	1	195	0.005128205
MAFA	1	195	0.005128205
NEIL1	2	390	0.005128205
NUDT16	1	195	0.005128205
RBMXL1	2	390	0.005128205
SELENOT	1	195	0.005128205
TMEM126A	1	195	0.005128205

ZNF20	2	390	0.005128205
BICC1	5	974	0.00513347
EXOC4	5	974	0.00513347
C8A	3	584	0.005136986
PDILT	3	584	0.005136986
CBX8	2	389	0.005141388
TROAP	4	778	0.005141388
FREM3	11	2139	0.00514259
HERC1	25	4861	0.005142975
LRRK2	13	2527	0.00514444
TREH	3	583	0.005145798
TTC39C	3	583	0.005145798
ARHGEF38	4	777	0.005148005
SEMA3D	4	777	0.005148005
CSRP3	1	194	0.005154639
DESI2	1	194	0.005154639
DGAT2	2	388	0.005154639
ITPA	1	194	0.005154639
LEKR1	2	388	0.005154639
MBD3L1	1	194	0.005154639
MYH3	10	1940	0.005154639
MYL1	1	194	0.005154639
PLK4	5	970	0.005154639
RAB22A	1	194	0.005154639
TIMM22	1	194	0.005154639
TNR	7	1358	0.005154639
TSC1	6	1164	0.005154639
ZDHHC18	2	388	0.005154639
DMD	19	3685	0.005156038
GLIS3	4	775	0.00516129
HPS6	4	775	0.00516129
GALNT12	3	581	0.005163511
PYROXD2	3	581	0.005163511
SLC15A3	3	581	0.005163511
C14orf37	4	774	0.005167959
EIPR1	2	387	0.005167959
LOXL2	4	774	0.005167959
SGCA	2	387	0.005167959
SLC2A4RG	2	387	0.005167959
TTC4	2	387	0.005167959
ACSM4	3	580	0.005172414
MBD4	3	580	0.005172414
ACPP	2	386	0.005181347
C2orf80	1	193	0.005181347
CHPF2	4	772	0.005181347
ETNK2	2	386	0.005181347
IMP3	3	579	0.005181347

MFRP	3	579	0.005181347
NFE2L1	4	772	0.005181347
RPS6KA4	4	772	0.005181347
SEMA3A	4	771	0.005188067
DYTN	3	578	0.005190311
EDEM2	3	578	0.005190311
GALNT4	3	578	0.005190311
IL10RA	3	578	0.005190311
USH2A	27	5202	0.005190311
TBC1D2B	5	963	0.005192108
BSG	2	385	0.005194805
CD34	2	385	0.005194805
LRP3	4	770	0.005194805
TBX10	2	385	0.005194805
TTC14	4	770	0.005194805
ITGB2	4	769	0.00520156
RELN	18	3460	0.005202312
AAR2	2	384	0.005208333
ACKR2	2	384	0.005208333
ADAD1	3	576	0.005208333
CALR3	2	384	0.005208333
FAM102A	2	384	0.005208333
FAM169B	1	192	0.005208333
MPP2	3	576	0.005208333
MRPL11	1	192	0.005208333
OPA1	5	960	0.005208333
PMVK	1	192	0.005208333
PROKR2	2	384	0.005208333
RNF183	1	192	0.005208333
RPL9	1	192	0.005208333
S1PR4	2	384	0.005208333
SH2D3A	3	576	0.005208333
TCF7	2	384	0.005208333
UPB1	2	384	0.005208333
WFIKKN2	3	576	0.005208333
SEMA5B	6	1151	0.005212858
GOLGA1	4	767	0.005215124
TBC1D16	4	767	0.005215124
SSC4D	3	575	0.005217391
TKFC	3	575	0.005217391
ACIN1	7	1341	0.005219985
WDR55	2	383	0.005221932
FMN2	9	1722	0.005226481
LOXL1	3	574	0.005226481
PKLR	3	574	0.005226481
ADAMTS20	10	1910	0.005235602
ADAT2	1	191	0.005235602

ANKRD22	1	191	0.005235602
CLEC2D	1	191	0.005235602
LAYN	2	382	0.005235602
MAPKAPK3	2	382	0.005235602
PDCD6	1	191	0.005235602
RAB3IL1	2	382	0.005235602
RHOG	1	191	0.005235602
DYRK1A	4	763	0.005242464
BHLHE22	2	381	0.005249344
FHDC1	6	1143	0.005249344
MCM9	6	1143	0.005249344
MTERF4	2	381	0.005249344
UBAP1L	2	381	0.005249344
URB2	8	1524	0.005249344
SOS2	7	1332	0.005255255
ANKRD26	9	1710	0.005263158
C8orf76	2	380	0.005263158
CCDC74B	2	380	0.005263158
DUSP21	1	190	0.005263158
FAM159A	1	190	0.005263158
FAM184A	6	1140	0.005263158
GPX2	1	190	0.005263158
MRPS23	1	190	0.005263158
MTRF1L	2	380	0.005263158
NPHP3	7	1330	0.005263158
OPRK1	2	380	0.005263158
PIGQ	4	760	0.005263158
PTGDS	1	190	0.005263158
SEBOX	1	190	0.005263158
SLC47A1	3	570	0.005263158
WDR20	3	569	0.005272408
CCDC66	5	948	0.005274262
CACTIN	4	758	0.005277045
KCNJ10	2	379	0.005277045
ABCC4	7	1325	0.005283019
B3GNT4	2	378	0.005291005
BHLHA15	1	189	0.005291005
IFNA21	1	189	0.005291005
IFNA6	1	189	0.005291005
LARGE1	4	756	0.005291005
METTL2A	2	378	0.005291005
NEPRO	3	567	0.005291005
OAZ2	1	189	0.005291005
PARK7	1	189	0.005291005
STMN4	1	189	0.005291005
TAPT1	3	567	0.005291005
WDR17	7	1322	0.005295008

GANAB	5	944	0.00529661
PROSER1	5	944	0.00529661
NFATC1	5	943	0.005302227
DHRS13	2	377	0.00530504
LRRC36	4	754	0.00530504
PEX14	2	377	0.00530504
PIK3R6	4	754	0.00530504
RPUSD4	2	377	0.00530504
TTL	2	377	0.00530504
ZADH2	2	377	0.00530504
BIN2	3	565	0.005309735
DEAF1	3	565	0.005309735
PTPN5	3	565	0.005309735
UMODL1	7	1318	0.005311077
ARL6IP5	1	188	0.005319149
C5orf24	1	188	0.005319149
CCDC157	4	752	0.005319149
CLEC5A	1	188	0.005319149
CTSW	2	376	0.005319149
FBXO36	1	188	0.005319149
GALNT5	5	940	0.005319149
MOCS2	1	188	0.005319149
SPOUT1	2	376	0.005319149
RNF123	7	1314	0.005327245
MAP3K15	7	1313	0.005331302
ADH1C	2	375	0.005333333
CCDC142	4	750	0.005333333
NUDT19	2	375	0.005333333
PDGFRL	2	375	0.005333333
ZNF142	9	1687	0.005334914
OTOP2	3	562	0.005338078
CCDC88A	10	1871	0.005344735
PDCD11	10	1871	0.005344735
CARS	4	748	0.005347594
CENPBD1	1	187	0.005347594
CENPT	3	561	0.005347594
CGB1	1	187	0.005347594
CIB2	1	187	0.005347594
DMRT2	3	561	0.005347594
ERICH5	2	374	0.005347594
IDNK	1	187	0.005347594
KLHL1	4	748	0.005347594
LMO7	9	1683	0.005347594
P2RY11	2	374	0.005347594
RXFP4	2	374	0.005347594
EVC2	7	1308	0.005351682
MTMR12	4	747	0.005354752

CNTNAP5	7	1306	0.005359877
DMRT1	2	373	0.00536193
MECR	2	373	0.00536193
RCL1	2	373	0.00536193
SAMSN1	2	373	0.00536193
TPRA1	2	373	0.00536193
XKR9	2	373	0.00536193
DNAJC11	3	559	0.005366726
NXPE2	3	559	0.005366726
PIGN	5	931	0.005370569
FANCM	11	2048	0.005371094
DOCK6	11	2047	0.005373718
C19orf84	1	186	0.005376344
C4orf45	1	186	0.005376344
FAAH	2	372	0.005376344
FMO4	3	558	0.005376344
IFT27	1	186	0.005376344
NME6	1	186	0.005376344
OPRD1	2	372	0.005376344
PPM1K	2	372	0.005376344
RBBP9	1	186	0.005376344
RNF34	2	372	0.005376344
UBE4B	7	1302	0.005376344
SYNPO	5	929	0.005382131
ZNF536	7	1300	0.005384615
BOC	6	1114	0.005385996
TBC1D2	5	928	0.005387931
COL4A3	9	1670	0.005389222
CDCA7	2	371	0.005390836
CREB3	2	371	0.005390836
TBC1D4	7	1298	0.005392912
NUP88	4	741	0.005398111
TMTC4	4	741	0.005398111
KCTD19	5	926	0.005399568
STOX2	5	926	0.005399568
CCDC32	1	185	0.005405405
FLVCR1	3	555	0.005405405
GPC6	3	555	0.005405405
HAO1	2	370	0.005405405
HMGCLL1	2	370	0.005405405
NAALADL1	4	740	0.005405405
PABPC4L	2	370	0.005405405
PIGS	3	555	0.005405405
PPID	2	370	0.005405405
RABAC1	1	185	0.005405405
RLN1	1	185	0.005405405
TMEM140	1	185	0.005405405

TRAM2	2	370	0.005405405
MYO6	7	1294	0.005409583
CAPN11	4	739	0.00541272
SLC26A2	4	739	0.00541272
HSD3B7	2	369	0.005420054
FAT4	27	4981	0.005420598
GRK7	3	553	0.005424955
DNER	4	737	0.005427408
ESYT2	5	921	0.005428882
BMF	1	184	0.005434783
BTBD11	6	1104	0.005434783
DUSP22	1	184	0.005434783
ESM1	1	184	0.005434783
ESYT1	6	1104	0.005434783
MAP3K6	7	1288	0.005434783
P3H1	4	736	0.005434783
RAP1A	1	184	0.005434783
TMEM26	2	368	0.005434783
TNFRSF17	1	184	0.005434783
LAMA2	17	3122	0.005445227
CDK5R2	2	367	0.005449591
CPXM1	4	734	0.005449591
CTNS	2	367	0.005449591
LRRC28	2	367	0.005449591
SMTN	5	917	0.005452563
SLC22A11	3	550	0.005454545
CDH4	5	916	0.005458515
C1orf105	1	183	0.005464481
DEFB129	1	183	0.005464481
EMC4	1	183	0.005464481
FASTK	3	549	0.005464481
FATE1	1	183	0.005464481
METTL24	2	366	0.005464481
MOBP	1	183	0.005464481
RAP2A	1	183	0.005464481
TMEM154	1	183	0.005464481
UBE2M	1	183	0.005464481
MGAT4B	3	548	0.005474453
APOB	25	4563	0.005478852
GPR68	2	365	0.005479452
SYTL5	4	730	0.005479452
VSX1	2	365	0.005479452
AP3B1	6	1094	0.005484461
CEP128	6	1094	0.005484461
DDX56	3	547	0.005484461
ZNF778	4	729	0.005486968
SH3PXD2B	5	911	0.005488474

ALDOA	2	364	0.005494505
CAVIN4	2	364	0.005494505
CMTM3	1	182	0.005494505
CRYGN	1	182	0.005494505
DCDC2C	2	364	0.005494505
DCTN5	1	182	0.005494505
ILDR1	3	546	0.005494505
MYBPC3	7	1274	0.005494505
NAP1L5	1	182	0.005494505
NKX2-3	2	364	0.005494505
RHBDD2	2	364	0.005494505
SYMPK	7	1274	0.005494505
NUP98	10	1817	0.005503577
KCNV2	3	545	0.005504587
SPATS2	3	545	0.005504587
SUOX	3	545	0.005504587
BCR	7	1271	0.005507474
SCAF8	7	1271	0.005507474
AGTR2	2	363	0.005509642
PIGO	6	1089	0.005509642
CYP2U1	3	544	0.005514706
LPCAT2	3	544	0.005514706
MKL2	6	1088	0.005514706
CARF	4	725	0.005517241
AHNAK2	32	5795	0.005522002
ABHD12B	2	362	0.005524862
ACKR3	2	362	0.005524862
ACRBP	3	543	0.005524862
ARF1	1	181	0.005524862
CALML6	1	181	0.005524862
DDX4	4	724	0.005524862
GRAMD1A	4	724	0.005524862
HHIPL2	4	724	0.005524862
HPSE	3	543	0.005524862
NAA30	2	362	0.005524862
NBL1	1	181	0.005524862
OR5AU1	2	362	0.005524862
PDAP1	1	181	0.005524862
SARM1	4	724	0.005524862
TRAPPC3L	1	181	0.005524862
MKI67	18	3256	0.005528256
MTBP	5	904	0.005530973
TARS	4	723	0.005532503
ZNF280A	3	542	0.005535055
CNTROB	5	903	0.005537099
ANKRD16	2	361	0.005540166
QPCT	2	361	0.005540166

2-Sep	2	361	0.005540166
ZNF600	4	722	0.005540166
SIPA1L1	10	1804	0.005543237
SLC1A5	3	541	0.005545287
SNX27	3	541	0.005545287
ACTN3	5	901	0.00554939
COL12A1	17	3063	0.005550114
TSHZ3	6	1081	0.005550416
AMZ2	2	360	0.005555556
ANKRD2	2	360	0.005555556
APRT	1	180	0.005555556
BAALC	1	180	0.005555556
CNR2	2	360	0.005555556
COL23A1	3	540	0.005555556
DDX28	3	540	0.005555556
GCG	1	180	0.005555556
GMPPB	2	360	0.005555556
HAPLN3	2	360	0.005555556
IL20	1	180	0.005555556
MRPL18	1	180	0.005555556
MRPS22	2	360	0.005555556
NIP7	1	180	0.005555556
PPP1R7	2	360	0.005555556
SPANXN2	1	180	0.005555556
TEX37	1	180	0.005555556
ABCA6	9	1617	0.005565863
FBXL6	3	539	0.005565863
CLCN2	5	898	0.005567929
COL14A1	10	1796	0.005567929
CCNYL1	2	359	0.005571031
MAD1L1	4	718	0.005571031
NAAA	2	359	0.005571031
NANS	2	359	0.005571031
PTGFR	2	359	0.005571031
SLC5A3	4	718	0.005571031
DSP	16	2871	0.005572971
LUZP1	6	1076	0.005576208
NAPRT	3	538	0.005576208
SPATA18	3	538	0.005576208
PER2	7	1255	0.005577689
COL13A1	4	717	0.005578801
PNN	4	717	0.005578801
CDKL1	2	358	0.005586592
FYN	3	537	0.005586592
LTB4R2	2	358	0.005586592
PDCD2L	2	358	0.005586592
PPP1R3G	2	358	0.005586592

PTGER2	2	358	0.005586592
PTPA	2	358	0.005586592
SEMA5A	6	1074	0.005586592
SLC9A3R1	2	358	0.005586592
ZNF835	3	537	0.005586592
LAMB2	9	1609	0.005593536
TMEM131L	9	1609	0.005593536
CCDC13	4	715	0.005594406
CCDC170	4	715	0.005594406
TNS4	4	715	0.005594406
ZDHHC5	4	715	0.005594406
WDR34	3	536	0.005597015
TBC1D9B	7	1250	0.0056
AUNIP	2	357	0.005602241
BORCS6	2	357	0.005602241
NOV	2	357	0.005602241
WNT9B	2	357	0.005602241
APTX	2	356	0.005617978
DCTD	1	178	0.005617978
EDN2	1	178	0.005617978
GCSAM	1	178	0.005617978
GPR32	2	356	0.005617978
HCN1	5	890	0.005617978
HOXB2	2	356	0.005617978
INO80B	2	356	0.005617978
KLLN	1	178	0.005617978
LMAN2	2	356	0.005617978
NOP16	1	178	0.005617978
PRR19	2	356	0.005617978
TMEM196	1	178	0.005617978
ADAMTS8	5	889	0.005624297
MST1	4	711	0.005625879
ORC3	4	711	0.005625879
KLHL33	3	533	0.005628518
NETO1	3	533	0.005628518
CCR3	2	355	0.005633803
OR1I1	2	355	0.005633803
SIRT6	2	355	0.005633803
SPSB3	2	355	0.005633803
VWA5B2	7	1242	0.005636071
ARG2	2	354	0.005649718
CA12	2	354	0.005649718
CD68	2	354	0.005649718
COPZ1	1	177	0.005649718
CYP27A1	3	531	0.005649718
DYDC1	1	177	0.005649718
ERFE	2	354	0.005649718

FANCD2OS	1	177	0.005649718
IL19	1	177	0.005649718
PLAC8L1	1	177	0.005649718
PPIH	1	177	0.005649718
RCBTB1	3	531	0.005649718
ERCC6	6	1061	0.005655042
CCDC177	4	707	0.005657709
CLPB	4	707	0.005657709
ZCCHC8	4	707	0.005657709
ZNF680	3	530	0.005660377
NADSYN1	4	706	0.005665722
SSUH2	2	353	0.005665722
TIMM50	2	353	0.005665722
USP40	7	1235	0.005668016
RIMS2	8	1411	0.005669738
DEPDC1B	3	529	0.005671078
MYH6	11	1939	0.005673027
PKP2	5	881	0.005675369
AGMAT	2	352	0.005681818
C5orf47	1	176	0.005681818
CLIC6	4	704	0.005681818
IL20	1	176	0.005681818
LCN1	1	176	0.005681818
LCN9	1	176	0.005681818
MAJIN	1	176	0.005681818
MCEE	1	176	0.005681818
MDP1	1	176	0.005681818
NDRG4	2	352	0.005681818
OTULIN	2	352	0.005681818
RPL18A	1	176	0.005681818
TMEM95	1	176	0.005681818
OSBPL5	5	879	0.005688282
POLRMT	7	1230	0.005691057
RGPD2	10	1756	0.005694761
ROM1	2	351	0.005698006
RPUSD3	2	351	0.005698006
MBTPS1	6	1052	0.005703422
DHX38	7	1227	0.005704971
NCAPD2	8	1401	0.005710207
BTBD2	3	525	0.005714286
CYP4V2	3	525	0.005714286
DLX6	1	175	0.005714286
FPR1	2	350	0.005714286
IKBIP	2	350	0.005714286
IMMP2L	1	175	0.005714286
INHBE	2	350	0.005714286
IQGAP2	9	1575	0.005714286

NDUFAF4	1	175	0.005714286
OGFOD2	2	350	0.005714286
PMPCA	3	525	0.005714286
REG3A	1	175	0.005714286
REG3G	1	175	0.005714286
ECM2	4	699	0.005722461
CYP4F12	3	524	0.005725191
TXNRD2	3	524	0.005725191
ZNF217	6	1048	0.005725191
CRYZL1	2	349	0.005730659
QSOX2	4	698	0.005730659
SIGLEC11	4	698	0.005730659
ST18	6	1047	0.005730659
SUPT16H	6	1047	0.005730659
KIAA0232	8	1395	0.005734767
KRT71	3	523	0.005736138
FAM129C	4	697	0.005738881
ZBTB24	4	697	0.005738881
ELMSAN1	6	1045	0.005741627
AKAP7	2	348	0.005747126
CRYGC	1	174	0.005747126
CRYGD	1	174	0.005747126
DNAJB5	2	348	0.005747126
DNPH1	1	174	0.005747126
GALE	2	348	0.005747126
HMX1	2	348	0.005747126
KRTAP1-5	1	174	0.005747126
NPFFR2	3	522	0.005747126
OCLN	3	522	0.005747126
OR4K15	2	348	0.005747126
RSPH14	2	348	0.005747126
UBL4B	1	174	0.005747126
WRB	1	174	0.005747126
CTC1	7	1217	0.005751849
GSE1	7	1217	0.005751849
MUSK	5	869	0.00575374
GAS2L3	4	694	0.005763689
SCAMP3	2	347	0.005763689
ARMC2	5	867	0.005767013
IMP3	3	520	0.005769231
KIAA0895	3	520	0.005769231
OXCT1	3	520	0.005769231
SAMD3	3	520	0.005769231
GMPS	4	693	0.005772006
CABP5	1	173	0.005780347
CALHM1	2	346	0.005780347
CDC20B	3	519	0.005780347

CMTM8	1	173	0.005780347
9-Mar	2	346	0.005780347
MFAP5	1	173	0.005780347
MRPS25	1	173	0.005780347
OR13D1	2	346	0.005780347
RAX	2	346	0.005780347
RIOK3	3	519	0.005780347
SFRP4	2	346	0.005780347
TBCC	2	346	0.005780347
TTC31	3	519	0.005780347
PPP1R37	4	691	0.005788712
TRAF3IP1	4	691	0.005788712
EPHA6	6	1036	0.005791506
INTS14	3	518	0.005791506
MFSD4B	3	518	0.005791506
UTP15	3	518	0.005791506
ANKRD60	2	345	0.005797101
ATP4A	6	1035	0.005797101
CAPN9	4	690	0.005797101
GAS1	2	345	0.005797101
OBSL1	11	1896	0.005801688
TSHZ2	6	1034	0.005802708
ATXN1L	4	689	0.005805515
C10orf76	4	689	0.005805515
COL9A2	4	689	0.005805515
PLXNA2	11	1894	0.005807814
ARFGAP1	3	516	0.005813953
C1orf186	1	172	0.005813953
MRAP	1	172	0.005813953
MYL12B	1	172	0.005813953
NDUFA8	1	172	0.005813953
PRICKLE4	2	344	0.005813953
SCEL	4	688	0.005813953
SEC24D	6	1032	0.005813953
TMEM91	1	172	0.005813953
VOPP1	1	172	0.005813953
ZDHHC4	2	344	0.005813953
ZNF580	1	172	0.005813953
DNAH10	26	4471	0.005815254
EPG5	15	2579	0.005816208
MAP3K5	8	1374	0.005822416
SDAD1	4	687	0.005822416
CTU2	3	515	0.005825243
KIAA0141	3	515	0.005825243
ASPRV1	2	343	0.005830904
HOXD13	2	343	0.005830904
OR6K6	2	343	0.005830904

PPM1H	3	514	0.005836576
TMEM117	3	514	0.005836576
COX4I2	1	171	0.005847953
ETV2	2	342	0.005847953
FAM209B	1	171	0.005847953
FUT7	2	342	0.005847953
HBS1L	4	684	0.005847953
KLF4	3	513	0.005847953
NEB	39	6669	0.005847953
PTAFR	2	342	0.005847953
SLC39A11	2	342	0.005847953
TKT	5	855	0.005847953
TTC9C	1	171	0.005847953
ESPN	5	854	0.005854801
LRCH4	4	683	0.005856515
CCDC61	3	512	0.005859375
POP1	6	1024	0.005859375
CHST13	2	341	0.005865103
DDO	2	341	0.005865103
DNAJC22	2	341	0.005865103
MACC1	5	852	0.005868545
MLXIPL	5	852	0.005868545
ATP6V1B2	3	511	0.005870841
CYP4B1	3	511	0.005870841
KRT72	3	511	0.005870841
RTN4	7	1192	0.005872483
SDK1	13	2213	0.005874379
STAT2	5	851	0.005875441
A4GNT	2	340	0.005882353
ATN1	7	1190	0.005882353
SFXN5	2	340	0.005882353
SPATC1L	2	340	0.005882353
TPPP2	1	170	0.005882353
TSPO2	1	170	0.005882353
PPP4R3B	5	849	0.005889282
DLC1	9	1528	0.005890052
ITGA11	7	1188	0.005892256
TCP11L1	3	509	0.00589391
USP34	6	1018	0.00589391
SLCO5A1	5	848	0.005896226
AUH	2	339	0.005899705
FAN1	6	1017	0.005899705
FNBP4	6	1017	0.005899705
NCF4	2	339	0.005899705
SNRNP48	2	339	0.005899705
FASTKD1	5	847	0.005903188
FAM217A	3	508	0.005905512

JMJD1C	15	2540	0.005905512
TMEM102	3	508	0.005905512
ZSWIM5	7	1185	0.005907173
BRF1	4	677	0.005908419
CDHR5	5	845	0.00591716
CMTM1	1	169	0.00591716
KANK1	8	1352	0.00591716
NEBL	6	1014	0.00591716
SNX24	1	169	0.00591716
UROC1	4	676	0.00591716
ARHGEF33	5	844	0.005924171
ARHGEF37	4	675	0.005925926
RREB1	10	1687	0.005927682
PITPNM2	8	1349	0.005930319
C7	5	843	0.005931198
CSNK1A1L	2	337	0.005934718
GIMAP2	2	337	0.005934718
1-Mar	2	337	0.005934718
RASAL3	6	1011	0.005934718
NBEAL1	16	2694	0.005939124
SNTA1	3	505	0.005940594
KIF2B	4	673	0.005943536
TTLL10	4	673	0.005943536
PTK2B	6	1009	0.005946482
ATP6V0A4	5	840	0.005952381
CD226	2	336	0.005952381
DESI1	1	168	0.005952381
GREM2	1	168	0.005952381
GRTP1	2	336	0.005952381
KCNK1	2	336	0.005952381
LARS	7	1176	0.005952381
MFSD2B	3	504	0.005952381
NAT1	3	504	0.005952381
PLK5	2	336	0.005952381
PNPLA2	3	504	0.005952381
SALL2	6	1007	0.005958292
CUL9	15	2517	0.005959476
VPS16	5	839	0.005959476
PTPN21	7	1174	0.005962521
CYP3A4	3	503	0.005964215
DSCAM	12	2012	0.005964215
ZFPM1	6	1006	0.005964215
DDX18	4	670	0.005970149
DECR1	2	335	0.005970149
GPR119	2	335	0.005970149
OSGEP	2	335	0.005970149
PRAM1	4	670	0.005970149

PSG11	2	335	0.005970149
TELO2	5	837	0.005973716
P4HTM	3	502	0.005976096
SGSH	3	502	0.005976096
COL5A1	11	1838	0.005984766
C17orf74	3	501	0.005988024
C19orf57	4	668	0.005988024
CLDND2	1	167	0.005988024
CLECL1	1	167	0.005988024
IRX3	3	501	0.005988024
NRBP2	3	501	0.005988024
PIP5K1C	4	668	0.005988024
TYW1B	4	668	0.005988024
YBEY	1	167	0.005988024
CRISPLD1	3	500	0.006
PTGIS	3	500	0.006
XDH	8	1333	0.0060015
C11orf42	2	333	0.006006006
CD1B	2	333	0.006006006
FAXDC2	2	333	0.006006006
NDUFAF6	2	333	0.006006006
NKX3-2	2	333	0.006006006
ST6GALNAC6	2	333	0.006006006
TNK1	4	666	0.006006006
ZNF844	4	666	0.006006006
PTPRB	12	1997	0.006009014
CDC20	3	499	0.006012024
MYT1	3	499	0.006012024
PKMYT1	3	499	0.006012024
SIGLEC8	3	499	0.006012024
DUSP16	4	665	0.006015038
KIF22	4	665	0.006015038
CDKN2D	1	166	0.006024096
CGRRF1	2	332	0.006024096
FERD3L	1	166	0.006024096
LDHAL6A	2	332	0.006024096
MC4R	2	332	0.006024096
MTFP1	1	166	0.006024096
OPTC	2	332	0.006024096
PTGES3L	1	166	0.006024096
TXN2	1	166	0.006024096
C1orf106	4	663	0.006033183
ERICH6	4	663	0.006033183
SYBU	4	663	0.006033183
PTPN13	15	2485	0.006036217
TRMT6	3	497	0.006036217
HEPHL1	7	1159	0.006039689

AKR7A3	2	331	0.006042296
CCDC189	2	331	0.006042296
DOK6	2	331	0.006042296
JSRP1	2	331	0.006042296
RFNG	2	331	0.006042296
UBXN2B	2	331	0.006042296
CDK16	3	496	0.006048387
EVC	6	992	0.006048387
SMAD6	3	496	0.006048387
SNX19	6	992	0.006048387
ASAH2B	1	165	0.006060606
BCAR3	5	825	0.006060606
CACNB2	4	660	0.006060606
CGB7	1	165	0.006060606
CNTD1	2	330	0.006060606
EEF2KMT	2	330	0.006060606
FKRP	3	495	0.006060606
FOXS1	2	330	0.006060606
LMO4	1	165	0.006060606
NKG7	1	165	0.006060606
NPW	1	165	0.006060606
SCMH1	4	660	0.006060606
TMEM128	1	165	0.006060606
ZSCAN5B	3	495	0.006060606
TRIP11	12	1979	0.006063669
MAP1A	17	2803	0.00606493
ZNF853	4	659	0.006069803
CLCN1	6	988	0.006072874
POLM	3	494	0.006072874
DNAH3	25	4116	0.006073858
MAGI3	9	1481	0.006076975
DNTTIP1	2	329	0.006079027
LSG1	4	658	0.006079027
OR52L1	2	329	0.006079027
PTGR1	2	329	0.006079027
SCAMP2	2	329	0.006079027
TECTB	2	329	0.006079027
ZNF135	4	658	0.006079027
CFTR	9	1480	0.006081081
XPO4	7	1151	0.006081668
ZFPM2	7	1151	0.006081668
CABYR	3	493	0.006085193
MYH11	12	1972	0.006085193
TRIM35	3	493	0.006085193
GALNT7	4	657	0.00608828
SYNRG	8	1314	0.00608828
BIVM-ERCC5	10	1640	0.006097561

CA10	2	328	0.006097561
CDKN1A	1	164	0.006097561
GNRHR	2	328	0.006097561
RNASEH2C	1	164	0.006097561
RNF175	2	328	0.006097561
SESN1	3	492	0.006097561
SOHLH1	2	328	0.006097561
TEX19	1	164	0.006097561
SREBF1	7	1147	0.006102877
SLC4A9	6	983	0.006103764
UBQLN3	4	655	0.00610687
PPP1R12B	6	982	0.00610998
CTH	2	327	0.006116208
COL6A5	16	2615	0.006118547
SPEG	20	3267	0.006121824
IDUA	4	653	0.006125574
NEK9	6	979	0.006128703
CCDC40	7	1142	0.006129597
C1orf167	9	1468	0.00613079
ALX1	2	326	0.006134969
C6orf222	4	652	0.006134969
CDK6	2	326	0.006134969
FBXL12	2	326	0.006134969
FOSL2	2	326	0.006134969
HINT2	1	163	0.006134969
LCN6	1	163	0.006134969
MLST8	2	326	0.006134969
NTN5	3	489	0.006134969
RARRES2	1	163	0.006134969
TMEM251	1	163	0.006134969
VKORC1	1	163	0.006134969
TENM4	17	2769	0.006139401
RBL2	7	1139	0.006145742
NUMA1	13	2115	0.006146572
DGAT1	3	488	0.006147541
STEAP3	3	488	0.006147541
MUC6	15	2439	0.006150062
SNX29	5	813	0.006150062
APLP1	4	650	0.006153846
FOXB1	2	325	0.006153846
MAS1	2	325	0.006153846
OR1S1	2	325	0.006153846
OR5H6	2	325	0.006153846
OR6Y1	2	325	0.006153846
OTP	2	325	0.006153846
RNPEP	4	650	0.006153846
SYNE3	6	975	0.006153846

STK4	3	487	0.006160164
TMEM161B	3	487	0.006160164
URB1	14	2271	0.006164685
PIWIL2	6	973	0.006166495
C3orf18	1	162	0.00617284
FNDC8	2	324	0.00617284
HCLS1	3	486	0.00617284
PACSIN2	3	486	0.00617284
PPP2R5A	3	486	0.00617284
ZNF43	5	809	0.00618047
NLE1	3	485	0.006185567
SLC30A10	3	485	0.006185567
DEGS2	2	323	0.00619195
TAS2R40	2	323	0.00619195
MYH8	12	1937	0.006195147
TMEM63A	5	807	0.006195787
BPIFB1	3	484	0.006198347
NEU4	3	484	0.006198347
THNSL2	3	484	0.006198347
DNAH11	28	4516	0.006200177
WDHD1	7	1129	0.006200177
TP53BP2	7	1128	0.006205674
CXXC5	2	322	0.00621118
LAMTOR1	1	161	0.00621118
LYVE1	2	322	0.00621118
MRPL30	1	161	0.00621118
PNISR	5	805	0.00621118
RBKS	2	322	0.00621118
SH3TC2	8	1288	0.00621118
TAF12	1	161	0.00621118
THY1	1	161	0.00621118
TIFAB	1	161	0.00621118
ZNF74	4	644	0.00621118
IFIT5	3	482	0.006224066
MICAL2	7	1124	0.006227758
CD109	9	1445	0.006228374
ATOH8	2	321	0.00623053
AFG1L	3	481	0.006237006
HYAL4	3	481	0.006237006
OTUD1	3	481	0.006237006
ZNF780A	4	641	0.00624025
AAK1	6	961	0.006243496
THBS4	6	961	0.006243496
ACSM6	3	480	0.00625
ANXA5	2	320	0.00625
BIK	1	160	0.00625
FAM159B	1	160	0.00625

MYB	4	640	0.00625
OR2T12	2	320	0.00625
OR2T33	2	320	0.00625
OR52H1	2	320	0.00625
OR5C1	2	320	0.00625
RBM42	3	480	0.00625
SFT2D2	1	160	0.00625
TTLL11	5	800	0.00625
UMPS	3	480	0.00625
ZBBX	5	800	0.00625
ZNF330	2	320	0.00625
CASZ1	11	1759	0.006253553
DSG2	7	1118	0.006261181
CXorf38	2	319	0.006269592
FOXE3	2	319	0.006269592
OR51F1	2	319	0.006269592
OR56B4	2	319	0.006269592
SPRY1	2	319	0.006269592
ADAMTS12	10	1594	0.006273526
GRIK4	6	956	0.006276151
CACHD1	8	1274	0.006279435
IRF2BPL	5	796	0.006281407
UNC13B	10	1591	0.006285355
AGTRAP	1	159	0.006289308
C5orf52	1	159	0.006289308
C8orf44	1	159	0.006289308
HAP1	2	318	0.006289308
HSPB9	1	159	0.006289308
OR52D1	2	318	0.006289308
SCOC	1	159	0.006289308
TMEM42	1	159	0.006289308
TMEM89	1	159	0.006289308
TRIM17	3	477	0.006289308
TJP1	11	1748	0.006292906
PEG3	10	1588	0.006297229
VWA8	12	1905	0.006299213
CPE	3	476	0.006302521
RAB3IP	3	476	0.006302521
TRIP6	3	476	0.006302521
SOWAHB	5	793	0.00630517
NPAT	9	1427	0.006306938
OR6N2	2	317	0.006309148
PHACTR2	4	634	0.006309148
RNF207	4	634	0.006309148
ZNF771	2	317	0.006309148
ADAMTS15	6	950	0.006315789
PARS2	3	475	0.006315789

PCDHA1	6	950	0.006315789
SLC46A2	3	475	0.006315789
TMEM44	3	475	0.006315789
PCDHA11	6	949	0.006322445
MTR	8	1265	0.006324111
PLCG2	8	1265	0.006324111
ACP1	1	158	0.006329114
AKR1B1	2	316	0.006329114
AKR1B10	2	316	0.006329114
ANXA13	2	316	0.006329114
C16orf95	1	158	0.006329114
CDK18	3	474	0.006329114
CLEC10A	2	316	0.006329114
CYS1	1	158	0.006329114
DBNDD1	1	158	0.006329114
FAIM2	2	316	0.006329114
JMJD7	2	316	0.006329114
MRPL50	1	158	0.006329114
MZT2A	1	158	0.006329114
MZT2B	1	158	0.006329114
NUDT6	2	316	0.006329114
OR1K1	2	316	0.006329114
OR5K2	2	316	0.006329114
RACGAP1	4	632	0.006329114
TRPV3	5	790	0.006329114
CDK5RAP2	12	1893	0.006339144
SPAG16	4	631	0.006339144
SPTLC1	3	473	0.006342495
OR51L1	2	315	0.006349206
PRDM5	4	630	0.006349206
SLC25A18	2	315	0.006349206
SPRY2	2	315	0.006349206
CD36	3	472	0.006355932
CPQ	3	472	0.006355932
DMRT3	3	472	0.006355932
FOXA1	3	472	0.006355932
CNTN5	7	1100	0.006363636
SEC16A	15	2357	0.006364022
DCHS1	21	3298	0.006367495
EMP1	1	157	0.006369427
FAM218A	1	157	0.006369427
HS6ST3	3	471	0.006369427
OR10A3	2	314	0.006369427
OR52B4	2	314	0.006369427
OR52K1	2	314	0.006369427
OR5B17	2	314	0.006369427
OR5H2	2	314	0.006369427

OR8D4	2	314	0.006369427
OR9Q2	2	314	0.006369427
SIRT4	2	314	0.006369427
TRIM60	3	471	0.006369427
ZNF256	4	627	0.006379585
ZNF805	4	627	0.006379585
PNPT1	5	783	0.006385696
FCN2	2	313	0.006389776
OR10K1	2	313	0.006389776
OR1J4	2	313	0.006389776
OR2B6	2	313	0.006389776
OR5H1	2	313	0.006389776
OR5H15	2	313	0.006389776
OR6M1	2	313	0.006389776
OR8B2	2	313	0.006389776
RAX	2	313	0.006389776
TEX264	2	313	0.006389776
ZFR2	6	939	0.006389776
CYP39A1	3	469	0.006396588
MINDY1	3	469	0.006396588
PRAG1	9	1406	0.006401138
NLRP14	7	1093	0.006404392
POM121	8	1249	0.006405124
DEFB125	1	156	0.006410256
DHRS7C	2	312	0.006410256
ERICH2	1	156	0.006410256
FDXACB1	4	624	0.006410256
MTX3	2	312	0.006410256
OR51B5	2	312	0.006410256
OR5J2	2	312	0.006410256
OR6C70	2	312	0.006410256
OR6N1	2	312	0.006410256
TAS2R9	2	312	0.006410256
TEX13B	2	312	0.006410256
TMIE	1	156	0.006410256
TSR3	2	312	0.006410256
CIT	13	2027	0.006413419
GFM2	5	779	0.006418485
VEZT	5	779	0.006418485
C11orf16	3	467	0.006423983
MSH2	6	934	0.006423983
TINAGL1	3	467	0.006423983
MATN4	4	622	0.006430868
MRPL4	2	311	0.006430868
OR2M4	2	311	0.006430868
OR2Y1	2	311	0.006430868
OR4K1	2	311	0.006430868

OR52J3	2	311	0.006430868
OR8B8	2	311	0.006430868
PCF11	10	1555	0.006430868
PIM2	2	311	0.006430868
RDH8	2	311	0.006430868
SLC25A48	2	311	0.006430868
VSIR	2	311	0.006430868
ZDHHC13	4	622	0.006430868
MYZAP	3	466	0.006437768
PCDHGA12	6	932	0.006437768
PCDHGA6	6	932	0.006437768
C10orf113	1	155	0.006451613
FBXO48	1	155	0.006451613
IL36RN	1	155	0.006451613
LMNB2	4	620	0.006451613
MFSD1	3	465	0.006451613
MGST1	1	155	0.006451613
OR5W2	2	310	0.006451613
SNRNP27	1	155	0.006451613
TAF8	2	310	0.006451613
ELF1	4	619	0.006462036
ZBTB7C	4	619	0.006462036
NOTCH3	15	2321	0.006462732
FAM90A1	3	464	0.006465517
KRT28	3	464	0.006465517
NUF2	3	464	0.006465517
DNAH12	20	3092	0.006468305
FAM149A	5	773	0.006468305
MYO5A	12	1855	0.006469003
ASB12	2	309	0.006472492
C4orf47	2	309	0.006472492
DNAJB7	2	309	0.006472492
FAM71F2	2	309	0.006472492
FN3K	2	309	0.006472492
OR4A47	2	309	0.006472492
OR5B21	2	309	0.006472492
OR7A10	2	309	0.006472492
RSPH1	2	309	0.006472492
SLC8A3	6	927	0.006472492
SPEM1	2	309	0.006472492
CTSC	3	463	0.006479482
GPRC6A	6	926	0.006479482
IGSF10	17	2623	0.006481128
SLC6A5	4	617	0.006482982
CEP104	6	925	0.006486486
CCDC73	7	1079	0.006487488
C17orf98	1	154	0.006493506

DAB2	5	770	0.006493506
ERRFI1	3	462	0.006493506
FUOM	1	154	0.006493506
GDF15	2	308	0.006493506
GTF2H3	2	308	0.006493506
HAGH	2	308	0.006493506
KIAA1143	1	154	0.006493506
KRTAP9-9	1	154	0.006493506
OR2T6	2	308	0.006493506
RIMS3	2	308	0.006493506
SPAST	4	616	0.006493506
ZNF610	3	462	0.006493506
CELSR2	19	2923	0.006500171
PCDHGB4	6	923	0.006500542
RGSL1	7	1076	0.006505576
FKTN	3	461	0.006507592
SIL1	3	461	0.006507592
PRR14L	14	2151	0.006508601
TNRC6C	11	1690	0.006508876
RADIL	7	1075	0.006511628
KCNK7	2	307	0.006514658
OR5M3	2	307	0.006514658
OR8K5	2	307	0.006514658
UCP1	2	307	0.006514658
ANGPTL3	3	460	0.006521739
MGA	20	3065	0.006525285
OBSCN	52	7968	0.006526104
SNCAIP	6	919	0.006528836
BLOC1S1	1	153	0.006535948
CSDC2	1	153	0.006535948
EBNA1BP2	2	306	0.006535948
MALL	1	153	0.006535948
NHP2	1	153	0.006535948
NMS	1	153	0.006535948
PABPN1	2	306	0.006535948
RNF25	3	459	0.006535948
STEAP4	3	459	0.006535948
TMEM181	4	612	0.006535948
SRRM4	4	611	0.006546645
ENPP7	3	458	0.006550218
GTDC1	3	458	0.006550218
MLIP	3	458	0.006550218
HADH	5	763	0.00655308
ANKRD23	2	305	0.006557377
CLGN	4	610	0.006557377
RNPS1	2	305	0.006557377
SPATA4	2	305	0.006557377

ST6GALNAC3	2	305	0.006557377
MAP2	12	1827	0.006568144
NUMBL	4	609	0.006568144
KIF13B	12	1826	0.006571742
CP	7	1065	0.00657277
NLRC3	7	1065	0.00657277
ARL14EPL	1	152	0.006578947
C19orf12	1	152	0.006578947
HEY1	2	304	0.006578947
HOXC11	2	304	0.006578947
IFT46	2	304	0.006578947
MAMDC4	8	1216	0.006578947
NAT1	3	456	0.006578947
NINJ1	1	152	0.006578947
PLP2	1	152	0.006578947
RRP8	3	456	0.006578947
UBE2B	1	152	0.006578947
ZNF318	15	2279	0.006581834
BCAN	6	911	0.006586169
PIDD1	6	910	0.006593407
SERINC2	3	455	0.006593407
SH3BP5	3	455	0.006593407
SND1	6	910	0.006593407
ASPH	5	758	0.006596306
PKHD1L1	28	4243	0.006599104
CASP7	2	303	0.00660066
KLHL41	4	606	0.00660066
OR10A2	2	303	0.00660066
SLC26A11	4	606	0.00660066
CD248	5	757	0.00660502
PIBF1	5	757	0.00660502
C1orf27	3	454	0.00660793
MIEF2	3	454	0.00660793
PPM1F	3	454	0.00660793
NEIL3	4	605	0.00661157
BPIFB6	3	453	0.006622517
C1QTNF12	2	302	0.006622517
CAV3	1	151	0.006622517
ECI1	2	302	0.006622517
KLHL5	5	755	0.006622517
NGB	1	151	0.006622517
NPPA	1	151	0.006622517
PRAP1	1	151	0.006622517
RIPPLY1	1	151	0.006622517
SMPDL3A	3	453	0.006622517
ZSCAN12	4	604	0.006622517
SLC25A2	2	301	0.006644518

SLC6A13	4	602	0.006644518
TCEA1	2	301	0.006644518
ALDH1L1	6	902	0.006651885
MUM1	3	451	0.006651885
NKD2	3	451	0.006651885
SH2D7	3	451	0.006651885
TUBB1	3	451	0.006651885
TUBGCP2	6	902	0.006651885
ABCC6	10	1503	0.00665336
PATJ	12	1801	0.006662965
COX5A	1	150	0.006666667
DUSP23	1	150	0.006666667
GYPA	1	150	0.006666667
PDE6D	1	150	0.006666667
PHYKPL	3	450	0.006666667
RGMA	3	450	0.006666667
SLC25A35	2	300	0.006666667
SURF1	2	300	0.006666667
ZC3HAV1L	2	300	0.006666667
PTPRS	13	1948	0.006673511
SLC34A3	4	599	0.006677796
SYNE2	46	6885	0.006681191
DNAJC13	15	2243	0.006687472
FBXW8	4	598	0.006688963
GFRA4	2	299	0.006688963
SLC25A21	2	299	0.006688963
SPRY4	2	299	0.006688963
YJEFN3	2	299	0.006688963
PLEKHH2	10	1493	0.006697924
DNAH7	27	4024	0.006709742
ATP8B4	8	1192	0.006711409
CEND1	1	149	0.006711409
CKAP2L	5	745	0.006711409
DTWD2	2	298	0.006711409
MTTP	6	894	0.006711409
OOEP	1	149	0.006711409
SLC26A5	5	744	0.00672043
TCOF1	10	1488	0.00672043
GATA6	4	595	0.006722689
KIAA1614	8	1190	0.006722689
ACTN1	6	892	0.006726457
RUNDC3A	3	446	0.006726457
SOX8	3	446	0.006726457
FAM160A1	7	1040	0.006730769
ERCC1	2	297	0.006734007
PLSCR2	2	297	0.006734007
SH3TC1	9	1336	0.006736527

CTBP2	3	445	0.006741573
PIEZO1	17	2521	0.006743356
ELF2	4	593	0.006745363
RBM47	4	593	0.006745363
PACS2	6	889	0.006749156
ACAP1	5	740	0.006756757
CRIM1	7	1036	0.006756757
GEM	2	296	0.006756757
PACRG	2	296	0.006756757
PCDH12	8	1184	0.006756757
PDXP	2	296	0.006756757
POM121L12	2	296	0.006756757
RNFT2	3	444	0.006756757
SSBP1	1	148	0.006756757
TMEM241	2	296	0.006756757
MPDZ	14	2070	0.006763285
IL17RD	5	739	0.0067659
IQSEC3	8	1182	0.00676819
MET	7	1034	0.006769826
ABCB10	5	738	0.006775068
ZNF235	5	738	0.006775068
C7orf57	2	295	0.006779661
LIME1	2	295	0.006779661
NADK2	3	442	0.00678733
SDC3	3	442	0.00678733
TFAP2E	3	442	0.00678733
CLEC4F	4	589	0.006791171
BACH1	5	736	0.006793478
BRSK2	5	736	0.006793478
DVL2	5	736	0.006793478
KRBA1	7	1030	0.006796117
PTPRD	13	1912	0.006799163
ANG	1	147	0.006802721
C5orf51	2	294	0.006802721
C8orf74	2	294	0.006802721
CARHSP1	1	147	0.006802721
ETS1	3	441	0.006802721
FHIT	1	147	0.006802721
MGST2	1	147	0.006802721
NDUFAF7	3	441	0.006802721
OXLD1	1	147	0.006802721
PASK	9	1323	0.006802721
POLE3	1	147	0.006802721
RMI2	1	147	0.006802721
RNASE4	1	147	0.006802721
TMEM110	2	294	0.006802721
TRMO	3	441	0.006802721

UBE2D3	1	147	0.006802721
ARHGAP17	6	881	0.006810443
FAM111B	5	734	0.006811989
FPGS	4	587	0.00681431
MYPN	9	1320	0.006818182
MTCL1	13	1905	0.006824147
ABHD14A-ACY1	4	586	0.006825939
EXOSC2	2	293	0.006825939
GGT5	4	586	0.006825939
NEK10	8	1172	0.006825939
TBC1D7	2	293	0.006825939
TNFRSF13B	2	293	0.006825939
EXOSC9	3	439	0.006833713
GTF2E1	3	439	0.006833713
SPOCK1	3	439	0.006833713
TTC21B	9	1316	0.006838906
ACP7	3	438	0.006849315
AKTIP	2	292	0.006849315
C8orf76	2	292	0.006849315
CALML5	1	146	0.006849315
CST3	1	146	0.006849315
DECR2	2	292	0.006849315
ECHDC2	2	292	0.006849315
GCDH	3	438	0.006849315
JTB	1	146	0.006849315
MED9	1	146	0.006849315
PIP	1	146	0.006849315
RHBDL1	3	438	0.006849315
SLC48A1	1	146	0.006849315
THSD7B	11	1606	0.006849315
TMEM207	1	146	0.006849315
ZNF677	4	584	0.006849315
PLB1	10	1458	0.006858711
KLHL35	4	583	0.006861063
TSPYL1	3	437	0.006864989
TMPRSS15	7	1019	0.00686948
ATP4B	2	291	0.006872852
BPHL	2	291	0.006872852
IMP4	2	291	0.006872852
LARP7	4	582	0.006872852
STOML3	2	291	0.006872852
TRAFD1	4	582	0.006872852
PLOD1	5	727	0.006877579
KRT34	3	436	0.006880734
PM20D2	3	436	0.006880734
SPOCK3	3	436	0.006880734
TTF2	8	1162	0.006884682

C7orf43	4	580	0.006896552
CARF	4	580	0.006896552
CCDC141	10	1450	0.006896552
CCNJL	3	435	0.006896552
CD274	2	290	0.006896552
CREG2	2	290	0.006896552
CSTL1	1	145	0.006896552
NDUFA12	1	145	0.006896552
PPP1R14D	1	145	0.006896552
PROSER2	3	435	0.006896552
RPS19	1	145	0.006896552
SELENOM	1	145	0.006896552
SYNJ2BP	1	145	0.006896552
AKAP6	16	2319	0.006899526
TAF1C	6	869	0.006904488
SLC15A5	4	579	0.006908463
ENO1	3	434	0.006912442
FAM83A	3	434	0.006912442
FBXL21	3	434	0.006912442
GPAT3	3	434	0.006912442
NR1I2	3	434	0.006912442
PBX3	3	434	0.006912442
PKD1L2	17	2459	0.006913379
AXDND1	7	1012	0.006916996
CCP110	7	1012	0.006916996
CECR6	4	578	0.006920415
IYD	2	289	0.006920415
NOX4	4	578	0.006920415
ARRDC1	3	433	0.006928406
GJA8	3	433	0.006928406
IL17RA	6	866	0.006928406
ZSCAN4	3	433	0.006928406
PDZD8	8	1154	0.006932409
SHQ1	4	577	0.006932409
LARGE2	5	721	0.006934813
XYLT2	6	865	0.006936416
MROH2B	11	1585	0.006940063
CACNA1S	13	1873	0.006940737
ACADSB	3	432	0.006944444
IMPA2	2	288	0.006944444
LTK	6	864	0.006944444
MED21	1	144	0.006944444
PLA2G2A	1	144	0.006944444
RHOXF2	2	288	0.006944444
PLXNA3	13	1871	0.006948156
POLQ	18	2590	0.006949807
SLC44A5	5	719	0.006954103

MAN2A2	8	1150	0.006956522
CARD8	3	431	0.006960557
KRT40	3	431	0.006960557
NOCT	3	431	0.006960557
EPHA8	7	1005	0.006965174
IQCK	2	287	0.006968641
LRRIQ1	12	1722	0.006968641
SLC25A10	2	287	0.006968641
SUN2	5	717	0.006973501
FAT1	32	4588	0.006974717
DBNL	3	430	0.006976744
GOT2	3	430	0.006976744
MRPS5	3	430	0.006976744
NOM1	6	860	0.006976744
DIP2B	11	1576	0.006979695
MPEG1	5	716	0.00698324
OPLAH	9	1288	0.006987578
AGBL3	7	1001	0.006993007
CC2D1B	6	858	0.006993007
NAT6	2	286	0.006993007
PNPLA5	3	429	0.006993007
SRRM5	5	715	0.006993007
ZNF544	5	715	0.006993007
CHD6	19	2715	0.006998158
HTT	22	3142	0.00700191
CAPN1	5	714	0.007002801
PSG3	3	428	0.007009346
MIP	5	713	0.007012623
DPH5	2	285	0.007017544
EME1	4	570	0.007017544
FRMD5	4	570	0.007017544
GOLPH3L	2	285	0.007017544
RERE	11	1566	0.007024266
ZNF134	3	427	0.007025761
TPRN	5	711	0.007032349
ITPR2	19	2701	0.007034432
MYO5B	13	1848	0.007034632
ARHGEF28	12	1705	0.007038123
BBS12	5	710	0.007042254
BLOC1S2	1	142	0.007042254
CHCHD4	1	142	0.007042254
CLC	1	142	0.007042254
FAM181B	3	426	0.007042254
GMFG	1	142	0.007042254
INAFM1	1	142	0.007042254
PAPLN	9	1278	0.007042254
RLN3	1	142	0.007042254

SEZ6	7	994	0.007042254
SMAD7	3	426	0.007042254
ZNF662	3	426	0.007042254
KANK2	6	851	0.007050529
XIRP1	13	1843	0.007053717
ATP11A	8	1134	0.007054674
IL4I1	4	567	0.007054674
SPHKAP	12	1700	0.007058824
CUL7	12	1698	0.007067138
VDAC3	2	283	0.007067138
LMF2	5	707	0.007072136
SHKBP1	5	707	0.007072136
BMP10	3	424	0.007075472
PACSIN3	3	424	0.007075472
ZP3	3	424	0.007075472
AQP6	2	282	0.007092199
CD320	2	282	0.007092199
KRT6A	4	564	0.007092199
LIPM	3	423	0.007092199
LIX1	2	282	0.007092199
NDUFA11	1	141	0.007092199
NOX1	4	564	0.007092199
PIANP	2	282	0.007092199
FAM114A1	4	563	0.007104796
NCLN	4	563	0.007104796
DUOX2	11	1548	0.007105943
AKAP13	20	2813	0.007109847
CEP63	5	703	0.007112376
ZAN	20	2812	0.007112376
PKN2	7	984	0.007113821
CCDC155	4	562	0.007117438
ZNF783	2	281	0.007117438
DOCK4	14	1966	0.007121058
CNP	3	421	0.007125891
CPA4	3	421	0.007125891
AHRR	5	701	0.007132668
BACH2	6	841	0.007134364
CCDC126	1	140	0.007142857
IL5RA	3	420	0.007142857
NXT1	1	140	0.007142857
TMEM18	1	140	0.007142857
CWH43	5	699	0.007153076
CHAF1B	4	559	0.007155635
USP8	8	1118	0.007155635
GPR151	3	419	0.007159905
KIFC2	6	838	0.007159905
POU4F1	3	419	0.007159905

PSG4	3	419	0.007159905
MTRR	5	698	0.007163324
VPS54	7	977	0.00716479
FMO2	4	558	0.007168459
KIF21A	12	1674	0.007168459
R3HDM2	7	976	0.007172131
LEXM	3	418	0.007177033
SMYD5	3	418	0.007177033
TEKT1	3	418	0.007177033
CAPS2	4	557	0.007181329
CCT8L2	4	557	0.007181329
IK	4	557	0.007181329
SLC22A5	4	557	0.007181329
CCDC158	8	1113	0.007187781
SYCP2	11	1530	0.007189542
ACAP3	6	834	0.007194245
ADCY8	9	1251	0.007194245
AGPAT2	2	278	0.007194245
AMPH	5	695	0.007194245
CCDC121	2	278	0.007194245
FBXO17	2	278	0.007194245
GIF	3	417	0.007194245
HEXB	4	556	0.007194245
INSL4	1	139	0.007194245
MSMP	1	139	0.007194245
MTIF3	2	278	0.007194245
NEDD9	6	834	0.007194245
PPP4R2	3	417	0.007194245
TPP1	2	278	0.007194245
VHLL	1	139	0.007194245
GAB1	5	694	0.007204611
SLC22A2	4	555	0.007207207
ACTL9	3	416	0.007211538
DDX11	7	970	0.007216495
NID1	9	1247	0.007217322
SASH1	9	1247	0.007217322
INSIG1	2	277	0.007220217
MYO1C	8	1108	0.007220217
RAB40A	2	277	0.007220217
ZNF354C	4	554	0.007220217
AKAP8	5	692	0.007225434
TG	20	2768	0.007225434
CSNK1D	3	415	0.007228916
PDSS1	3	415	0.007228916
PLBD1	4	553	0.007233273
CDH3	6	829	0.007237636
THSD7A	12	1657	0.007242004

TET3	13	1795	0.00724234
C5orf63	1	138	0.007246377
PPP6R2	7	966	0.007246377
RASGRP3	5	690	0.007246377
SCGN	2	276	0.007246377
SOX17	3	414	0.007246377
TGFB2	3	414	0.007246377
WDR27	6	827	0.007255139
PLEC	34	4684	0.007258753
CFAP45	4	551	0.007259528
CLCC1	4	551	0.007259528
SIGLEC5	4	551	0.007259528
TRAF3IP3	4	551	0.007259528
ELAC2	6	826	0.007263923
ARHGAP30	8	1101	0.007266122
ROBO1	12	1651	0.007268322
TPRG1	2	275	0.007272727
TSGA13	2	275	0.007272727
ZNF687	9	1237	0.007275667
LAMB1	13	1786	0.007278835
ZACN	3	412	0.007281553
CTDP1	7	961	0.007284079
EPB41L4A	5	686	0.00728863
CRABP1	1	137	0.00729927
MRPS16	1	137	0.00729927
ODF3L1	2	274	0.00729927
PYCR3	2	274	0.00729927
STIM1	5	685	0.00729927
ZFYVE27	3	411	0.00729927
LAMA5	27	3695	0.007307172
CAPN3	6	821	0.007308161
DRP2	7	957	0.007314525
HOXA10	3	410	0.007317073
ABLIM3	5	683	0.007320644
SLC27A3	5	683	0.007320644
PAQR4	2	273	0.007326007
AFAP1L2	6	818	0.007334963
DEPTOR	3	409	0.007334963
TTI1	8	1089	0.007346189
TEX14	11	1497	0.007348029
COQ8B	4	544	0.007352941
GATC	1	136	0.007352941
N4BP3	4	544	0.007352941
PCP2	1	136	0.007352941
SRP14	1	136	0.007352941
SYPL2	2	272	0.007352941
ZNF324B	4	544	0.007352941

EPB41L3	8	1087	0.007359706
SFSWAP	7	951	0.007360673
CAPN15	8	1086	0.007366483
CYP1B1	4	543	0.007366483
YES1	4	543	0.007366483
CDH3	6	814	0.007371007
EIF3B	6	814	0.007371007
GDF11	3	407	0.007371007
ALPK2	16	2170	0.007373272
COLEC11	2	271	0.007380074
FUK	8	1084	0.007380074
PLPP4	2	271	0.007380074
PLSCR5	2	271	0.007380074
RELL1	2	271	0.007380074
KDM3B	13	1761	0.007382169
FKBP15	9	1219	0.007383101
GLB1	5	677	0.007385524
ANGPTL4	3	406	0.007389163
ARFGAP1	3	406	0.007389163
LRRC41	6	812	0.007389163
MTG2	3	406	0.007389163
UST	3	406	0.007389163
MYCBPAP	7	947	0.007391763
EXTL1	5	676	0.00739645
IFT81	5	676	0.00739645
TMPRSS6	6	811	0.007398274
RIPOR3	7	946	0.007399577
MYO18B	19	2567	0.007401636
TARBP1	12	1621	0.007402838
CCSAP	2	270	0.007407407
CELA3B	2	270	0.007407407
CITED2	2	270	0.007407407
DMTN	3	405	0.007407407
H3F3C	1	135	0.007407407
HDAC6	9	1215	0.007407407
HOXD12	2	270	0.007407407
KCNIP2	2	270	0.007407407
PLG	6	810	0.007407407
RBP1	1	135	0.007407407
SLC2A9	4	540	0.007407407
SPI1	2	270	0.007407407
SAMD15	5	674	0.007418398
ZNF429	5	674	0.007418398
ALDH7A1	4	539	0.00742115
ADCY4	8	1077	0.007428041
CLPTM1L	4	538	0.007434944
FAM214B	4	538	0.007434944

NECTIN2	4	538	0.007434944
NOTCH1	19	2555	0.007436399
NHSL1	12	1610	0.007453416
TMC6	6	805	0.007453416
CENPQ	2	268	0.007462687
EXOC6	6	804	0.007462687
TEX43	1	134	0.007462687
XYLB	4	536	0.007462687
ADGRA2	10	1338	0.007473842
FBN3	21	2809	0.00747597
ALPP	4	535	0.007476636
FMO2	4	535	0.007476636
LAT2	4	535	0.007476636
ARHGEF19	6	802	0.007481297
MARVELD3	3	401	0.007481297
SCNN1D	6	802	0.007481297
CEP192	19	2537	0.00748916
DENND1C	6	801	0.007490637
ITM2C	2	267	0.007490637
PHOSPHO1	2	267	0.007490637
RWDD3	2	267	0.007490637
TET1	16	2136	0.007490637
TTYH2	4	534	0.007490637
UBE3B	8	1068	0.007490637
ASCC1	3	400	0.0075
TRERF1	9	1200	0.0075
PLD2	7	933	0.00750268
LCP2	4	533	0.00750469
RBM46	4	533	0.00750469
C10orf105	1	133	0.007518797
C16orf47	1	133	0.007518797
FABP3	1	133	0.007518797
GRB7	4	532	0.007518797
POLR1D	1	133	0.007518797
TTC30A	5	665	0.007518797
MYO1C	8	1063	0.00752587
ATP10B	11	1461	0.00752909
FANCI	10	1328	0.00753012
FAM53A	3	398	0.007537688
KDF1	3	398	0.007537688
ADGRL2	11	1459	0.007539411
CTCFL	5	663	0.007541478
TIAM1	12	1591	0.007542426
FAM129A	7	928	0.007543103
C17orf99	2	265	0.00754717
CLEC12A	2	265	0.00754717
DNA2	8	1060	0.00754717

SLC29A4	4	530	0.00754717
VWDE	12	1590	0.00754717
ZNF628	8	1059	0.007554297
VPS37A	3	397	0.007556675
ERN2	7	926	0.007559395
FANCA	11	1455	0.007560137
GEMIN4	8	1058	0.007561437
CCDC175	6	793	0.007566204
DNAJC10	6	793	0.007566204
C19orf24	1	132	0.007575758
FAM161A	5	660	0.007575758
IFT20	1	132	0.007575758
SNRNP25	1	132	0.007575758
STK32A	3	396	0.007575758
MROH5	10	1318	0.007587253
DLGAP2	8	1054	0.007590133
TBCE	4	527	0.007590133
CRACR2A	3	395	0.007594937
ZNF385D	3	395	0.007594937
ANKRD28	8	1053	0.007597341
ALG8	4	526	0.007604563
C1orf35	2	263	0.007604563
FLVCR2	4	526	0.007604563
RPGRIP1L	10	1315	0.007604563
STK36	10	1315	0.007604563
PTPRO	11	1446	0.007607192
BCLAF1	7	920	0.007608696
HAL	5	657	0.00761035
ALKBH5	3	394	0.007614213
HID1	6	788	0.007614213
PDXDC1	6	788	0.007614213
SETD1A	13	1707	0.0076157
PWP2	7	919	0.007616975
ANKS3	5	656	0.007621951
SMO	6	787	0.007623888
ALPL	4	524	0.007633588
CA13	2	262	0.007633588
FAM83H	9	1179	0.007633588
KATNB1	5	655	0.007633588
LEPR	1	131	0.007633588
MED31	1	131	0.007633588
PPOX	1	131	0.007633588
SRPK1	5	655	0.007633588
VWA5A	6	786	0.007633588
ERCC4	7	916	0.007641921
EARS2	4	523	0.007648184
CACNA1H	18	2353	0.007649809

CCDC57	7	915	0.007650273
FEZ1	3	392	0.007653061
KCP	12	1568	0.007653061
PAAF1	3	392	0.007653061
GLB1L3	5	653	0.007656968
KLK3	2	261	0.007662835
PIK3CD	8	1044	0.007662835
SLA2	2	261	0.007662835
SPATA46	2	261	0.007662835
CSNK2A3	3	391	0.007672634
WASHC4	9	1173	0.007672634
MEX3D	5	651	0.007680492
THADA	15	1953	0.007680492
BAHD1	6	780	0.007692308
C4orf26	1	130	0.007692308
CAVIN1	3	390	0.007692308
DPM1	2	260	0.007692308
DUS3L	5	650	0.007692308
H2AC15	1	130	0.007692308
HIST1H2AM	1	130	0.007692308
PTGER3	3	390	0.007692308
STRN	6	780	0.007692308
TUFT1	3	390	0.007692308
VNN2	4	520	0.007692308
TNN	10	1299	0.007698229
EML2	5	649	0.00770416
ADAMTS13	11	1427	0.007708479
ALKBH1	3	389	0.007712082
SIRT2	3	389	0.007712082
ATAD3B	5	648	0.007716049
TCHH	15	1943	0.007720021
GFY	4	518	0.007722008
IGFBP1	2	259	0.007722008
UNC50	2	259	0.007722008
UNC5CL	4	518	0.007722008
CDH2	7	906	0.007726269
SCN7A	13	1682	0.007728894
ECEL1	6	775	0.007741935
MCM2	7	904	0.007743363
CDH23	26	3354	0.007751938
FSHB	1	129	0.007751938
PALM	3	387	0.007751938
PFN4	1	129	0.007751938
PGLS	2	258	0.007751938
ZNF808	7	903	0.007751938
KRT1	5	644	0.007763975
TTLL12	5	644	0.007763975

MROH2A	13	1674	0.00776583
SLC16A10	4	515	0.00776699
CPT1B	6	772	0.007772021
NECAB2	3	386	0.007772021
TIA1	3	386	0.007772021
TTLL3	6	772	0.007772021
SPG11	19	2443	0.007777323
ATP5SL	2	257	0.007782101
EXD1	4	514	0.007782101
KCTD1	2	257	0.007782101
LRRC14B	4	514	0.007782101
TM9SF4	5	642	0.007788162
ALDH3B2	3	385	0.007792208
PNKD	3	385	0.007792208
PSKH2	3	385	0.007792208
SEMA4F	6	770	0.007792208
KRT82	4	513	0.007797271
TSNARE1	4	513	0.007797271
VNN1	4	513	0.007797271
CSF2RB	7	897	0.00780379
DOT1L	12	1537	0.007807417
HIST1H2AI	1	128	0.0078125
KRTAP2-1	1	128	0.0078125
MIER1	4	512	0.0078125
MRPL51	1	128	0.0078125
NDUFA6	1	128	0.0078125
PHLDB3	5	640	0.0078125
RIPPLY2	1	128	0.0078125
RNASET2	2	256	0.0078125
SLC2A7	4	512	0.0078125
UMOD	5	640	0.0078125
CTTNBP2	13	1663	0.007817198
RIN2	7	895	0.007821229
AMPD3	6	767	0.007822686
PTCHD3	6	767	0.007822686
SEC23B	6	767	0.007822686
SLC34A1	5	639	0.007824726
PPP2R3A	9	1150	0.007826087
HERC6	8	1022	0.007827789
CNTLN	11	1405	0.007829181
KRT76	5	638	0.007836991
ANKK1	6	765	0.007843137
OLFM4	4	510	0.007843137
SKA1	2	255	0.007843137
SUSD3	2	255	0.007843137
ATXN7	7	892	0.007847534
MYO9A	20	2548	0.007849294

APOBEC3B	3	382	0.007853403
AKAP12	14	1782	0.007856341
SHB	4	509	0.007858546
USP35	8	1018	0.007858546
EGFLAM	8	1017	0.007866273
BICDL2	4	508	0.007874016
FAM229A	1	127	0.007874016
HIST1H2BA	1	127	0.007874016
KLK4	2	254	0.007874016
LIMD2	1	127	0.007874016
MAML1	8	1016	0.007874016
ODF3	2	254	0.007874016
PDYN	2	254	0.007874016
POLE	18	2286	0.007874016
RAB6C	2	254	0.007874016
TLL2	8	1015	0.007881773
C16orf96	9	1141	0.007887818
OSBPL3	7	887	0.00789177
SPTBN5	29	3674	0.007893304
DMTF1	6	760	0.007894737
TTC19	3	380	0.007894737
CLPX	5	633	0.007898894
CPNE7	5	633	0.007898894
XRCC1	5	633	0.007898894
FGFR1OP2	2	253	0.007905138
TRIM46	6	759	0.007905138
N4BP2	14	1770	0.007909605
ILVBL	5	632	0.007911392
THEG	3	379	0.007915567
CYP4Z1	4	505	0.007920792
FRK	4	505	0.007920792
OGDHL	8	1010	0.007920792
CEP68	6	757	0.007926024
C2orf76	1	126	0.007936508
EMX2	2	252	0.007936508
H2BC6	1	126	0.007936508
H2BC7	1	126	0.007936508
HIST1H2BB	1	126	0.007936508
HIST1H2BD	1	126	0.007936508
HIST1H2BH	1	126	0.007936508
HIST2H2BF	1	126	0.007936508
NMNAT3	2	252	0.007936508
RCAN1	2	252	0.007936508
RFPL2	3	378	0.007936508
FBF1	9	1133	0.007943513
PWWP2A	6	755	0.00794702
CYP3A43	4	503	0.007952286

FFAR4	3	377	0.00795756
NCK1	3	377	0.00795756
TDRD9	11	1382	0.007959479
EPB41L2	8	1005	0.007960199
ZNF528	5	628	0.007961783
AZU1	2	251	0.007968127
C22orf42	2	251	0.007968127
HDGFL1	2	251	0.007968127
PM20D1	4	502	0.007968127
WASF3	4	502	0.007968127
VAV2	7	878	0.007972665
SERPINB6	3	376	0.007978723
ARMC6	4	501	0.007984032
PDX1	4	501	0.007984032
SLC13A4	5	626	0.00798722
GFM1	6	751	0.007989348
DNAJB12	3	375	0.008
FANCL	3	375	0.008
KLK9	2	250	0.008
LRP11	4	500	0.008
LYPD2	1	125	0.008
MRPS6	1	125	0.008
ODF1	2	250	0.008
TRMT2A	5	625	0.008
OTOF	16	1997	0.008012018
ARHGAP24	6	748	0.00802139
SUMF1	3	374	0.00802139
TKT	5	623	0.008025682
ARMC3	7	872	0.008027523
DIO1	2	249	0.008032129
TCHP	4	498	0.008032129
MYO5C	14	1742	0.008036739
LRRC70	5	622	0.008038585
APOBEC3F	3	373	0.008042895
EXOC3L1	6	746	0.008042895
FOXE1	3	373	0.008042895
HYKK	3	373	0.008042895
PGLYRP4	3	373	0.008042895
ACAD9	5	621	0.00805153
SFI1	10	1242	0.00805153
FSD1	4	496	0.008064516
GRXCR2	2	248	0.008064516
NCBP3	5	620	0.008064516
RABEPK	3	372	0.008064516
SFTPA1	2	248	0.008064516
TP53AIP1	1	124	0.008064516
FAM160B2	6	743	0.00807537

DCAF4	4	495	0.008080808
MBOAT1	4	495	0.008080808
LRRC58	3	371	0.008086253
SLC39A13	3	371	0.008086253
TTF1	3	371	0.008086253
DPP6	7	865	0.008092486
ABCC12	11	1359	0.008094187
CYP2A6	4	494	0.008097166
FBXL22	2	247	0.008097166
NIPSNAP3A	2	247	0.008097166
DCP1B	5	617	0.008103728
ZNF813	5	617	0.008103728
COL16A1	13	1604	0.008104738
DAPK2	3	370	0.008108108
USP13	7	863	0.00811124
KRT83	4	493	0.00811359
C11orf52	1	123	0.008130081
CAPS	11	1353	0.008130081
KCNE2	1	123	0.008130081
MELTF	6	738	0.008130081
MEN1	5	615	0.008130081
MRI1	3	369	0.008130081
PLOD3	6	738	0.008130081
RPL35	1	123	0.008130081
RTP4	2	246	0.008130081
TMUB1	2	246	0.008130081
TXNDC17	1	123	0.008130081
VPREB3	1	123	0.008130081
MPHOSPH8	7	860	0.008139535
ZNF276	5	614	0.008143322
FDXR	4	491	0.00814664
P3H3	6	736	0.008152174
ZNF804B	11	1349	0.008154188
BDH2	2	245	0.008163265
CYP2C9	4	490	0.008163265
CYP2W1	4	490	0.008163265
FOLR3	2	245	0.008163265
GNAS	2	245	0.008163265
PLD3	4	490	0.008163265
SHANK2	12	1470	0.008163265
TSPAN16	2	245	0.008163265
ADAMTS16	10	1224	0.008169935
OSMR	8	979	0.008171604
ZNF541	11	1346	0.008172363
GDAP1L1	3	367	0.008174387
KCNAB2	3	367	0.008174387
TSSK1B	3	367	0.008174387

DNAH6	34	4158	0.008177008
SAMD9	13	1589	0.008181246
CFAP100	5	611	0.008183306
DDR2	7	855	0.008187135
AAMDC	1	122	0.008196721
C10orf25	1	122	0.008196721
C6orf141	2	244	0.008196721
C7orf34	1	122	0.008196721
CEACAM4	2	244	0.008196721
DPEP3	4	488	0.008196721
IDH3A	3	366	0.008196721
INO80E	2	244	0.008196721
LYRM1	1	122	0.008196721
MED19	2	244	0.008196721
POLR1D	1	122	0.008196721
SELE	5	610	0.008196721
TMEM25	3	366	0.008196721
TRIM34	4	488	0.008196721
ULBP3	2	244	0.008196721
NLRX1	8	975	0.008205128
C17orf80	5	609	0.008210181
MST1	4	487	0.008213552
FUT1	3	365	0.008219178
SULT2B1	3	365	0.008219178
AGRN	17	2068	0.008220503
C17orf77	2	243	0.008230453
C1orf174	2	243	0.008230453
GAL3ST4	4	486	0.008230453
HEXDC	4	486	0.008230453
ZNF561	4	486	0.008230453
LIG1	9	1093	0.008234218
ALDOB	3	364	0.008241758
C2CD4B	3	364	0.008241758
GDF3	3	364	0.008241758
HAVCR1	3	364	0.008241758
WWC3	9	1092	0.008241758
SON	20	2426	0.008244023
GMIP	8	970	0.008247423
ZNF214	5	606	0.008250825
UTP20	23	2785	0.008258528
AIFM3	5	605	0.008264463
ANKZF1	6	726	0.008264463
C15orf65	1	121	0.008264463
C1orf21	1	121	0.008264463
C6orf223	2	242	0.008264463
CASP14	2	242	0.008264463
CBY3	2	242	0.008264463

CLPSL1	1	121	0.008264463
DKKL1	2	242	0.008264463
EIF4E1B	2	242	0.008264463
EMC7	2	242	0.008264463
FSTL5	7	847	0.008264463
MAP1LC3A	1	121	0.008264463
NAA60	2	242	0.008264463
PAPD4	4	484	0.008264463
PYGL	7	847	0.008264463
RAD23A	3	363	0.008264463
SP9	4	484	0.008264463
HELB	9	1087	0.008279669
NNT	9	1086	0.008287293
TMEM129	3	362	0.008287293
IGSF6	2	241	0.008298755
PYGB	7	843	0.008303677
USP42	11	1324	0.008308157
SURF6	3	361	0.008310249
PYGM	7	842	0.008313539
ARVCF	8	962	0.008316008
C1orf116	5	601	0.008319468
CASKIN2	10	1202	0.008319468
PRR11	3	360	0.008333333
RPRML	1	120	0.008333333
SNUPN	3	360	0.008333333
SOD3	2	240	0.008333333
TMEM198	3	360	0.008333333
PAK5	6	719	0.008344924
ZNF530	5	599	0.008347245
DIS3	8	958	0.008350731
FUT10	4	479	0.008350731
AKR7A2	3	359	0.008356546
PTGDR	3	359	0.008356546
PCNX3	17	2034	0.008357915
IQCB1	5	598	0.008361204
KAT2A	7	837	0.008363202
ABTB1	4	478	0.008368201
EFHD1	2	239	0.008368201
PSMB6	2	239	0.008368201
THAP3	2	239	0.008368201
POSTN	7	836	0.008373206
TEP1	22	2627	0.008374572
GPR87	3	358	0.008379888
TRIM54	3	358	0.008379888
OSGIN1	4	477	0.008385744
ABCA7	18	2146	0.008387698
USP53	9	1073	0.008387698

GAK	11	1311	0.008390542
EPX	6	715	0.008391608
ADPRH	3	357	0.008403361
ATP6V1F	1	119	0.008403361
EFNA3	2	238	0.008403361
GPRC5A	3	357	0.008403361
ID3	1	119	0.008403361
NUDT7	2	238	0.008403361
PRDM6	5	595	0.008403361
RASSF3	2	238	0.008403361
RHNO1	2	238	0.008403361
YPEL3	1	119	0.008403361
ZNF317	5	595	0.008403361
ZNF467	5	595	0.008403361
ZNF563	4	476	0.008403361
ZC3H6	10	1189	0.008410429
ABCC2	13	1545	0.008414239
FAM71A	5	594	0.008417508
ASZ1	4	475	0.008421053
LTV1	4	475	0.008421053
TULP4	13	1543	0.008425146
HIP1R	9	1068	0.008426966
ZBTB39	6	712	0.008426966
STXBP2	5	593	0.008431703
REXO2	2	237	0.008438819
SOX4	4	474	0.008438819
G6PC2	3	355	0.008450704
LRIG2	9	1065	0.008450704
RGL3	6	710	0.008450704
PPAN	4	473	0.00845666
C8B	5	591	0.008460237
UVSSA	6	709	0.008462623
ATP6V1G3	1	118	0.008474576
BMP3	4	472	0.008474576
C19orf25	1	118	0.008474576
ELOB	1	118	0.008474576
GNAT2	3	354	0.008474576
LCE1C	1	118	0.008474576
LCE1F	1	118	0.008474576
LCE5A	1	118	0.008474576
PON3	3	354	0.008474576
RBM24	2	236	0.008474576
SNRPD2	1	118	0.008474576
TXNDC16	7	825	0.008484848
CPAMD8	16	1885	0.008488064
PCDH1	9	1060	0.008490566
MPND	4	471	0.008492569

P2RX2	4	471	0.008492569
BDKRB1	3	353	0.008498584
TCP10L2	3	353	0.008498584
TGM6	6	706	0.008498584
ZNF225	6	706	0.008498584
VPS11	8	941	0.008501594
CCIN	5	588	0.008503401
MAL	2	235	0.008510638
MPZL3	2	235	0.008510638
SLC7A13	4	470	0.008510638
SNX17	4	470	0.008510638
TMEM176A	2	235	0.008510638
TOR1AIP2	4	470	0.008510638
ABCC3	13	1527	0.008513425
AMBP	3	352	0.008522727
PRSS55	3	352	0.008522727
BRAT1	7	821	0.008526188
ETS2	4	469	0.008528785
USP21	9	1055	0.008530806
LMNB1	5	586	0.008532423
COL8A2	6	703	0.008534851
USP6	12	1406	0.008534851
ADAM29	7	820	0.008536585
ATF4	3	351	0.008547009
GHRL	1	117	0.008547009
KRT26	4	468	0.008547009
TAAR2	3	351	0.008547009
TMOD2	3	351	0.008547009
KRT10	5	584	0.008561644
ASB10	4	467	0.00856531
DISP2	12	1401	0.00856531
SLC35E4	3	350	0.008571429
ANO7	8	933	0.008574491
PDE11A	8	933	0.008574491
KNDC1	15	1749	0.008576329
DNAH2	38	4427	0.008583691
PSD3	9	1048	0.008587786
ABHD5	3	349	0.008595989
DRAXIN	3	349	0.008595989
GLT8D2	3	349	0.008595989
KHDRBS2	3	349	0.008595989
USP45	7	814	0.008599509
GFRA1	4	465	0.008602151
WDR49	6	697	0.008608321
ITGAD	10	1161	0.008613264
GALP	1	116	0.00862069
LTBP4	14	1624	0.00862069

NDUFA5	1	116	0.00862069
GRIN3B	9	1043	0.008628955
IQCE	6	695	0.008633094
MICALCL	6	695	0.008633094
CDKAL1	5	579	0.008635579
NHLRC3	3	347	0.008645533
GRK4	5	578	0.008650519
KIAA0556	14	1618	0.008652658
HSPG2	38	4391	0.008654065
ATG9B	8	924	0.008658009
PRRG3	2	231	0.008658009
TBC1D10B	7	808	0.008663366
ACSM1	5	577	0.008665511
CCDC136	10	1154	0.008665511
AGA	3	346	0.00867052
CDK20	3	346	0.00867052
TNKS1BP1	15	1729	0.008675535
GPRC5D	3	345	0.008695652
MLN	1	115	0.008695652
OGG1	3	345	0.008695652
PKD2L1	7	805	0.008695652
TMEM218	1	115	0.008695652
TMOD4	3	345	0.008695652
WSCD1	5	575	0.008695652
ZNF230	2	230	0.008695652
CRYBG1	15	1723	0.008705746
LY75	15	1722	0.008710801
SQLE	5	574	0.008710801
TRAF3IP2	5	574	0.008710801
TMEM143	4	459	0.008714597
AURKB	3	344	0.00872093
NOL6	10	1146	0.008726003
ATG4C	4	458	0.008733624
ATRAID	2	229	0.008733624
DEPDC5	14	1603	0.008733624
KBTBD13	4	458	0.008733624
NAA11	2	229	0.008733624
RAB33B	2	229	0.008733624
ZNF19	4	458	0.008733624
CRMP1	5	572	0.008741259
DBX1	3	343	0.008746356
FUT2	3	343	0.008746356
PRSS8	3	343	0.008746356
CCDC62	6	684	0.00877193
IFITM10	2	228	0.00877193
PDZK1IP1	1	114	0.00877193
PPDPF	1	114	0.00877193

S100A9	1	114	0.00877193
ZNF76	5	570	0.00877193
IFIH1	9	1025	0.008780488
CKAP2	6	683	0.008784773
GGT1	5	569	0.008787346
MMP21	5	569	0.008787346
CACNA2D4	10	1137	0.008795075
MCF2L	10	1137	0.008795075
SMG7	10	1137	0.008795075
TBC1D5	7	795	0.008805031
BASP1	2	227	0.008810573
KCNIP1	2	227	0.008810573
MCMDC2	6	681	0.008810573
PAK5	6	681	0.008810573
SLC5A9	6	681	0.008810573
TMED1	2	227	0.008810573
UBTD1	2	227	0.008810573
PPP1R16B	5	567	0.008818342
SLC10A7	3	340	0.008823529
RC3H1	10	1133	0.008826125
PCDHB7	7	793	0.008827238
PLEKHH3	7	793	0.008827238
HTRA3	4	453	0.008830022
SIGLEC6	4	453	0.008830022
ANKRD33	4	452	0.008849558
ATP6V1E2	2	226	0.008849558
CCL15	1	113	0.008849558
CD79A	2	226	0.008849558
DHRS7	3	339	0.008849558
EIF1B	1	113	0.008849558
FBP2	3	339	0.008849558
IFI44L	4	452	0.008849558
KRT80	4	452	0.008849558
MICALL2	8	904	0.008849558
NPFF	1	113	0.008849558
SLC25A12	6	678	0.008849558
VIT	6	678	0.008849558
ASAP3	8	903	0.008859358
FARS2	4	451	0.00886918
MSR1	4	451	0.00886918
ADAMTSL3	15	1691	0.008870491
DSC3	8	901	0.008879023
TRIOBP	21	2365	0.008879493
ZKSCAN1	5	563	0.008880995
BCAS2	2	225	0.008888889
CLCF1	2	225	0.008888889
CLDN8	2	225	0.008888889

GSTM3	2	225	0.008888889
PRTFDC1	2	225	0.008888889
TAF1A	4	450	0.008888889
ADAM32	7	787	0.008894536
MMP20	5	562	0.008896797
CAND2	11	1236	0.008899676
ZNF534	6	674	0.008902077
DENND4C	17	1909	0.008905186
AMH	5	560	0.008928571
C16orf54	2	224	0.008928571
CRP	2	224	0.008928571
DSC3	8	896	0.008928571
EPDR1	2	224	0.008928571
KRT32	4	448	0.008928571
MRPL53	1	112	0.008928571
CTNNA3	8	895	0.008938547
IFFO1	5	559	0.008944544
FBXO5	4	447	0.008948546
KAT14	7	782	0.008951407
C10orf120	3	335	0.008955224
OR10R2	3	335	0.008955224
SVEP1	32	3571	0.008961075
LRRC29	2	223	0.00896861
PTPRH	10	1115	0.00896861
RAB37	2	223	0.00896861
SIGMAR1	2	223	0.00896861
AFF3	11	1226	0.008972268
OSTM1	3	334	0.008982036
MMEL1	7	779	0.008985879
WFS1	8	890	0.008988764
PGS1	5	556	0.008992806
BEX5	1	111	0.009009009
COX18	3	333	0.009009009
DDX51	6	666	0.009009009
EID3	3	333	0.009009009
HCRTR2	4	444	0.009009009
LXN	2	222	0.009009009
MAGI1	5	555	0.009009009
NUDT14	2	222	0.009009009
PLEKHB2	2	222	0.009009009
XK	4	444	0.009009009
GPRIN3	7	776	0.009020619
WRNIP1	6	665	0.009022556
ZNF573	6	665	0.009022556
TMEM132C	10	1108	0.009025271
ADORA2B	3	332	0.009036145
ALKBH8	6	664	0.009036145

CNGA2	6	664	0.009036145
HAS3	5	553	0.009041591
SLC45A3	5	553	0.009041591
PTPRO	11	1216	0.009046053
FAM46A	4	442	0.009049774
IZUMO2	2	221	0.009049774
MAL	2	221	0.009049774
MYMK	2	221	0.009049774
TULP3	4	442	0.009049774
ZNF44	6	663	0.009049774
SPTLC3	5	552	0.009057971
DFFA	3	331	0.009063444
NEUROD4	3	331	0.009063444
OR6S1	3	331	0.009063444
RDH13	3	331	0.009063444
ADAMTS10	10	1103	0.009066183
CDH19	7	772	0.009067358
EMID1	4	441	0.009070295
RHBG	4	441	0.009070295
DLGAP4	9	992	0.009072581
CEP68	6	661	0.009077156
GRID2IP	11	1211	0.009083402
C1orf122	1	110	0.009090909
CABP2	2	220	0.009090909
CLDN3	2	220	0.009090909
DGCR6	2	220	0.009090909
IGFL1	1	110	0.009090909
LCE1A	1	110	0.009090909
LCE2B	1	110	0.009090909
LRIT2	5	550	0.009090909
OR11H6	3	330	0.009090909
POMGNT1	6	660	0.009090909
WIPF2	4	440	0.009090909
ZSWIM4	9	989	0.009100101
RPS6KL1	5	549	0.009107468
ZNF18	5	549	0.009107468
FBLN7	4	439	0.009111617
NPHP4	13	1426	0.00911641
DMBT1	22	2413	0.009117281
TRIM55	5	548	0.009124088
DNAH14	32	3507	0.009124608
ADAMTS3	11	1205	0.009128631
CIDEA	2	219	0.00913242
SLC10A5	4	438	0.00913242
FHOD3	13	1422	0.009142053
HCN4	11	1203	0.009143807
CD207	3	328	0.009146341

DLX2	3	328	0.009146341
PINX1	3	328	0.009146341
RAD51D	3	328	0.009146341
SIGLEC15	3	328	0.009146341
TSSK4	3	328	0.009146341
ZBTB49	7	765	0.009150327
ADGRD1	8	874	0.009153318
ZNF154	4	437	0.009153318
FAM124A	5	546	0.009157509
TNFRSF21	6	655	0.009160305
PPP6R3	8	873	0.009163803
TTC16	8	873	0.009163803
CFAP44	17	1854	0.009169364
CD1A	3	327	0.009174312
CLSTN1	9	981	0.009174312
FKBP6	3	327	0.009174312
GRM2	8	872	0.009174312
GSTM4	2	218	0.009174312
GSTM5	2	218	0.009174312
KCNJ14	4	436	0.009174312
OCM	1	109	0.009174312
OR9G4	3	327	0.009174312
RAB42	2	218	0.009174312
ZBED2	2	218	0.009174312
ZNF77	5	545	0.009174312
ACE	12	1306	0.009188361
CBFA2T3	6	653	0.009188361
CHM	6	653	0.009188361
DBR1	5	544	0.009191176
NXPE4	5	544	0.009191176
BCAR1	8	870	0.009195402
T	4	435	0.009195402
AKR1D1	3	326	0.009202454
PEX10	3	326	0.009202454
CSRNP2	5	543	0.009208103
MOB3A	2	217	0.00921659
SLC23A1	6	650	0.009230769
TMED8	3	325	0.009230769
UGT8	5	541	0.009242144
DBN1	6	649	0.009244992
FLRT3	6	649	0.009244992
ASCC2	7	757	0.009247028
HR	11	1189	0.009251472
GINS3	2	216	0.009259259
GVQW2	1	108	0.009259259
NDUFV3	1	108	0.009259259
OR1L3	3	324	0.009259259

OR51D1	3	324	0.009259259
PRG4	13	1404	0.009259259
UBP1	5	540	0.009259259
BAIAP3	11	1187	0.00926706
TYK2	11	1187	0.00926706
SGK1	4	431	0.009280742
SH2D4B	4	431	0.009280742
ADAM7	7	754	0.00928382
ANXA3	3	323	0.009287926
CLEC11A	3	323	0.009287926
FHL1	3	323	0.009287926
MCAM	6	646	0.009287926
PEX7	3	323	0.009287926
SRCIN1	11	1183	0.009298394
NACA	2	215	0.009302326
TCEAL4	2	215	0.009302326
HEATR5A	19	2040	0.009313725
KLHL34	6	644	0.00931677
MRGPRX1	3	322	0.00931677
PHLPP1	16	1717	0.009318579
ACTL6A	4	429	0.009324009
PHC2	8	858	0.009324009
ARL13B	4	428	0.009345794
ATP5J2-PTCD1	7	749	0.009345794
CPTP	2	214	0.009345794
FBL	3	321	0.009345794
LRRC46	3	321	0.009345794
MREG	2	214	0.009345794
N6AMT1	2	214	0.009345794
NVL	8	856	0.009345794
RPL10L	2	214	0.009345794
SFXN3	3	321	0.009345794
SH3BGRL2	1	107	0.009345794
SMDT1	1	107	0.009345794
SMLR1	1	107	0.009345794
TM2D2	2	214	0.009345794
XAB2	8	855	0.009356725
TRPM1	15	1603	0.009357455
UGT1A3	5	534	0.009363296
ZFP69B	5	534	0.009363296
RENBP	4	427	0.009367681
AKR1E2	3	320	0.009375
HNRNPA1L2	3	320	0.009375
OR13C8	3	320	0.009375
PGAP3	3	320	0.009375
UBA2	6	640	0.009375
TTC3	19	2025	0.009382716

LONP1	9	959	0.009384776
ATP5O	2	213	0.009389671
FAM177A1	2	213	0.009389671
RAB25	2	213	0.009389671
STARD5	2	213	0.009389671
ALPPL2	5	532	0.009398496
CSGALNACT1	5	532	0.009398496
ICAM1	5	532	0.009398496
PNPLA1	5	532	0.009398496
DHRS9	3	319	0.009404389
GPA33	3	319	0.009404389
LIMK2	6	638	0.009404389
OR13F1	3	319	0.009404389
CCDC87	8	849	0.00942285
COA3	1	106	0.009433962
NXNL1	2	212	0.009433962
OR13C5	3	318	0.009433962
LRP4	18	1905	0.009448819
SPINT1	5	529	0.009451796
FECH	4	423	0.009456265
FLII	12	1269	0.009456265
ATAD3A	6	634	0.009463722
CC2D1A	9	951	0.009463722
OR2G2	3	317	0.009463722
TNFSF11	3	317	0.009463722
ADARB2	7	739	0.00947226
EFCAB1	2	211	0.009478673
FAM71D	4	422	0.009478673
KYAT1	4	422	0.009478673
P2RX5	4	422	0.009478673
PITHD1	2	211	0.009478673
SOCS1	2	211	0.009478673
SPP2	2	211	0.009478673
DNAJB13	3	316	0.009493671
OR8J1	3	316	0.009493671
TGFBR3L	3	316	0.009493671
ZC3H3	9	948	0.009493671
GYS1	7	737	0.009497965
AEBP1	11	1158	0.009499136
DUSP27	11	1158	0.009499136
CPA3	4	421	0.009501188
TOX	5	526	0.009505703
VPS9D1	6	631	0.009508716
AAGAB	3	315	0.00952381
C10orf128	1	105	0.00952381
CDC42EP2	2	210	0.00952381
CYB5RL	3	315	0.00952381

KRTAP4-7	2	210	0.00952381
LBH	1	105	0.00952381
LRRC75B	3	315	0.00952381
NDUFS8	2	210	0.00952381
RFX4	7	735	0.00952381
RPH3AL	3	315	0.00952381
SNX25	8	840	0.00952381
SPATA8	1	105	0.00952381
ZNF678	5	525	0.00952381
EFCAB7	6	629	0.009538951
OR1E1	3	314	0.00955414
OR9I1	3	314	0.00955414
PKN1	9	942	0.00955414
ANKMY1	9	941	0.009564293
CACNA2D2	11	1150	0.009565217
ANK1	18	1881	0.009569378
DTD1	2	209	0.009569378
LCP1	6	627	0.009569378
PGPEP1	2	209	0.009569378
SCAF11	14	1463	0.009569378
OTOG	28	2925	0.00957265
WDR66	11	1149	0.009573542
PLIN1	5	522	0.009578544
COLGALT2	6	626	0.009584665
OR4M2	3	313	0.009584665
ATP2A3	10	1043	0.009587728
RAG1	10	1043	0.009587728
CPA3	4	417	0.009592326
MTERF3	4	417	0.009592326
GADL1	5	521	0.009596929
ANKS1B	12	1248	0.009615385
IFNE	2	208	0.009615385
KDM8	4	416	0.009615385
OR2M3	3	312	0.009615385
OR6C68	3	312	0.009615385
OR6C74	3	312	0.009615385
OR8K3	3	312	0.009615385
PDXK	3	312	0.009615385
PPRC1	16	1664	0.009615385
S100A14	1	104	0.009615385
SAA2-SAA4	2	208	0.009615385
TMEM222	2	208	0.009615385
ZNF414	3	312	0.009615385
A2ML1	14	1454	0.009628611
LDB3	7	727	0.009628611
C2CD6	6	623	0.009630819
TCP11L2	5	519	0.009633911

TNK2	10	1038	0.009633911
OR7G1	3	311	0.009646302
RNF126	3	311	0.009646302
SOX13	6	622	0.009646302
TMBIM1	3	311	0.009646302
KBTBD4	5	518	0.00965251
EDEM3	9	932	0.009656652
AANAT	2	207	0.009661836
EXD2	6	621	0.009661836
NKAIN1	2	207	0.009661836
PLET1	2	207	0.009661836
SERPINA12	4	414	0.009661836
VEGFB	2	207	0.009661836
ALDH1B1	5	517	0.00967118
SLC4A5	11	1137	0.009674582
ABHD17A	3	310	0.009677419
OR10G2	3	310	0.009677419
OR8B12	3	310	0.009677419
SDC1	3	310	0.009677419
UPP1	3	310	0.009677419
DRC3	7	723	0.009681881
PCDHGB3	9	929	0.009687836
ARFGAP3	5	516	0.009689922
LRRCC1	10	1032	0.009689922
NCKIPSD	7	722	0.009695291
SLCO4A1	7	722	0.009695291
LRGUK	8	825	0.00969697
BCAS3	9	928	0.009698276
BRIP1	1	103	0.009708738
GP1BB	2	206	0.009708738
H4C1	1	103	0.009708738
MRPL58	2	206	0.009708738
OR1A1	3	309	0.009708738
PRR15L	1	103	0.009708738
SLC39A2	3	309	0.009708738
SLURP1	1	103	0.009708738
SOSTDC1	2	206	0.009708738
TCEAL5	2	206	0.009708738
TCTA	1	103	0.009708738
MADD	16	1647	0.009714633
RSF1	14	1441	0.009715475
COL6A6	22	2263	0.009721608
AGXT2	5	514	0.009727626
MCM2	7	719	0.009735744
UIMC1	7	719	0.009735744
ATP6V1B1	5	513	0.009746589
ENDOU	4	410	0.009756098

PMF1	2	205	0.009756098
PPP3CC	5	512	0.009765625
SPNS3	5	512	0.009765625
MAGEF1	3	307	0.009771987
PCDHB1	8	818	0.009779951
TGM1	8	817	0.009791922
LIG1	9	919	0.009793254
ABCC1	15	1531	0.009797518
MYH1	19	1939	0.009798865
AHSP	1	102	0.009803922
DDA1	1	102	0.009803922
DOK5	3	306	0.009803922
HDDC2	2	204	0.009803922
LBH	1	102	0.009803922
LDLRAD4	3	306	0.009803922
LSM3	1	102	0.009803922
LYSMD3	3	306	0.009803922
MXRA7	2	204	0.009803922
NCMAP	1	102	0.009803922
PINLYP	2	204	0.009803922
ZNF764	4	408	0.009803922
THSD4	10	1018	0.009823183
EGLN2	4	407	0.00982801
CA5A	3	305	0.009836066
VWA5B1	12	1220	0.009836066
FCRL2	5	508	0.00984252
ZFYVE26	25	2539	0.009846396
GPR153	6	609	0.009852217
RPL13A	2	203	0.009852217
GRIN3A	11	1115	0.009865471
FAM92B	3	304	0.009868421
SULT1C3	3	304	0.009868421
SPEF2	18	1822	0.009879254
BTBD16	5	506	0.009881423
IFRD2	5	506	0.009881423
ACTR5	6	607	0.009884679
RNF169	7	708	0.009887006
PPFIBP1	10	1011	0.009891197
C9orf47	2	202	0.00990099
KRT33A	4	404	0.00990099
KRT81	5	505	0.00990099
METTL22	4	404	0.00990099
NUDT22	3	303	0.00990099
PECR	3	303	0.00990099
SPARC	3	303	0.00990099
TMEM223	2	202	0.00990099
SPDL1	6	605	0.009917355

SHMT2	5	504	0.009920635
AURKA	4	403	0.009925558
PEX13	4	403	0.009925558
CCDC190	3	302	0.009933775
FBXL18	8	805	0.009937888
CHMP6	2	201	0.009950249
GCNT2	4	402	0.009950249
SAMD12	2	201	0.009950249
ZNF322	4	402	0.009950249
FBLN1	7	703	0.009957326
ADAT1	5	502	0.009960159
BCHE	6	602	0.009966777
ORC1	3	301	0.009966777
TBX15	6	602	0.009966777
SYT8	4	401	0.009975062
LENG9	5	501	0.00998004
PARP12	7	701	0.009985735
ZFHX3	37	3703	0.009991898
ACOX3	7	700	0.01
DEF6	1	100	0.01
HPS1	7	700	0.01
MAK16	3	300	0.01
PLXDC1	5	500	0.01
RNF149	4	400	0.01
SHISA6	5	500	0.01
TCEAL6	2	200	0.01
TMC3	11	1100	0.01
TREML4	2	200	0.01
VAMP8	1	100	0.01
VSTM5	2	200	0.01
ATP10A	15	1499	0.010006671
DSG3	10	999	0.01001001
CAMK2D	5	499	0.01002004
DDIAS	10	998	0.01002004
CASQ2	4	399	0.010025063
LANCL1	4	399	0.010025063
AHSA2	3	299	0.010033445
FCN3	3	299	0.010033445
SLC23A1	6	598	0.010033445
TAS2R5	3	299	0.010033445
TCEA2	3	299	0.010033445
PCDHB11	8	797	0.010037641
AMZ1	5	498	0.010040161
APOL1	4	398	0.010050251
C1orf185	2	199	0.010050251
COMMD2	2	199	0.010050251
GRM1	12	1194	0.010050251

TAGLN2	2	199	0.010050251
GOLIM4	7	696	0.010057471
GPAT2	8	795	0.010062893
BIRC7	3	298	0.010067114
ANKRD44	10	993	0.010070493
MESP2	4	397	0.010075567
PCDHB6	8	794	0.010075567
CERCAM	6	595	0.010084034
UNC13D	11	1090	0.010091743
APOC3	1	99	0.01010101
C19orf81	2	198	0.01010101
CRCT1	1	99	0.01010101
DEFB127	1	99	0.01010101
EDA2R	3	297	0.01010101
ENTPD2	5	495	0.01010101
FCF1	2	198	0.01010101
MPPE1	4	396	0.01010101
NDUFA2	1	99	0.01010101
ITIH3	9	890	0.01011236
DCLRE1C	7	692	0.010115607
ARID3A	6	593	0.010118044
ALDH9A1	5	494	0.010121457
BRF2	5	494	0.010121457
DHODH	4	395	0.010126582
NINL	14	1382	0.010130246
BLVRA	3	296	0.010135135
MMADHC	3	296	0.010135135
TMX2	3	296	0.010135135
SLC12A4	11	1085	0.010138249
CSAD	5	493	0.010141988
GGT6	5	493	0.010141988
MLYCD	5	493	0.010141988
PKDCC	5	493	0.010141988
AARS2	10	985	0.010152284
FZD9	6	591	0.010152284
NKAIN3	2	197	0.010152284
NUDT1	2	197	0.010152284
DPP7	5	492	0.010162602
ATP13A2	12	1180	0.010169492
DUSP15	3	295	0.010169492
GRK5	6	590	0.010169492
PLPP6	3	295	0.010169492
PRR18	3	295	0.010169492
SULT1A2	3	295	0.010169492
TMEM71	3	295	0.010169492
ZNF703	6	590	0.010169492
TP53I13	4	393	0.010178117

KRT39	5	491	0.010183299
ZC3H12C	9	883	0.010192525
C2CD3	24	2353	0.010199745
ARL11	2	196	0.010204082
C11orf94	1	98	0.010204082
CALML4	2	196	0.010204082
CSTA	1	98	0.010204082
CXCL10	1	98	0.010204082
CYP2C18	5	490	0.010204082
IFNL3	2	196	0.010204082
KRTAP3-2	1	98	0.010204082
PATE4	1	98	0.010204082
PLEKHO2	5	490	0.010204082
UTP4	7	686	0.010204082
CDC42EP1	4	391	0.010230179
TSPOAP1	19	1857	0.010231556
TOX2	5	488	0.010245902
TRIM6	5	488	0.010245902
IGF1	2	195	0.01025641
MARCKSL1	2	195	0.01025641
LPCAT3	5	487	0.01026694
FITM1	3	292	0.010273973
NOS1	3	292	0.010273973
MTFMT	4	389	0.010282776
OSCP1	4	389	0.010282776
APBB3	5	486	0.010288066
MATN3	5	486	0.010288066
RUBCN	10	972	0.010288066
ANGPTL5	4	388	0.010309278
B4GALT5	4	388	0.010309278
C17orf58	1	97	0.010309278
C1orf158	2	194	0.010309278
CCDC184	2	194	0.010309278
LRTOMT	3	291	0.010309278
8-Mar	3	291	0.010309278
MCRIP1	1	97	0.010309278
NRDE2	12	1164	0.010309278
SPANXC	1	97	0.010309278
CDC42BPG	16	1551	0.010315925
DHX58	7	678	0.010324484
BMP2K	12	1161	0.010335917
SNX7	4	387	0.010335917
ABCB5	13	1257	0.010342084
ARIH2OS	3	290	0.010344828
NAT1	3	290	0.010344828
MMP20	5	483	0.010351967
ACSM5	6	579	0.010362694

CSRP2	2	193	0.010362694
DDIT4L	2	193	0.010362694
PTGIR	4	386	0.010362694
RHBDD3	4	386	0.010362694
RHOC	2	193	0.010362694
BHLHE41	5	482	0.010373444
ODF3L2	3	289	0.010380623
ACOD1	5	481	0.01039501
RASGRP4	7	673	0.010401189
CERS6	4	384	0.010416667
DUSP5	4	384	0.010416667
DYNLRB1	1	96	0.010416667
DYNLRB2	1	96	0.010416667
GATA2	5	480	0.010416667
KRTAP12-3	1	96	0.010416667
MPP7	6	576	0.010416667
RNF185	2	192	0.010416667
STRBP	7	672	0.010416667
MICALL1	9	863	0.010428737
SH2B3	6	575	0.010434783
DDX19B	5	479	0.010438413
ALPK1	13	1244	0.010450161
CABLES2	5	478	0.010460251
NOP53	5	478	0.010460251
SHPK	5	478	0.010460251
HDAC10	7	669	0.010463378
QPCTL	4	382	0.010471204
ZNF747	2	191	0.010471204
FOXM1	8	763	0.010484928
DSG1	11	1049	0.010486177
ALKBH3	3	286	0.01048951
HAAO	3	286	0.01048951
IFI35	3	286	0.01048951
PAPD5	6	572	0.01048951
TLR5	9	858	0.01048951
DHX34	12	1143	0.010498688
DGCR14	5	476	0.010504202
NOXA1	5	476	0.010504202
C15orf39	11	1047	0.010506208
CES3	6	571	0.010507881
LGR4	10	951	0.010515247
COMMD1	2	190	0.010526316
DHCR7	5	475	0.010526316
EAPP	3	285	0.010526316
LPL	5	475	0.010526316
LRP5	17	1615	0.010526316
MIOX	3	285	0.010526316

OSBPL1A	10	950	0.010526316
PADI3	7	664	0.010542169
HNF4A	5	474	0.010548523
MFSD9	5	474	0.010548523
LYAR	4	379	0.01055409
FNDC1	20	1894	0.010559662
NOX3	6	568	0.01056338
COMP	8	757	0.010568032
DGKE	6	567	0.010582011
DNAJC5G	2	189	0.010582011
IFNA14	2	189	0.010582011
LMF1	6	567	0.010582011
PLA2G12A	2	189	0.010582011
REEP5	2	189	0.010582011
ZNF684	4	378	0.010582011
ATP12A	11	1039	0.010587103
PUS7	7	661	0.010590015
CORO6	5	472	0.01059322
PLCH2	15	1416	0.01059322
SELENBP1	5	472	0.01059322
SENP5	8	755	0.010596026
B4GALNT2	6	566	0.010600707
ANO10	7	660	0.010606061
NEDD4	14	1319	0.010614102
CXCL11	1	94	0.010638298
DUSP18	2	188	0.010638298
EXO1	9	846	0.010638298
FOXL2	4	376	0.010638298
HOXC12	3	282	0.010638298
PNMT	3	282	0.010638298
PRPH	5	470	0.010638298
PSMD13	4	376	0.010638298
SELENOK	1	94	0.010638298
SLC28A2	7	658	0.010638298
TMEM232	7	657	0.01065449
KCNJ14	4	375	0.010666667
SIPA1L3	19	1781	0.010668164
CDRT15L2	3	281	0.010676157
TMPRSS7	9	843	0.010676157
GOLGA3	16	1498	0.010680908
CFAP46	29	2715	0.0106814
TNFRSF10A	5	468	0.010683761
ZNF836	10	936	0.010683761
PLEKHA7	12	1121	0.010704728
CCDC174	5	467	0.010706638
RGS11	5	467	0.010706638
FHL3	3	280	0.010714286

MCM8	9	840	0.010714286
FILIP1	13	1213	0.01071723
LRRC4	7	653	0.010719755
PCDHGA8	10	932	0.010729614
MPP7	6	559	0.010733453
ADAMTS2	13	1211	0.01073493
CCDC81	7	652	0.010736196
GGN	7	652	0.010736196
ARPC1B	4	372	0.010752688
GPC1	6	558	0.010752688
SCGB2A2	1	93	0.010752688
SNX8	5	465	0.010752688
GLI3	17	1580	0.010759494
MGA	20	1857	0.010770059
LSR	7	649	0.010785824
KRTAP5-1	3	278	0.010791367
COL6A2	11	1019	0.010794897
DCLK3	7	648	0.010802469
CIB4	2	185	0.010810811
GJD4	4	370	0.010810811
NSG1	2	185	0.010810811
SMO	6	555	0.010810811
TDRP	2	185	0.010810811
LRRC10	3	277	0.010830325
RIC3	4	369	0.010840108
SLC22A9	6	553	0.01084991
KIAA1324	11	1013	0.010858835
AZIN2	5	460	0.010869565
BDP1	5	460	0.010869565
CITED4	2	184	0.010869565
CNPY1	1	92	0.010869565
FAM151B	3	276	0.010869565
FXYD1	1	92	0.010869565
GALR3	4	368	0.010869565
GNRH1	1	92	0.010869565
LCN15	2	184	0.010869565
S100A12	1	92	0.010869565
SCARB1	6	552	0.010869565
SH3GL1	4	368	0.010869565
TANGO2	3	276	0.010869565
MTMR2	7	643	0.01088647
FGGY	6	551	0.010889292
ZPR1	5	459	0.010893246
APOBEC4	4	367	0.010899183
KIF26B	23	2108	0.010910816
KAT8	5	458	0.010917031
KRT13	5	458	0.010917031

EPN2	7	641	0.010920437
ADAM8	9	824	0.01092233
FRMD1	6	549	0.010928962
FTHL17	2	183	0.010928962
KEL	8	732	0.010928962
APC2	9	822	0.010948905
CCT8	6	548	0.010948905
KDM6B	18	1643	0.010955569
FOXH1	4	365	0.010958904
MAPK13	4	365	0.010958904
HS3ST4	5	456	0.010964912
NRAP	19	1730	0.010982659
CDH24	9	819	0.010989011
DRG2	4	364	0.010989011
HSPB2	2	182	0.010989011
LSM5	1	91	0.010989011
UQCRH	1	91	0.010989011
ZNF80	3	273	0.010989011
ZFAND4	8	727	0.011004127
CPOX	5	454	0.011013216
TMC8	8	726	0.011019284
MAPK15	6	544	0.011029412
ASB16	5	453	0.011037528
TMCO4	7	634	0.011041009
BTD	6	543	0.011049724
KRTAP4-5	2	181	0.011049724
RGS5	2	181	0.011049724
ZNF225	6	543	0.011049724
EHHADH	8	723	0.011065007
ADAM33	9	813	0.011070111
DMRTA2	6	542	0.011070111
TARM1	3	271	0.011070111
VSX2	4	361	0.011080332
NFATC4	10	902	0.011086475
OGFRL1	5	451	0.011086475
SLC22A10	6	541	0.011090573
HNF1A	7	631	0.011093502
BVES	4	360	0.011111111
CCDC115	2	180	0.011111111
CXCR2	4	360	0.011111111
IGF2	2	180	0.011111111
KRT25	5	450	0.011111111
NELL1	9	810	0.011111111
PAX8	5	450	0.011111111
SCGB1D1	1	90	0.011111111
CPSF4L	2	179	0.011173184
FZD4	6	537	0.011173184

HDDC3	2	179	0.011173184
TYRP1	6	537	0.011173184
ZNF516	13	1163	0.011177988
ADCK2	7	626	0.011182109
GRHL3	7	626	0.011182109
LAMC1	18	1609	0.011187073
SCIN	8	715	0.011188811
EAF1	3	268	0.01119403
RASAL1	9	804	0.01119403
IRAK2	7	625	0.0112
LRPAP1	4	357	0.011204482
BTNL9	6	535	0.011214953
KRT79	6	535	0.011214953
MED28	2	178	0.011235955
MRPL13	2	178	0.011235955
NCOA3	16	1424	0.011235955
POMC	3	267	0.011235955
SPECC1	12	1068	0.011235955
SPRR1B	1	89	0.011235955
TBX2	8	712	0.011235955
TFR2	9	801	0.011235955
WFDC9	1	89	0.011235955
PCK1	7	622	0.011254019
GNA14	4	355	0.011267606
GPR1	4	355	0.011267606
IGFLR1	4	355	0.011267606
EPHA2	11	976	0.011270492
CFAP97	6	532	0.011278195
FCAMR	6	532	0.011278195
NPHS1	14	1241	0.011281225
SLCO2B1	8	709	0.011283498
GLIS1	7	620	0.011290323
KLHL32	7	620	0.011290323
CCDC9	6	531	0.011299435
CCT6A	6	531	0.011299435
GNAT3	4	354	0.011299435
GRAMD2A	4	354	0.011299435
IL25	2	177	0.011299435
BUD13	7	619	0.011308562
C5orf22	5	442	0.011312217
CNNM3	8	707	0.011315417
C16orf78	3	265	0.011320755
C5orf15	3	265	0.011320755
PCDHB5	9	795	0.011320755
TK2	3	265	0.011320755
UGT1A8	6	530	0.011320755
S1PR2	4	353	0.011331445

KRT74	6	529	0.011342155
CALD1	9	793	0.011349306
EPB41L1	10	881	0.011350738
MYH13	22	1938	0.011351909
AARS	11	968	0.011363636
CHST11	4	352	0.011363636
HEPN1	1	88	0.011363636
7-Mar	8	704	0.011363636
MOCS2	1	88	0.011363636
NUDT13	4	352	0.011363636
PNOC	2	176	0.011363636
SBSPON	3	264	0.011363636
SCTR	5	440	0.011363636
SH3GL2	4	352	0.011363636
SMPX	1	88	0.011363636
SYCE3	1	88	0.011363636
TMEM41A	3	264	0.011363636
TTC5	5	440	0.011363636
KRI1	8	703	0.011379801
RAG2	6	527	0.011385199
ZIM2	6	527	0.011385199
TTLL9	5	439	0.011389522
ADAT3	4	351	0.011396011
EMILIN2	12	1053	0.011396011
TMEM30B	4	351	0.011396011
SKOR1	11	965	0.011398964
SLC12A5	13	1139	0.011413521
PPFIBP2	10	876	0.011415525
CRYAB	2	175	0.011428571
LAG3	6	525	0.011428571
MTNR1A	4	350	0.011428571
NAB2	6	525	0.011428571
SCAF1	15	1312	0.011432927
LAPTM5	3	262	0.011450382
ZNF138	3	262	0.011450382
GPD1	4	349	0.011461318
RPTN	9	784	0.011479592
AQP8	3	261	0.011494253
CAPG	4	348	0.011494253
CHD5	2	174	0.011494253
COA4	1	87	0.011494253
GJB6	3	261	0.011494253
XBP1	3	261	0.011494253
ZNF664	3	261	0.011494253
D2HGDH	6	521	0.011516315
LRRC69	4	347	0.011527378
OR13C3	4	347	0.011527378

TNS1	20	1735	0.011527378
ACAD11	9	780	0.011538462
BRICD5	3	260	0.011538462
MMP28	6	520	0.011538462
PPIL2	6	520	0.011538462
ROBO3	16	1386	0.011544012
SMYD2	5	433	0.011547344
B4GALNT4	12	1039	0.011549567
AMOTL2	9	779	0.011553273
BBS4	6	519	0.011560694
CDK7	4	346	0.011560694
CRYAA	2	173	0.011560694
CRYAA	2	173	0.011560694
GCSH	2	173	0.011560694
HS3ST5	4	346	0.011560694
MYL5	2	173	0.011560694
PAQR7	4	346	0.011560694
WDR36	11	951	0.011566772
PCM1	7	605	0.011570248
KRTAP10-8	3	259	0.011583012
ASTL	5	431	0.011600928
ASCL4	2	172	0.011627907
CCRL2	4	344	0.011627907
CETN1	2	172	0.011627907
CYP1A2	6	516	0.011627907
FGFBP3	3	258	0.011627907
LPAR6	4	344	0.011627907
WDR45B	4	344	0.011627907
ZNF83	6	516	0.011627907
NID2	16	1375	0.011636364
MYOF	24	2061	0.011644833
PPP2R1B	7	601	0.011647255
SMAP1	11	944	0.011652542
RHPN2	8	686	0.011661808
APH1B	3	257	0.011673152
GSC	3	257	0.011673152
GZMM	3	257	0.011673152
ACP6	5	428	0.011682243
GCNT1	5	428	0.011682243
MTUS2	16	1369	0.011687363
BMP6	6	513	0.011695906
CAPN14	8	684	0.011695906
HEATR4	12	1026	0.011695906
HS3ST5	4	342	0.011695906
HS3ST6	4	342	0.011695906
VPS41	10	854	0.011709602
AKT1S1	3	256	0.01171875

CYP1A1	6	512	0.01171875
PRTN3	3	256	0.01171875
DCAF8	7	597	0.011725293
BBS5	4	341	0.011730205
CD2BP2	4	341	0.011730205
ETV7	4	341	0.011730205
LIMS2	4	341	0.011730205
FCRL2	5	426	0.011737089
HSF5	7	596	0.011744966
CCDC151	7	595	0.011764706
COMMD6	1	85	0.011764706
FGF22	2	170	0.011764706
TOGARAM2	12	1019	0.011776251
KIF1C	13	1103	0.011786038
DNTT	6	509	0.011787819
PLA2G3	6	509	0.011787819
SLC16A9	6	509	0.011787819
PTPN14	14	1187	0.01179444
KCNQ5	11	932	0.011802575
EPS8L3	7	593	0.011804384
NRP2	11	931	0.011815252
TSKS	7	592	0.011824324
BNC2	13	1099	0.011828935
BRF1	4	338	0.01183432
CAD	4	338	0.01183432
DDIT3	2	169	0.01183432
PHYH	4	338	0.01183432
SPRR3	2	169	0.01183432
TAF3	11	929	0.011840689
TMC1	9	760	0.011842105
C16orf70	5	422	0.011848341
ZNF550	5	422	0.011848341
HSPA12A	8	675	0.011851852
C1orf43	3	253	0.011857708
PCDHGB1	11	927	0.011866235
SLC9A3R2	4	337	0.011869436
C2CD4C	5	421	0.011876485
OTUD7A	11	926	0.01187905
BLK	6	505	0.011881188
ANAPC11	1	84	0.011904762
ATP5D	2	168	0.011904762
DMRTA1	6	504	0.011904762
HSPA4	10	840	0.011904762
KANK3	10	840	0.011904762
PEX16	4	336	0.011904762
TRIM29	7	588	0.011904762
XRCC4	4	336	0.011904762

HSPA4L	10	839	0.011918951
TAS1R2	10	839	0.011918951
PPM1E	9	755	0.01192053
BRF2	5	419	0.011933174
FAH	5	419	0.011933174
FDPS	5	419	0.011933174
NDE1	4	335	0.011940299
TCERG1L	7	586	0.011945392
CNOT3	9	753	0.011952191
SPATA5L1	9	753	0.011952191
OAS3	13	1087	0.011959522
AVPR1A	5	418	0.011961722
FBXL14	5	418	0.011961722
MPP3	7	585	0.011965812
CNMD	4	334	0.011976048
MRPS24	2	167	0.011976048
TCF24	2	167	0.011976048
ZIC4	4	334	0.011976048
SARDH	11	918	0.011982571
SPTA1	29	2419	0.011988425
SERPINA9	5	417	0.011990408
STRIP2	10	834	0.011990408
CFAP57	15	1250	0.012
ALCAM	7	583	0.012006861
ELP1	16	1332	0.012012012
ZNF333	8	665	0.012030075
C2orf68	2	166	0.012048193
KRTAP21-2	1	83	0.012048193
KRTAP4-4	2	166	0.012048193
MAMSTR	5	415	0.012048193
RAP1GAP	8	663	0.012066365
SCML4	5	414	0.012077295
VSIG8	5	414	0.012077295
SPON2	4	331	0.012084592
SEZ6L2	11	910	0.012087912
CARNS1	10	827	0.012091898
MRO	3	248	0.012096774
CLUAP1	5	413	0.012106538
HAS1	7	578	0.012110727
KRT77	7	578	0.012110727
TTBK1	16	1321	0.012112036
ENGASE	9	743	0.012113055
CWC22	11	908	0.012114537
C14orf80	6	495	0.012121212
FAM86C1	2	165	0.012121212
NRN1L	2	165	0.012121212
COLEC12	9	742	0.01212938

ACADS	5	412	0.012135922
PRR30	5	412	0.012135922
ZNF304	8	659	0.012139605
ARHGAP19	6	494	0.012145749
ACSF3	7	576	0.012152778
CNN3	4	329	0.012158055
RNF225	4	329	0.012158055
ANO1	12	986	0.012170385
FAN1	6	493	0.012170385
HHAT	6	493	0.012170385
CES5A	7	575	0.012173913
CNGA4	7	575	0.012173913
FAM198A	7	575	0.012173913
LETM1	9	739	0.01217862
FRS3	6	492	0.012195122
KANSL2	6	492	0.012195122
LRRC43	8	656	0.012195122
MUSTN1	1	82	0.012195122
PHGR1	1	82	0.012195122
PMM2	3	246	0.012195122
SGK494	5	410	0.012195122
SVOPL	6	492	0.012195122
TSPYL6	5	410	0.012195122
HGFAC	8	655	0.01221374
KIAA1257	5	409	0.012224939
PLEKHO1	5	409	0.012224939
ART1	4	327	0.012232416
ZYX	7	572	0.012237762
PIK3C2B	20	1634	0.012239902
CYP2C8	6	490	0.012244898
EIF3A	3	245	0.012244898
METTL27	3	245	0.012244898
OCIAD1	3	245	0.012244898
USP37	12	979	0.012257406
KLHL28	7	571	0.012259194
OR10X1	4	326	0.012269939
EPB41L5	9	733	0.012278308
ZFP91	7	570	0.012280702
METTL7B	3	244	0.012295082
ZNF273	7	569	0.012302285
DBP	4	325	0.012307692
OR52E2	4	325	0.012307692
PLPPR1	4	325	0.012307692
ZC2HC1A	4	325	0.012307692
EFCAB13	12	973	0.012332991
ATP6V0E2	1	81	0.012345679
LCTL	7	567	0.012345679

OR6K2	4	324	0.012345679
TFDP3	5	405	0.012345679
GEMIN8	3	242	0.012396694
STAP2	5	403	0.012406948
ATR	7	564	0.012411348
MRGPRX4	4	322	0.01242236
TAF15	2	161	0.01242236
LHX5	5	402	0.012437811
GSTO1	3	241	0.012448133
NGF	3	241	0.012448133
TNFRSF18	3	241	0.012448133
OR3A2	4	321	0.012461059
OR51G1	4	321	0.012461059
OR51V1	4	321	0.012461059
TOR2A	4	321	0.012461059
ADAMTSL5	6	481	0.012474012
EFS	7	561	0.012477718
TDRKH	7	561	0.012477718
CDC6	7	560	0.0125
EFHC1	8	640	0.0125
METTL12	3	240	0.0125
MFSD12	6	480	0.0125
RNASE3	2	160	0.0125
UPK3B	4	320	0.0125
PCDHB2	10	798	0.012531328
VSIG4	5	399	0.012531328
UGGT2	19	1516	0.012532982
OGFOD3	4	319	0.012539185
OR7C2	4	319	0.012539185
ZP1	8	638	0.012539185
AHI1	15	1196	0.012541806
COL18A1	22	1754	0.012542759
C9orf152	3	239	0.012552301
DAP3	5	398	0.012562814
S1PR5	5	398	0.012562814
SAXO2	5	398	0.012562814
TJAP1	7	557	0.012567325
DPPA3	2	159	0.012578616
SDHD	2	159	0.012578616
GATA5	5	397	0.012594458
BPIFB3	6	476	0.012605042
MYH7B	25	1983	0.012607161
TRPC1	10	793	0.01261034
KCNQ3	11	872	0.012614679
DHRS12	4	317	0.012618297
OR10A5	4	317	0.012618297
OR2F1	4	317	0.012618297

OR51Q1	4	317	0.012618297
RFPL3	4	317	0.012618297
SFRP5	4	317	0.012618297
SLC35A2	5	396	0.012626263
ZBTB10	11	871	0.012629162
TRPM2	19	1503	0.012641384
FHOD3	13	1028	0.012645914
EGFL6	7	553	0.012658228
EPN3	8	632	0.012658228
MORN2	1	79	0.012658228
ST20	1	79	0.012658228
XKR8	5	395	0.012658228
COL5A2	19	1499	0.012675117
RPN2	8	631	0.012678288
CDC25C	6	473	0.012684989
MSLN	8	630	0.012698413
OR10H5	4	315	0.012698413
OR3A1	4	315	0.012698413
BIRC8	3	236	0.012711864
STX8	3	236	0.012711864
DMPK	8	629	0.012718601
TLR1	10	786	0.012722646
SLC11A1	7	550	0.012727273
EPB41	11	864	0.012731481
CPPED1	4	314	0.012738854
DUSP2	4	314	0.012738854
EPCAM	4	314	0.012738854
OR5B12	4	314	0.012738854
TMEM50A	2	157	0.012738854
CCDC39	12	941	0.012752391
RBMXL2	5	392	0.012755102
TLR2	10	784	0.012755102
DCSTAMP	6	470	0.012765957
C11orf65	4	313	0.012779553
RIMKLA	5	391	0.012787724
SOX1	5	391	0.012787724
GBA3	6	469	0.012793177
MMP1	6	469	0.012793177
SEC24C	14	1094	0.012797075
ATG7	9	703	0.012802276
CDHR1	11	859	0.012805588
HTR1B	5	390	0.012820513
LYRM9	1	78	0.012820513
MMP21	5	390	0.012820513
MUTYH	7	546	0.012820513
OR2M7	4	312	0.012820513
OR4F15	4	312	0.012820513

OR52A1	4	312	0.012820513
OR6C1	4	312	0.012820513
OR6C2	4	312	0.012820513
RNASE1	2	156	0.012820513
SCP2D1	2	156	0.012820513
TGFB1	5	390	0.012820513
PLEKHA4	10	779	0.01283697
MMP8	6	467	0.012847966
SYTL2	12	934	0.012847966
TRIT1	6	467	0.012847966
ATP6V0A2	11	856	0.012850467
ANKRD42	5	389	0.01285347
MTCH1	5	389	0.01285347
NOL8	15	1167	0.01285347
OR10G9	4	311	0.012861736
SYNGR1	3	233	0.012875536
UNC5C	12	931	0.012889366
ID1	2	155	0.012903226
MKNK1	6	465	0.012903226
ONECUT1	6	465	0.012903226
OR4C16	4	310	0.012903226
SEMA6C	12	930	0.012903226
ZNF100	7	542	0.012915129
DENND2C	12	928	0.012931034
ISM1	6	464	0.012931034
MIXL1	3	232	0.012931034
SRPX	6	464	0.012931034
VEGFA	3	232	0.012931034
FTCD	7	541	0.012939002
TTLL8	11	850	0.012941176
SORCS2	15	1159	0.012942192
BIRC2	8	618	0.012944984
FAM26E	4	309	0.012944984
IRGC	6	463	0.012958963
MIEF1	6	463	0.012958963
PI16	6	463	0.012958963
ECM1	7	540	0.012962963
TMPO	9	694	0.0129683
APLN	1	77	0.012987013
BPNT1	4	308	0.012987013
CRIP1	1	77	0.012987013
FAM162A	2	154	0.012987013
OCIAD2	2	154	0.012987013
PLEKHG2	18	1386	0.012987013
ZBTB7B	7	539	0.012987013
CLOCK	11	846	0.013002364
AJUBA	7	538	0.013011152

SNTB1	7	538	0.013011152
FAM71E2	12	922	0.013015184
AIPL1	5	384	0.013020833
NMNAT2	4	307	0.013029316
ADAMTSL4	14	1074	0.013035382
ADGRL4	9	690	0.013043478
DHRS4L2	3	230	0.013043478
TREM2	3	230	0.013043478
CCDC116	8	613	0.013050571
LPP	8	612	0.013071895
MAL	2	153	0.013071895
ARHGEF15	11	841	0.013079667
HKDC1	12	917	0.01308615
ANKRD24	15	1146	0.013089005
SLC26A3	10	764	0.013089005
HNRNPA0	4	305	0.013114754
OR4X1	4	305	0.013114754
PEX2	4	305	0.013114754
GANC	12	914	0.013129103
UNC93A	6	457	0.013129103
GALC	9	685	0.013138686
BRINP1	10	761	0.013140604
MYBPC2	15	1141	0.013146363
CYB5D1	3	228	0.013157895
MRPL38	5	380	0.013157895
NDUFC1	1	76	0.013157895
NEU2	5	380	0.013157895
RABL2A	3	228	0.013157895
NLGN2	11	835	0.013173653
RBM28	10	759	0.013175231
PKM	7	531	0.013182674
ABCD4	8	606	0.01320132
AFMID	4	303	0.01320132
SEC22C	4	303	0.01320132
EFHB	11	833	0.013205282
CHST2	7	530	0.013207547
ZNRF1	3	227	0.013215859
ACAD10	14	1059	0.013220019
METTL2B	5	378	0.013227513
GPR161	7	529	0.013232514
ALDH3A1	6	453	0.013245033
DHRS3	4	302	0.013245033
DLST	6	453	0.013245033
ME3	8	604	0.013245033
MYL6	2	151	0.013245033
SULT1C4	4	302	0.013245033
FAM81B	6	452	0.013274336

MRVI1	12	904	0.013274336
PAH	6	452	0.013274336
HAVCR2	4	301	0.013289037
NOXO1	5	376	0.013297872
SIDT1	11	827	0.013301088
DLGAP1	13	977	0.013306039
GDF7	6	450	0.013333333
GNG4	1	75	0.013333333
PDIA2	7	525	0.013333333
PRODH2	8	600	0.013333333
DMWD	9	674	0.013353116
NUP58	8	599	0.013355593
PCOLCE	6	449	0.013363029
PALM3	9	673	0.013372957
DNASE1L2	4	299	0.013377926
DRC3	7	523	0.013384321
APOBEC2	3	224	0.013392857
ATP6AP1L	3	224	0.013392857
KLHL21	8	597	0.013400335
CMKLR1	5	373	0.013404826
DIAPH3	16	1193	0.013411567
FAM221A	4	298	0.013422819
ACTRT3	5	372	0.01344086
MKRN2OS	3	223	0.013452915
PSMD9	3	223	0.013452915
DYSF	28	2080	0.013461538
KLHDC4	7	520	0.013461538
UBXN11	7	520	0.013461538
FNIP2	15	1114	0.013464991
MC2R	4	297	0.013468013
NRP2	11	816	0.013480392
PRRC1	6	445	0.013483146
EPHA5	14	1037	0.013500482
C2orf40	2	148	0.013513514
CHAC1	3	222	0.013513514
DRC1	10	740	0.013513514
GNGT1	1	74	0.013513514
GSTA5	3	222	0.013513514
IQCF5	2	148	0.013513514
NEMP1	6	444	0.013513514
SMIM15	1	74	0.013513514
C9orf66	4	295	0.013559322
SULT1A1	4	295	0.013559322
GPX6	3	221	0.013574661
HIST1H1D	3	221	0.013574661
SDF2L1	3	221	0.013574661
TIRAP	3	221	0.013574661

PRR5L	5	368	0.013586957
ASB15	8	588	0.013605442
C1orf100	2	147	0.013605442
CAMKK2	8	588	0.013605442
HBD	2	147	0.013605442
PPP1R14A	2	147	0.013605442
CRTAC1	9	661	0.013615734
AHSG	5	367	0.013623978
WFDC1	3	220	0.013636364
ZCCHC4	7	513	0.013645224
MYOM2	20	1465	0.013651877
GAA	13	952	0.013655462
ZNF254	9	659	0.013657056
TSNAXIP1	9	658	0.013677812
AMY2B	7	511	0.01369863
GNG11	1	73	0.01369863
MED25	2	146	0.01369863
PAGE1	2	146	0.01369863
POLG	17	1239	0.013720743
HGF	10	728	0.013736264
PARVB	5	364	0.013736264
SSTR5	5	364	0.013736264
ITIH2	13	946	0.013742072
SLC17A9	6	436	0.013761468
ZNF101	6	436	0.013761468
ARHGEF26	12	871	0.013777268
SCARF2	12	871	0.013777268
FARSA	7	508	0.013779528
CLCA2	13	943	0.01378579
C21orf59	4	290	0.013793103
GNLY	2	145	0.013793103
CPZ	9	652	0.013803681
CYHR1	5	362	0.013812155
ZC3H4	18	1303	0.013814275
YY1AP1	11	796	0.013819095
HOXB7	3	217	0.013824885
RGS19	3	217	0.013824885
DHX37	16	1157	0.013828868
1-Mar	4	289	0.01384083
TEX26	4	289	0.01384083
ATXN3	5	361	0.013850416
SLC22A16	8	577	0.013864818
ZBP1	8	577	0.013864818
AHCY	6	432	0.013888889
FGFRL1	7	504	0.013888889
HHATL	7	504	0.013888889
STX2	4	288	0.013888889

GRID2	14	1007	0.013902681
IL17RC	11	791	0.013906448
APBA3	8	575	0.013913043
ST7L	8	575	0.013913043
HSPBP1	5	359	0.013927577
SORBS1	18	1292	0.013931889
CYP3A5	7	502	0.013944223
LTBP1	24	1721	0.013945381
RELT	6	430	0.013953488
SPACA3	3	215	0.013953488
TLDC2	3	215	0.013953488
ATP13A5	17	1218	0.013957307
CLN5	5	358	0.01396648
TMEM132E	15	1074	0.01396648
TRIB3	5	358	0.01396648
CHRDL2	6	429	0.013986014
CYBRD1	4	286	0.013986014
TET2	28	2002	0.013986014
ATRN	20	1429	0.013995801
LIPG	7	500	0.014
WDR92	5	357	0.014005602
RUFY4	8	571	0.014010508
PTK7	15	1070	0.014018692
ZNF708	7	499	0.014028056
FKBP9	8	570	0.014035088
NEURL2	4	285	0.014035088
PIF1	9	641	0.014040562
PCK2	9	640	0.0140625
C12orf73	1	71	0.014084507
GTPBP8	4	284	0.014084507
MORN1	7	497	0.014084507
TGM7	10	710	0.014084507
NRG2	12	850	0.014117647
POLR3B	16	1133	0.014121801
KIRREL2	10	708	0.014124294
MYBPHL	5	354	0.014124294
SLC14A2	13	920	0.014130435
PDX1	4	283	0.014134276
MNS1	7	495	0.014141414
ELP4	6	424	0.014150943
RAB17	3	212	0.014150943
PLEK2	5	353	0.014164306
CBARP	10	705	0.014184397
MMACHC	4	282	0.014184397
OSCAR	4	282	0.014184397
TMEM242	2	141	0.014184397
TNFRSF19	6	423	0.014184397

OSBP2	13	916	0.01419214
MKX	5	352	0.014204545
TRAP1	10	704	0.014204545
HCN3	11	774	0.014211886
CLDN1	3	211	0.014218009
PRR22	6	422	0.014218009
RHOF	3	211	0.014218009
USP20	13	914	0.014223195
PASD1	11	773	0.014230272
EEF1D	4	281	0.014234875
PTCRA	4	281	0.014234875
CYP2F1	7	491	0.014256619
ZNF655	7	491	0.014256619
CCDC153	3	210	0.014285714
CERS1	5	350	0.014285714
GYG1	5	350	0.014285714
MAN1C1	9	630	0.014285714
PFN1	2	140	0.014285714
TPD52L3	2	140	0.014285714
TSPAN31	3	210	0.014285714
DRD4	6	419	0.014319809
DCDC2B	5	349	0.014326648
SRMS	7	488	0.014344262
KIF26A	27	1882	0.01434644
C19orf71	3	209	0.014354067
GUCA1C	3	209	0.014354067
TM4SF19	3	209	0.014354067
MRPL3	5	348	0.014367816
C1QTNF6	4	278	0.014388489
CNIH4	2	139	0.014388489
CPB1	6	417	0.014388489
LGALS13	2	139	0.014388489
SPATA33	2	139	0.014388489
WNT10A	6	417	0.014388489
MN1	19	1320	0.014393939
TFEC	5	347	0.014409222
FBXO33	8	555	0.014414414
ZDHHC1	7	485	0.01443299
CBR1	4	277	0.014440433
PRPH	5	346	0.014450867
NMUR2	6	415	0.014457831
TCIRG1	12	830	0.014457831
PEAR1	15	1037	0.014464802
PGM2L1	9	622	0.014469453
EXOSC8	4	276	0.014492754
KLRF2	3	207	0.014492754
MRPS27	6	414	0.014492754

POM121L2	15	1035	0.014492754
SLC22A24	8	552	0.014492754
SLC36A2	7	483	0.014492754
TNP2	2	138	0.014492754
TVP23C	4	276	0.014492754
UCMA	2	138	0.014492754
CDHR2	19	1310	0.014503817
LOXHD1	30	2067	0.014513788
KRT75	8	551	0.014519056
RASSF1	5	344	0.014534884
PTH2R	8	550	0.014545455
MXD3	3	206	0.014563107
PCSK4	11	755	0.014569536
MASP2	10	686	0.014577259
RBP5	2	137	0.01459854
RPL28	2	137	0.01459854
SOX30	11	753	0.014608234
CNPPD1	6	410	0.014634146
DPYD	15	1025	0.014634146
SPATA24	3	205	0.014634146
TMEM132A	15	1023	0.014662757
OLFML2B	11	750	0.014666667
RHAG	6	409	0.014669927
A3GALT2	5	340	0.014705882
CCDC179	1	68	0.014705882
CHAT	11	748	0.014705882
CHIA	7	476	0.014705882
H3C1	2	136	0.014705882
MMP10	7	476	0.014705882
NREP	1	68	0.014705882
LRIT3	10	679	0.014727541
PATL2	8	543	0.014732965
EMILIN3	14	949	0.014752371
PLPP7	4	271	0.014760148
AFP	9	609	0.014778325
RNF152	3	203	0.014778325
TDO2	6	406	0.014778325
ZPBP2	5	338	0.014792899
PLCZ1	9	608	0.014802632
RBP5	2	135	0.014814815
SLC39A5	8	540	0.014814815
ADGRF3	16	1079	0.014828545
CLASRP	10	674	0.014836795
DCPS	5	337	0.014836795
EXD3	13	876	0.014840183
SNTG2	8	539	0.014842301
NR2F6	6	404	0.014851485

PTTG2	3	202	0.014851485
AP5Z1	12	807	0.014869888
C8orf34	8	538	0.014869888
TNNT3	4	269	0.014869888
ZNF559	8	538	0.014869888
GRIP1	5	336	0.014880952
PAN2	5	336	0.014880952
TBC1D21	5	336	0.014880952
SLC36A3	7	470	0.014893617
PRKG1	10	671	0.01490313
C2orf70	3	201	0.014925373
CCDC114	10	670	0.014925373
CPLX1	2	134	0.014925373
FGF5	4	268	0.014925373
HOXA1	5	335	0.014925373
MOGAT1	5	335	0.014925373
PRODH2	8	536	0.014925373
SLC13A3	9	602	0.014950166
CCDC191	14	936	0.014957265
KRTAP10-4	6	401	0.014962594
WDR54	5	334	0.01497006
KLK14	4	267	0.014981273
MRPL9	4	267	0.014981273
PNLIPRP1	7	467	0.014989293
BLZF1	6	400	0.015
CATSPERZ	3	200	0.015
MED22	3	200	0.015
ZNF543	9	600	0.015
PTGS1	9	599	0.015025042
ANKRD45	4	266	0.015037594
DKK1	4	266	0.015037594
FGFR1OP	6	399	0.015037594
LEF1	6	399	0.015037594
EFCC1	9	598	0.015050167
LAX1	6	398	0.015075377
PNMT	3	199	0.015075377
ARHGAP8	7	464	0.015086207
PDLIM5	9	596	0.015100671
SHOX2	5	331	0.01510574
PIK3R2	11	728	0.01510989
F2RL1	6	397	0.01511335
PRDM2	26	1718	0.015133877
GPR142	7	462	0.015151515
KLHDC7B	9	594	0.015151515
LGALS2	2	132	0.015151515
OCEL1	4	264	0.015151515
SLC22A14	9	594	0.015151515

CCDC7	21	1385	0.015162455
SLC5A4	10	659	0.015174507
PNPLA7	20	1317	0.015186029
GRASP	6	395	0.015189873
VN1R2	6	395	0.015189873
SBSN	9	590	0.015254237
SDR42E1	6	393	0.015267176
AGBL1	17	1112	0.01528777
GLYCTK	8	523	0.015296367
TTYH3	8	523	0.015296367
ZNF599	9	588	0.015306122
FARP1	16	1045	0.015311005
ALKBH2	4	261	0.01532567
CLDN18	4	261	0.01532567
RIN1	12	783	0.01532567
GSN	12	782	0.015345269
METTL17	7	456	0.015350877
PKD1	14	912	0.015350877
CYP4F3	8	520	0.015384615
FAM122C	3	195	0.015384615
FAM83D	9	585	0.015384615
MC5R	5	325	0.015384615
RD3	3	195	0.015384615
SERPINB3	6	390	0.015384615
SLC2A14	8	520	0.015384615
TMEM70	4	260	0.015384615
TTC6	8	520	0.015384615
ADCY6	18	1168	0.015410959
10-Sep	7	454	0.015418502
TTC27	13	843	0.015421115
ABCB6	13	842	0.01543943
RUNX1	7	453	0.015452539
UQCRC2	7	453	0.015452539
MMP14	9	582	0.015463918
TMEM59	5	323	0.015479876
DCBLD2	12	775	0.015483871
SOCS7	9	581	0.015490534
GALR2	6	387	0.015503876
TRIM45	9	580	0.015517241
REEP4	4	257	0.015564202
PIGT	9	578	0.015570934
ZKSCAN8	9	578	0.015570934
TPSG1	5	321	0.015576324
SLFN13	14	897	0.015607581
KRBOX1	2	128	0.015625
LRTOMT	3	192	0.015625
MRPL55	2	128	0.015625

NOP10	1	64	0.015625
SLC7A7	8	511	0.015655577
DLK2	6	383	0.015665796
ACOT8	5	319	0.015673981
MCC	13	829	0.015681544
H1FNT	4	255	0.015686275
BST1	5	318	0.01572327
RDH11	5	318	0.01572327
MRFAP1L1	2	127	0.015748031
NFU1	4	254	0.015748031
DRD2	7	443	0.015801354
PGAM2	4	253	0.015810277
MYBBP1A	21	1328	0.015813253
CR1L	9	569	0.015817223
OR10A7	5	316	0.015822785
EME2	6	379	0.015831135
CHRNA3	8	505	0.015841584
CLTCL1	26	1640	0.015853659
ATP2C2	15	946	0.015856237
CCDC74A	6	378	0.015873016
OR5M1	5	315	0.015873016
TMEM175	8	504	0.015873016
LCAT	7	440	0.015909091
MUC7	6	377	0.015915119
NDUFA9	6	377	0.015915119
HADH	5	314	0.015923567
OR51I1	5	314	0.015923567
WDR38	5	314	0.015923567
USP21	9	565	0.015929204
PPEF2	12	753	0.015936255
ACOT9	7	439	0.01594533
MYC	7	439	0.01594533
MRPL35	3	188	0.015957447
OVCH2	9	564	0.015957447
PDHX	8	501	0.015968064
IGFL3	2	125	0.016
MEIS3	6	375	0.016
WISP2	4	250	0.016
PLIN2	7	437	0.016018307
CARMIL3	22	1372	0.016034985
FAM184B	17	1060	0.016037736
GPX6	3	187	0.016042781
CPA5	7	436	0.016055046
STAMBPL1	7	436	0.016055046
CLN6	5	311	0.01607717
OR4D10	5	311	0.01607717
AIFM2	6	373	0.016085791

TEKT4	7	435	0.016091954
JUP	12	745	0.016107383
GPI	9	558	0.016129032
PACRGL	4	248	0.016129032
RANGRF	3	186	0.016129032
TAX1BP3	2	124	0.016129032
TEAD4	7	434	0.016129032
TSPAN18	4	248	0.016129032
ZFPL1	5	310	0.016129032
ZNF366	12	744	0.016129032
LRRC2	6	371	0.016172507
NPSR1	6	371	0.016172507
GPR35	5	309	0.01618123
KCNK16	5	309	0.01618123
OR8B4	5	309	0.01618123
SLC25A41	6	370	0.016216216
SLC25A47	5	308	0.016233766
SEMA4D	14	862	0.016241299
HK3	15	923	0.016251354
FSCN2	8	492	0.016260163
PSMG4	2	123	0.016260163
ACADL	7	430	0.01627907
TEKT2	7	430	0.01627907
MYO1A	17	1043	0.016299137
CHAC2	3	184	0.016304348
IMP3	3	184	0.016304348
PRKRIP1	3	184	0.016304348
TNFRSF13C	3	184	0.016304348
UPK2	3	184	0.016304348
USH1C	9	552	0.016304348
TNIP2	7	429	0.016317016
EIF6	4	245	0.016326531
STEAP2	8	490	0.016326531
SPICE1	14	855	0.016374269
CES1	1	61	0.016393443
LEFTY2	6	366	0.016393443
LMO7DN	2	122	0.016393443
LRRC25	5	305	0.016393443
XRRA1	13	792	0.016414141
SLCO1A2	11	670	0.01641791
FCRLB	7	426	0.016431925
RRS1	6	365	0.016438356
ZDHHC20	6	365	0.016438356
SELENOO	11	669	0.016442451
SLC25A38	5	304	0.016447368
MON1B	9	547	0.016453382
LAT2	4	243	0.016460905

CD33	6	364	0.016483516
MAP4	19	1152	0.016493056
PDCD7	8	485	0.016494845
LGI2	9	545	0.016513761
MRPS7	4	242	0.016528926
TOR2A	4	242	0.016528926
SULT1C2	5	302	0.016556291
ALOX15	11	662	0.016616314
FSTL4	14	842	0.016627078
CCT5	9	541	0.01663586
AMPD1	13	780	0.016666667
ATR	7	420	0.016666667
PFKL	13	780	0.016666667
POP1	6	360	0.016666667
SMIM3	1	60	0.016666667
UQCRC1	8	480	0.016666667
VSIG10	9	540	0.016666667
CAPN12	12	719	0.016689847
GCAT	7	419	0.016706444
ABCA4	38	2273	0.016717994
CARMIL2	24	1435	0.016724739
OLFM3	8	478	0.016736402
ARRDC4	7	418	0.016746411
CERK	9	537	0.016759777
KRTAP10-11	5	298	0.016778523
BNIPL	6	357	0.016806723
P2RX7	10	595	0.016806723
TCF3	11	654	0.016819572
ACAD8	7	415	0.01686747
C14orf105	5	296	0.016891892
MRPS2	5	296	0.016891892
SULT1C2	5	296	0.016891892
KRT16	8	473	0.016913319
ESPNL	17	1005	0.016915423
MTMR11	12	709	0.016925247
CLIC3	4	236	0.016949153
EVI2A	4	236	0.016949153
NEGR1	6	354	0.016949153
TMEM190	3	177	0.016949153
TREX2	4	236	0.016949153
ZIM3	8	472	0.016949153
RAPSN	7	412	0.016990291
VWA3B	22	1294	0.017001546
RARRES1	5	294	0.017006803
SLFN12L	10	588	0.017006803
TMEM249	4	235	0.017021277
DFNB59	6	352	0.017045455

DUSP28	3	176	0.017045455
GPATCH2	9	528	0.017045455
KNG1	11	644	0.017080745
PISD	7	409	0.017114914
SLC26A1	12	701	0.017118402
FAM20C	10	584	0.017123288
SDCBP2	5	292	0.017123288
MYL7	3	175	0.017142857
PLEK	6	350	0.017142857
ATL2	10	583	0.017152659
BCL10	4	233	0.017167382
ART5	5	291	0.017182131
SHC2	10	582	0.017182131
PLCD1	13	756	0.017195767
ADAMTS5	16	930	0.017204301
PRKN	8	465	0.017204301
PINK1	10	581	0.017211704
FBRSL1	18	1045	0.01722488
C2orf49	4	232	0.017241379
DNAJC19	2	116	0.017241379
FBXW12	8	464	0.017241379
NDUFB1	1	58	0.017241379
NTN3	10	580	0.017241379
RSRP1	5	290	0.017241379
SEC14L4	7	406	0.017241379
SST	2	116	0.017241379
LSM14A	8	463	0.017278618
GBGT1	6	347	0.017291066
PURG	6	347	0.017291066
KLHL30	10	578	0.017301038
CYP4A22	9	519	0.01734104
FAM180A	3	173	0.01734104
PLPP2	5	288	0.017361111
SYT7	7	403	0.017369727
FAM98A	9	518	0.017374517
MOSPD2	9	518	0.017374517
C11orf86	2	115	0.017391304
GUCA2A	2	115	0.017391304
MIF	2	115	0.017391304
TAAR6	6	345	0.017391304
TMEM144	6	345	0.017391304
ZNF587	10	575	0.017391304
RABEP1	15	862	0.017401392
POMT1	13	747	0.017402945
BMP8B	7	402	0.017412935
TSR1	14	804	0.017412935
STX11	5	287	0.017421603

B4GALT4	6	344	0.01744186
CCNG2	6	344	0.01744186
MGME1	6	344	0.01744186
TTC13	15	860	0.01744186
SSFA2	22	1259	0.017474186
CAPZA1	5	286	0.017482517
SSR1	5	286	0.017482517
AIM2	6	343	0.017492711
SEC14L3	7	400	0.0175
CGN	21	1197	0.01754386
NAALAD2	13	740	0.017567568
RAB11FIP2	9	512	0.017578125
NTRK1	14	796	0.01758794
PGLYRP3	6	341	0.017595308
SH2D4A	8	454	0.017621145
PRUNE1	8	453	0.017660044
CASQ1	7	396	0.017676768
GABRD	8	452	0.017699115
GP6	6	339	0.017699115
NDUFA7	2	113	0.017699115
TAC4	2	113	0.017699115
RBM19	17	960	0.017708333
C1QBP	5	282	0.017730496
ISPD	8	451	0.017738359
CNDP1	9	507	0.017751479
KDELC2	9	507	0.017751479
NME3	3	169	0.017751479
VMAC	3	169	0.017751479
GGTLC1	4	225	0.017777778
NEK3	9	506	0.017786561
PPM1J	9	505	0.017821782
CHST15	10	561	0.017825312
C1orf168	13	728	0.017857143
CLDN19	4	224	0.017857143
DHRS2	5	280	0.017857143
MYOG	4	224	0.017857143
PAK1IP1	7	392	0.017857143
RRP7A	5	280	0.017857143
TBCK	16	893	0.017917133
NSRP1	10	558	0.017921147
SEC16B	19	1060	0.017924528
PFAS	24	1338	0.01793722
NMBR	7	390	0.017948718
STARD3	8	445	0.017977528
CXCL14	2	111	0.018018018
SERPINA10	8	444	0.018018018
SPACA9	4	222	0.018018018

P2RX4	7	388	0.018041237
TNFAIP6	5	277	0.018050542
GPRC5C	8	441	0.01814059
PIWIL3	16	882	0.01814059
GLDN	10	551	0.01814882
MUS81	10	551	0.01814882
C20orf141	3	165	0.018181818
FOXK2	12	660	0.018181818
HS6ST2	11	605	0.018181818
STARD6	4	220	0.018181818
TOMM7	1	55	0.018181818
SDSL	6	329	0.018237082
NQO1	5	274	0.018248175
EMILIN3	14	766	0.018276762
NPHS2	7	383	0.018276762
PRSS12	16	875	0.018285714
DSTYK	17	929	0.018299247
KBTBD8	11	601	0.018302829
DRC7	16	874	0.018306636
ABCF3	13	709	0.018335684
GGTLC2	4	218	0.018348624
SLIRP	2	109	0.018348624
ABRA	7	381	0.018372703
SHCBP1L	12	653	0.018376723
OLIG3	5	272	0.018382353
FAM167B	3	163	0.018404908
LILRA1	9	489	0.018404908
TMEM255B	6	326	0.018404908
ASPN	7	380	0.018421053
MMS19	19	1030	0.018446602
MLXIP	17	919	0.018498368
OR5A2	6	324	0.018518519
STK32C	9	486	0.018518519
EIF5	8	431	0.018561485
P2RY2	7	377	0.018567639
ACTRT1	7	376	0.018617021
EID3	3	161	0.01863354
EPYC	6	322	0.01863354
MROH8	9	483	0.01863354
SLC24A3	12	644	0.01863354
C7orf31	11	590	0.018644068
ZBP1	8	429	0.018648019
DAPL1	2	107	0.018691589
MFNG	6	321	0.018691589
PTRH1	4	214	0.018691589
TCF3	11	588	0.018707483
CCDC89	7	374	0.018716578

MPHOSPH6	3	160	0.01875
TBXAS1	10	533	0.018761726
PAX3	9	479	0.018789144
MPO	14	745	0.018791946
PPP1R32	8	425	0.018823529
ZNF302	9	478	0.018828452
CROCC	38	2017	0.018839861
ACAA1	8	424	0.018867925
FAM174B	3	159	0.018867925
LCE2A	2	106	0.018867925
OR4D5	6	318	0.018867925
TAS2R7	6	318	0.018867925
TRAPPC6A	3	159	0.018867925
TTC24	11	582	0.018900344
CPVL	9	476	0.018907563
DCDC2	9	476	0.018907563
LRTM2	7	370	0.018918919
DNPEP	9	475	0.018947368
GDNF	4	211	0.018957346
FAM187B	7	369	0.01897019
UGT2A1	10	527	0.018975332
SPTY2D1	13	685	0.018978102
MIP	5	263	0.019011407
TAS1R1	16	841	0.01902497
ABHD14B	4	210	0.019047619
WDR83	6	315	0.019047619
1-Sep	7	367	0.019073569
EMC10	5	262	0.019083969
DACT1	16	836	0.019138756
NTSR1	8	418	0.019138756
GAB4	11	574	0.019163763
C8orf58	7	365	0.019178082
IGFN1	24	1251	0.019184652
ABHD15	9	468	0.019230769
AKAP7	2	104	0.019230769
APC2	9	468	0.019230769
CDKN2A	3	156	0.019230769
DCK	5	260	0.019230769
IFNLR1	10	520	0.019230769
KRTAP19-2	1	52	0.019230769
NOD2	20	1040	0.019230769
RAB6A	4	208	0.019230769
RNASE13	3	156	0.019230769
TMEM199	4	208	0.019230769
MPP5	13	675	0.019259259
RPP40	7	363	0.019283747
DKK2	5	259	0.019305019

FOXO3	13	673	0.019316493
IFNK	4	207	0.019323671
FOXN4	10	517	0.01934236
MKNK2	9	465	0.019354839
C6orf132	23	1188	0.019360269
H4C2	2	103	0.019417476
PRM3	2	103	0.019417476
S100A16	2	103	0.019417476
TRMT11	9	463	0.019438445
C12orf42	7	360	0.019444444
ZFAND2B	5	257	0.019455253
AP1S3	3	154	0.019480519
EIF5AL1	3	154	0.019480519
SIM2	13	667	0.019490255
DPYSL5	11	564	0.019503546
CLIC5	8	410	0.019512195
PRDX3	5	256	0.01953125
CEBPA	7	358	0.019553073
APLF	10	511	0.019569472
ATP5E	1	51	0.019607843
C15orf40	3	153	0.019607843
CA9	9	459	0.019607843
DAP	2	102	0.019607843
TOE1	10	510	0.019607843
KIAA1024	18	916	0.019650655
NEUROD1	7	356	0.019662921
MLLT1	11	559	0.019677996
BPIFA3	5	254	0.019685039
GSDMC	10	508	0.019685039
MBP	6	304	0.019736842
ENTHD1	12	607	0.019769357
AKAP2	17	859	0.019790454
C1orf189	2	101	0.01980198
SAMD10	4	202	0.01980198
TRABD2A	10	505	0.01980198
TMPO	9	454	0.019823789
GABRA6	9	453	0.01986755
MMP17	12	603	0.019900498
NKAPL	8	402	0.019900498
CCDC173	11	552	0.019927536
PSMB11	6	300	0.02
RP2	7	350	0.02
SOAT1	11	550	0.02
B4GALNT3	20	998	0.02004008
GSG1	7	349	0.020057307
ELOVL5	6	299	0.02006689
C1orf50	4	199	0.020100503

IL11	4	199	0.020100503
MS4A6A	5	248	0.02016129
DRC1	10	495	0.02020202
LRMDA	4	198	0.02020202
CMA1	5	247	0.020242915
TLCD1	5	247	0.020242915
PITRM1	21	1037	0.020250723
LDLRAD3	7	345	0.020289855
HNRNPLL	11	542	0.020295203
CLEC3A	4	197	0.020304569
FAM110A	6	295	0.020338983
IPPK	10	491	0.020366599
SEMA6A	21	1030	0.02038835
GORASP1	9	440	0.020454545
SOCS7	9	440	0.020454545
LSS	15	732	0.020491803
FAM151A	12	585	0.020512821
NRSN1	4	195	0.020512821
COX6A2	2	97	0.020618557
RNF157	14	679	0.020618557
TMEM41B	6	291	0.020618557
ETV4	10	484	0.020661157
FTMT	5	242	0.020661157
HOXD8	6	290	0.020689655
ZNF740	4	193	0.020725389
RPS6KB2	10	482	0.020746888
ST6GAL2	11	529	0.020793951
CYSRT1	3	144	0.020833333
RAD51B	8	384	0.020833333
TMEM170A	3	144	0.020833333
AK2	5	239	0.020920502
ABR	18	859	0.020954598
ENPP5	10	477	0.020964361
DUSP6	8	381	0.020997375
CLNK	9	428	0.021028037
BTN2A2	11	523	0.021032505
RORA	11	523	0.021032505
DEFB132	2	95	0.021052632
SMIM18	2	95	0.021052632
STAU2	12	570	0.021052632
CD300LG	7	332	0.021084337
RAB33A	5	237	0.021097046
SAXO1	10	474	0.021097046
MINDY4	16	757	0.021136063
FMN1	30	1419	0.021141649
S1PR3	8	378	0.021164021
SELENOS	4	189	0.021164021

PAICS	9	425	0.021176471
FBXW5	12	566	0.021201413
ASB3	11	518	0.021235521
DSC1	19	894	0.021252796
ATP5G2	3	141	0.021276596
NUPL2	9	423	0.021276596
RAD51C	8	376	0.021276596
TFF3	2	94	0.021276596
IL12B	7	328	0.021341463
RBM11	6	281	0.021352313
ACOT4	9	421	0.021377672
SNX21	8	373	0.021447721
TTF2	8	373	0.021447721
BTRC	13	605	0.021487603
NUMB	14	651	0.021505376
STX16	7	325	0.021538462
JMJD4	10	463	0.021598272
PRF1	12	555	0.021621622
NQO2	5	231	0.021645022
HYI	6	277	0.02166065
BFSP2	9	415	0.021686747
MLN	1	46	0.02173913
MSS51	10	460	0.02173913
NOS2	3	138	0.02173913
RAX2	4	184	0.02173913
RP1	8	368	0.02173913
TUB	11	506	0.02173913
TREML2	7	321	0.021806854
SKA3	9	412	0.02184466
ERAP2	21	960	0.021875
DSE	21	958	0.021920668
SLC18B1	10	456	0.021929825
KITLG	6	273	0.021978022
POU4F2	9	409	0.02200489
C1GALT1C1	7	318	0.022012579
MTUS1	28	1270	0.022047244
IRGM	4	181	0.022099448
SLFNL1	9	407	0.022113022
TRIM51	10	452	0.022123894
ELMO3	16	720	0.022222222
GJB3	6	270	0.022222222
SMCO3	5	225	0.022222222
DDRGK1	7	314	0.022292994
OR52K2	7	314	0.022292994
ADAD2	13	583	0.022298456
DAGLB	15	672	0.022321429
FOXQ1	9	403	0.022332506

UBE2C	4	179	0.022346369
ARHGEF25	13	580	0.022413793
OR2L5	7	312	0.022435897
VASH2	8	355	0.022535211
SPATA6	11	488	0.022540984
COQ4	6	265	0.022641509
OR4S1	7	309	0.022653722
TSKU	8	353	0.02266289
P2RX3	9	397	0.022670025
ADRA1A	13	572	0.022727273
CCR5	8	352	0.022727273
CDKN2A	3	132	0.022727273
JMJD8	6	264	0.022727273
PMP2	3	132	0.022727273
PRND	4	176	0.022727273
SETD4	10	440	0.022727273
TTC21A	30	1320	0.022727273
ZC3H12D	12	527	0.022770398
KRT8	11	483	0.022774327
MYADML2	7	307	0.022801303
PDE7A	11	482	0.022821577
CLDN20	5	219	0.02283105
MOXD1	14	613	0.022838499
FOXL2NB	4	175	0.022857143
LBP	11	481	0.022869023
SCARF1	19	830	0.022891566
KLHDC9	8	349	0.022922636
NKPD1	14	610	0.02295082
AK8	11	479	0.022964509
CRYGA	4	174	0.022988506
TEAD3	10	435	0.022988506
TMED3	5	217	0.023041475
DHRS11	6	260	0.023076923
TBX22	12	520	0.023076923
ABCB8	17	735	0.023129252
SLC39A12	16	691	0.023154848
TTC29	11	475	0.023157895
QRFPR	10	431	0.023201856
HABP2	13	560	0.023214286
KCNE1	3	129	0.023255814
SFT2D3	5	215	0.023255814
SUMF2	7	301	0.023255814
EHF	7	300	0.023333333
PCTP	5	214	0.023364486
F2RL3	9	385	0.023376623
CAPZA3	7	299	0.023411371
TCN2	10	427	0.023419204

EPHX2	13	555	0.023423423
HIST1H2AH	3	128	0.0234375
LARP4	17	724	0.023480663
PNLIPRP3	11	467	0.023554604
SMAP1	11	467	0.023554604
BOK	5	212	0.023584906
PPIC	5	212	0.023584906
ARSD	2	593	0.023608769
KRT85	12	507	0.023668639
MDH2	8	338	0.023668639
TP53RK	6	253	0.023715415
TMEM61	5	210	0.023809524
CTSH	8	335	0.023880597
OR2AK2	8	335	0.023880597
FAM163A	4	167	0.023952096
TCAP	4	167	0.023952096
MAP1LC3B	3	125	0.024
SDHAF3	3	125	0.024
PRKAG1	8	331	0.024169184
INSC	14	579	0.02417962
FAM133A	6	248	0.024193548
GDF1	9	372	0.024193548
IAH1	6	248	0.024193548
SPIC	6	248	0.024193548
STPG4	6	248	0.024193548
PGAM5	7	289	0.024221453
SELENOF	4	165	0.024242424
TLX1	8	330	0.024242424
CYP2A7	12	494	0.024291498
ALDH5A1	13	535	0.024299065
FLG	20	822	0.0243309
DEFB118	3	123	0.024390244
IQCF2	4	164	0.024390244
OR13A1	8	328	0.024390244
SIGIRR	10	410	0.024390244
TMEM234	4	164	0.024390244
RP1	8	327	0.024464832
TUBAL3	11	446	0.024663677
CTNNBIP1	2	81	0.024691358
CTXN2	2	81	0.024691358
OR52I1	8	324	0.024691358
PET117	2	81	0.024691358
IST1	9	364	0.024725275
AKR1C3	8	323	0.024767802
SLC25A27	8	323	0.024767802
DNASE1	7	282	0.024822695
ENDOV	7	282	0.024822695

VTCN1	7	282	0.024822695
MORN5	4	161	0.02484472
COL9A3	17	684	0.024853801
AVEN	9	362	0.024861878
DPT	5	201	0.024875622
NT5C	5	201	0.024875622
KLHDC10	11	442	0.024886878
DNAJC4	6	241	0.024896266
TYMP	12	482	0.024896266
ETV3L	9	361	0.024930748
FAM229B	2	80	0.025
NRG1	16	640	0.025
OR52N1	8	320	0.025
R3HCC1	11	440	0.025
OCA2	21	838	0.025059666
CRACR2B	10	399	0.025062657
TRAP1	10	399	0.025062657
ABHD12	10	398	0.025125628
LIPN	10	398	0.025125628
TAS2R60	8	318	0.025157233
CCDC130	10	396	0.025252525
OR10H4	8	316	0.025316456
WDR88	12	472	0.025423729
BCAM	16	628	0.025477707
OR10A6	8	314	0.025477707
OR2W3	8	314	0.025477707
CRELD2	9	353	0.025495751
CRYBA4	5	196	0.025510204
PGPEP1L	5	196	0.025510204
ANGPTL6	12	470	0.025531915
HOXC6	6	235	0.025531915
ASB2	15	587	0.025553663
DHX35	18	703	0.025604552
CIAPIN1	8	312	0.025641026
PKIB	2	78	0.025641026
PPARA	12	468	0.025641026
ZNF732	15	585	0.025641026
LRIT1	16	623	0.025682183
CCT3	14	545	0.025688073
EBLN2	7	272	0.025735294
EXOSC6	7	272	0.025735294
TRUB1	9	349	0.025787966
RIOX2	12	465	0.025806452
SREK1IP1	4	155	0.025806452
LRRC56	14	542	0.025830258
CYLC2	9	348	0.025862069
HFE	9	348	0.025862069

OR8U1	8	309	0.025889968
SLC7A6OS	8	309	0.025889968
TAS2R8	8	309	0.025889968
PWP1	13	501	0.025948104
FAM240A	2	77	0.025974026
CPN1	14	537	0.026070764
ANXA9	9	345	0.026086957
CHRNB4	13	498	0.026104418
DNAAF4	11	420	0.026190476
BPIFB2	12	458	0.026200873
CHGA	12	457	0.026258206
DNAJC17	8	304	0.026315789
KAZALD1	8	304	0.026315789
METTL23	5	190	0.026315789
PKP3	21	797	0.026348808
PLEKHG7	10	379	0.026385224
PRDM7	13	492	0.026422764
APOD	5	189	0.026455026
HEBP1	5	189	0.026455026
UGT2B11	14	529	0.026465028
CYB5D2	7	264	0.026515152
TLCD2	7	264	0.026515152
UGT2B11	14	528	0.026515152
ACTRT2	10	377	0.026525199
SLC10A6	10	377	0.026525199
EEF1AKMT3	6	226	0.026548673
YY1	11	414	0.026570048
NCF2	14	526	0.02661597
F2RL2	10	374	0.026737968
SNAPC3	11	411	0.02676399
HMGB4	5	186	0.02688172
ACOT2	13	483	0.026915114
ENDOG	8	297	0.026936027
MBP	6	222	0.027027027
PCDHB16	21	776	0.027061856
ZNF404	15	552	0.027173913
B3GALNT1	9	331	0.027190332
C5orf49	4	147	0.027210884
UBE2D4	4	147	0.027210884
HOXC13	9	330	0.027272727
SDR39U1	8	293	0.027303754
CFAP206	17	622	0.02733119
NANOS1	8	292	0.02739726
PET100	2	73	0.02739726
ACOXL	15	547	0.027422303
RTL10	10	364	0.027472527
POPDC3	8	291	0.027491409

ACTL7A	12	435	0.027586207
PLA2G2D	4	145	0.027586207
GFAP	12	432	0.027777778
SDHAF4	3	108	0.027777778
STOM	8	288	0.027777778
TMEM139	6	216	0.027777778
CALHM2	9	323	0.027863777
ADRA1A	13	466	0.027896996
NACA2	6	215	0.027906977
IQCF6	3	107	0.028037383
KCTD17	9	321	0.028037383
TRIM22	14	498	0.02811245
OR10J1	9	320	0.028125
ARSB	15	533	0.028142589
HBQ1	4	142	0.028169014
NINJ2	4	142	0.028169014
DYDC2	5	177	0.028248588
OTC	10	354	0.028248588
SLC39A8	13	460	0.02826087
OPALIN	4	141	0.028368794
PDLIM2	10	352	0.028409091
ALDH4A1	16	563	0.028419183
H2BFWT	5	175	0.028571429
KRTAP26-1	6	210	0.028571429
RNF151	7	245	0.028571429
TMPRSS3	13	454	0.028634361
GMPR2	10	348	0.028735632
OR52E6	9	313	0.028753994
SLC35E3	9	313	0.028753994
TIMM44	13	452	0.028761062
TESPA1	15	521	0.028790787
IGSF22	26	903	0.028792913
OR1L6	10	347	0.028818444
OR2M5	9	312	0.028846154
OR51I2	9	312	0.028846154
CPEB2	17	589	0.028862479
DGUOK	8	277	0.028880866
NOS3	5	173	0.028901734
TCF7L1	17	588	0.028911565
ATP5I	2	69	0.028985507
KRTAP27-1	6	207	0.028985507
OSGEPL1	12	414	0.028985507
PEX11G	7	241	0.029045643
CACNG5	8	275	0.029090909
MRPL22	6	206	0.029126214
OR7A17	9	309	0.029126214
SPATA13	19	652	0.029141104

TMEM82	10	343	0.029154519
DBH	18	617	0.02917342
OR5K1	9	308	0.029220779
OR8D1	9	308	0.029220779
ANKLE1	18	615	0.029268293
ABRAXAS1	12	409	0.029339853
NOC2L	22	749	0.029372497
CADM1	13	442	0.029411765
FBXO39	13	442	0.029411765
ADRM1	12	407	0.029484029
APOBEC1	7	236	0.029661017
ANXA11	15	505	0.02970297
TMPRSS3	13	437	0.029748284
APOL3	12	402	0.029850746
GPR157	10	335	0.029850746
OLFML1	12	402	0.029850746
R3HDM4	8	268	0.029850746
ZG16	5	167	0.02994012
GUCA1B	6	200	0.03
LACTBL1	15	500	0.03
IFITM3	4	133	0.030075188
IZUMO4	7	232	0.030172414
PSRC1	11	363	0.03030303
CCDC69	9	296	0.030405405
INMT	8	263	0.030418251
RTP1	8	263	0.030418251
FCHSD1	21	690	0.030434783
NUDT17	10	328	0.030487805
KLK1	8	262	0.030534351
TOR1AIP2	4	131	0.030534351
CPN1	14	458	0.030567686
BCL2L14	10	327	0.03058104
IER5	10	327	0.03058104
OR51T1	10	327	0.03058104
OR5T2	11	359	0.030640669
DTHD1	24	781	0.030729834
SMKR1	2	65	0.030769231
GFI1	13	422	0.030805687
IDI1	7	227	0.030837004
AK1	6	194	0.030927835
OR52N4	10	321	0.031152648
KRT31	13	416	0.03125
SLC25A45	9	288	0.03125
ACOT11	19	607	0.031301483
AMBN	14	447	0.031319911
FAM83E	15	478	0.031380753
ELSPBP1	7	223	0.031390135

WWOX	13	414	0.031400966
PRPS1L1	10	318	0.031446541
ELMOD3	12	381	0.031496063
FAM98C	11	349	0.031518625
FAM166A	10	317	0.031545741
NOB1	13	412	0.031553398
SRSF9	7	221	0.031674208
LRP5L	8	252	0.031746032
POP4	7	220	0.031818182
TERB2	7	220	0.031818182
CCL26	3	94	0.031914894
C17orf62	6	187	0.032085561
CDNF	6	187	0.032085561
LMOD3	18	560	0.032142857
SLC1A7	18	560	0.032142857
KRT33B	13	404	0.032178218
KNCN	4	124	0.032258065
FN3KRP	10	309	0.03236246
RFLNA	7	216	0.032407407
CLHC1	19	586	0.032423208
LDLRAP1	10	308	0.032467532
SLC22A1	18	554	0.032490975
GKN2	6	184	0.032608696
TAAR5	11	337	0.03264095
ISX	8	245	0.032653061
TPMT	8	245	0.032653061
DUSP1	12	367	0.032697548
CCDC68	11	335	0.032835821
CD48	8	243	0.032921811
BTN3A2	11	334	0.032934132
TMCO2	6	182	0.032967033
SULT6B1	10	303	0.0330033
CINP	7	212	0.033018868
DLG1	30	904	0.033185841
AKR7L	11	331	0.033232628
CD7	8	240	0.033333333
PPM1E	9	270	0.033333333
SHARPIN	13	387	0.033591731
NDUFAF1	11	327	0.033639144
THEM6	7	208	0.033653846
FRZB	11	325	0.033846154
ADPRHL1	12	354	0.033898305
KRT14	16	472	0.033898305
OR1J1	11	322	0.034161491
ZNF883	13	379	0.034300792
C17orf50	6	174	0.034482759
MRPS21	3	87	0.034482759

SLC10A2	12	348	0.034482759
CASP5	15	434	0.034562212
APEX1	11	318	0.034591195
PRKAG3	17	489	0.034764826
EXO5	13	373	0.034852547
HTT	22	630	0.034920635
CCDC28B	7	200	0.035
OR4D6	11	314	0.035031847
TEKT5	17	485	0.035051546
OR5D18	11	313	0.03514377
OR8B3	11	313	0.03514377
OR1F1	11	312	0.03525641
DNAJA4	14	397	0.035264484
CLYBL	12	340	0.035294118
SIGLEC12	21	595	0.035294118
GJB2	8	226	0.03539823
RAB21	8	225	0.035555556
3-Mar	9	253	0.035573123
P2RY4	13	365	0.035616438
KRT37	16	449	0.035634744
CLPS	4	112	0.035714286
CMPK1	7	196	0.035714286
SPINT2	9	252	0.035714286
TPD52	8	224	0.035714286
OR13G1	11	307	0.035830619
C7orf50	7	194	0.036082474
KLK13	10	277	0.036101083
IMMP1L	6	166	0.036144578
PPIL1	6	166	0.036144578
REG1B	6	166	0.036144578
CYB5R2	10	276	0.036231884
ARL2BP	6	163	0.036809816
C11orf40	8	217	0.036866359
ABHD14A	10	271	0.036900369
ATF1	10	271	0.036900369
ITLN2	12	325	0.036923077
FAM162B	6	162	0.037037037
HSD17B14	10	270	0.037037037
SYF2	9	243	0.037037037
TST	11	297	0.037037037
SYCE1L	9	242	0.037190083
MBLAC1	10	266	0.037593985
CHRNA10	17	450	0.037777778
OR52E8	12	317	0.03785489
PRG4	13	343	0.037900875
TAF11	8	211	0.037914692
CHST6	15	395	0.037974684

OR4C15	12	316	0.037974684
ROMO1	3	79	0.037974684
TNNI3	8	210	0.038095238
GNB3	13	340	0.038235294
CMTM6	7	183	0.038251366
KCNK6	12	313	0.038338658
SPATA21	18	469	0.038379531
APMAP	16	416	0.038461538
FGL1	12	312	0.038461538
SMIM1	3	78	0.038461538
SMIM24	5	130	0.038461538
RRP36	10	259	0.038610039
SCRN1	16	414	0.038647343
LEMD1	7	181	0.038674033
RBM23	17	439	0.038724374
DOK2	16	412	0.038834951
DCAKD	9	231	0.038961039
HEBP2	8	205	0.03902439
C6orf229	9	230	0.039130435
CCDC50	12	306	0.039215686
MRPL57	4	102	0.039215686
PARP2	23	583	0.039451115
C1QTNF4	13	329	0.039513678
C6orf226	4	101	0.03960396
PUSL1	12	303	0.03960396
RNF186	9	227	0.039647577
TMEM238	7	176	0.039772727
AIF1L	6	150	0.04
AKR1A1	13	325	0.04
CAPS	11	275	0.04
FAM166B	11	275	0.04
LIMS1	13	325	0.04
ZNF784	13	323	0.040247678
TTC23	18	447	0.040268456
MIP	5	124	0.040322581
TMEM54	9	222	0.040540541
BOD1L2	7	172	0.040697674
CCDC3	11	270	0.040740741
CFHR2	11	270	0.040740741
SNTN	6	147	0.040816327
TNFAIP8L3	12	292	0.04109589
DEFA4	4	97	0.041237113
NPY	4	97	0.041237113
OR10A4	13	315	0.041269841
OR5B3	13	314	0.041401274
OR5F1	13	314	0.041401274
OR51A4	13	313	0.041533546

MTRNR2L3	1	24	0.041666667
MTRNR2L9	1	24	0.041666667
CRYBA1	9	215	0.041860465
EDARADD	9	215	0.041860465
CIDEC	10	238	0.042016807
C12orf65	7	166	0.042168675
METTL11B	12	283	0.042402827
BUB1B-PAK6	6	141	0.042553191
C7orf73	2	47	0.042553191
ACTR3C	9	210	0.042857143
DEFB128	4	93	0.043010753
SERPINA1	18	418	0.043062201
VWC2	14	325	0.043076923
ZSCAN9	17	394	0.043147208
KRTAP4-8	8	185	0.043243243
NOL3	9	208	0.043269231
KRTAP13-4	7	160	0.04375
THAP8	12	274	0.04379562
C12orf49	9	205	0.043902439
C20orf196	9	205	0.043902439
SBDS	11	250	0.044
CYTL1	6	136	0.044117647
PCP4L1	3	68	0.044117647
RPL3L	18	407	0.044226044
NATD1	5	113	0.044247788
BLOC1S3	9	202	0.044554455
RFESD	7	157	0.044585987
ETFBKMT	12	262	0.045801527
GNB1L	15	327	0.04587156
MPC1	5	109	0.04587156
EVA1A	7	152	0.046052632
FANK1	16	345	0.046376812
LRRC3C	13	275	0.047272727
MVB12A	13	273	0.047619048
IGFBP5	13	272	0.047794118
KRTAP12-2	7	146	0.047945205
NAA38	6	125	0.048
L3HYPDH	17	354	0.048022599
OR10V1	15	309	0.048543689
OR5D16	16	328	0.048780488
TYMSOS	6	123	0.048780488
PTPMT1	10	201	0.049751244
DUSP27	11	220	0.05
KIAA1644	10	199	0.050251256
KRTAP5-9	12	238	0.050420168
ETFB	13	255	0.050980392
OR2C1	16	312	0.051282051

TBL2	23	447	0.051454139
IGLL1	11	213	0.051643192
HMOX1	15	288	0.052083333
COPS9	3	57	0.052631579
TMEM123	11	208	0.052884615
AGRP	7	132	0.053030303
C1orf210	6	113	0.053097345
CFAP77	17	320	0.053125
PABPN1L	15	278	0.053956835
TEP1	22	403	0.054590571
OTOR	7	128	0.0546875
APOH	19	345	0.055072464
NEUROG3	12	214	0.056074766
FAM25A	5	89	0.056179775
C19orf33	6	106	0.056603774
CALCA	8	141	0.056737589
LYRM2	5	88	0.056818182
NDUFA1	4	70	0.057142857
MRPL36	6	103	0.058252427
PRSS38	19	326	0.058282209
SSX7	11	188	0.058510638
DDIT3	2	34	0.058823529
TSPAN10	21	355	0.05915493
LAIR2	9	152	0.059210526
S100A3	6	101	0.059405941
DEFB119	5	84	0.05952381
DUSP13	12	198	0.060606061
GTSF1L	9	148	0.060810811
IL17F	10	163	0.061349693
PSCA	7	114	0.061403509
PAGE5	8	130	0.061538462
CRYBB3	13	211	0.061611374
CCDC122	17	273	0.062271062
CALCA	8	128	0.0625
EFCAB8	9	144	0.0625
C12orf54	8	127	0.062992126
DUSP13	12	188	0.063829787
VPS37C	23	355	0.064788732
MEA1	12	185	0.064864865
C1orf194	11	169	0.065088757
C16orf74	5	76	0.065789474
OR4D9	21	314	0.066878981
FXYD4	6	89	0.06741573
POP1	6	89	0.06741573
CENPW	6	88	0.068181818
RLF	9	131	0.06870229
MRPS34	15	218	0.068807339

MC1R	22	317	0.069400631
KRTAP5-9	12	169	0.071005917
NRTN	14	197	0.07106599
ODF3B	18	253	0.071146245
C14orf177	9	125	0.072
OR10G3	23	313	0.073482428
FKBP9	8	108	0.074074074
LYZ	11	148	0.074324324
DSC1	19	252	0.075396825
NMU	14	174	0.08045977
PLAC9	8	97	0.082474227
MIEF1	6	70	0.085714286
LECT2	13	151	0.086092715
PHLDA3	11	127	0.086614173
KISS1	12	138	0.086956522
TSACC	11	125	0.088
APC2	9	101	0.089108911
SPANXN4	9	99	0.090909091
GJB4	25	266	0.093984962
DLC1	9	89	0.101123596
GPHB5	14	130	0.107692308
TMEM254	14	123	0.113821138
GNGT2	8	69	0.115942029
POP4	7	45	0.155555556
ARMCX5-GPRASP2	3	NA	NA
C7orf55-LUC7L2	1	NA	NA
C8orf44-SGK3	2	NA	NA
CNPY3-GNMT	1	NA	NA
FPGT-TNNI3K	6	NA	NA
GLRA4	1	NA	NA
JMJD7-PLA2G4B	3	NA	NA
LOC100131107	2	NA	NA
LOC100134391	4	NA	NA
LOC100507507	1	NA	NA
LOC100996693	2	NA	NA
LOC102724428	1	NA	NA
LOC283710	1	NA	NA
LOC388780	10	NA	NA
LOC729159	1	NA	NA
